# Automated Rational Design of Metal–Organic Polyhedra

**DOI:** 10.1021/jacs.2c03402

**Published:** 2022-06-22

**Authors:** Aleksandar Kondinski, Angiras Menon, Daniel Nurkowski, Feroz Farazi, Sebastian Mosbach, Jethro Akroyd, Markus Kraft

**Affiliations:** † Department of Chemical Engineering and Biotechnology, University of Cambridge, Philippa Fawcett Drive, Cambridge CB3 0AS, U.K.; ‡ CMCL Innovations, Sheraton House, Castle Park, Cambridge CB3 0AX, U.K.; § CARES, Cambridge Centre for Advanced Research and Education in Singapore, 1 Create Way, CREATE Tower, #05-05, Singapore 138602; ∥ School of Chemical and Biomedical Engineering, Nanyang Technological University, 62 Nanyang Drive, Singapore 637459; ⊥ The Alan Turing Institute, 2QR, John Dodson House, 96 Euston Road, London NW1 2DB, U.K.

## Abstract

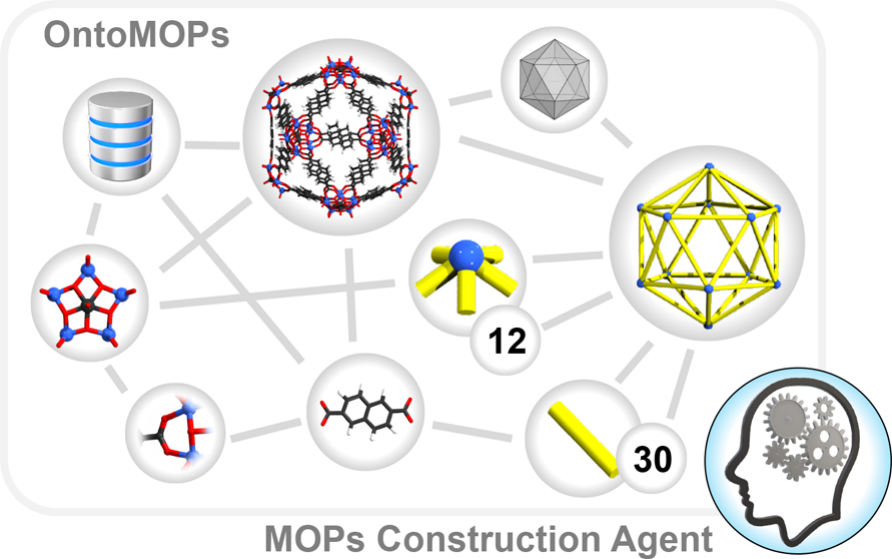

Metal–organic polyhedra (MOPs) are hybrid organic–inorganic nanomolecules, whose rational design depends on harmonious consideration of chemical complementarity and spatial compatibility between two or more types of chemical building units (CBUs). In this work, we apply knowledge engineering technology to automate the derivation of MOP formulations based on existing knowledge. For this purpose we have (i) curated relevant MOP and CBU data; (ii) developed an assembly model concept that embeds rules in the MOP construction; (iii) developed an OntoMOPs ontology that defines MOPs and their key properties; (iv) input agents that populate The World Avatar (TWA) knowledge graph; and (v) input agents that, using information from TWA, derive a list of new constructible MOPs. Our result provides rapid and automated instantiation of MOPs in TWA and unveils the immediate chemical space of known MOPs, thus shedding light on new MOP targets for future investigations.

## Introduction

Molecular engineering is an emerging study of molecular components with the aim of tailoring their programmed assembly toward new and functional materials.^1^ Molecular engineering relies on a cognitive design thinking approach (i.e., rational design), and thus it has shown a strong innovation reliability across multiple domains spanning nanotechnology,^2,3^ molecular machinery,^4^ OLEDs,^5^ flexible solar cells, and other technologies.^6^ A special advancement to molecular engineering has been the conceptualization of building blocks, that is, molecular components that can be developed and reused across different material families. In this regard, the combination of inorganic and organic building units has subsequently led to the flourish of various molecular and functional hybrids such as supramolecular assemblies,^7,8^ hybrid polyoxometalates (POMs),^9,10^ metal–organic polyhedra (MOPs),^11−13^ and also extended reticular systems like metal–organic frameworks (MOFs).^14,15^

**Figure 1:**
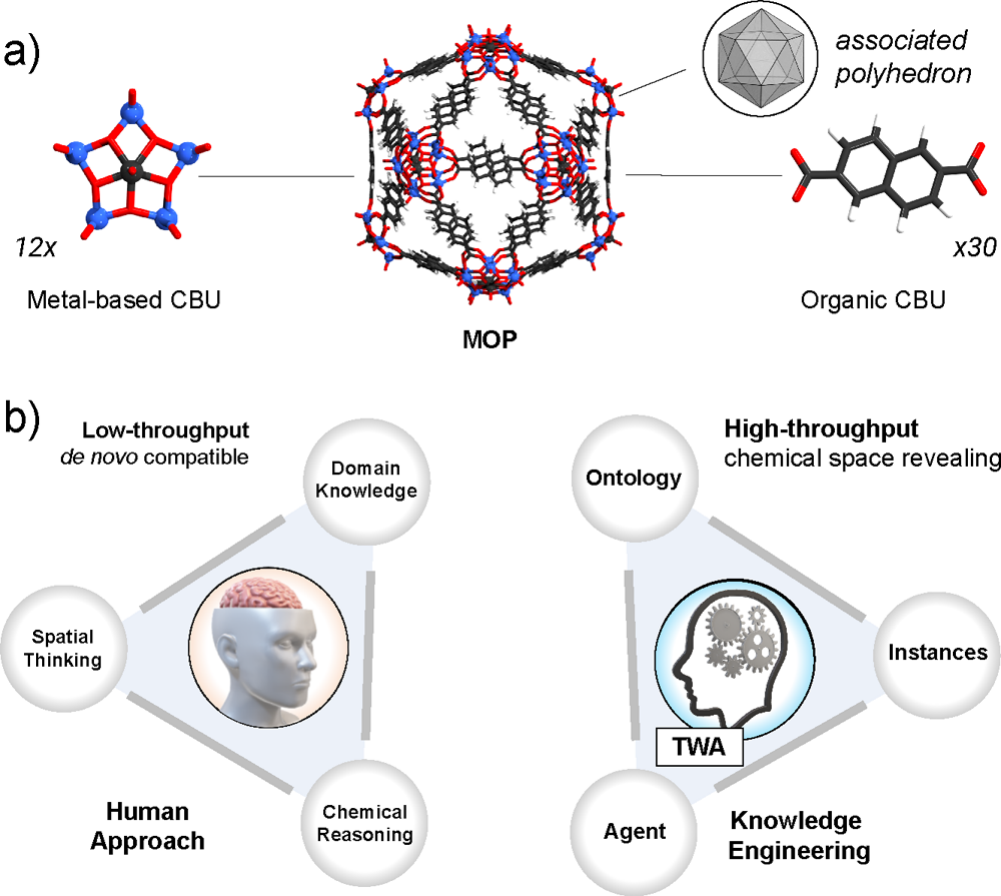
(a) Experimentally reported MOP,^47^ its components, and perceived icosahedral shape. (b) Comparison of the human and the knowledge engineering approach in the context of rational MOP design.

Among the different molecular and nanoscopic hybrids, MOPs are renowned for their virtual adoption of shapes of highly symmetrical polyhedra.^11^ MOPs also share similarities to other more early established hybrids, which may have contributed to their slower comprehensive recognition as a distinct material domain.^12,13,16^ MOPs are typically constructed from a pair of complementary organic and inorganic chemical building units (CBU) as shown in Figure 1a. Cases when more than two CBUs form MOPs are also known.^12^ Similarly to MOFs, the organic building units in MOPs are typically carboxylate-based.^13,17−19^ Owing to the nature of the binding organic functionality, MOPs are occasionally differentiated from other types of supramolecular assemblies.^20^ The inorganic units in MOPs may be monometallic, but they are predominantly bimetallic and multimetallic.^12^ Multimetallic inorganic CBUs may be metal-oxo clusters as POMs.^21^ Like MOFs and other supramolecular cages, MOPs are porous and exhibit internal cavities suitable for molecular guest encapsulation,^13,22^ and gas capture and separation (e.g., CO_2_).^23,24^ The high number of metal centers makes MOPs attractive in catalysis,^25−27^ while their discrete shape and topology makes them suitable nanocomponents for building porous soft materials^28^ and porous salts.^29^

Interested in the development of future AI-driven chemical scientists and laboratories capable of solving emerging real world problems,^30−32^ we envision a tremendous opportunity for the development of new knowledge and logic driven technologies that are capable of emulating different aspects of the expert’s decision making process. Knowledge engineering (KE) is one technology^33^ that efficiently couples ontological representation of key concepts, relational data in a knowledge graph (KG), and logic execution software agents toward a particular goal. (see Figure 1b). In comparison to the widely used database approaches for storage and exploration of chemical data, KGs are based on semantics depicting a complex network of concepts and information, thus they are relatively uncharted territory in chemistry.^34−36^ Over the past decade, KGs have been aiding the elucidation of the relationship between chemical structures and biological responses,^37^ which has an obvious relevance in the development of new pharmaceuticals.^38,39^ KGs can be highly modular and dynamic, and as such their application has become popular cross many different industries.^40,41^ Synergistic use of KGs can be established by interconnecting KGs in an interoperable manner, toward solving a complex goal. This has enabled the creation of a world model called *The World Avatar* (TWA), which potentially comprises any concept, instances of these concepts, and agents that operate on both concepts and instances. Hence, TWA can be viewed as an universal digital twin (UDT).^41,42^ The chemical and process development component of TWA so far contains information on quantum chemistry, chemical species, reaction networks, and experimental observations including agents capable of model calibration and cross domain linkage.^43−46^

The purpose of this work is to expand on the capabilities of *The World Avatar* by developing knowledge graph technologies for the representation and rational design of MOP and projection of their immediate chemical space. To achieve this, we first develop a concept of assembly models to represent the geometric features of a MOP and how it is constructed from its constituent CBUs. These relations between chemical and topological features are encoded via the newly developed “OntoMOPs” ontology representing MOPs in TWA. MOP data have been systematically curated, cleaned, and organized with consideration of their composition and structure. TWA is populated with 151 MOP and 137 CBU instances (see Figures S1–S8 in the SI) with a set of custom built software tools. Finally, a MOP Discovery agent has been developed and used to perform a series of queries and set operations from which it identifies new MOP formulations by considering chemical and spatial compatibility of different CBUs known to build MOPs.

## Methodology

This section clarifies the existing domain uncertainties and reasoning constraints. On the basis of the latter, a rational design with the help of assembly models is being proposed and conceptualized. The knowledge modeling, information curation, algorithm development, and implementation schemes behind the OntoMOPs KG and the MOPs Discovery Agent are consequently described in a stepwise manner.

### Immediate Chemical Space and Its Uncertainties

“How can one design a structure if its “blueprint” is unknown?” is a question that Yaghi and co-workers raise in their recent perspective defining the digital reticular chemistry covering 1-/2-/3-dimensional metal–organic materials.^48^ This overview provides a perspective on how to merge machine learning (ML), database technology, and mechatronics for the automated discovery and development of MOFs. In the work, the authors acknowledge the vastness of chemical space that emerges as a result of building block, topological, and isomeric variability; however, they also emphasize the value of being able to preselect and recognize viable material targets with promising precalculated properties. This is in contrast to the more common material development followed by property description.

In the article, material construction is described as the linking of different building units based on “empirical” knowledge of what the structural outcome might be.^48^ The authors see this approach as having “a heavily reliance on experience” and circumventing this represents an open challenge. However, this empirical knowledge approach also comes with uncertainties, some of which may derive from the synthetic complexity where the reagents likely include additional chemical species not considered in the conceptual modeling, but also due to uncertainties in the expected outcome. Secondary building units “SBUs” that appear compatible with a particular symmetric framework, when actually reacting in a synthetic pathway, may form another unanticipated structure at the end. This can occur because the SBUs may adopt different modularities^48^ during different reactive processes. These uncertainties arising from different modularities are genuine, and they are not unique to MOFs and COFs, but also to MOPs.^12^

**Figure 2:**
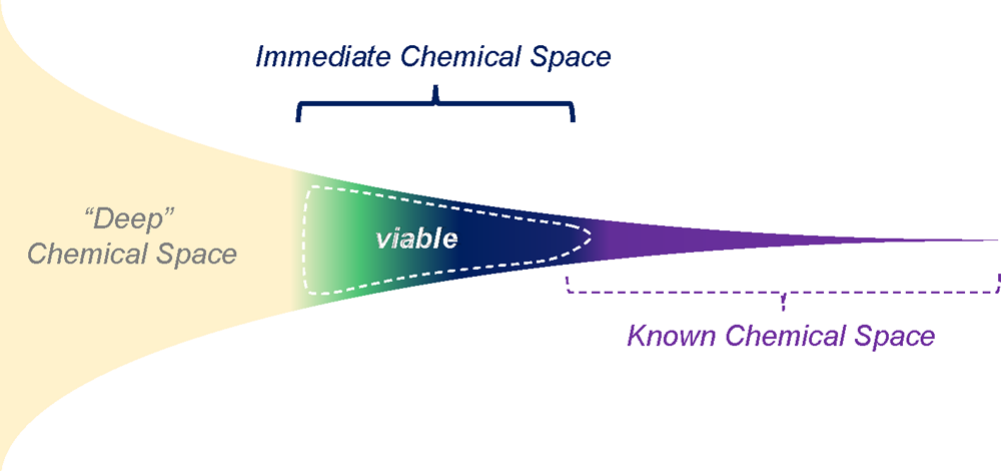
Schematic illustration of the three regions of the chemical space of MOPs: known domain, its immediate chemical space that can be logically constructed, and the uncharted (i.e., deep) chemical space normally “unlocked” by new AM and CBU development.

From a viewpoint of molecular engineering, a key question is how many and what variety of new structures can be constructed based on known building units? Answering this complex question provides (i) a better overview on what new materials are in the immediate vicinity of our current knowledge and (ii) the possibility to estimate the structural uncertainties occurring when a pair of building units can construct more than one structure. An automated approach to this problem suggests potential formulation targets. Molecular modeling and calculations can then be used to predict material properties. This in turn is useful for future targeted synthesis. Consequently, the “immediate chemical space” (ICS) can be unearthed in this way (Figure 2). The ICS is thus predominantly focused on “constructible” topologies without further explicit concern of how many additional constructed derivatives can be combinatorially derived as a function of conformational and configurational variances in the redox, protonation, and chiral nature of the building units. In this view, the ICS is an instance-based projection that at the same time is restrictive, but also pragmatic in terms of molecular engineering.

In contrast to the ML and database approach, which essentially relies on learning from vast amounts of data,^48^ the KE builds on the knowledge and experience of a domain expert and thus new predictions can also be made for domains where data are not vast. The KE approach also provides the possibility to formulate new concepts and assess their value in terms of algorithmic output quality. In this context, we have effectively differentiated between the chemical and geometric nature of Yaghi’s SBU concept,^49^ thus developing a new representation via a chemical building unit “CBU” that functions as a generic (i.e., geometric) building unit “GBU”. Topologically complementary GBUs act as the key components in the construction of assembly models (AMs) that then provide the “blueprints” for the formulation of MOPs based on complementary CBUs related to the starting GBUs (see the Assembly Models section below for more details). By studying the relationship between CBUs, GBUs, AMs, and MOPs we can project the ICS of MOPs. As more than one outcome may be formed when two CBUs interact, we obtain awareness of the uncertainty, which is useful when designing a synthetic approach. When the outcome is a new and an unanticipated MOP, then this structure and its AM are added to the knowledge graph, followed by an update of the ICS in an instance-based manner.

The ICS is part of the overall chemical space, and it connects the known domain (i.e., experimentally verified MOPs) with the uncharted or deep chemical space (Figure 2). The MOP instances of the ICS are rationally designed constructs based on known CBUs, and they can be further computationally modeled (see Comment 1 in the SI). The automated rational proposal of constructible MOPs is not only of synthetic interest, but also in terms of molecular modeling and calculations. Unlike the modeling and calculation of organic cages,^50^ we are unaware of accurate calculations on multimetallic MOPs based on force field methods,^51^ and thus more computationally demanding DFT approaches may be needed.^52^ The latter approach can be very informative in terms of structure and electronic properties, and when a particular target fulfills criteria to be regarded as realistic or “viable”,^53^ the predictions of its properties can be suitable for further selection of technologically relevant targets.^54^

### Assembly Models

#### Polyhedra Modeling during Early Cognitive Development

In contrast to adults, children learn how to think abstractly through sensory input.^55^ Construction of polyhedral and reticular assemblies is an abstract and intellectually challenging topic. However, research with didactic toy-based hands-on manipulatives points to the contrary. Using a generic set of interlocking disks and only the restriction to build symmetrically, children have been shown to be able to construct subcomponents and to assemble them into larger high-symmetry assemblies resembling reticular and polyhedral structures.^56,57^ Children are able to achieve this in the absence of prior mathematical knowledge (e.g., dihedral angles) through playful experimentation with the different subcomponents, leading them to discover assemblies of reticular and polyhedral materials. This motivates the concept of an assembly model (AM) for MOPs, by which a larger structure is assembled from smaller subcomponents, in this case generic building units (GBUs). The assembly model concept also provides a framework of meta-rules for algorithmic discovery of new MOPs, analogous to how children intuitively derive new structures from subcomponents without explicit instruction.

#### Chemical Complementarity

Whether two CBUs are chemically complementary depends on the features of their “binding sites”. In MOPs, the interaction is typically between cationic metal-based CBUs and anionic organic CBUs acting as Lewis acids and bases, respectively. The organic ligands typically are bidentate (carboxylate) ligands, but other modularities may be observed as well. For successful integration in highly symmetrical assemblies, the metal sites also need to connect to the organic ligands in an orderly manner. Finally the local stereochemistry between the binding sites is another important feature. Within MOPs, the binding sites of a pair of complementary CBUs are well aligned with the virtual line connecting the central points of each CBU. This is normally different for many other supramolecular coordination cages where the binding to the metal occurs via sideway-binding pyridyl-imine groups that subsequently generate local *mer*-/*fac*-isomerism.^58^ The basic aspects of chemical complementarity need to be taken into consideration when structures are being algorithmically assembled.

#### Topological Compatibility

Coordination cages comprising single metal nodes (M) and organic bridging ligands (L) are typically noted as M_*x*_L_*y*_ (e.g., M_12_L_24_).^59^ However, the latter notation does not explicitly describe the overall arrangement and may cause ambiguity when describing isomeric topologies such as cuboctahedral and anticuboctahedral M_12_L_24_.^12^ The ambiguity can be eliminated when describing MOPs as polyhedral shapes.^11^ In the latter approach, a particular atom or a moiety is aligned with an element of a polyhedral shape (e.g., corner, edge, or face). However, MOPs can be ideally highly symmetrical molecules (i.e., “Keplerates”),^60^ and so differences in prioritization of one molecular fragment over the other may lead to envisioning more than one single shape, leading to correct but inconsistent shape descriptions.

**Figure 3:**
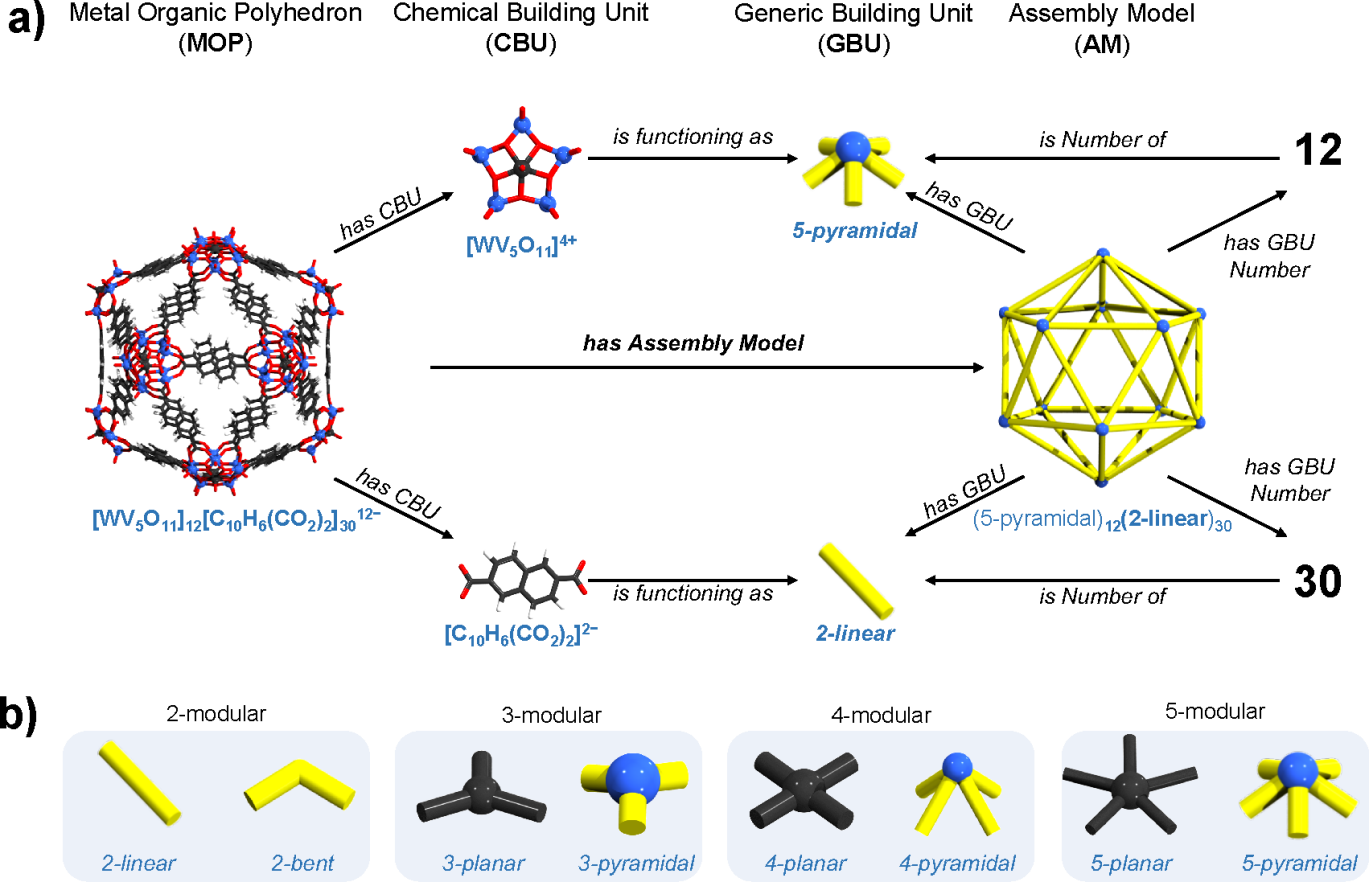
(a) Relations between MOPs, CBUs, GBUs, and assembly models. (b) Four general types of GBUs.

To solve problems with ambiguities and shape inconsistencies, we derived an “assembly model” based approach. In our approach, a MOP is envisioned as a highly symmetrical assembly comprised of a pair of chemical building units (CBUs) appearing in strictly defined numbers. Each CBU shows particular modularity and shape features similar to that of a coordination complex, which we refer to as “planarity”. The combination of modularity and planarity provides a foundation to define a virtual “generic building unit” (i.e., GBU). Similarly, to the CBUs, GBUs appearing in strictly defined numbers can interconnect into larger and virtual Assembly Models (AMs), which in the case of MOPs are polyhedral and cage-like. The AMs come with an ideal symmetry point group and in terms of interconnectivity resemble the MOP. In this way, AMs act as a “construction template” for MOPs. Considering that one needs at least two GBUs to construct an AM, the AM has the advantage to relate to a single shape. An illustration of this is the icosahedral MOP [WV_5_O_11_]_12_[C_10_H_6_(CO_2_)_2_]_30_^12–^, which is comprised of 12 inorganic [WV_5_O_11_]4 + CBUs functioning as “5-pyramidal” GBUs and 30 organic [C_10_H_6_(CO_2_))_2_]_30_^2–^ CBUs functioning as “2-linear” GBUs. The latter MOP has an assembly model (5-pyramidal)_12_(2-linear)_30_ with I_*h*_ symmetry (see Figure 3a).

#### Driving Forces in MOP Self-Assembly

The chemical complementarity and topological compatibility for MOP formation connect to more fundamental natural principles (i.e., mathematics and thermodynamics) that guide MOP assembly. To be able to form discrete assembly models with a particular geometry, at least one GBU in a GBU pair needs to be nonplanar or nonlinear. Further on, the CBUs that construct the MOPs need to exhibit angles between their points of an extension within particular ranges.^61^ With the use of the assembly model concept (i.e., combining GBUs, GBU numbers, and overall symmetry), the explicit reliance on angles has been omitted. The combination of topological compatibility and chemical complementarity enables MOPs to obtain more negative energies of formation, which essentially favors their thermodynamic formation.^62,63^ As MOPs are highly symmetrical and can pack well in crystals, it is also a question to what level the thermodynamics of crystal formation also contributes to their predominant formation and isolation.^64^

#### Derivation of Assembly Models

**Figure 4:**
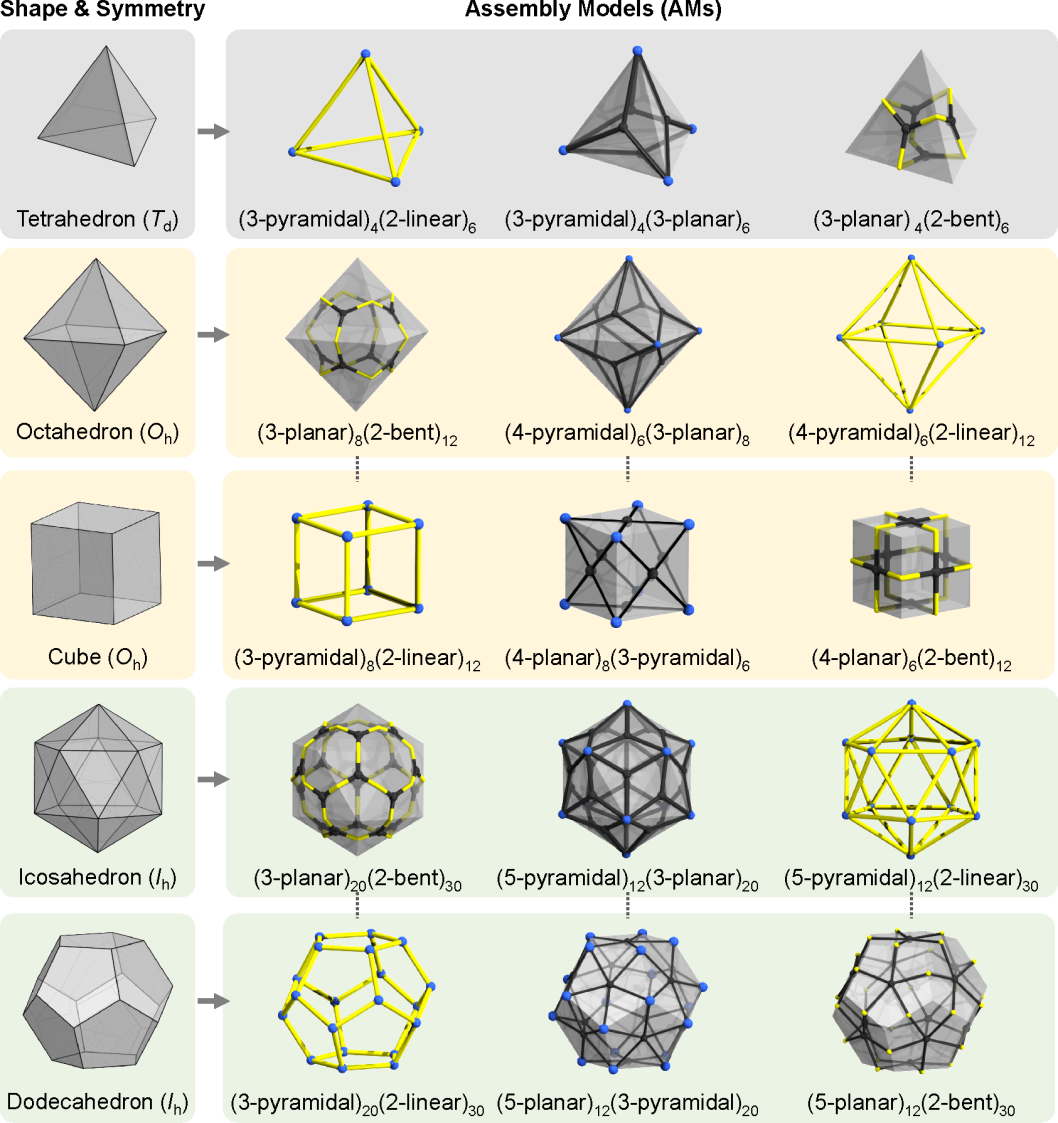
Derivation of assembly models from the shape of the well-known platonic solids.

Solely on the basis of planarity and modularity, one can derive a set of GBUs (see Figure 3b). This set of GBUs is sufficient to build many different AMs resembling different shapes. This is because the GBUs can be abstractly compared to elements of a polyhedron. For example, 2-linear building units derive from edges, while 3-, 4-, and 5-pyramidal GBUs typically act as vertices. On the other hand, the 3-, 4-, and 5-planar GBUs align well with the center of the trigonal, square, and pentagonal faces, respectively. The 2-bent GBUs can be seen as edge-based cross-points connecting planar GBUs from different faces of the polyhedron (see Figure 4).

The derivation of assembly models from the platonic solids provides two additional insights. First, the close interconnection of an AM with a single shape is essential because, most fundamentally, it is not only the building units that define the MOP. In return, the symmetry and shape of the assembly model “softly encode” particular properties of the building units, such as differences in dihedral angles. In this regard, a “3-pyramidal” GBU involved in the construction of a tetrahedral (3-pyramidal)_4_(2-linear)_6_ assembly model is not the same as the “3-pyramidal” GBU involved in the construction of dodecahedral (3-pyramidal)_20_(2-linear)_30_ (i.e., the dihedral increase from 70.52° to 116.56°). Further on, pairs of shapes sharing the same symmetries derive pairs of “inverse” assembly models where the GBU retains its modularity. Still, there is an inversion in terms of planarity (i.e., planar becomes pyramidal, linear becomes bent, and vice versa). One example may be the O_*h*_-symmetric (4-pyramidal)_6_(3-planar)_8_ and (4-planar)_8_(3-pyramidal)_6_ models that derive from an octahedron and cube, respectively. A virtual transformation from such a pair of assembly models goes through yet another (4-pyramidal)_6_(3-pyramidal)_8_ assembly model, whose shape may be traced to the Catalan-type rhombic dodecahedron (vide infra).

### The World Avatar: OntoMOPs

#### MOP Discovery as Part of a Digital Ecosystem

Pragmatic multiscale material development connecting lab-scale to industrial-scale production relies on accurate life cycle assessment.^65^ In the context of digital transformation, the latter is a real cross-domain world problem that can be virtually represented by a universal digital twin. The universal digital twin receives an influx of knowledge and operates through a complex network of concepts, relationships, and synergetic software agents that simulate and analyze different what-if scenarios, based on which decisions are made and implemented.^42,66^

**Figure 5:**
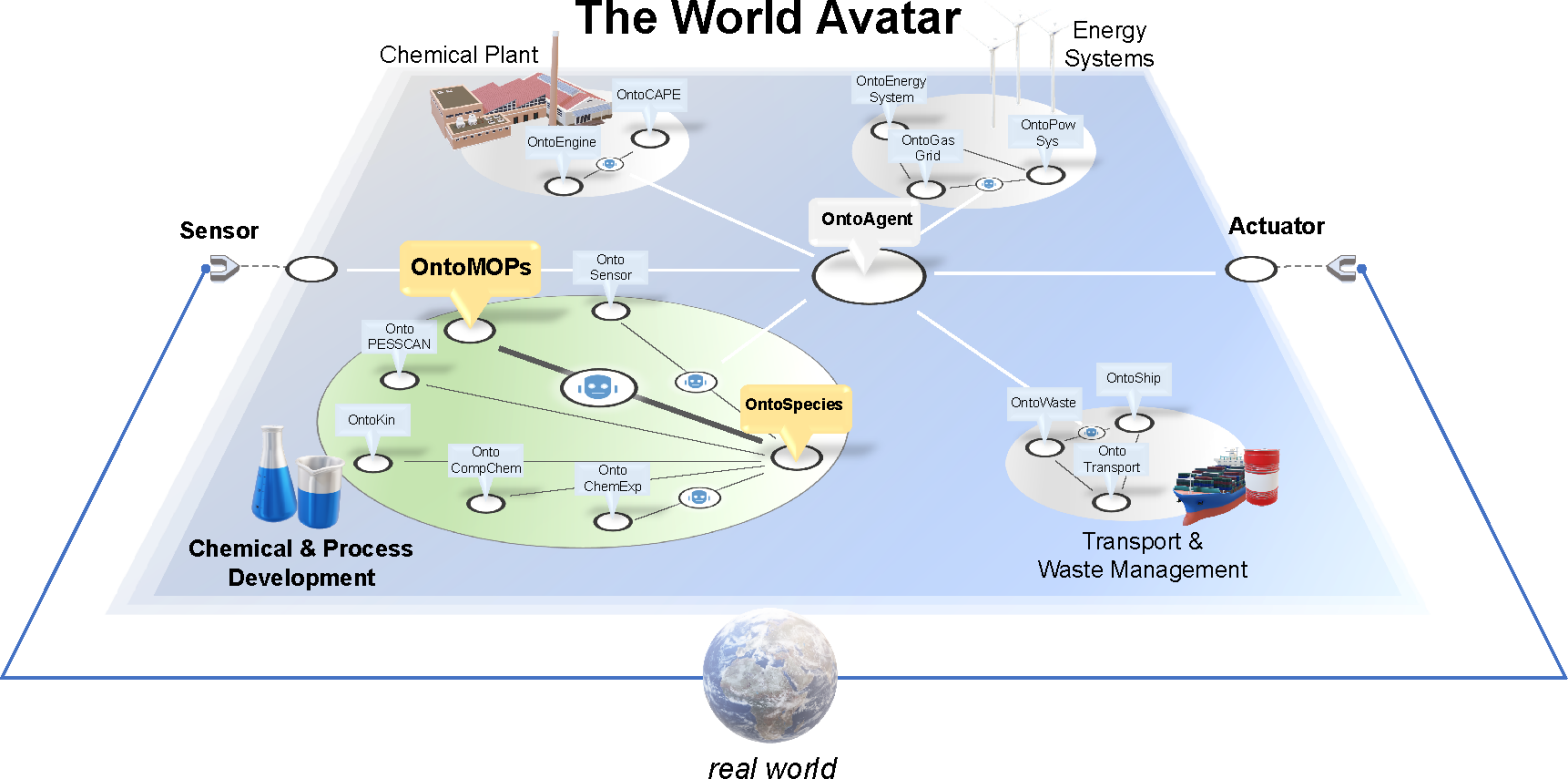
A selection of ontologies and their connectivity that have been integrated in TWA. OntoMOPs and OntoSpecies are part of the Chemical and Process knowledge representation.

*The World Avatar* (www.theworldavatar.com) is a universal digital twin, implemented using Semantic Web technology (see Figure 5).^67^ The choice of the technology is based on the FAIR Guiding Principles for scientific data, that is, findable, accessible, interoperable, and reusable.^68^ In the context of chemistry, TWA hosts a federation of chemical and process development ontologies combining experimental, modeling, and theoretical aspects.^43−46^

The chemical ontologies including the herein developed OntoMOPs can share concepts with other ontologies, while software agents can enable interoperability, allowing for complex queries and model phenomena.

*The World Avatar* platform is a cross-domain and multiscale operational digital twin.^69^ Considering the urgency and interest in industrialization of metal organic material hybrids,^70^*The World Avatar* has the potential to connect material development^31^ with scaled-up process implementation in chemical plants, with further optimization of the energy consumption, material logistics, and waste minimization in the overall process.

#### Ontological Modeling

To apply the knowledge engineering approach,^33^ we developed the OntoMOPs ontology iteratively, following standard ontology development practices.^71−77^ The primary goal of the OntoMOPs ontology is to provide semantics to the relationship between MOPs, CBUs, and assembly models, ultimately laying the foundation for the development of a knowledge graph that is comprehensible to agents that can be integrated in TWA. The second goal of the OntoMOPs ontology is to provide a semantics-enabled complex query answering system that can inform professionals working on the modeling and preparation of MOPs. The former targets offer a way to define the scope of the ontology. The scope, in this case, is to answer problems regarding the construction of MOPs by providing information that can be used for informed decisions.

Our work depends on developing a terminological component that essentially defines classes and properties and a domain vocabulary (i.e., TBox). The assertion component (i.e., ABox) brings facts associated with the concepts of the TBox (i.e., information about MOPs, CBUs and AMs). The combination of TBox and ABoxes can then be used to answer the following competency questions:

- List all MOPs having a particular CBU.
- List all MOPs having a particular AM.
- What type of AMs have been constructed using a particular CBU?
- Show all MOPs having tetrahedral shape.
- Show all GBUs required to form a particular shape/AM.
- Show the substituting functionality of a particular CBU.
- What is the associated modularity of a particular species acting as a CBU in MOPs?

**Figure 6:**
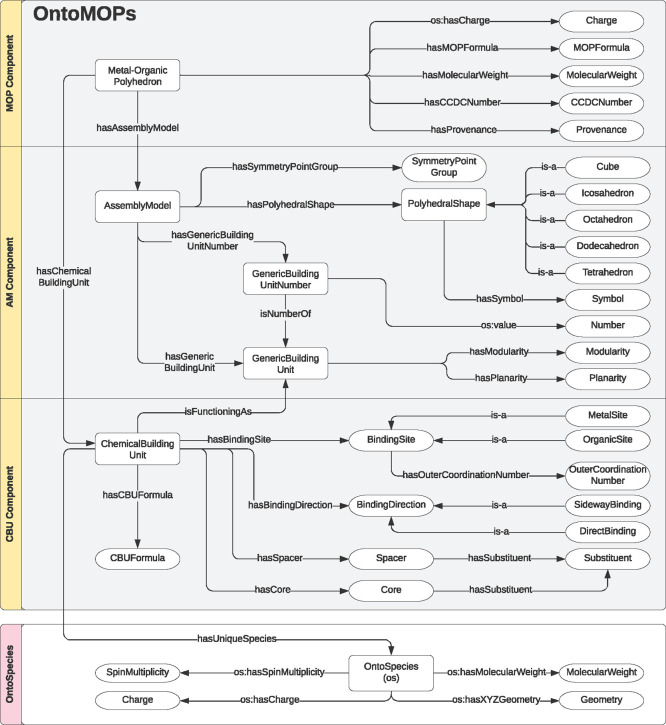
Core concepts and properties of the OntoMOPs ontology.

To answer these questions, we structure our ontology into three main components (see Figure 6). These components and concepts are created and interconnected using is-a, has-a, and is-functioning-as relations. In the MOP component, the main concept is a Metal–Organic Polyhedron which “is-a” Coordination Cage pointing out of our ontology. The Metal–Organic Polyhedron “has-a” Chemical Building Unit and “has” Assembly Model, representing the two central concepts in the second and third components, respectively. The Chemical building unit is interconnected to the Assembly Model component through “isFunctioningAs” relation pointing to the Generic building Unit concept.

In the MOP component, we see connections of the MOPs class with other concepts such as MOPcharge, MOPformula, and molecular mass. The concept of MOP also connects to the concept of Provenance, which contains data properties such as the DOI number of the article where a particular MOP is being reported. As many MOPs are related to motifs in crystalline materials, we also connected the concept of MOP to a CCDC number that can help locate the structure of the MOP in the Cambridge Crystallographic Data Centre.^78^ The MOP component also provides opportunities for future developments. One example is the presence of the “Cavity” and “CavityVolume”, which are intended to be populated in the near future with calculated void data, relevant for porosity applications.

In the Assembly Model component, the concept Assembly Model is connected to GBU and a GBU Number via has-a relations. The assembly model is also related to a symmetry point group and polyhedral shapes. Here, polyhedra such as Tetrahedron, Octahedron, Cube, Dodecahedron, Icosahedron, Rhombicuboctahedron, and Cuboctahedron are encoded. The polyhedral shape also has a data property—a shape symbol that uses the letter nomenclature for polyhedra reported in the reticular chemistry resource.^79^ The planarity and the modularity are encoded as data properties of the GBU.

The CBU component provides a connection between the OntoMOPs ontology and the OntoSpecies ontology. OntoSpecies is an ontology currently consisting of nearly 11 000 instances of chemical species for which there are a number of properties. This includes geometry, charge, spin multiplicity, and InChI. The OntoSpecies ontology has been primarily introduced to help with identifying chemical species uniquely.^43^ This identification occurs via Internationalized Resource Identifiers (IRIs) that help to connect chemical species with CBUs of MOPs, labeled using arbitrary strings. The CBU component in OntoMOPs does not aim to store these properties again; however, it models what chemical functionalities relate to the particular species in the context of the larger MOP assembly. These functionalities may be related to the (stereo)chemical nature of the binding site and thus used to model information suitable for distinguishing chemical complementarity between two CBUs. The CBU component also models information related to the central component, namely, the presence of substituents and spacer groups, which can provide help when querying MOP for a specific substituent or functionality. Using IRIs, the CBU component is connected to one or more GBUs, which models in how many different ways the CBU can connect and build a structure.

The OntoMOPs Ontology consists of 32 classes, 25 object properties, and 18 data properties (see the SI for more details). The concepts are consistently arranged when exploring using the HermiT reasoner.^80,81^

#### MOP Information and Geometry Data Curation

When collecting information and geometry data on MOPs and their CBUs, we kept in mind that although synthetic chemists may benefit from the projections of our work, our work in the first line is intended to aid directly future high-throughput computations of MOPs. According to the reviewed literature, the latter domain of MOP research is currently lacking in pace compared to experimental developments.^12,13^ Computations, especially DFT-based ones, can provide further information on optimized geometry, molecular viability, and electronic insights and speed-up innovation.^53,54^ However, one has to acknowledge that MOPs, like with many POMs, represent relatively heavy molecules that are often computationally expensive for DFT approaches.^82,83^ Further on, differences in training and qualitative thinking^84^ may also be present in the communication between synthetic MOP experts and computational chemists. Collaborative workflows where formulation proposals by synthetic experts are modeled and calculated by computational chemists remain low-throughput. At the same time, direct computational modeling without consideration of synthetically accessible building units can also lead to proposals that have little chance for experimental realization. In this regard, our data collection and output are intended to close this existing gap in knowledge and communication.

When considering molecular modeling of heavy inorganic and hybrid molecules such as MOPs or POMs, typically, the structure of interest is modeled with only a simple approximation of the surrounding environment with a conductor like screening model.^82,83^ Analogous to MOF research, to start computations on existing MOPs, one would need computation-ready geometries.^85^ To systematically model new MOPs, one needs geometries of building units and assembly models as templates for the rational design of MOP targets.

**Figure 7:**
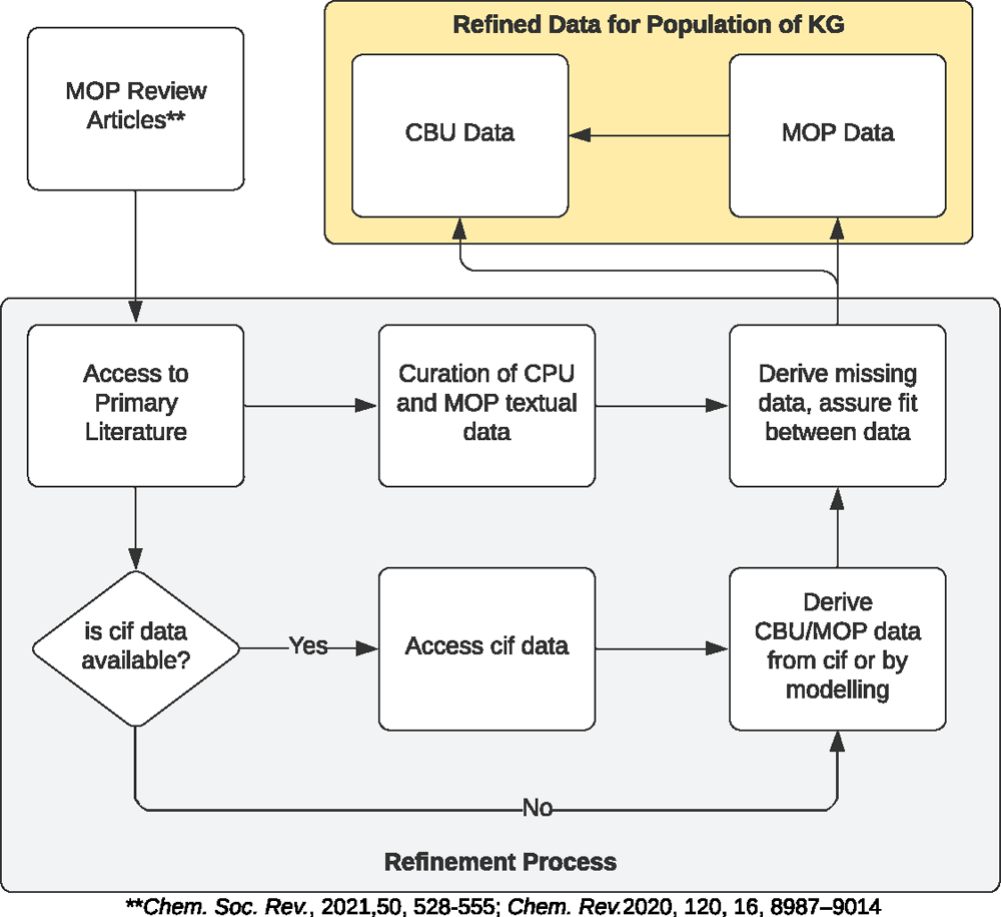
Schematic representation of the different steps applied to derive and structure the MOP and CBU data.

Our data collection starts by consultation of two recently reported MOP milestone reviews (see Figure 7).^12,13^ These reviews also have a strong tutorial-like character, targeting predominantly synthetic and applied chemist readers. The review articles are thoroughly illustrated and provide sufficient visual aids in allocating information through the literature. However, at the same time, most of the presented information is not practical for the direct extraction of data, but serves as a guiding overview of the primary literature. Following this, we consulted the primary literature from which we obtained information on the CBUs, MOPs, and MOPs’ crystallographic information files. The crystallographic information files were further used to extract *xyz* structures for the MOPs and parse them to obtain the *xyz* coordinates of the constituent CBUs. This was done in a way where solvent units and other cocrystallized molecules or labile units binding to the metal sites were manually removed.

For MOPs where the crystallographic structure was not reported or the structure showed some anomalies for direct *xyz* export (e.g., disorder, atoms missing, etc.), we used the graphical user interface of the Amsterdam Modeling Suite (www.scm.com) software for structure modeling.^86^ For most of the MOPs for which crystallographic structure was not reported, their structure could be derived from other previously known MOPs or through modeling of some peripheral organic substituents resulting from postfunctionalization. For addition of those organic functionalities and for the optimization of the organic CBUs, the universal force field was used.^86,87^ In this way, the geometries of 151 MOPs and 137 CBUs suitable for further DFT calculations (i.e., computation-ready) were obtained. The preparation of the working geometries was also a useful strategy that allowed us to cross-check the simplified MOP and CBU formulas and also to ensure that additional data based on the CBU geometries (i.e., molecular mass and InChI) are cleanly and correctly calculated.

The two review articles^12,13^ provide insights into the MOP construction based on the shape construct.^11,79^ However, the overall charge of individual MOPs is not mentioned. With consideration that MOP and CBU structures may undergo DFT calculations in the future, we manually derived the overall charge for some of the structures. Considering that many building blocks are metal-based, the charge also may affect their spin multiplicity. Although molecular magnetism is not part of our current KE studies, for data completeness, we systematically assigned the maximum possible spin multiplicity to all nondiamagnetic CBUs (i.e., approximating all spin-up). The topic of magnetism is not systematically discussed in the literature,^12,13^ although we acknowledge that many different magnetic scenarios may be possible.

#### Population of the KG

The data on MOPs and their chemical building units collected from the literature is stored in two CSV files (see the SI). These are then instantiated in OntoMOPs using an input agent consisting of a collection of written python scripts, which take the data from the CSV files and process them to produce JSON and then OWL files, which are then stored in the knowledge graph. This process results in each unique MOP being its own instance in OntoMOPs, with each chemical building unit also being a unique instance in OntoSpecies.

The developed software is freely accessible online: https://github.com/cambridge-cares/TheWorldAvatar/.

### Algorithms and Implementation

If one attempts to assemble a MOP directly by allocating chemically complementary CBUs to the corresponding GBUs of its particular assembly model, there is a high risk that irrational MOP structures will be proposed. The reason is that in this approach it is difficult to account for differences in dihedrals. An alternative strategy is to first locate all possible MOPs for a given AM. The next step is to derive the associated CBUs of those MOPs. Finally, the CBUs can be separated into “sets” based on their GBU characteristics. Using the AM as a template, MOPs can be combinatorially constructed by finding chemically complementary CBUs from these two sets. Some of the constructed MOPs will correspond to instances already present in TWA, while others will be completely new (Figure S9a in the SI). However, this approach is highly restrictive, and thus, if a small number of MOPs are represented by a certain AM (i.e., low versatility), the number of new structures that can be derived will be also highly limited. To derive a higher versatility of new rationally constructed MOP structures, one has to expand the CBU basis beyond just a single AM. To be able to achieve the latter without compromising the accuracy of the rational construction, the original set of CBUs is updated with CBUs from other sets for other assembly models with which it has a CBU instance in common (Figure S9b in the SI).

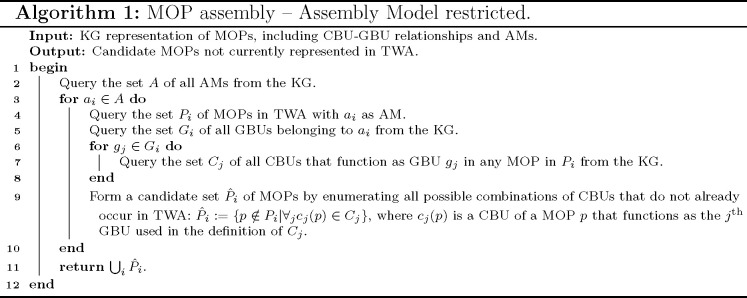



In this line, we developed two algorithmic approaches. Algorithm 1 represents the direct application of the AMs method and thus restricts the construction of MOPs without CBU share between sets corresponding to different AMs. When applying Algorithm 1, the sets populated with many MOPs are expected to have many different CBUs and thus project a higher potential for new instantiation. In Algorithm 2, exchanges between sets are allowed, providing an opportunity for an increase in the number of MOPs with assembly models that were originally sparsely populated.

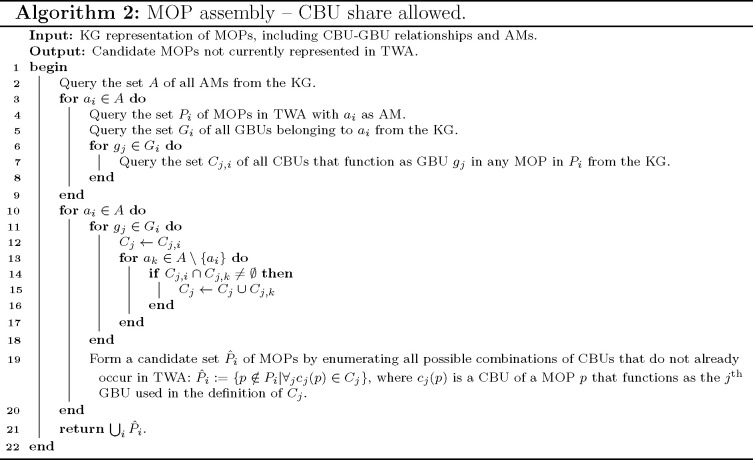



## Results and Discussion

### Prediction of New MOPs Structures: Algorithmic Output

**Figure 8:**
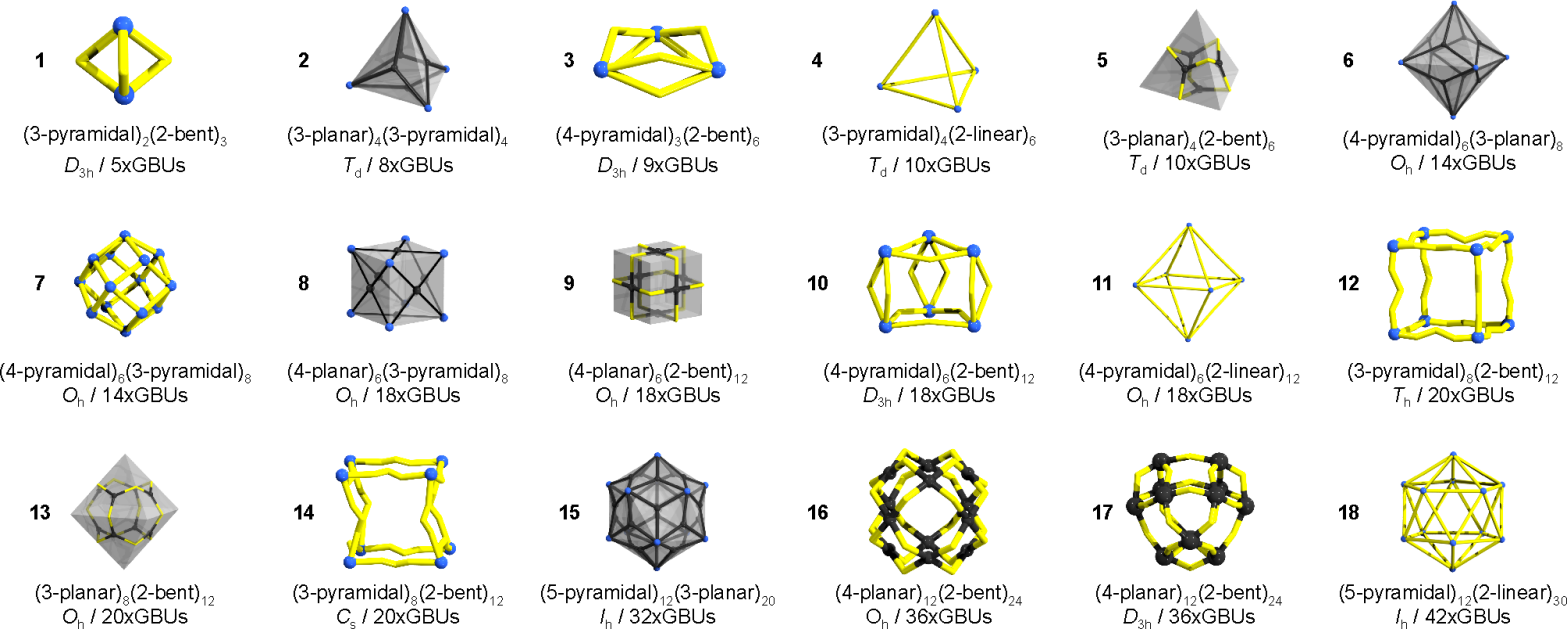
Assembly models present in the OntoMOPs cage, representing the construction principles of 151 reported MOP instances.

In the OntoMOPs KG there are 18 different AMs (see Figure 8 and Table S1 in the SI). All AMs are based on two different types of GBUs. The smallest AM is built using 5 GBUs and it is the diadic (3-pyramidal)_2_(2-bent)_3_ with D_3*h*_ symmetry point group. The largest AM is built using 42 GBUs and it is the (5-pyramidal)_12_(2-linear)_30_ with I_*h*_ symmetry point group. The remaining AMs span the range between these two extremes. All 18 AMs consist of pairs of seven different GBUs, namely 2-linear/bent 3-/4-/5-pyramidal and 3-/4-planar. The 5-planar CBUs are rare in chemistry (probably due to unusual coordination and strain), and thus the 5-planar GBU is not found among the GBUs currently in TWA. This implies that certain AMs such as the formally derived (5-planar)_12_(2-bent)_30_ have not been “discovered” among MOPs yet (Figure 4). However, other AMs reminiscent of Archimedean, Catalan, and Johnson solids are present in the TWA. In addition, nonpolyhedral AMs such as a polygon, a prism, and a diad AM are also present in TWA.

The latter three AMs may appear as “outliers”. However, they are purposely present as their associated CBUs participate in the construction of other MOPs with different AMs. All AMs adopt one of the five symmetries T_*d*_, O_*h*_, I_*h*_, C_*s*_, D_3*h*_, and T_*h*_. There are also two pairs of isomeric AMs, namely the(anti)cuboctahedral (4-planar)_12_(2-bent)_20_ and the cuboidal (3-pyramidal)_8_(2-bent)_12_, where the isomerism originates from the configurational orientation of the 2-bent GBUs. The cuboidal (3-pyramidal)_8_(2-linear)_12_ is absent from TWA, as well as the icosahedral (3-pyramidal)_2_(2-linear)_30_. The reason is that, to the best of our knowledge, there is an absence of reported inorganic CBUs that can exhibit the wide angles suitable for the construction of those AMs.

**Figure 9:**
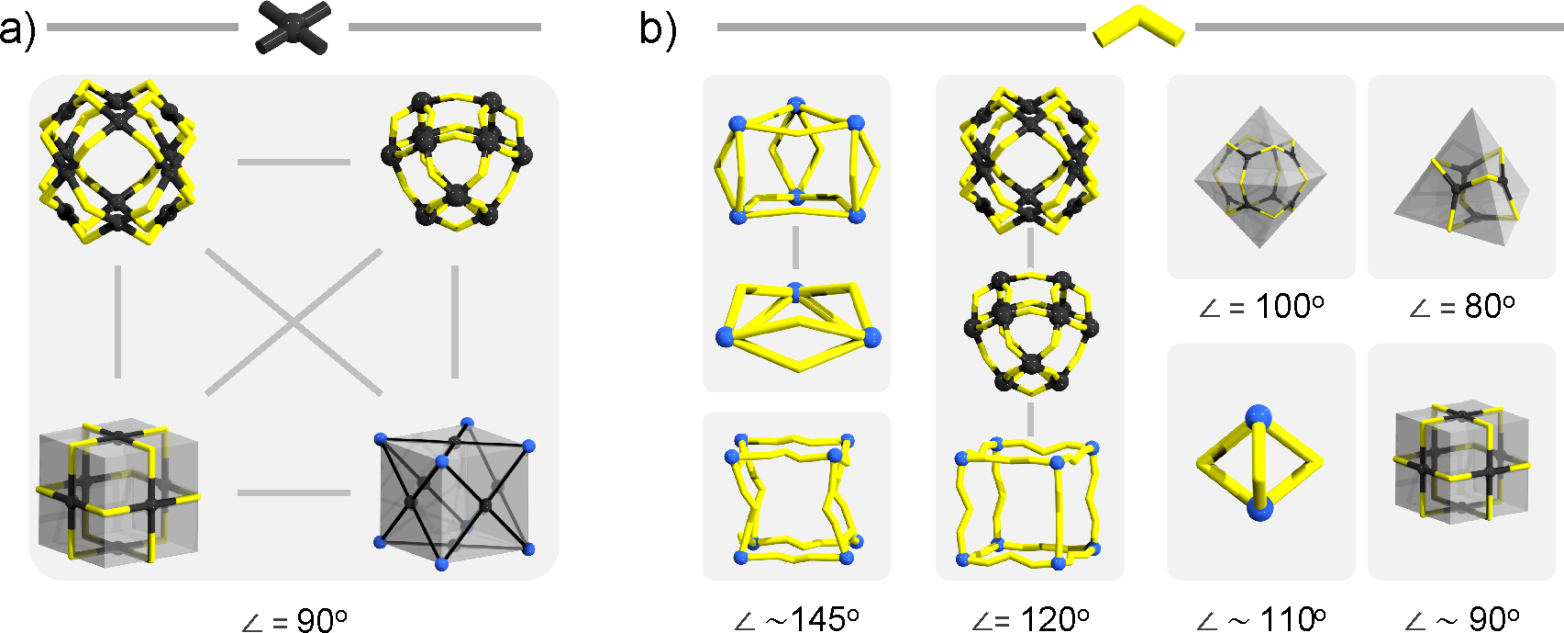
Highly interrelated sets of different AMs containing (a) 4-planar CBUs and (b) 2-bent CBUs.

In OntoMOPs there are seven general GBUs. If placed as nodes on a graph, the general GBUs are interconnected via 18 assembly models (see Figure 9a). From the GBU nodes, the most interconnected is the one referring to the 2-bent unit, which as discussed earlier (see the Derivation of Assembly Models section above) may be represented by CBUs with different dihedral angles. Therefore further differentiation between 2-bent GBUs is crucial. One of the discoveries of Algorithm 2 is that there are in total 37 related sets that have at least one CBU in common, and thus they can exchange CBUs.

One of the most interconnected sets is the one referring to 4-planar GBUs (see Figure 9b). This is the case because from a coordination chemistry viewpoint, most transition-metal based complexes can function as 4-planar CBUs, and thus there is no strong dihedral differentiation. However, in the case of the 2-bent GBU, our algorithm has found common ligands between particular sets, while other sets of 2-bent ligands have not been altered (Figure 9b). This implies that even without hard-coding, the algorithm can successfully deduce that certain differences in dihedrals are acceptable when exchanging CBUs, but not all.

**Figure 10:**
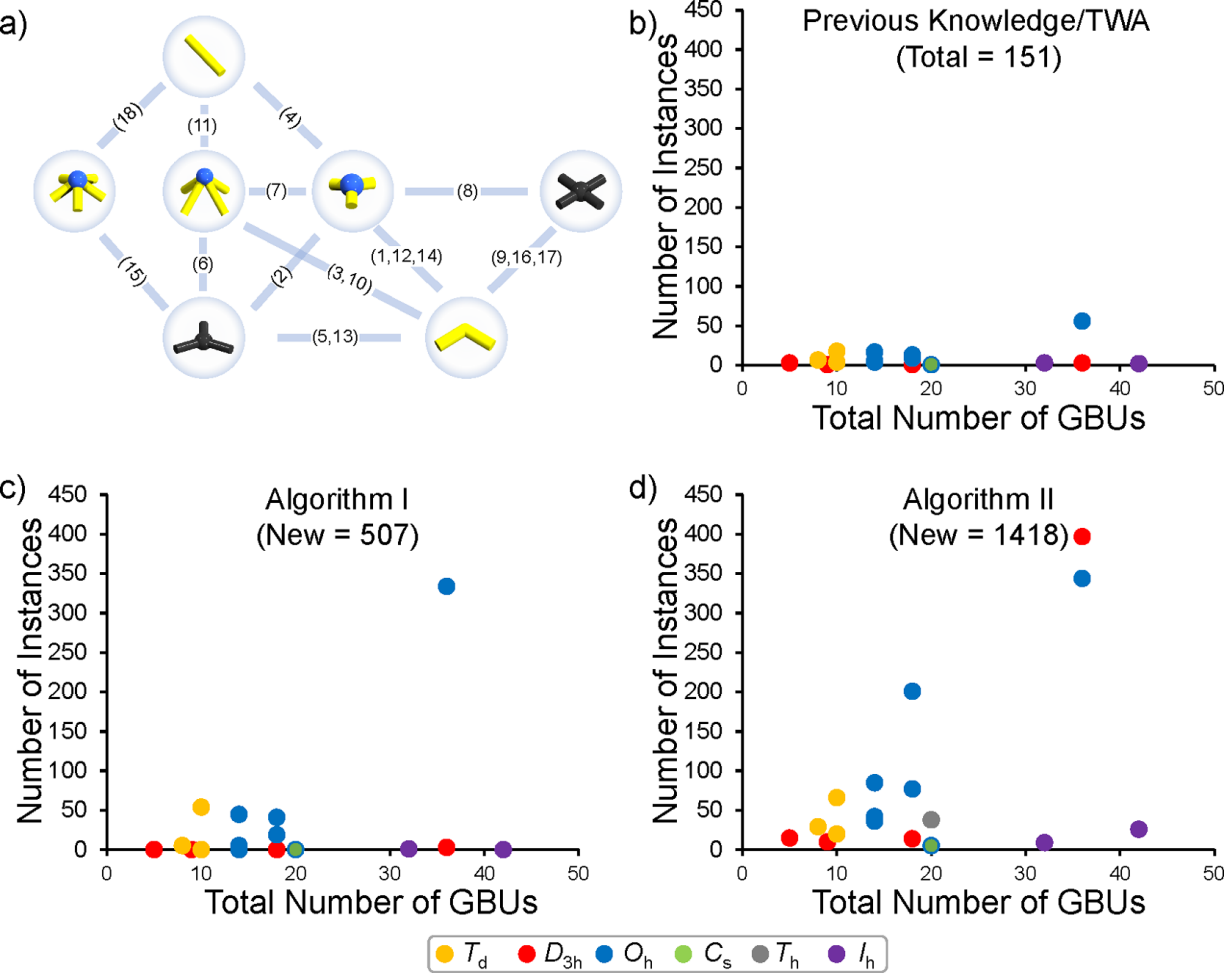
(a) Graph depicting the GBUs and the Assembly Models as nodes and links, respectively. Number of MOP instances as a function of the total GBU sum present in TWA (b) and obtained following Algorithm 1 (c) and Algorithm 2 (d).

In order to have a perspective on the obtained number of instances from the application of the algorithms, one may consider a rough estimation of the exploratory chemical space. The exploratory chemical space associated with high-throughput synthetic explorations and such space may emerge by multiplying the combinations to be studied across number of changed parameters. If 91-organic and 46-inorganic CBUs are reacted across 18 different scenarios, then the total exploratory space would be 75 348 unique chemical environments. In stark contrast to the exploratory space, Algorithms 1 and 2 project an immediate chemical space of 506 and 1418 constructible MOP instances, respectively (see Figure 10 and a complete list in Table S2 in the SI). This implies that the algorithms can effectively narrow down exploratory spaces and thus make automated synthetic explorations more focused. In comparison to the MOP instances currently present in TWA, where the (4-planar)_12_(2-bent)_24_ (O_*h*_) archetype counts for approximately 37% of all structures, Algorithm 1 projects that assembly model (4-planar)_12_(2-bent)_24_ (O_*h*_) accounts for approximately 66% of the newly derived structures. The reason for this is that there can be many combinations between metal nodes (e.g., [Pd_2_], [Cu_2_], [Rh_2_], etc.) and other 2-bent organic CBUs in this AM. By contrast, in Algorithm 2, it is deduced that MOPs represented by the anticuboctahedral derivative of (4-planar)_12_(2-bent)_24_ (O_*h*_) (i.e., (4-planar)_12_(2-bent)_24_ (D_3*h*_)) can also be constructed in large numbers. As the anticuboctahedral derivative appears to find suitable CBUs in the (3-pyramidal)_8_(2-bent)_12_ (T_*h*_) set, the number of new predicted anticuboctahedral MOPs amounts to 397, the largest number for any of the AMs. However, this could change if additional MOPs instances that have CBUs that connect previously unconnected AMs are introduced into the KG.

**Figure 11:**
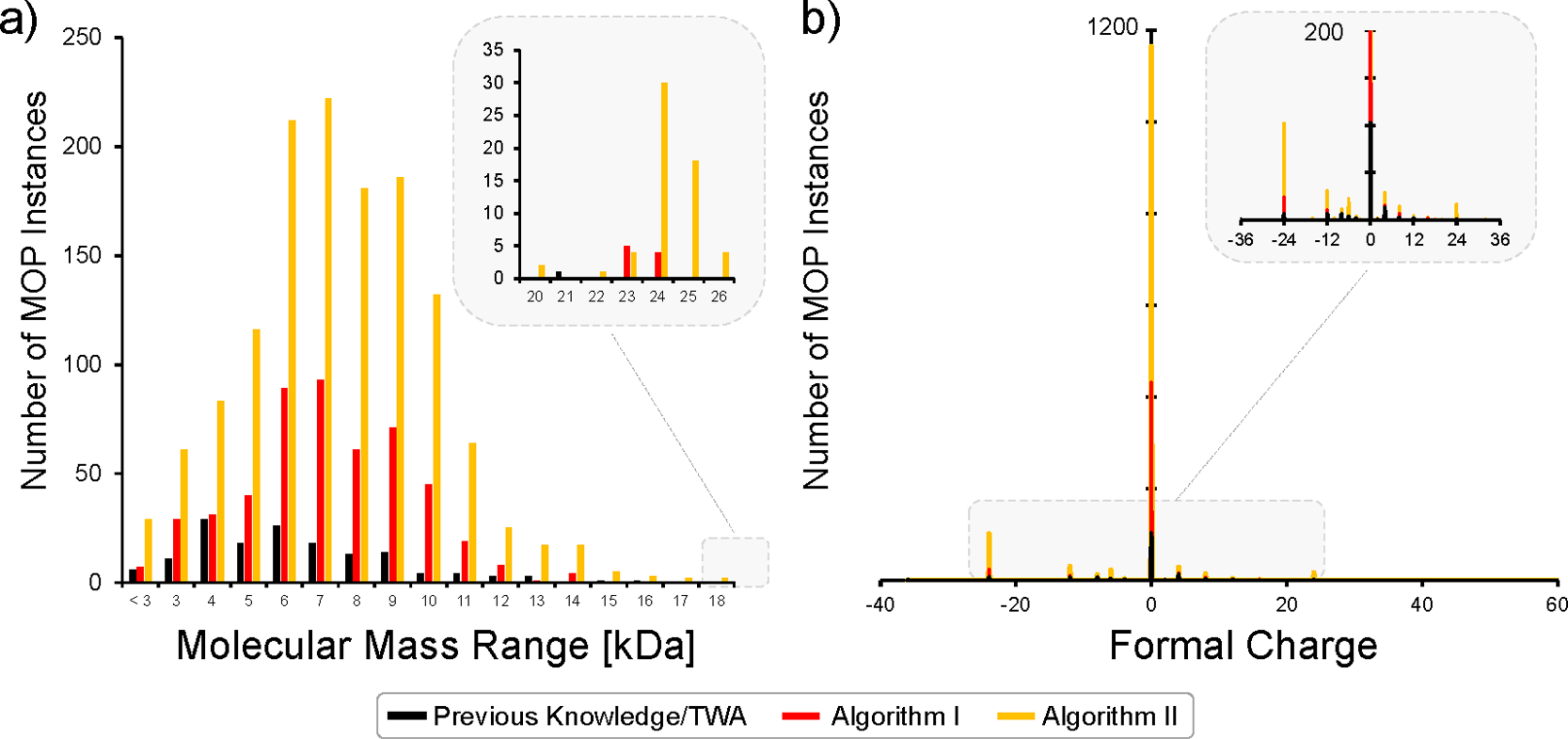
Distribution of reported MOPs instances and the newly algorithmically derived MOPs instances as a function of (a) their molecular mass ranges and (b) their overall charge.

Our algorithmic implementation allows us to query the molecular mass of the CBUs, and using the respective GBU numbers associated with the respective AM, one can derive the mass of the new MOPs. The molecular mass between most of the MOP instances differs except for the cases when isomers can be constructed. A histogram projection allows convenient analysis of the mass distributions in separate ranges of 1 kDa. Most of the starting MOP structures found in the literature show distribution maxima at 4 and 6 kDa with an overall median at 6584.55 g·mol^–1^. In comparison, the new MOPs derived using Algorithm 1 and Algorithm 2 show maxima at 7 and 8 kDa, and median molecular mass values of 7586.83 g·mol^–1^ and 7875.685 g·mol^–1^, respectively. The shift in median is due to the fact that the newly derived MOP sets are predominantly represented by MOPs that associate with (anti)cuboctahedral AMs employing 36 GBUs. In addition, when turning from reported to algorithmically derived MOPs, one also observes a rise in the number of very heavy MOP structures, which are those that span the region of 23–26 kDa (Figure 11a). The reasons for this rise are that there are new (anti)cuboctahedral MOP constructions that employ heavy organic CBUs (e.g., those with long alkyl chains) as well the general rise of MOPs employing heavy POM-based inorganic nodes. This is not an unexpected outcome considering that CBUs suitable for constructing (anti)cuboctahedral MOPs are very common in the OntoSpecies KG, while POM-based CBUs are one of the heaviest CBUs used to build MOPs.

The overall MOP charge is highly relevant when devising new porous ionic solid combinations that rely on both positively and negatively charged MOPs. However, one in general needs to be careful with this interpretation as charged MOPs may be able to coexist in a set of different charge states. The different charge states may be associated with different oxidation numbers of protonation states of the CBUs. Our algorithm is currently exploring the constructability problem, where the protonation and the oxidation state may be less relevant unless they block the binding site of the CBUs.

The distribution of the overall MOP charges show that most instances, from literature and those algorithmically derived, are in the range of −36 up to +24 (Figure 11b). To have a complete and saturated assembly model, the number of binding units from the organic and inorganic units should match. As the number of binding units typically “mirror” the magnitude of the absolute charge, the net charge outcome of the MOP ends up being neutral. Indeed some 64% of all MOP instances in the OntoMOPs KG are neutral. However, when there is a deviation from this scenario, the overall MOP structure may appear as charged. For instance, positively charged MOPs result from the use of neutral organic linkers (e.g., [C_6_H_4_(C_3_H_2_N_2_)_2_]) and positively charged inorganic CBUs. On the other hand, negatively charged MOPs typically derive from the combination of highly negative POM based CBUs (e.g., [PW_9_O_37_Ni_6_NH_2_C_4_H_3_]) and negatively charged carboxylate ligands, or use of 4-pyramidal organic ligands (e.g., [(C_6_HO_3_)_4_(C_4_H_8_)_4_]_6_)] and low charged metal cations (e.g., [M_3_]^6+^). Although not fully arbitrary, negative charges may derive from the use of benzene-1,3,5-tricarboxylate ligand (i.e., BTC = [(C_6_H_3_)(CO_2_)_3_]^2–^) as 2-bent units. The BTC is well-known as a 3-planar organic CBU. When employed as 2-bent CBU, one site remains unsaturated, making the structures interesting in postsynthetic functionalization.^88^ When modeling, one may consider a scenario where the free carboxylate binding site is protonated, deprotonated, or combination of both. As we were interested in obtaining the maximum outcome on constructable MOPs, BTC was considered to be a deprotonated CBU.

**Figure 12:**
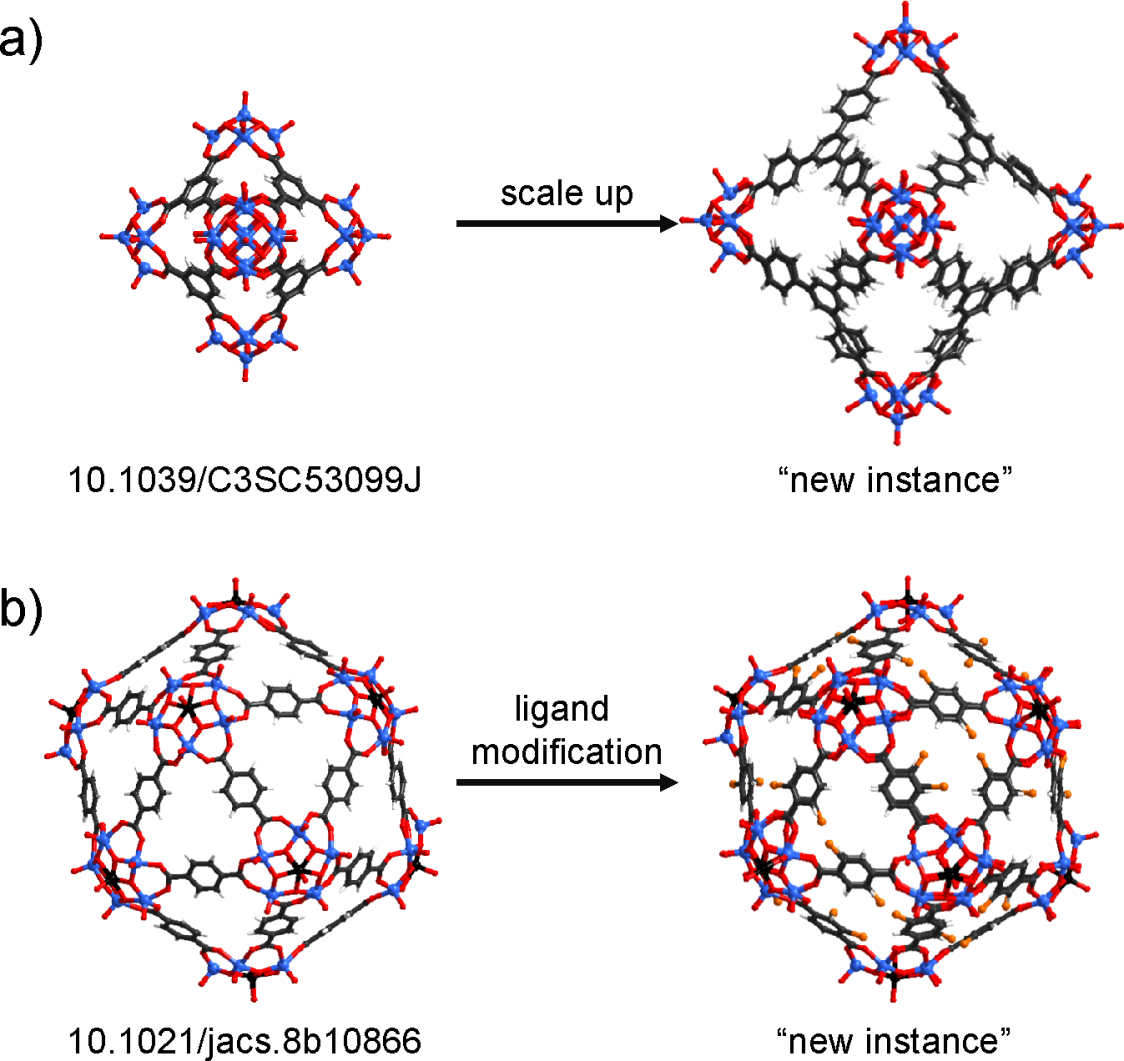
Models of MOPs based on output from Algorithm 2: (a) size increase based on utilization of a spacer moieties and (b) Br-substituted derivatives.

As mentioned earlier, the data curation has been based on information presented in the two most recent and most influential review articles, both covering reported MOPs until mid-2020.^12,13^ By not adding newly reported MOP instances after that period, one can observe if the algorithm predicts instances that experts would also envision and attempt to prepare. In this line, one general trend is to substitute a smaller with a larger organic unit. Considering that the octahedral MOP [V_5_O_9_]_6_[(C_6_H_3_)(CO_2_)_3_]_8_^6–^ is present in TWA,^89^ the algorithm has derived a new larger structure with formula [V_5_O_9_]_6_[L]_8_^6–^ where L = [(C_3_N_3_)(C_6_H_4_)_3_(CO_2_)_3_], [(C_6_H_3_)(C_6_H_4_)_3_(CO_2_)_3_], [(C_6_H_3_)(C_2_C_6_H_4_)_3_(CO_2_)_3_], and [(C_6_H_3_)((C_6_H_4_)_2_)_3_(CO_2_)_3_]. Among the different ligands, the use of 1,3,5-tris(4-carboxyphenyl)-benzene to form [V_5_O_9_]_6_[(C_6_H_3_)(C_6_H_4_)_3_(CO_2_)_3_]_8_^6–^ has been reported by Su’s group in August 2020.^90^ The obtained structure was not covered in the review articles; however, its prediction suggests that our algorithm can replicate the rational designs of experts to a significant level (see Figure 12a). Considering the icosahedral [WV_5_O_11_]_12_[C_6_H_4_(CO_2_)_2_]_30_^12–^,^47^ the algorithm proposed a derivative structure in which one hydrogen atom of the organic CBU is formally substituted by a halogen atom. One proposed formulation is [WV_5_O_11_]_12_[C_6_H_3_Br(CO_2_)_2_]_30_^12–^. This structure would be the subject of rich configurational isomerism. This would imply that in addition to the present model (see Figure 12b), many other configurations may be possible to be constructed. In that regard, MOPs similarly as POMs are likely to be a subject of rich configurational isomerism, which is not the focus of the present work.^83,91^ However, very recently an algorithm capable of treating configurational problems for polyhedral species has been developed,^92^ which in principle can be a modular extension to the present work (see Comment 2 in the SI).

In addition to the MOP examples presented in Figure 12, the Supporting Information file contains a list of 18 graphical illustrations of new MOP constructions representative for each assembly model.

## Summary and Outlook

The classical concept of secondary building units has been an important concept over the past two decades, leading to the rational design and discovery of many of many MOPs, MOFs, and COFs. In this work, we differentiated between the chemical and structural nature of the SBU, and derived a conceptual description of MOPs based on assembly models. The key concepts were then used to extend TWA with the OntoMOPs ontology connecting to existing concepts from OntoSpecies. The TWA was populated with MOP data, which we curated from the literature and structured in a systematic way to facilitate its further use in the exploration of the immediate chemical space.

Algorithms were constructed for the discovery of new MOPs that make use of information in OntoMOPs. On the basis of the available 137 CBUs and 151 experimentally verified MOPs, this MOP Discovery agent rationally proposed up to 1418 new MOPs that were previously not recorded in the literature (i.e., in TWA). The overall study also shows that semantically driven and instance-based approaches can function simply based on meta-rules. In such a system, “outliers” do not break the meta-rules, but only update the set of assembly “blueprints”; thus, the next iteration is more refined and potential uncertainties are predicted. Our computer-aided rational design approach can be combined with other developments such as Waller’s algorithm that discovers chemical reactivity.^93^ This can identify species that can potentially function as new CBUs and thus enable for more rapid exploration of the deep (i.e., uncharted) chemical space of MOPs in conjunction with existing data in our knowledge graph. Similarly, adaptations of the existing algorithms for automated molecular modeling algorithms^94−98^ can be used as part of a larger workflow enabling further calculations and dynamic updates of the MOP knowledge in TWA.

The semantically based, ontology-driven discover algorithms successfully undertook rational structural proposals for MOPs, and we are currently extending this approach to related polyhedral and reticular materials. Using natural language processing for chemistry, our group has currently developed the “Marie” platform^99^ that is able to interact with chemists and provide feedback. It is planned to extend Marie to make complex queries for MOPs and other reticular and polyhedral materials possible. This will make it more natural for MOP chemists to interact with *The World Avatar*, with the aim to improve the quality and quantity of data in TWA, which will in turn allow for increased potential of new discoveries in the MOPs field.

## Supporting Information Available

Summary of algorithmic output, description Logic, list of chemical building units, illustration of the CBU sharing phenomenon, comments, algorithmic Output II and visual construction of new MOPs based on Algorithmic Output II (PDF)
Supporting Video S1 (MP4)

## Notes

Comprehensive research data supporting this publication is available in the University of Cambridge data repository (DOI: 10.17863/CAM.82775).
