# Supplementary material for: Automated Rational Design of Metal–Organic Polyhedra
Source: J Am Chem Soc. 2022 Jun 22;144(26):11713–28. doi: 10.1021/jacs.2c03402 (PMC9264355; doi:10.1021/jacs.2c03402)

Supporting Information

Automated Rational Design of Metal-Organic Polyhedra

Aleksandar Kondinski[1], Angiras Menon[1], Daniel Nurkowski[2], Feroz Farazi[1], Sebastian Mosbach[1], Jethro Akroyd[1], Markus Kraft[1,2,3,4,5]*

1. Department of Chemical Engineering and Biotechnology, University of Cambridge, Philippa Fawcett Drive, Cambridge, CB3 0AS, UK.
2. CMCL Innovations Sheraton House Cambridge CB3 0AX United Kingdom
3. CARES Cambridge Centre for Advanced Research and Education in Singapore, 1 Create Way, CREATE Tower, #05-05, Singapore, 138602.
4. School of Chemical and Biomedical Engineering, Nanyang Technological University, 62 Nanyang Drive Singapore, 637459.
5. The Alan Turing Institute, London, United Kingdom.

* Correspondence: mk306@cam.ac.uk

Table of Contents:

Section:	Page
1. Summary of Algorithmic Output	2
2. OntoMOPs – Description Logic	3
3. List of Chemical Building Units (Figures S1-S8)	6
4. CBU Sharing Phenomenon (Figure S9)	13
5. Comments (Figure S10)	14
6. Algorithmic Output II (Table S2)	16
7. Algorithmic Output II - Visual Construction of new MOPs	48

1. Summary of Algorithmic Output

Table S1: Number of MOP Formula models derived using algorithm I and algorithm II in comparison with the MOP formulas in the KG

Nr.	Assembly Model	In KG	Algorithm I	Algorithm II
1	(3-pyramidal)$_2$(2-bent)$_3$ (D_{3h})	3	0	15
2	(3-planar)$_4$(3-pyramidal)$_4$ (T_d)	7	5	29
3	(4-pyramidal)$_3$(2-bent)$_6$ (D_{3h})	1	0	10
4	(3-pyramidal)$_4$(2-linear)$_6$ (T_d)	18	54	66
5	(3-planar)$_4$(2-bent)$_6$ (T_d)	4	0	20
6	(4-pyramidal)$_6$(3-planar)$_8$ (O_h)	17	45	85
7	(4-pyramidal)$_6$(3-pyramidal)$_8$ (O_h)	6	5	42
8	(4-planar)$_6$(3-pyramidal)$_8$ (O_h)	4	0	36
9	(4-planar)$_6$(2-bent)$_{12}$ (O_h)	13	41	77
10	(4-pyramidal)$_6$(2-bent)$_{12}$ (D_{3h})	1	0	14
11	(4-pyramidal)$_6$(2-linear)$_{12}$ (O_h)	9	19	201
12	(3-pyramidal)$_8$(2-bent)$_{12}$ (T_h)	1	0	38
13	(3-planar)$_8$(2-bent)$_{12}$ (O_h)	1	0	5
14	(3-pyramidal)$_8$(2-bent)$_{12}$ (C_s)	1	0	5
15	(5-pyramidal)$_1$2(3-planar)$_{20}$ (I_h)	3	1	9
16	(4-planar)$_1$2(2-bent)$_{24}$ (O_h)	57	333	343
17	(4-planar)$_1$2(2-bent)$_{24}$ (D_{3h})	3	3	397
18	(5-pyramidal)$_1$2(2-linear)$_{30}$ (I_h)	2	0	26

2. OntoMOPs – Description Logic

Classes

$$
\begin{aligned}
\text{MolecularCage} &\sqsubseteq \top \\
\text{CoordinationCage} &\sqsubseteq \text{MolecularCage} \\
\text{MetalOrganicPolyhedron} &\sqsubseteq \text{CoordinationCage} \\
\text{AssemblyModel} &\sqsubseteq \top \\
\text{GenericBuildingUnit} &\sqsubseteq \top \\
\text{GenericBuildingUnitNumber} &\sqsubseteq \top \\
\text{ChemicalBuildingUnit} &\sqsubseteq \top \\
\text{Cavity} &\sqsubseteq \top \\
\text{BindingDirection} &\sqsubseteq \top \\
\text{SidewayBinding} &\sqsubseteq \text{BindingDirection} \\
\text{DirectBinding} &\sqsubseteq \text{BindingDirection} \\
\text{BindingSite} &\sqsubseteq \top \\
\text{OrganicSite} &\sqsubseteq \text{BindingSite} \\
\text{MetalSite} &\sqsubseteq \text{BindingSite} \\
\text{Spacer} &\sqsubseteq \top \\
\text{Provenance} &\sqsubseteq \top \\
\text{PolyhedralShape} &\sqsubseteq \top \\
\text{Tetrahedron} &\sqsubseteq \text{PolyhedralShape} \\
\text{Cube} &\sqsubseteq \text{PolyhedralShape} \\
\text{Octahedron} &\sqsubseteq \text{PolyhedralShape} \\
\text{Icosahedron} &\sqsubseteq \text{PolyhedralShape} \\
\text{Dodecahedron} &\sqsubseteq \text{PolyhedralShape} \\
\text{Cuboctahedron} &\sqsubseteq \text{PolyhedralShape} \\
\text{Rhombicuboctahedron} &\sqsubseteq \text{PolyhedralShape} \\
\text{RhombicDodecahedron} &\sqsubseteq \text{PolyhedralShape} \\
\text{Species} &\sqsubseteq \top \\
\text{Charge} &\sqsubseteq \top \\
\text{MolecularWeight} &\sqsubseteq \top
\end{aligned}
$$

Object Properties

$$
\begin{aligned}
\text{MetalOrganicPolyhedron} &\sqsubseteq \leq 1\ \text{hasChemicalBuildingUnit.ChemicalBuildingUnit} \sqcap \\
&\geq 1\ \text{hasChemicalBuildingUnit.ChemicalBuildingUnit} \\
\text{MetalOrganicPolyhedron} &\sqsubseteq \leq 1\ \text{hasAssemblyModel.AssemblyModel} \sqcap \\
&\geq 1\ \text{hasAssemblyModel.AssemblyModel} \\
\text{MetalOrganicPolyhedron} &\sqsubseteq \leq 1\ \text{hasCavity.Cavity} \sqcap \\
&\geq 1\ \text{hasCavity.Cavity} \\
\text{MetalOrganicPolyhedron} &\sqsubseteq \leq 1\ \text{hasProvenance.Provenance} \sqcap \\
&\geq 1\ \text{hasProvenance.Provenance} \\
\text{Cavity} &\sqsubseteq \leq 1\ \text{hasMOPCavityVolume.Volume} \sqcap \\
&\geq 1\ \text{hasMOPCavityVolume.Volume} \\
\text{AssemblyModel} &\sqsubseteq \leq 1\ \text{hasGenericBuildingUnit.GenericBuildingUnit} \sqcap \\
&\geq 1\ \text{hasGenericBuildingUnit.GenericBuildingUnit} \\
\text{AssemblyModel} &\sqsubseteq \leq 1\ \text{hasGenericBuildingUnitNumber.GenericBuildingUnitNumber} \sqcap \\
&\geq 1\ \text{hasGenericBuildingUnitNumber.GenericBuildingUnitNumber} \\
\text{AssemblyModel} &\sqsubseteq \leq 1\ \text{hasPolyhedralShape.PolyhedralShape} \sqcap \\
&\geq 1\ \text{hasPolyhedralShape.PolyhedralShape} \\
\text{ChemicalBuildingUnit} &\sqsubseteq \leq 1\ \text{hasBindingDirection.BindingDirection} \sqcap \\
&\geq 1\ \text{hasBindingDirection.BindingDirection} \\
\text{ChemicalBuildingUnit} &\sqsubseteq \leq 1\ \text{hasCore.Core} \sqcap \\
&\geq 1\ \text{hasCore.Core} \\
\text{ChemicalBuildingUnit} &\sqsubseteq \leq 1\ \text{hasSpacer.Spacer} \sqcap \\
&\geq 1\ \text{hasSpacer.Spacer} \\
\text{ChemicalBuildingUnit} &\sqsubseteq \leq 1\ \text{hasBindingSite.Binding Site} \sqcap \\
&\geq 1\ \text{hasBindingSite.Binding Site} \\
\text{GenericBuildingUnitNumbert} &\sqsubseteq \leq 1\ \text{isNumberOf.GenericBuildingUnit} \sqcap \\
&\geq 1\ \text{isNumberOf.GenericBuildingUnit} \\
\text{PolyhedralShape} &\sqsubseteq \leq 1\ \text{hasSymbol.Symbol} \sqcap \\
&\geq 1\ \text{hasSymbol.Symbol} \\
\text{ChemicalBuildingUnit} &\sqsubseteq \leq 1\ \text{OS:hasUniqueSpecies.OS:Species} \sqcap \\
&\geq 1\ \text{OS:hasUniqueSpecies.OS:Species} \\
\text{MetalOrganicPolyhedron} &\sqsubseteq \leq 1\ \text{OS:hasMolecularWeight.OS:MolecularWeight} \sqcap \\
&\geq 1\ \text{OS:hasMolecularWeight.OS:MolecularWeight} \\
\text{MetalOrganicPolyhedron} &\sqsubseteq \leq 1\ \text{OS:hasCharge.OS:Charge} \sqcap \\
&\geq 1\ \text{OS:hasCharge.OS:Charge}
\end{aligned}
$$

Data Properties

$$\exists \text{ hasCBUFormula}.\top \sqsubseteq \text{ChemicalBuildingUnit}$$
$$\top \sqsubseteq \forall \text{ hasCBUFormula.String}$$
$$\exists \text{ hasMOPFormula}.\top \sqsubseteq \text{MetalOrganicPolyhedron}$$
$$\top \sqsubseteq \forall \text{ hasMOPFormula.String}$$
$$\exists \text{ hasCCDCNumber}.\top \sqsubseteq \text{MetalOrganicPolyhedron}$$
$$\top \sqsubseteq \forall \text{ hasCCDCNumber.Integer}$$
$$\exists \text{ hasCCDCNumber}.\top \sqsubseteq \text{MetalOrganicPolyhedron}$$
$$\top \sqsubseteq \forall \text{ hasCCDCNumber.Integer}$$
$$\exists \text{ hasModularity}.\top \sqsubseteq \text{GenericBuildingUnit}$$
$$\top \sqsubseteq \forall \text{ hasModularit.Integer}$$
$$\exists \text{ hasXYZGeometry}.\top \sqsubseteq \text{MetalOrganicPolyhedron}$$
$$\top \sqsubseteq \forall \text{ hasXYZGeometry.String}$$
$$\exists \text{ hasPlanarity}.\top \sqsubseteq \text{GenericBuildingUnit}$$
$$\top \sqsubseteq \forall \text{ hasPlanarity.String}$$
$$\exists \text{ hasUnitNumberValue}.\top \sqsubseteq \text{GenericBuildingUnitNumber}$$
$$\top \sqsubseteq \forall \text{ hasUnitNumberValue.Integer}$$
$$\exists \text{ hasSymmetryPointGroup}.\top \sqsubseteq \text{AssemblyModel}$$
$$\top \sqsubseteq \forall \text{ hasSymmetryPointGroup.String}$$
$$\exists \text{ hasReferenceDOI}.\top \sqsubseteq \text{Provenance}$$
$$\top \sqsubseteq \forall \text{ hasReferenceDOI.String}$$
$$\exists \text{ hasSymbol}.\top \sqsubseteq \text{PolyhedralShape}$$
$$\top \sqsubseteq \forall \text{ hasSymbol.String}$$
$$\exists \text{ hasSymbol}.\top \sqsubseteq \text{PolyhedralShape}$$
$$\top \sqsubseteq \forall \text{ hasSymbol.String}$$
$$\exists \text{ hasOuterCoordinationNumber}.\top \sqsubseteq \text{BindingSite}$$
$$\top \sqsubseteq \forall \text{ hasOuterCoordinationNumber.Integer}$$
$$\exists \text{ hasSymbol}.\top \sqsubseteq \text{PolyhedralShape}$$
$$\top \sqsubseteq \forall \text{ hasSymbol.String}$$
$$\exists \text{ OS:value}.\top \sqsubseteq \text{AssemblyModel}$$

3. List of Chemical Building Units

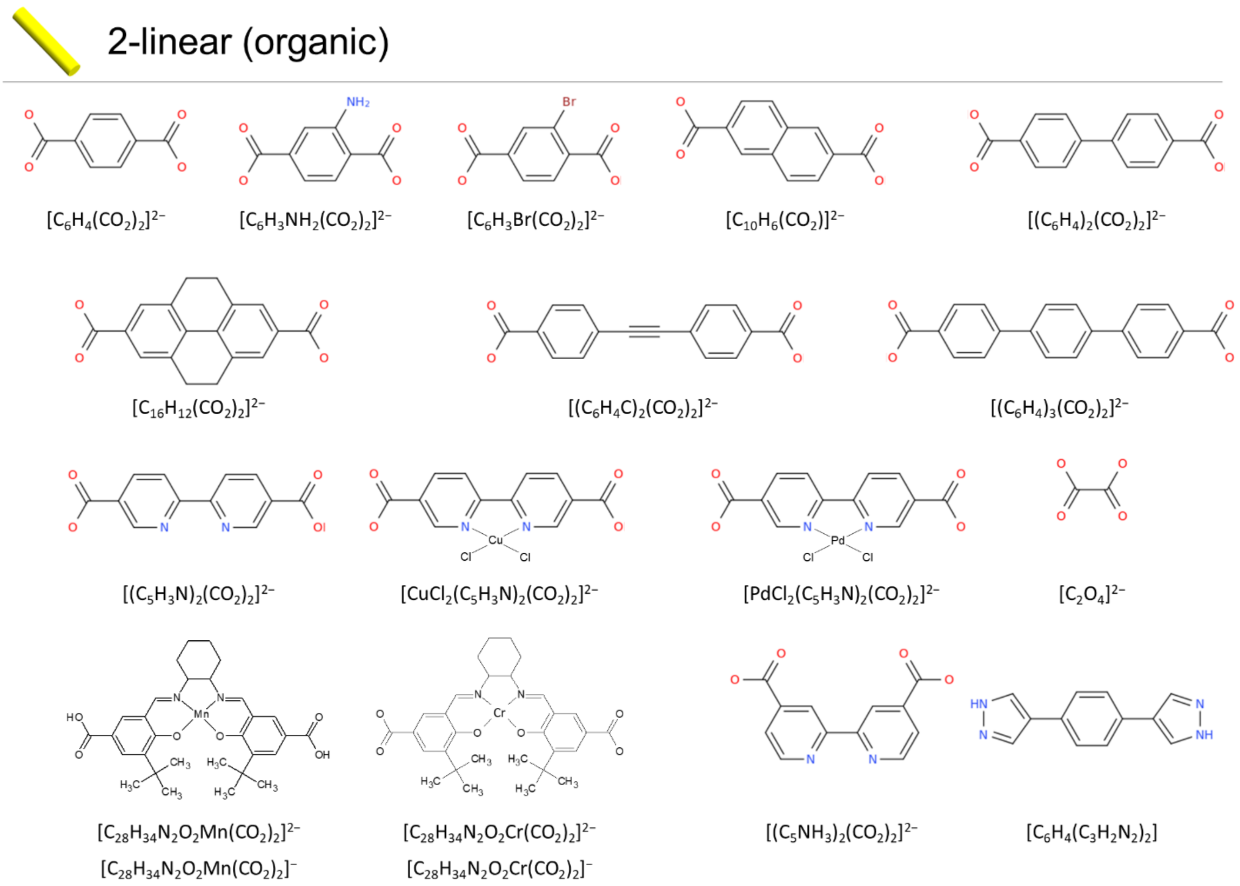

Figure S1: Organic CBUs functioning as 2-linear GBUs.

2-bent (organic)

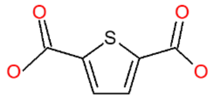

$[C_4H_2S(CO_2)_2]^{2-}$

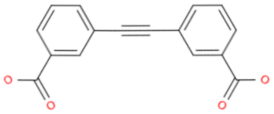

$[(C_6H_4)_2C_2)(CO_2)_2)]^{2-}$

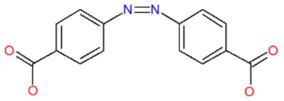

$[(N_2(C_6H_4)_2)(CO_2)_2)]^{2-}$

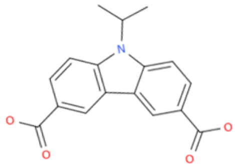

$[((C_6H_4)_2NCH(CH_3)_2)(CO_2)_2)]^{2-}$

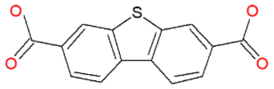

$[S(C_6H_3)_2(CO_2)_2]^{2-}$

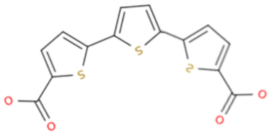

$[(C_4H_2S)_3(CO_2)_2]^{2-}$

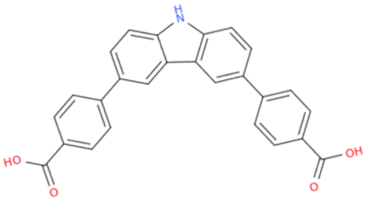

$[C_{12}H_6NH(C_6H_4)_2(CO_2)_2]^{2-}$

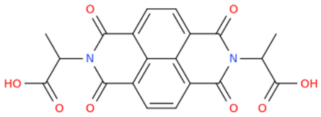

$[(C_{10}H_4)(CO)_4N_2)(CHCH_3)_2(CO_2)_2]^{2-}$

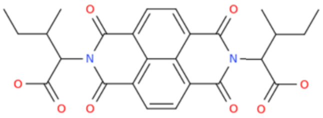

$[(C_{10}H_4)(CO)_4N_2)(CHCH)_2(CH_3)_2(CH_3)_2(CO_2)_2]^{2-}$

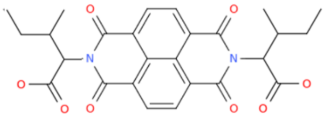

$[(C_{10}H_4)(CO)_4N_2)(CHCH)_2(CH_3)_2(CH_2CH_3)_2(CO_2)_2]^{2-}$

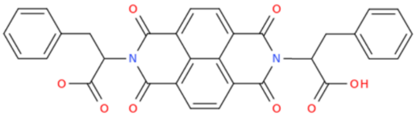

$[(C_{10}H_4)(CO)_4N_2)(CHCH_2)_2(C_6H_5)_2(CO_2)_2]^{2-}$

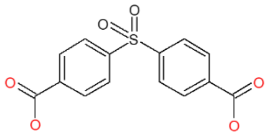

$[SO_2(C_6H_4)_2(CO_2)_2]^{2-}$

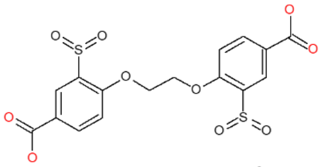

$[C_2H_4O_2(C_6H_3SO_3)_2(CO_2)_2]^{2-}$

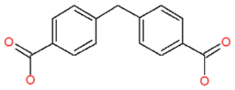

$[CH_2(C_6H_4)_2(CO_2)_2]^{2-}$

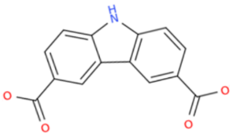

$[(C_6H_3)_2NH(CO_2)_2)]^{2-}$

Figure S2: Organic CBUs functioning as 2-bent GBUs (part 1/4).

2-bent (organic)

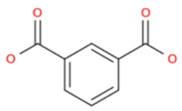

$[C_6H_4(CO_2)_2]^{2-}$

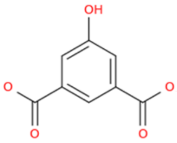

$[(C_6H_3OH)(CO_2)_2]^{2-}$

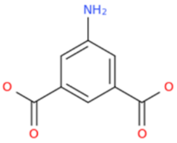

$[(C_6H_3NH_2)(CO_2)_2]^{2-}$

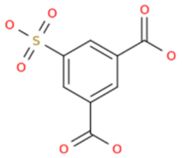

$[(C_6H_3SO_3)(CO_2)_2]^{2-}$

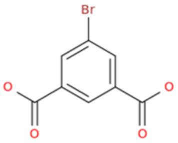

$[(C_6H_3Br)(CO_2)_2]^{2-}$

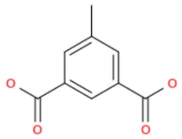

$[(C_6H_3(CH_3))(CO_2)_2]^{2-}$

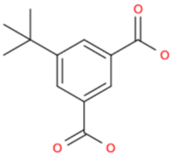

$[((C_6H_3)C(CH_3)_3)(CO_2)_2]^{2-}$

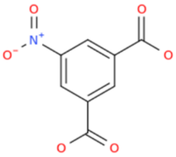

$[(C_6H_3(NO_2))(CO_2)_2]^{2-}$

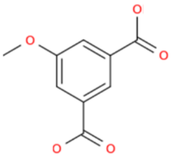

$[(C_6H_3)(OCH_3)(CO_2)_2]^{2-}$

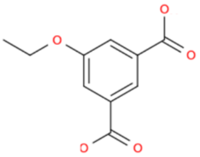

$[(C_6H_3)(OCH_2CH_3)(CO_2)_2]^{2-}$

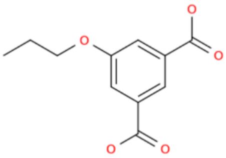

$[(C_6H_3)(OCH_2CH_2CH_3)(CO_2)_2]^{2-}$

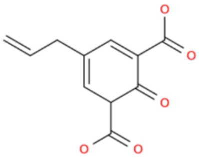

$[(C_6H_2O)(CH_2CHCH_2)(CO_2)_2]^{2-}$

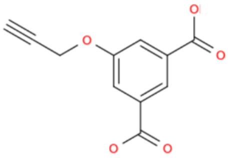

$[(C_6H_3)(OCH_2C_2H)(CO_2)_2]^{2-}$

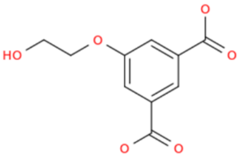

$[(C_6H_3)(OC_2H_4OH)(CO_2)_2]^{2-}$

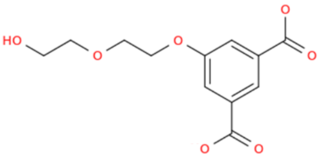

$[(C_6H_3)(OC_2H_4)_2(OH)(CO_2)_2]^{2-}$

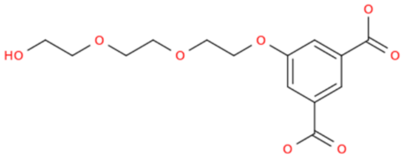

$[(C_6H_3)(OC_2H_4)_3(OH)(CO_2)_2]^{2-}$

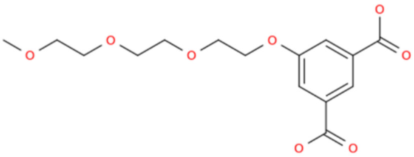

[(C6H3)(OC2H4)3(OCH3)(CO2)2]

Figure S3: Organic CBUs functioning as 2-bent GBUs (part 2/4).

2-bent (organic)

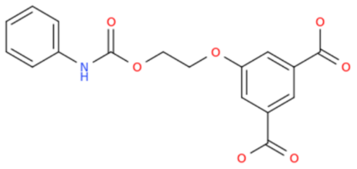

$[(C_6H_3)((C_2H_4O_2)(CCONHC_6H_5))(CO_2)_2]^{2-}$

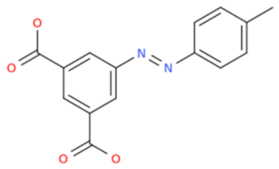

$[(C_6H_3)N_2(C_6H_4CH_3)(CO_2)_2]^{2-}$

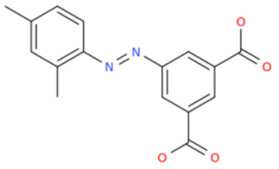

$[(C_6H_3)N_2C_6H_4(CH_3)_2(CO_2)_2]^{2-}$

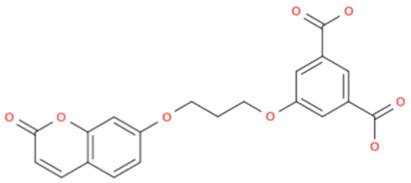

$[(C_6H_3)(C_3H_6O_2)(C_9H_5O_2)(CO_2)_2]^{2-}$

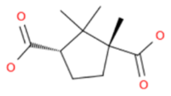

$[(C_5H_4)(CH_3)_3(CO_2)_2]^{2-}$

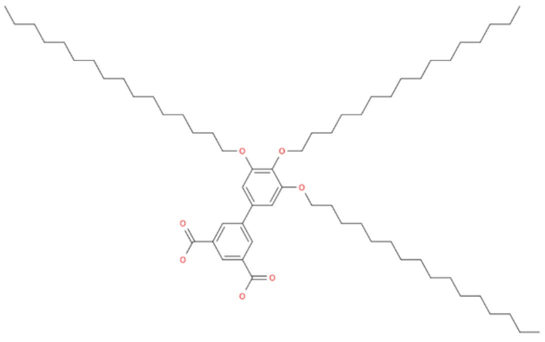

$[(C_6H_3)C_6H_2(OC_{16}H_{33})_3(CO_2)_2]^{2-}$

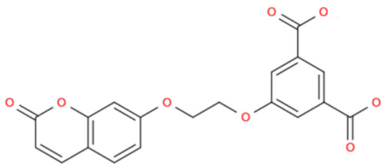

$[(C_6H_3)((C_2H_4O_2)(C_9H_5O_2))(CO_2)_2]^{2-}$

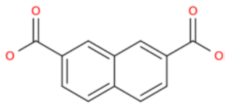

$[(C_{10}H_6)(CO_2)_2]^{2-}$

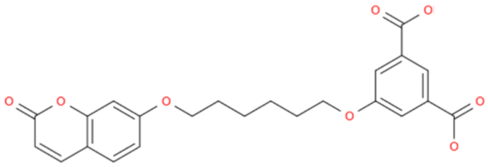

$[(C_6H_3)(C_6H_{12}O_2)(C_9H_5O_2)(CO_2)_2]^{2-}$

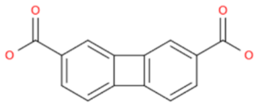

$[(C_6H_3)_2(CO_2)_2]^{2-}$

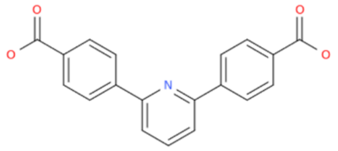

$[(C_5H_3N)(C_6H_4)_2(CO_2)_2]^{2-}$

Figure S4: Organic CBUs functioning as 2-bent GBUs (part 3/4).

2-bent (organic)

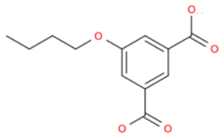

$[(C_6H_3)O(CH_2)_3CH_3(CO_2)_2]^{2-}$

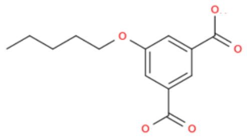

$[(C_6H_3)O(CH_2)_4CH_3(CO_2)_2]^{2-}$

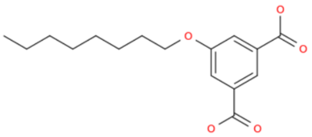

$[(C_6H_3)O(CH_2)_7CH_3(CO_2)_2]^{2-}$

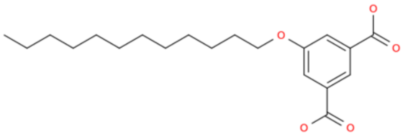

$[(C_6H_3)O(CH_2)_{11}CH_3(CO_2)_2]^{2-}$

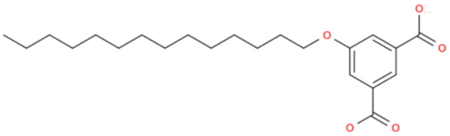

$[(C_6H_3)O(CH_2)_{13}CH_3(CO_2)_2]^{2-}$

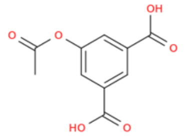

$[C_6H_3CO_2CH_3(CO_2)_2]^{2-}$

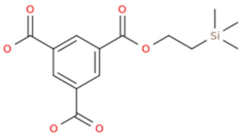

$[C_6H_3CO_2C_2H_4Si(CH_3)_3(CO_2)_2]^{2-}$

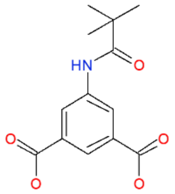

$[(C_6H_3)NHCOC(CH_3)_3(CO_2)_2]^{2-}$

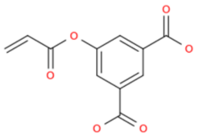

$[C_6H_3OCOCHCH_2(CO_2)_2]^{2-}$

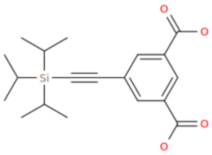

$[C_6H_3C_2Si(C_3H_7)_3(CO_2)_2]^{2-}$

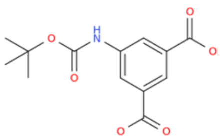

$[C_6H_3NHCO_2C(CH_3)_3(CO_2)_2]^{2-}$

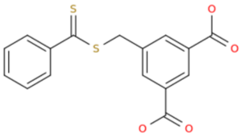

$[(C6H_3)CH_2CS_2(C_6H_5)(CO_2)_2]^{2-}$

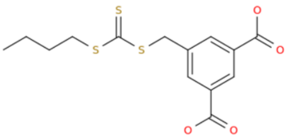

$[C_6H_3CH_2CS_3(CH_2)_3CH_3(CO_2)_2]^{2-}$

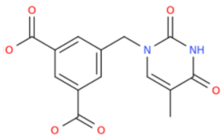

$[(C_6H_3)(CH_2)(C_4N_2H_2O_2CH_3)(CO_2)_2]^{2-}$

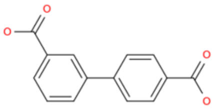

$[(C_6H_4)_2(CO_2)_2]^{2-}$

Figure S5: Organic CBUs functioning as 2-bent GBUs (part 4/4).

Figure S1: Organic CBUs functioning as 2-linear GBUs.

Figure S2: Organic CBUs functioning as 2-bent GBUs (part 1/4).

Figure S3: Organic CBUs functioning as 2-bent GBUs (part 2/4).

Figure S4: Organic CBUs functioning as 2-bent GBUs (part 3/4).

Figure S5: Organic CBUs functioning as 2-bent GBUs (part 4/4).

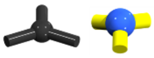

3-planar and 3-pyramidal (organic)

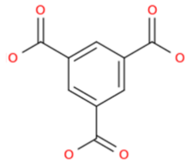

$[(C_6H_3)(CO_2)_3]^{3-}$

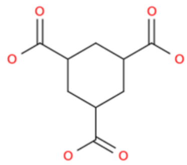

$[(C_6H_9)(CO_2)_3]^{3-}$

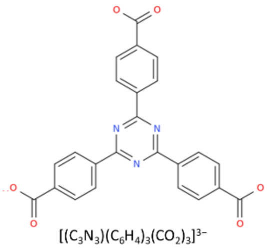

$[(C_3N_3)(C_6H_4)_3(CO_2)_3]^{3-}$

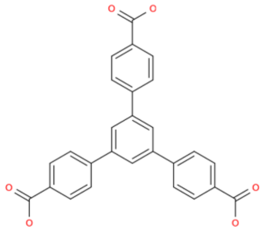

$[(C_6H_3)(C_6H_4)_3(CO_2)_3]^{3-}$

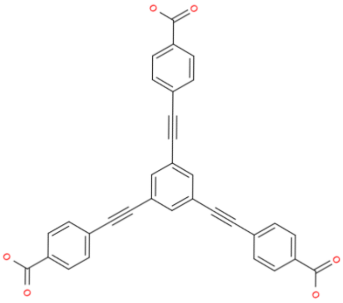

$[(C_6H_3)(C_2C_6H_4)_3(CO_2)_3]^{3-}$

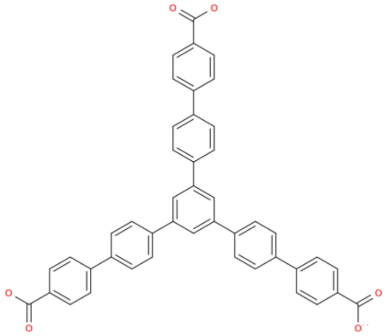

$[(C_6H_3)((C_6H_4)_2)_3(CO_2)_3]^{3-}$

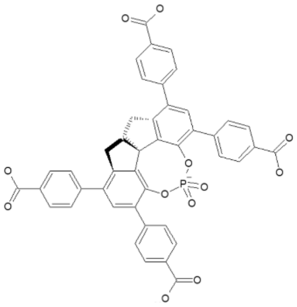

$[(C_{10}H_6)_4(C_6HC_2H_4)_2(CPO_4)(CO_2)_4]^{4-}$

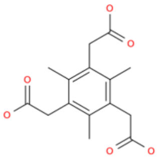

$[(C_6(CH_3)_3)(CH_2)_3(CO_2)_3]^{3-}$

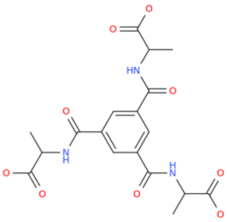

$[C_6H_3(CONHCHCH_3)_3(CO_2)_3]^{3-}$

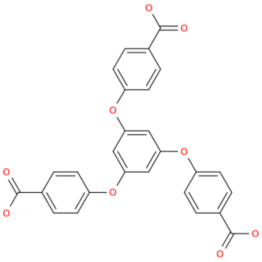

$[(C_6H_3)(OC_6H_4)_3(CO_2)_3]^{3-}$

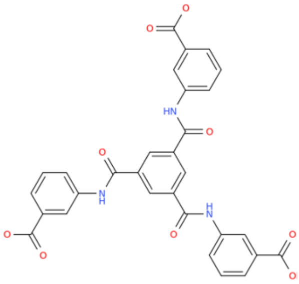

$[(C_6H_3)(CONHC_6H_4)_3(CO_2)_3]^{3-}$

Figure S6: Organic CBUs functioning as 3-planar/pyramidal GBUs.

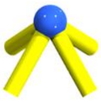

4-pyramidal (organic)

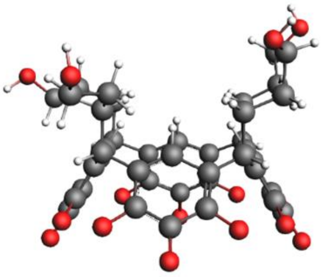

$[C_{40}H_{36}O_{16}]^{12-}$

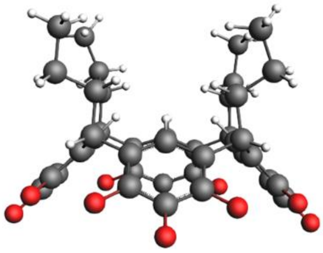

$[C_{40}H_{36}O_{12}]^{12-}$

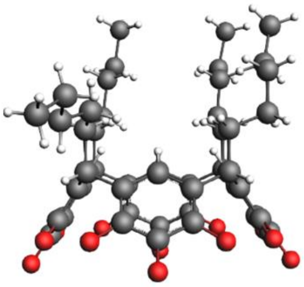

$[(C_6HO_3)_4(C_4H_9)_4]^{12-}$

Figure S7: Organic CBUs functioning as 3-planar/pyramidal GBUs.

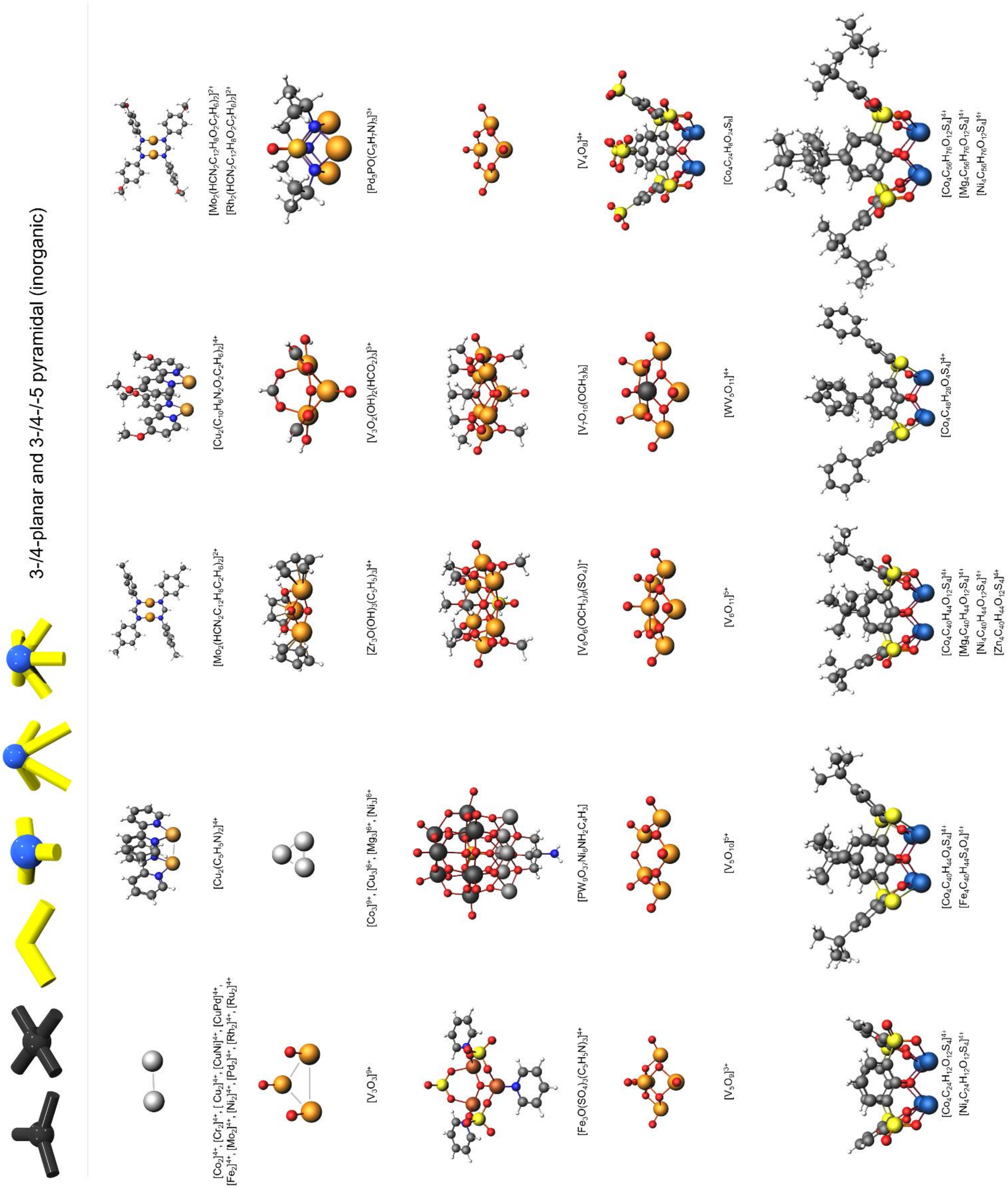

Figure S8: Inorganic CBUs functioning as 2-/3-4-5-bent/pyramidal and 3-/4-planar GBUs.

4. CBU Sharing Phenomenon

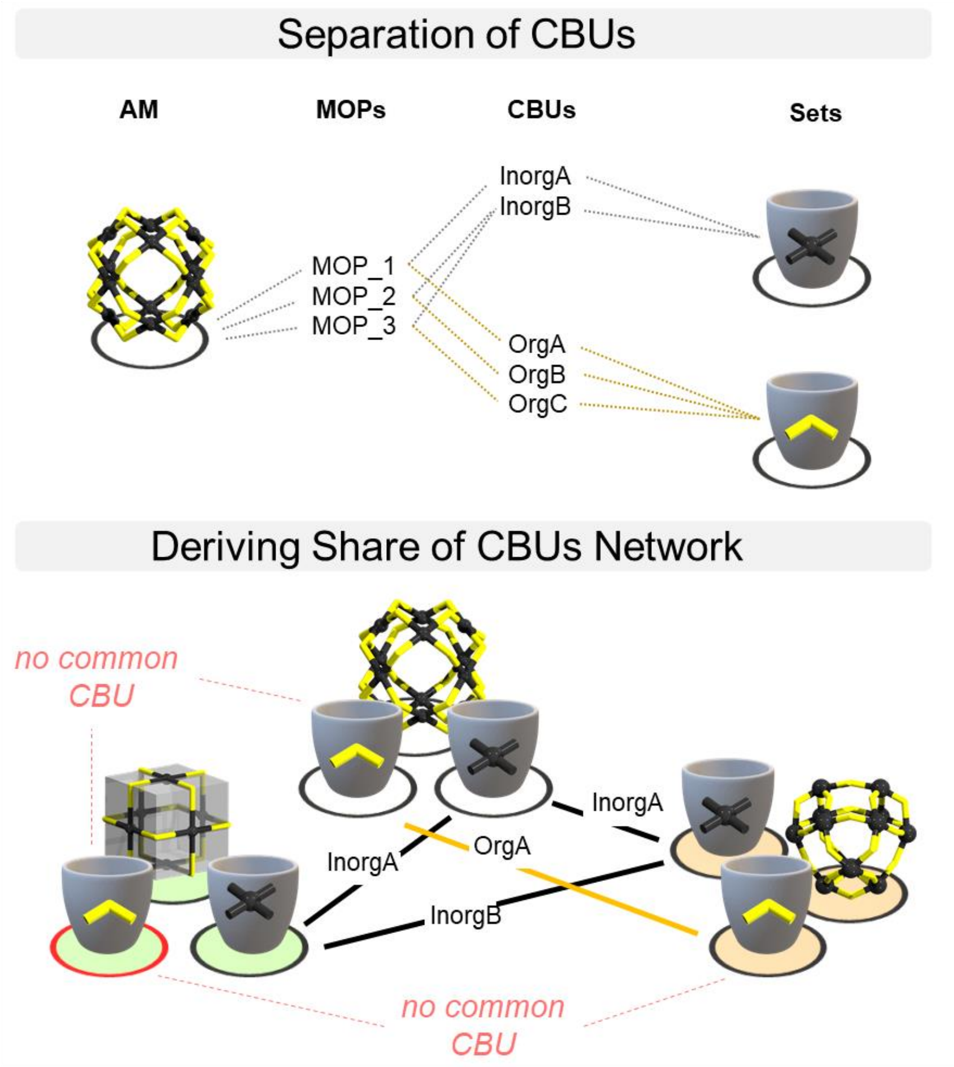

Figure S9: Schematic representation of the creation of sets of CBUs associated with certain GBU and AM (top); creation of CBU share network between different sets of CBUs based on having CBUs in common. Illustration is only provided as an example OrgA, InorgA etc. represent organic and inorganic CBUs respectively. Between two sets of CBUs there will be no CBU common especially when stark differences in terms of dihedral angle exist (e.g. 90◦ vs 120◦).

5. Comments

Comment 1: Method positioning and comparison

In molecular engineering, rational design often indicates a chemical target. Based on the chemical target, a synthetic and computational approach can be developed. In other words, the rationally designed target answers the question of "**what** should be synthesized" or "what should be computationally modelled". The synthetic and computational approach on the other hand answer "**how** the target should be synthesized" or "**how** the target should be modelled and computed". In this context, the approach and tools needed to answer "how" questions are very closely dependent on the answer to the "what is the target" questions.

Our work answers **what** new rational (i.e. reasonable) MOP targets can be constructed? In other words, the combination of ontology, instances and agent models the thinking process of a domain expert when deciding which new MOPs can be constructed from known CBUs. As the CBUs and the blueprints are part of one umbrella digital twin, the evidence-based reasoning of software agents allows automation of the process and high throughput decision making (Figure S10).

Stitching together molecular fragments to create a host or a cage-like molecule and the mathematical operations behind this process answer the question of **how** a target can be modelled in a scalable way. Software packages such as HostDesinger,[1] stk,[2] and cgbind[3] are software packages that allow automated stitching of building unit geometry based on a structural blueprint to create a starting guess geometry files of such a host or cage molecule. The latter packages are operated by a human requiring to provide a rational combination of building units and targeted templates. If this is not the case, the software will generate unreasonable structures, which subsequently increase the subsequent computational cost however without providing a meaningful outcome.

In a recent critical review, Duarte, Lusby and coworkers suggest that some of the shortcomings of HostDesinger, stk, and cgbind is that they are generally operated through a command-line, which may not be very convenient for chemists that lack programming skills.[4] The latter aspect is not a shortcoming for knowledge engineering technologies, but rather an advantage as it is desired that a software package is accessible through a command-line so that it can be accessed and operated by a software agent. In other words, we see a potential synergy in closely integrating packages such as HostDesinger, stk, and cgbind as parts of universal and dynamic digital twins dedicated to chemical discovery and development.

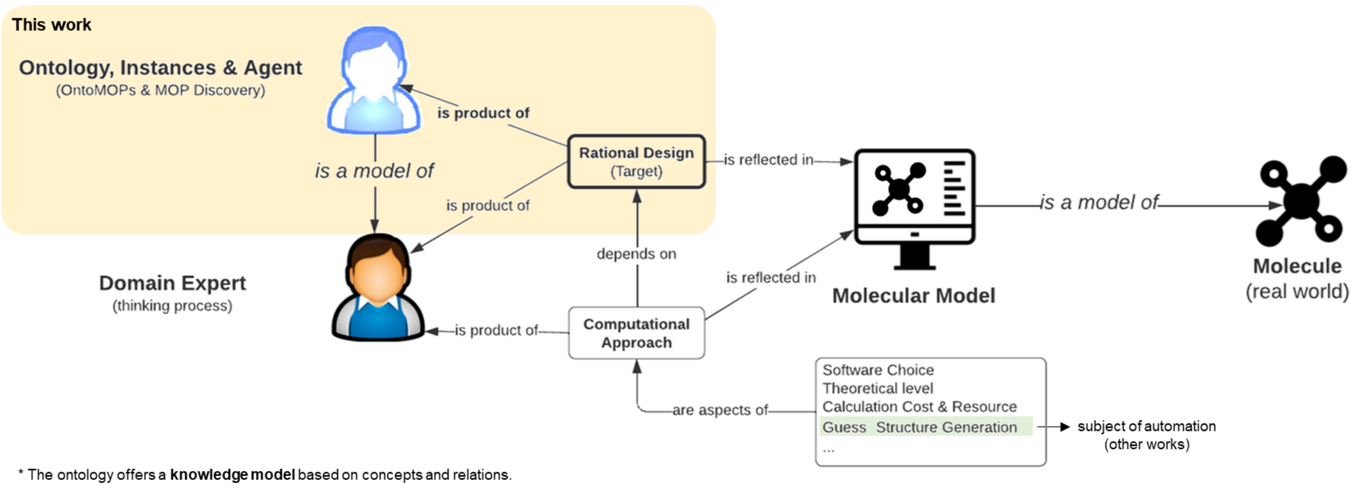

Figure S10: Interrelation between Knowledge modelling and Molecular Modelling.

[1] Hay, B. P.; Firman, T. K. *Inorg. Chem.* **2002**, 41 (21), 5502– 5512,
[2] Turcani, L.; Berardo, E.; Jelfs, K. E. J. *Comput. Chem.* **2018**, 39 (23), 1931– 1942,
[3] Young, T. A.; Gheorghe, R.; Duarte, F. *J. Chem. Inf. Model.* **2020**, 60 (7), 3546– 3557,
[4] Piskorz, T. K.; Martí-Centelles, V.; Young, T. A.; Lusby, P.J.; Duarte F. *ACS Catal.* **2022**, 12, 5806–5826.

Comment 2: Isomeric MOPs

Except for the anticuboctahedral and cuboctahedral MOPs this article did not further expand on treatment of structures that may have an isomer problem.

MOPs where one GBU can be simultaneously substituted by two different CBUs would technically lead to a three GBU based assembly models.

In a scenario where the two different CBUs correspond to single metals the total number of GBUs covered by these CBUs will be split among them. To illustrate this one can consider a cuboctahedral MOP with an AM $[4\text{-planar}]_{12}[2\text{-bent}]_{24}$ and O_h point group symmetry. Each 4-planar GBU is represented with a single metal center and all 2-bent GBUs are organic ligands (L) then one can describe this mop as $M_{12}L_{24}$. In the case when substation occurs with another metal center (M'), then the mixed-metal MOP entity can be described as $M_xM'_{12-x}L_{24}$. Entities such as $M_xM'_{12-x}L_{24}$ are inherently isomeric and for every x different numbers of configurational isomers are possible. For instance when x = 4 there will be in total 18 different configurational isomers.[5]

Another scenario where two different CBUs correspond to organic ligands, there is a possibility that they significantly differ in size. The differences in sizes are likely to cause internal strains. Having the cuboctahedral MOP $M_{12}L_{24}$ from before, substitution with a second organic ligand would be formally written as $M_{12}L_{24-y}L'_y$. The latter MOP complex would again be a subject of isomer problem, however in this case owing to the emergence of strains, certain degrees of substitution and certain configurations may trigger programmable **archetype dynamics** that affects the number of metal centers. These types of archetype dynamics is currently reported POMs,[6] and in the present case would imply dramatic shift between assembly models.

Substitution essentially requires recording of different assembly models for each different configuration. Due to the existing combinatorial complexity and possible dynamics between assembly models, such systems are not part of this work, but a subject of future modular integration

[5] a) Simms, C.; Kondinski, A.; Parac-Vogt, T. N. *Eur. J. Inorg. Chem.*, **2020**, 2559-2572. b) Izarova, N. V.; Kondinski, A.; Vankova, N.; Heine, T.; Jäger, P.; Schinle, F.; Hampe, O., Kortz, U. *Chem. Eur. J.*, **2014**, 20, 8556-8560. c) Kondinski, A. *Chem. Modell.*, 2021, 16, 39–71 .
[6] Kondinski, A.; Rasmussen, M.; Mangelsen, S.; Pienack, N.; Simjanoski, V.; Nather, C.; Stares, D. L.; Schalley, C. A.; Bensch, W. Chem. Sci., 2022. https://doi.org/10.1039/D2SC01004F

6. Algorithmic Output II

Table S2: Output of Algorithm 2.

Assembly Model	MOP Formula	MOP Charge	MOP MW	ReferenceDOI
(4-planar)x12(2-bent)x24___(Oh)	[Rh2]12[(C6H4)(CO2)2]24	0	6408.489216	10.1021/jacs.8b13593
(4-planar)x12(2-bent)x24___(Oh)	[Rh2]12[(C6H3OH)(CO2)2]24	0	6792.477	10.1021/jacs.8b13593
(4-planar)x12(2-bent)x24___(Oh)	[Rh2]12[(C6H3(NO2))(CO2)2]24	0	7488.437664	Not in OntoMOPs KG
(4-planar)x12(2-bent)x24___(Oh)	[Rh2]12[(C6H3(CH3))(CO2)2]24	0	6745.121856	Not in OntoMOPs KG
(4-planar)x12(2-bent)x24___(Oh)	[Rh2]12[(C6H3Br)(CO2)2]24	0	8301.997296	Not in OntoMOPs KG
(4-planar)x12(2-bent)x24___(Oh)	[Rh2]12[(C6H3)((C3H6O2)(C9H5O2))(CO2)2]24	0	11645.40115	Not in OntoMOPs KG
(4-planar)x12(2-bent)x24___(Oh)	[Rh2]12[(C6H3)((C6H12O2)(C9H5O2))(CO2)2]24	0	12655.29907	Not in OntoMOPs KG
(4-planar)x12(2-bent)x24___(Oh)	[Rh2]12[(C6H3)(OC2H4)3(OCH3)(CO2)2]24	0	10300.86883	Not in OntoMOPs KG
(4-planar)x12(2-bent)x24___(Oh)	[Rh2]12[(C6H3)(OCH2C2H)(CO2)2]24	0	7705.62324	Not in OntoMOPs KG
(4-planar)x12(2-bent)x24___(Oh)	[Rh2]12[(C6H3NH2)(CO2)2]24	0	6768.837936	Not in OntoMOPs KG
(4-planar)x12(2-bent)x24___(Oh)	[Rh2]12[(C6H3)(OCH2CH2CH3)(CO2)2]24	0	7802.37492	Not in OntoMOPs KG
(4-planar)x12(2-bent)x24___(Oh)	[Rh2]12[(C10H6)(CO2)2]24	0	7609.892256	Not in OntoMOPs KG
(4-planar)x12(2-bent)x24___(Oh)	[Rh2]12[(C6H3)2(CO2)2]24	-24	7440.533664	Not in OntoMOPs KG
(4-planar)x12(2-bent)x24___(Oh)	[Rh2]12[(C6H3)(OCH2CH3)(CO2)2]24	0	7465.74228	Not in OntoMOPs KG
(4-planar)x12(2-bent)x24___(Oh)	[Rh2]12[(C6H3)(OC2H4)2(OH)(CO2)2]24	0	8906.983128	Not in OntoMOPs KG
(4-planar)x12(2-bent)x24___(Oh)	[Rh2]12[(C6H3)(OC2H4)3(OH)(CO2)2]24	0	9964.236192	Not in OntoMOPs KG
(4-planar)x12(2-bent)x24___(Oh)	[Rh2]12[(C6H3)O(CH2)11CH3(CO2)2]24	0	10832.06868	10.1038/s41467-018-04834-0
(4-planar)x12(2-bent)x24___(Oh)	[Rh2]12[(C6H3)O(CH2)4CH3(CO2)2]24	0	8475.6402	Not in OntoMOPs KG
(4-planar)x12(2-bent)x24___(Oh)	[Rh2]12[(C6H3)(OC2H4OH)(CO2)2]24	0	7849.730064	Not in OntoMOPs KG
(4-planar)x12(2-bent)x24___(Oh)	[Rh2]12[(C6H3)NHCOC(CH3)3(CO2)2]24	0	8787.61308	10.1039/C9CC07083D
(4-planar)x12(2-bent)x24___(Oh)	[Rh2]12[(C6H3)CH2CS2(C6H5)(CO2)2]24	0	10398.79114	Not in OntoMOPs KG
(4-planar)x12(2-bent)x24___(Oh)	[Rh2]12[C6H3CH2CS3C4H9(CO2)2]24	0	10688.58922	Not in OntoMOPs KG
(4-planar)x12(2-bent)x24___(Oh)	[Rh2]12[C6H3C2Si(C3H7)3(CO2)2]24	0	10737.12442	Not in OntoMOPs KG
(4-planar)x12(2-bent)x24___(Oh)	[Rh2]12[(C6H3)(CH2)(C4N2H2O2CH3)(CO2)2]24	0	9723.454704	Not in OntoMOPs KG
(4-planar)x12(2-bent)x24___(Oh)	[Rh2]12[(C6H3)((C2H4O2)(C9H5O2))(CO2)2]24	0	11308.76851	Not in OntoMOPs KG
(4-planar)x12(2-bent)x24___(Oh)	[Rh2]12[(C6H3)(N2(C6H4CH3))(CO2)2]24	0	9243.735936	Not in OntoMOPs KG
(4-planar)x12(2-bent)x24___(Oh)	[Rh2]12[((C6H3)C(CH3)3)(CO2)2]24	0	7755.019776	Not in OntoMOPs KG
(4-planar)x12(2-bent)x24___(Oh)	[Rh2]12[(C6H3SO3)(CO2)2]24	0	8305.824648	Not in OntoMOPs KG
(4-planar)x12(2-bent)x24___(Oh)	[Rh2]12[(C6H3)O(CH2)13CH3(CO2)2]24	0	11505.33396	10.1016/j.chempr.2017.02.002
(4-planar)x12(2-bent)x24___(Oh)	[Rh2]12[C6H3CO2C2H4Si(CH3)3(CO2)2]24	0	9870.312624	10.1039/C9CC07083D
(4-planar)x12(2-bent)x24___(Oh)	[Rh2]12[(C6H3)(CO2)3]24	-24	7440.533664	10.1039/C9CC07083D
(4-planar)x12(2-bent)x24___(Oh)	[Rh2]12[C6H3CO2CH3(CO2)2]24	0	7801.354224	10.1021/jacs.8b13593
(4-planar)x12(2-bent)x24___(Oh)	[Rh2]12[C6H3OCOCHCH2(CO2)2]24	0	8089.611024	10.1021/jacs.8b13593
(4-planar)x12(2-bent)x24___(Oh)	[Rh2]12[C6H3NHCO2C(CH3)3(CO2)2]24	0	9171.600864	10.1039/C9CC07083D
(4-planar)x12(2-bent)x24___(Oh)	[Rh2]12[(C6H2O)(CH2CHCH2)(CO2)2]24	0	7753.99908	Not in OntoMOPs KG
(4-planar)x12(2-bent)x24___(Oh)	[Rh2]12[(C6H3)O(CH2)3CH3(CO2)2]24	0	8139.00756	Not in OntoMOPs KG
(4-planar)x12(2-bent)x24___(Oh)	[Rh2]12[(C6H3)C6H2(OC16H33)3(CO2)2]24	0	25496.73593	Not in OntoMOPs KG
(4-planar)x12(2-bent)x24___(Oh)	[Rh2]12[C6H3N2C6H3(CH3)2(CO2)2]24	0	9580.368576	Not in OntoMOPs KG
(4-planar)x12(2-bent)x24___(Oh)	[Rh2]12[(C6H3)(C2H4O2)(CONHC6H5)(CO2)2]24	0	10708.61585	Not in OntoMOPs KG
(4-planar)x12(2-bent)x24___(Oh)	[Rh2]12[(C6H3)(OCH3)(CO2)2]24	0	7129.10964	Not in OntoMOPs KG
(4-planar)x12(2-bent)x24___(Oh)	[Ru2]12[(C6H4)(CO2)2]24	0	6364.437216	10.1016/j.ica.2014.09.010
(4-planar)x12(2-bent)x24___(Oh)	[Ru2]12[(C6H3OH)(CO2)2]24	0	6748.425	Not in OntoMOPs KG
(4-planar)x12(2-bent)x24___(Oh)	[Ru2]12[(C6H3(NO2))(CO2)2]24	0	7444.385664	Not in OntoMOPs KG
(4-planar)x12(2-bent)x24___(Oh)	[Ru2]12[(C6H3(CH3))(CO2)2]24	0	6701.069856	Not in OntoMOPs KG
(4-planar)x12(2-bent)x24___(Oh)	[Ru2]12[(C6H3Br)(CO2)2]24	0	8257.945296	Not in OntoMOPs KG
(4-planar)x12(2-bent)x24___(Oh)	[Ru2]12[(C6H3)((C3H6O2)(C9H5O2))(CO2)2]24	0	11601.34915	Not in OntoMOPs KG

(4-planar)x12(2-bent)x24___(Oh)	[Ru2]12[(C6H3)((C6H12O2)(C9H5O2))(CO2)2]24	0	12611.24707	Not in OntoMOPs KG
(4-planar)x12(2-bent)x24___(Oh)	[Ru2]12[(C6H3)(OC2H4)3(OCH3)(CO2)2]24	0	10256.81683	Not in OntoMOPs KG
(4-planar)x12(2-bent)x24___(Oh)	[Ru2]12[(C6H3)(OCH2C2H)(CO2)2]24	0	7661.57124	Not in OntoMOPs KG
(4-planar)x12(2-bent)x24___(Oh)	[Ru2]12[(C6H3NH2)(CO2)2]24	0	6724.785936	Not in OntoMOPs KG
(4-planar)x12(2-bent)x24___(Oh)	[Ru2]12[(C6H3)(OCH2CH2CH3)(CO2)2]24	0	7758.32292	Not in OntoMOPs KG
(4-planar)x12(2-bent)x24___(Oh)	[Ru2]12[(C10H6)(CO2)2]24	0	7565.840256	Not in OntoMOPs KG
(4-planar)x12(2-bent)x24___(Oh)	[Ru2]12[(C6H3)2(CO2)2]24	-24	7396.481664	Not in OntoMOPs KG
(4-planar)x12(2-bent)x24___(Oh)	[Ru2]12[(C6H3)(OCH2CH3)(CO2)2]24	0	7421.69028	Not in OntoMOPs KG
(4-planar)x12(2-bent)x24___(Oh)	[Ru2]12[(C6H3)(OC2H4)2(OH)(CO2)2]24	0	8862.931128	Not in OntoMOPs KG
(4-planar)x12(2-bent)x24___(Oh)	[Ru2]12[(C6H3)(OC2H4)3(OH)(CO2)2]24	0	9920.184192	Not in OntoMOPs KG
(4-planar)x12(2-bent)x24___(Oh)	[Ru2]12[(C6H3)O(CH2)11CH3(CO2)2]24	0	10788.01668	Not in OntoMOPs KG
(4-planar)x12(2-bent)x24___(Oh)	[Ru2]12[(C6H3)O(CH2)4CH3(CO2)2]24	0	8431.5882	Not in OntoMOPs KG
(4-planar)x12(2-bent)x24___(Oh)	[Ru2]12[(C6H3)(OC2H4OH)(CO2)2]24	0	7805.678064	Not in OntoMOPs KG
(4-planar)x12(2-bent)x24___(Oh)	[Ru2]12[(C6H3)NHCOC(CH3)3(CO2)2]24	0	8743.56108	Not in OntoMOPs KG
(4-planar)x12(2-bent)x24___(Oh)	[Ru2]12[(C6H3)CH2CS2(C6H5)(CO2)2]24	0	10354.73914	Not in OntoMOPs KG
(4-planar)x12(2-bent)x24___(Oh)	[Ru2]12[C6H3CH2CS3C4H9(CO2)2]24	0	10644.53722	Not in OntoMOPs KG
(4-planar)x12(2-bent)x24___(Oh)	[Ru2]12[C6H3C2Si(C3H7)3(CO2)2]24	0	10693.07242	Not in OntoMOPs KG
(4-planar)x12(2-bent)x24___(Oh)	[Ru2]12[(C6H3)(CH2)(C4N2H2O2CH3)(CO2)2]24	0	9679.402704	Not in OntoMOPs KG
(4-planar)x12(2-bent)x24___(Oh)	[Ru2]12[(C6H3)((C2H4O2)(C9H5O2))(CO2)2]24	0	11264.71651	Not in OntoMOPs KG
(4-planar)x12(2-bent)x24___(Oh)	[Ru2]12[(C6H3)(N2(C6H4CH3))(CO2)2]24	0	9199.683936	Not in OntoMOPs KG
(4-planar)x12(2-bent)x24___(Oh)	[Ru2]12[((C6H3)C(CH3)3)(CO2)2]24	0	7710.967776	10.1016/j.ica.2014.09.010
(4-planar)x12(2-bent)x24___(Oh)	[Ru2]12[(C6H3SO3)(CO2)2]24	0	8261.772648	Not in OntoMOPs KG
(4-planar)x12(2-bent)x24___(Oh)	[Ru2]12[(C6H3)O(CH2)13CH3(CO2)2]24	0	11461.28196	Not in OntoMOPs KG
(4-planar)x12(2-bent)x24___(Oh)	[Ru2]12[C6H3CO2C2H4Si(CH3)3(CO2)2]24	0	9826.260624	Not in OntoMOPs KG
(4-planar)x12(2-bent)x24___(Oh)	[Ru2]12[(C6H3)(CO2)3]24	-24	7396.481664	Not in OntoMOPs KG
(4-planar)x12(2-bent)x24___(Oh)	[Ru2]12[C6H3CO2CH3(CO2)2]24	0	7757.302224	Not in OntoMOPs KG
(4-planar)x12(2-bent)x24___(Oh)	[Ru2]12[C6H3OCOCHCH2(CO2)2]24	0	8045.559024	Not in OntoMOPs KG
(4-planar)x12(2-bent)x24___(Oh)	[Ru2]12[C6H3NHCO2C(CH3)3(CO2)2]24	0	9127.548864	Not in OntoMOPs KG
(4-planar)x12(2-bent)x24___(Oh)	[Ru2]12[(C6H2O)(CH2CHCH2)(CO2)2]24	0	7709.94708	Not in OntoMOPs KG
(4-planar)x12(2-bent)x24___(Oh)	[Ru2]12[(C6H3)O(CH2)3CH3(CO2)2]24	0	8094.95556	Not in OntoMOPs KG
(4-planar)x12(2-bent)x24___(Oh)	[Ru2]12[(C6H3)C6H2(OC16H33)3(CO2)2]24	0	25452.68393	Not in OntoMOPs KG
(4-planar)x12(2-bent)x24___(Oh)	[Ru2]12[C6H3N2C6H3(CH3)2(CO2)2]24	0	9536.316576	Not in OntoMOPs KG
(4-planar)x12(2-bent)x24___(Oh)	[Ru2]12[(C6H3)(C2H4O2)(CONHC6H5)(CO2)2]24	0	10664.56385	Not in OntoMOPs KG
(4-planar)x12(2-bent)x24___(Oh)	[Ru2]12[(C6H3)(OCH3)(CO2)2]24	0	7085.05764	Not in OntoMOPs KG
(4-planar)x12(2-bent)x24___(Oh)	[Ni2]12[(C6H4)(CO2)2]24	0	5347.398816	Not in OntoMOPs KG
(4-planar)x12(2-bent)x24___(Oh)	[Ni2]12[(C6H3OH)(CO2)2]24	0	5731.3866	10.1021/acs.inorgchem.8b01130
(4-planar)x12(2-bent)x24___(Oh)	[Ni2]12[(C6H3(NO2))(CO2)2]24	0	6427.347264	Not in OntoMOPs KG
(4-planar)x12(2-bent)x24___(Oh)	[Ni2]12[(C6H3(CH3))(CO2)2]24	0	5684.031456	Not in OntoMOPs KG
(4-planar)x12(2-bent)x24___(Oh)	[Ni2]12[(C6H3Br)(CO2)2]24	0	7240.906896	Not in OntoMOPs KG
(4-planar)x12(2-bent)x24___(Oh)	[Ni2]12[(C6H3)((C3H6O2)(C9H5O2))(CO2)2]24	0	10584.31075	Not in OntoMOPs KG
(4-planar)x12(2-bent)x24___(Oh)	[Ni2]12[(C6H3)((C6H12O2)(C9H5O2))(CO2)2]24	0	11594.20867	Not in OntoMOPs KG
(4-planar)x12(2-bent)x24___(Oh)	[Ni2]12[(C6H3)(OC2H4)3(OCH3)(CO2)2]24	0	9239.778432	Not in OntoMOPs KG
(4-planar)x12(2-bent)x24___(Oh)	[Ni2]12[(C6H3)(OCH2C2H)(CO2)2]24	0	6644.53284	Not in OntoMOPs KG
(4-planar)x12(2-bent)x24___(Oh)	[Ni2]12[(C6H3NH2)(CO2)2]24	0	5707.747536	Not in OntoMOPs KG
(4-planar)x12(2-bent)x24___(Oh)	[Ni2]12[(C6H3)(OCH2CH2CH3)(CO2)2]24	0	6741.28452	Not in OntoMOPs KG
(4-planar)x12(2-bent)x24___(Oh)	[Ni2]12[(C10H6)(CO2)2]24	0	6548.801856	Not in OntoMOPs KG
(4-planar)x12(2-bent)x24___(Oh)	[Ni2]12[(C6H3)2(CO2)2]24	-24	6379.443264	Not in OntoMOPs KG
(4-planar)x12(2-bent)x24___(Oh)	[Ni2]12[(C6H3)(OCH2CH3)(CO2)2]24	0	6404.65188	Not in OntoMOPs KG
(4-planar)x12(2-bent)x24___(Oh)	[Ni2]12[(C6H3)(OC2H4)2(OH)(CO2)2]24	0	7845.892728	Not in OntoMOPs KG
(4-planar)x12(2-bent)x24___(Oh)	[Ni2]12[(C6H3)(OC2H4)3(OH)(CO2)2]24	0	8903.145792	Not in OntoMOPs KG

(4-planar)x12(2-bent)x24___(Oh)	[Ni2]12[(C6H3)O(CH2)11CH3(CO2)2]24	0	9770.97828	Not in OntoMOPs KG
(4-planar)x12(2-bent)x24___(Oh)	[Ni2]12[(C6H3)O(CH2)4CH3(CO2)2]24	0	7414.5498	Not in OntoMOPs KG
(4-planar)x12(2-bent)x24___(Oh)	[Ni2]12[(C6H3)(OC2H4OH)(CO2)2]24	0	6788.639664	Not in OntoMOPs KG
(4-planar)x12(2-bent)x24___(Oh)	[Ni2]12[(C6H3)NHCOC(CH3)3(CO2)2]24	0	7726.52268	Not in OntoMOPs KG
(4-planar)x12(2-bent)x24___(Oh)	[Ni2]12[(C6H3)CH2CS2(C6H5)(CO2)2]24	0	9337.700736	Not in OntoMOPs KG
(4-planar)x12(2-bent)x24___(Oh)	[Ni2]12[C6H3CH2CS3C4H9(CO2)2]24	0	9627.498816	Not in OntoMOPs KG
(4-planar)x12(2-bent)x24___(Oh)	[Ni2]12[C6H3C2Si(C3H7)3(CO2)2]24	0	9676.034016	Not in OntoMOPs KG
(4-planar)x12(2-bent)x24___(Oh)	[Ni2]12[(C6H3)(CH2)(C4N2H2O2CH3)(CO2)2]24	0	8662.364304	Not in OntoMOPs KG
(4-planar)x12(2-bent)x24___(Oh)	[Ni2]12[(C6H3)((C2H4O2)(C9H5O2))(CO2)2]24	0	10247.67811	Not in OntoMOPs KG
(4-planar)x12(2-bent)x24___(Oh)	[Ni2]12[(C6H3)(N2(C6H4CH3))(CO2)2]24	0	8182.645536	Not in OntoMOPs KG
(4-planar)x12(2-bent)x24___(Oh)	[Ni2]12[((C6H3)C(CH3)3)(CO2)2]24	0	6693.929376	Not in OntoMOPs KG
(4-planar)x12(2-bent)x24___(Oh)	[Ni2]12[(C6H3SO3)(CO2)2]24	0	7244.734248	Not in OntoMOPs KG
(4-planar)x12(2-bent)x24___(Oh)	[Ni2]12[(C6H3)O(CH2)13CH3(CO2)2]24	0	10444.24356	Not in OntoMOPs KG
(4-planar)x12(2-bent)x24___(Oh)	[Ni2]12[C6H3CO2C2H4Si(CH3)3(CO2)2]24	0	8809.222224	Not in OntoMOPs KG
(4-planar)x12(2-bent)x24___(Oh)	[Ni2]12[(C6H3)(CO2)3]24	-24	6379.443264	Not in OntoMOPs KG
(4-planar)x12(2-bent)x24___(Oh)	[Ni2]12[C6H3CO2CH3(CO2)2]24	0	6740.263824	Not in OntoMOPs KG
(4-planar)x12(2-bent)x24___(Oh)	[Ni2]12[C6H3OCOCHCH2(CO2)2]24	0	7028.520624	Not in OntoMOPs KG
(4-planar)x12(2-bent)x24___(Oh)	[Ni2]12[C6H3NHCO2C(CH3)3(CO2)2]24	0	8110.510464	Not in OntoMOPs KG
(4-planar)x12(2-bent)x24___(Oh)	[Ni2]12[(C6H2O)(CH2CHCH2)(CO2)2]24	0	6692.90868	Not in OntoMOPs KG
(4-planar)x12(2-bent)x24___(Oh)	[Ni2]12[(C6H3)O(CH2)3CH3(CO2)2]24	0	7077.91716	Not in OntoMOPs KG
(4-planar)x12(2-bent)x24___(Oh)	[Ni2]12[(C6H3)C6H2(OC16H33)3(CO2)2]24	0	24435.64553	Not in OntoMOPs KG
(4-planar)x12(2-bent)x24___(Oh)	[Ni2]12[C6H3N2C6H3(CH3)2(CO2)2]24	0	8519.278176	Not in OntoMOPs KG
(4-planar)x12(2-bent)x24___(Oh)	[Ni2]12[(C6H3)(C2H4O2)(CONHC6H5)(CO2)2]24	0	9647.525448	Not in OntoMOPs KG
(4-planar)x12(2-bent)x24___(Oh)	[Ni2]12[(C6H3)(OCH3)(CO2)2]24	0	6068.01924	Not in OntoMOPs KG
(4-planar)x12(2-bent)x24___(Oh)	[Cu2]12[(C6H4)(CO2)2]24	0	5463.861216	10.1021/ja0104352
(4-planar)x12(2-bent)x24___(Oh)	[Cu2]12[(C6H3OH)(CO2)2]24	0	5847.849	10.1038/nchem.803
(4-planar)x12(2-bent)x24___(Oh)	[Cu2]12[(C6H3(NO2))(CO2)2]24	0	6543.809664	10.1021/jacs.6b04971
(4-planar)x12(2-bent)x24___(Oh)	[Cu2]12[(C6H3(CH3))(CO2)2]24	0	5800.493856	10.1021/jacs.6b04971
(4-planar)x12(2-bent)x24___(Oh)	[Cu2]12[(C6H3Br)(CO2)2]24	0	7357.369296	10.1021/ja803783c
(4-planar)x12(2-bent)x24___(Oh)	[Cu2]12[(C6H3)((C3H6O2)(C9H5O2))(CO2)2]24	0	10700.77315	10.1039/C7CC04799A
(4-planar)x12(2-bent)x24___(Oh)	[Cu2]12[(C6H3)((C6H12O2)(C9H5O2))(CO2)2]24	0	11710.67107	10.1039/C7CC04799A
(4-planar)x12(2-bent)x24___(Oh)	[Cu2]12[(C6H3)(OC2H4)3(OCH3)(CO2)2]24	0	9356.240832	10.1002/asia.201701647
(4-planar)x12(2-bent)x24___(Oh)	[Cu2]12[(C6H3)(OCH2C2H)(CO2)2]24	0	6760.99524	10.1002/anie.201307486
(4-planar)x12(2-bent)x24___(Oh)	[Cu2]12[(C6H3NH2)(CO2)2]24	0	5824.209936	10.1021/ja803783c
(4-planar)x12(2-bent)x24___(Oh)	[Cu2]12[(C6H3)(OCH2CH2CH3)(CO2)2]24	0	6857.74692	10.1021/acsami.8b02015
(4-planar)x12(2-bent)x24___(Oh)	[Cu2]12[(C10H6)(CO2)2]24	0	6665.264256	10.1021/ja803783c
(4-planar)x12(2-bent)x24___(Oh)	[Cu2]12[(C6H3)2(CO2)2]24	-24	6495.905664	10.1021/ja803783c
(4-planar)x12(2-bent)x24___(Oh)	[Cu2]12[(C6H3)(OCH2CH3)(CO2)2]24	0	6521.11428	10.1002/chem.201700848
(4-planar)x12(2-bent)x24___(Oh)	[Cu2]12[(C6H3)(OC2H4)2(OH)(CO2)2]24	0	7962.355128	10.1016/j.ica.2010.06.022
(4-planar)x12(2-bent)x24___(Oh)	[Cu2]12[(C6H3)(OC2H4)3(OH)(CO2)2]24	0	9019.608192	10.1039/C5TA02286J
(4-planar)x12(2-bent)x24___(Oh)	[Cu2]12[(C6H3)O(CH2)11CH3(CO2)2]24	0	9887.44068	10.1021/ja062491e
(4-planar)x12(2-bent)x24___(Oh)	[Cu2]12[(C6H3)O(CH2)4CH3(CO2)2]24	0	7531.0122	10.1021/acsami.8b02015
(4-planar)x12(2-bent)x24___(Oh)	[Cu2]12[(C6H3)(OC2H4OH)(CO2)2]24	0	6905.102064	10.1039/B811004B
(4-planar)x12(2-bent)x24___(Oh)	[Cu2]12[(C6H3)NHCOC(CH3)3(CO2)2]24	0	7842.98508	10.1021/acs.chemmater.8b01667
(4-planar)x12(2-bent)x24___(Oh)	[Cu2]12[(C6H3)CH2CS2(C6H5)(CO2)2]24	0	9454.163136	10.1021/jacs.6b01758
(4-planar)x12(2-bent)x24___(Oh)	[Cu2]12[C6H3CH2CS3C4H9(CO2)2]24	0	9743.961216	10.1021/jacs.6b01758
(4-planar)x12(2-bent)x24___(Oh)	[Cu2]12[C6H3C2Si(C3H7)3(CO2)2]24	0	9792.496416	10.1039/C0CC02771E
(4-planar)x12(2-bent)x24___(Oh)	[Cu2]12[(C6H3)(CH2)(C4N2H2O2CH3)(CO2)2]24	0	8778.826704	10.1039/C3QI00042G
(4-planar)x12(2-bent)x24___(Oh)	[Cu2]12[(C6H3)((C2H4O2)(C9H5O2))(CO2)2]24	0	10364.14051	10.1039/C7CC04799A
(4-planar)x12(2-bent)x24___(Oh)	[Cu2]12[(C6H3)(N2(C6H4CH3))(CO2)2]24	0	8299.107936	10.1002/anie.201310211

(4-planar)x12(2-bent)x24___(Oh)	[Cu2]12[((C6H3)C(CH3)3)(CO2)2]24	0	6810.391776	10.1038/nchem.803
(4-planar)x12(2-bent)x24___(Oh)	[Cu2]12[(C6H3SO3)(CO2)2]24	0	7361.196648	10.1038/nchem.803
(4-planar)x12(2-bent)x24___(Oh)	[Cu2]12[(C6H3)O(CH2)13CH3(CO2)2]24	0	10560.70596	Not in OntoMOPs KG
(4-planar)x12(2-bent)x24___(Oh)	[Cu2]12[C6H3CO2C2H4Si(CH3)3(CO2)2]24	0	8925.684624	Not in OntoMOPs KG
(4-planar)x12(2-bent)x24___(Oh)	[Cu2]12[(C6H3)(CO2)3]24	-24	6495.905664	Not in OntoMOPs KG
(4-planar)x12(2-bent)x24___(Oh)	[Cu2]12[C6H3CO2CH3(CO2)2]24	0	6856.726224	Not in OntoMOPs KG
(4-planar)x12(2-bent)x24___(Oh)	[Cu2]12[C6H3OCOCHCH2(CO2)2]24	0	7144.983024	Not in OntoMOPs KG
(4-planar)x12(2-bent)x24___(Oh)	[Cu2]12[C6H3NHCO2C(CH3)3(CO2)2]24	0	8226.972864	Not in OntoMOPs KG
(4-planar)x12(2-bent)x24___(Oh)	[Cu2]12[(C6H2O)(CH2CHCH2)(CO2)2]24	0	6809.37108	10.1021/ja803783c
(4-planar)x12(2-bent)x24___(Oh)	[Cu2]12[(C6H3)O(CH2)3CH3(CO2)2]24	0	7194.37956	10.1021/acsami.8b02015
(4-planar)x12(2-bent)x24___(Oh)	[Cu2]12[(C6H3)C6H2(OC16H33)3(CO2)2]24	0	24552.10793	10.1039/C8CC03705A
(4-planar)x12(2-bent)x24___(Oh)	[Cu2]12[C6H3N2C6H3(CH3)2(CO2)2]24	0	8635.740576	10.1002/anie.201310211
(4-planar)x12(2-bent)x24___(Oh)	[Cu2]12[(C6H3)(C2H4O2)(CONHC6H5)(CO2)2]24	0	9763.987848	10.1039/B811004B
(4-planar)x12(2-bent)x24___(Oh)	[Cu2]12[(C6H3)(OCH3)(CO2)2]24	0	6184.48164	Not in OntoMOPs KG
(4-planar)x12(2-bent)x24___(Oh)	[Fe2]12[(C6H4)(CO2)2]24	0	5279.037216	Not in OntoMOPs KG
(4-planar)x12(2-bent)x24___(Oh)	[Fe2]12[(C6H3OH)(CO2)2]24	0	5663.025	Not in OntoMOPs KG
(4-planar)x12(2-bent)x24___(Oh)	[Fe2]12[(C6H3(NO2))(CO2)2]24	0	6358.985664	Not in OntoMOPs KG
(4-planar)x12(2-bent)x24___(Oh)	[Fe2]12[(C6H3(CH3))(CO2)2]24	0	5615.669856	10.1021/jacs.9b05872
(4-planar)x12(2-bent)x24___(Oh)	[Fe2]12[(C6H3Br)(CO2)2]24	0	7172.545296	Not in OntoMOPs KG
(4-planar)x12(2-bent)x24___(Oh)	[Fe2]12[(C6H3)((C3H6O2)(C9H5O2))(CO2)2]24	0	10515.94915	Not in OntoMOPs KG
(4-planar)x12(2-bent)x24___(Oh)	[Fe2]12[(C6H3)((C6H12O2)(C9H5O2))(CO2)2]24	0	11525.84707	Not in OntoMOPs KG
(4-planar)x12(2-bent)x24___(Oh)	[Fe2]12[(C6H3)(OC2H4)3(OCH3)(CO2)2]24	0	9171.416832	Not in OntoMOPs KG
(4-planar)x12(2-bent)x24___(Oh)	[Fe2]12[(C6H3)(OCH2C2H)(CO2)2]24	0	6576.17124	Not in OntoMOPs KG
(4-planar)x12(2-bent)x24___(Oh)	[Fe2]12[(C6H3NH2)(CO2)2]24	0	5639.385936	Not in OntoMOPs KG
(4-planar)x12(2-bent)x24___(Oh)	[Fe2]12[(C6H3)(OCH2CH2CH3)(CO2)2]24	0	6672.92292	Not in OntoMOPs KG
(4-planar)x12(2-bent)x24___(Oh)	[Fe2]12[(C10H6)(CO2)2]24	0	6480.440256	Not in OntoMOPs KG
(4-planar)x12(2-bent)x24___(Oh)	[Fe2]12[(C6H3)2(CO2)2]24	-24	6311.081664	Not in OntoMOPs KG
(4-planar)x12(2-bent)x24___(Oh)	[Fe2]12[(C6H3)(OCH2CH3)(CO2)2]24	0	6336.29028	Not in OntoMOPs KG
(4-planar)x12(2-bent)x24___(Oh)	[Fe2]12[(C6H3)(OC2H4)2(OH)(CO2)2]24	0	7777.531128	Not in OntoMOPs KG
(4-planar)x12(2-bent)x24___(Oh)	[Fe2]12[(C6H3)(OC2H4)3(OH)(CO2)2]24	0	8834.784192	Not in OntoMOPs KG
(4-planar)x12(2-bent)x24___(Oh)	[Fe2]12[(C6H3)O(CH2)11CH3(CO2)2]24	0	9702.61668	Not in OntoMOPs KG
(4-planar)x12(2-bent)x24___(Oh)	[Fe2]12[(C6H3)O(CH2)4CH3(CO2)2]24	0	7346.1882	Not in OntoMOPs KG
(4-planar)x12(2-bent)x24___(Oh)	[Fe2]12[(C6H3)(OC2H4OH)(CO2)2]24	0	6720.278064	Not in OntoMOPs KG
(4-planar)x12(2-bent)x24___(Oh)	[Fe2]12[(C6H3)NHCOC(CH3)3(CO2)2]24	0	7658.16108	Not in OntoMOPs KG
(4-planar)x12(2-bent)x24___(Oh)	[Fe2]12[(C6H3)CH2CS2(C6H5)(CO2)2]24	0	9269.339136	Not in OntoMOPs KG
(4-planar)x12(2-bent)x24___(Oh)	[Fe2]12[C6H3CH2CS3C4H9(CO2)2]24	0	9559.137216	Not in OntoMOPs KG
(4-planar)x12(2-bent)x24___(Oh)	[Fe2]12[C6H3C2Si(C3H7)3(CO2)2]24	0	9607.672416	Not in OntoMOPs KG
(4-planar)x12(2-bent)x24___(Oh)	[Fe2]12[(C6H3)(CH2)(C4N2H2O2CH3)(CO2)2]24	0	8594.002704	Not in OntoMOPs KG
(4-planar)x12(2-bent)x24___(Oh)	[Fe2]12[(C6H3)((C2H4O2)(C9H5O2))(CO2)2]24	0	10179.31651	Not in OntoMOPs KG
(4-planar)x12(2-bent)x24___(Oh)	[Fe2]12[(C6H3)(N2(C6H4CH3))(CO2)2]24	0	8114.283936	Not in OntoMOPs KG
(4-planar)x12(2-bent)x24___(Oh)	[Fe2]12[((C6H3)C(CH3)3)(CO2)2]24	0	6625.567776	Not in OntoMOPs KG
(4-planar)x12(2-bent)x24___(Oh)	[Fe2]12[(C6H3SO3)(CO2)2]24	0	7176.372648	Not in OntoMOPs KG
(4-planar)x12(2-bent)x24___(Oh)	[Fe2]12[(C6H3)O(CH2)13CH3(CO2)2]24	0	10375.88196	Not in OntoMOPs KG
(4-planar)x12(2-bent)x24___(Oh)	[Fe2]12[C6H3CO2C2H4Si(CH3)3(CO2)2]24	0	8740.860624	Not in OntoMOPs KG
(4-planar)x12(2-bent)x24___(Oh)	[Fe2]12[(C6H3)(CO2)3]24	-24	6311.081664	Not in OntoMOPs KG
(4-planar)x12(2-bent)x24___(Oh)	[Fe2]12[C6H3CO2CH3(CO2)2]24	0	6671.902224	Not in OntoMOPs KG
(4-planar)x12(2-bent)x24___(Oh)	[Fe2]12[C6H3OCOCHCH2(CO2)2]24	0	6960.159024	Not in OntoMOPs KG
(4-planar)x12(2-bent)x24___(Oh)	[Fe2]12[C6H3NHCO2C(CH3)3(CO2)2]24	0	8042.148864	Not in OntoMOPs KG
(4-planar)x12(2-bent)x24___(Oh)	[Fe2]12[(C6H2O)(CH2CHCH2)(CO2)2]24	0	6624.54708	Not in OntoMOPs KG
(4-planar)x12(2-bent)x24___(Oh)	[Fe2]12[(C6H3)O(CH2)3CH3(CO2)2]24	0	7009.55556	Not in OntoMOPs KG

(4-planar)x12(2-bent)x24___(Oh)	[Fe2]12[(C6H3)C6H2(OC16H33)3(CO2)2]24	0	24367.28393	Not in OntoMOPs KG
(4-planar)x12(2-bent)x24___(Oh)	[Fe2]12[C6H3N2C6H3(CH3)2(CO2)2]24	0	8450.916576	Not in OntoMOPs KG
(4-planar)x12(2-bent)x24___(Oh)	[Fe2]12[(C6H3)(C2H4O2)(CONHC6H5)(CO2)2]24	0	9579.163848	Not in OntoMOPs KG
(4-planar)x12(2-bent)x24___(Oh)	[Fe2]12[(C6H3)(OCH3)(CO2)2]24	0	5999.65764	Not in OntoMOPs KG
(4-planar)x12(2-bent)x24___(Oh)	[Co2]12[(C6H4)(CO2)2]24	0	5353.154016	Not in OntoMOPs KG
(4-planar)x12(2-bent)x24___(Oh)	[Co2]12[(C6H3OH)(CO2)2]24	0	5737.1418	Not in OntoMOPs KG
(4-planar)x12(2-bent)x24___(Oh)	[Co2]12[(C6H3(NO2))(CO2)2]24	0	6433.102464	Not in OntoMOPs KG
(4-planar)x12(2-bent)x24___(Oh)	[Co2]12[(C6H3(CH3))(CO2)2]24	0	5689.786656	10.1021/jacs.9b05872
(4-planar)x12(2-bent)x24___(Oh)	[Co2]12[(C6H3Br)(CO2)2]24	0	7246.662096	Not in OntoMOPs KG
(4-planar)x12(2-bent)x24___(Oh)	[Co2]12[(C6H3)((C3H6O2)(C9H5O2))(CO2)2]24	0	10590.06595	Not in OntoMOPs KG
(4-planar)x12(2-bent)x24___(Oh)	[Co2]12[(C6H3)((C6H12O2)(C9H5O2))(CO2)2]24	0	11599.96387	Not in OntoMOPs KG
(4-planar)x12(2-bent)x24___(Oh)	[Co2]12[(C6H3)(OC2H4)3(OCH3)(CO2)2]24	0	9245.533632	Not in OntoMOPs KG
(4-planar)x12(2-bent)x24___(Oh)	[Co2]12[(C6H3)(OCH2C2H)(CO2)2]24	0	6650.28804	Not in OntoMOPs KG
(4-planar)x12(2-bent)x24___(Oh)	[Co2]12[(C6H3NH2)(CO2)2]24	0	5713.502736	Not in OntoMOPs KG
(4-planar)x12(2-bent)x24___(Oh)	[Co2]12[(C6H3)(OCH2CH2CH3)(CO2)2]24	0	6747.03972	Not in OntoMOPs KG
(4-planar)x12(2-bent)x24___(Oh)	[Co2]12[(C10H6)(CO2)2]24	0	6554.557056	Not in OntoMOPs KG
(4-planar)x12(2-bent)x24___(Oh)	[Co2]12[(C6H3)2(CO2)2]24	-24	6385.198464	Not in OntoMOPs KG
(4-planar)x12(2-bent)x24___(Oh)	[Co2]12[(C6H3)(OCH2CH3)(CO2)2]24	0	6410.40708	Not in OntoMOPs KG
(4-planar)x12(2-bent)x24___(Oh)	[Co2]12[(C6H3)(OC2H4)2(OH)(CO2)2]24	0	7851.647928	Not in OntoMOPs KG
(4-planar)x12(2-bent)x24___(Oh)	[Co2]12[(C6H3)(OC2H4)3(OH)(CO2)2]24	0	8908.900992	Not in OntoMOPs KG
(4-planar)x12(2-bent)x24___(Oh)	[Co2]12[(C6H3)O(CH2)11CH3(CO2)2]24	0	9776.73348	Not in OntoMOPs KG
(4-planar)x12(2-bent)x24___(Oh)	[Co2]12[(C6H3)O(CH2)4CH3(CO2)2]24	0	7420.305	Not in OntoMOPs KG
(4-planar)x12(2-bent)x24___(Oh)	[Co2]12[(C6H3)(OC2H4OH)(CO2)2]24	0	6794.394864	Not in OntoMOPs KG
(4-planar)x12(2-bent)x24___(Oh)	[Co2]12[(C6H3)NHCOC(CH3)3(CO2)2]24	0	7732.27788	Not in OntoMOPs KG
(4-planar)x12(2-bent)x24___(Oh)	[Co2]12[(C6H3)CH2CS2(C6H5)(CO2)2]24	0	9343.455936	Not in OntoMOPs KG
(4-planar)x12(2-bent)x24___(Oh)	[Co2]12[C6H3CH2CS3C4H9(CO2)2]24	0	9633.254016	Not in OntoMOPs KG
(4-planar)x12(2-bent)x24___(Oh)	[Co2]12[C6H3C2Si(C3H7)3(CO2)2]24	0	9681.789216	Not in OntoMOPs KG
(4-planar)x12(2-bent)x24___(Oh)	[Co2]12[(C6H3)(CH2)(C4N2H2O2CH3)(CO2)2]24	0	8668.119504	Not in OntoMOPs KG
(4-planar)x12(2-bent)x24___(Oh)	[Co2]12[(C6H3)((C2H4O2)(C9H5O2))(CO2)2]24	0	10253.43331	Not in OntoMOPs KG
(4-planar)x12(2-bent)x24___(Oh)	[Co2]12[(C6H3)(N2(C6H4CH3))(CO2)2]24	0	8188.400736	Not in OntoMOPs KG
(4-planar)x12(2-bent)x24___(Oh)	[Co2]12[((C6H3)C(CH3)3)(CO2)2]24	0	6699.684576	Not in OntoMOPs KG
(4-planar)x12(2-bent)x24___(Oh)	[Co2]12[(C6H3SO3)(CO2)2]24	0	7250.489448	Not in OntoMOPs KG
(4-planar)x12(2-bent)x24___(Oh)	[Co2]12[(C6H3)O(CH2)13CH3(CO2)2]24	0	10449.99876	Not in OntoMOPs KG
(4-planar)x12(2-bent)x24___(Oh)	[Co2]12[C6H3CO2C2H4Si(CH3)3(CO2)2]24	0	8814.977424	Not in OntoMOPs KG
(4-planar)x12(2-bent)x24___(Oh)	[Co2]12[(C6H3)(CO2)3]24	-24	6385.198464	Not in OntoMOPs KG
(4-planar)x12(2-bent)x24___(Oh)	[Co2]12[C6H3CO2CH3(CO2)2]24	0	6746.019024	Not in OntoMOPs KG
(4-planar)x12(2-bent)x24___(Oh)	[Co2]12[C6H3OCOCHCH2(CO2)2]24	0	7034.275824	Not in OntoMOPs KG
(4-planar)x12(2-bent)x24___(Oh)	[Co2]12[C6H3NHCO2C(CH3)3(CO2)2]24	0	8116.265664	Not in OntoMOPs KG
(4-planar)x12(2-bent)x24___(Oh)	[Co2]12[(C6H2O)(CH2CHCH2)(CO2)2]24	0	6698.66388	Not in OntoMOPs KG
(4-planar)x12(2-bent)x24___(Oh)	[Co2]12[(C6H3)O(CH2)3CH3(CO2)2]24	0	7083.67236	Not in OntoMOPs KG
(4-planar)x12(2-bent)x24___(Oh)	[Co2]12[(C6H3)C6H2(OC16H33)3(CO2)2]24	0	24441.40073	Not in OntoMOPs KG
(4-planar)x12(2-bent)x24___(Oh)	[Co2]12[C6H3N2C6H3(CH3)2(CO2)2]24	0	8525.033376	Not in OntoMOPs KG
(4-planar)x12(2-bent)x24___(Oh)	[Co2]12[(C6H3)(C2H4O2)(CONHC6H5)(CO2)2]24	0	9653.280648	Not in OntoMOPs KG
(4-planar)x12(2-bent)x24___(Oh)	[Co2]12[(C6H3)(OCH3)(CO2)2]24	0	6073.77444	Not in OntoMOPs KG
(4-planar)x12(2-bent)x24___(Oh)	[Cr2]12[(C6H4)(CO2)2]24	0	5186.663616	10.1021/acsami.7b09339
(4-planar)x12(2-bent)x24___(Oh)	[Cr2]12[(C6H3OH)(CO2)2]24	0	5570.6514	10.1021/acs.chemmater.7b03361
(4-planar)x12(2-bent)x24___(Oh)	[Cr2]12[(C6H3(NO2))(CO2)2]24	0	6266.612064	Not in OntoMOPs KG
(4-planar)x12(2-bent)x24___(Oh)	[Cr2]12[(C6H3(CH3))(CO2)2]24	0	5523.296256	Not in OntoMOPs KG
(4-planar)x12(2-bent)x24___(Oh)	[Cr2]12[(C6H3Br)(CO2)2]24	0	7080.171696	Not in OntoMOPs KG
(4-planar)x12(2-bent)x24___(Oh)	[Cr2]12[(C6H3)((C3H6O2)(C9H5O2))(CO2)2]24	0	10423.57555	Not in OntoMOPs KG

(4-planar)x12(2-bent)x24___(Oh)	[Cr2]12[(C6H3)((C6H12O2)(C9H5O2))(CO2)2]24	0	11433.47347	Not in OntoMOPs KG
(4-planar)x12(2-bent)x24___(Oh)	[Cr2]12[(C6H3)(OC2H4)3(OCH3)(CO2)2]24	0	9079.043232	Not in OntoMOPs KG
(4-planar)x12(2-bent)x24___(Oh)	[Cr2]12[(C6H3)(OCH2C2H)(CO2)2]24	0	6483.79764	Not in OntoMOPs KG
(4-planar)x12(2-bent)x24___(Oh)	[Cr2]12[(C6H3NH2)(CO2)2]24	0	5547.012336	Not in OntoMOPs KG
(4-planar)x12(2-bent)x24___(Oh)	[Cr2]12[(C6H3)(OCH2CH2CH3)(CO2)2]24	0	6580.54932	Not in OntoMOPs KG
(4-planar)x12(2-bent)x24___(Oh)	[Cr2]12[(C10H6)(CO2)2]24	0	6388.066656	Not in OntoMOPs KG
(4-planar)x12(2-bent)x24___(Oh)	[Cr2]12[(C6H3)2(CO2)2]24	-24	6218.708064	Not in OntoMOPs KG
(4-planar)x12(2-bent)x24___(Oh)	[Cr2]12[(C6H3)(OCH2CH3)(CO2)2]24	0	6243.91668	Not in OntoMOPs KG
(4-planar)x12(2-bent)x24___(Oh)	[Cr2]12[(C6H3)(OC2H4)2(OH)(CO2)2]24	0	7685.157528	Not in OntoMOPs KG
(4-planar)x12(2-bent)x24___(Oh)	[Cr2]12[(C6H3)(OC2H4)3(OH)(CO2)2]24	0	8742.410592	Not in OntoMOPs KG
(4-planar)x12(2-bent)x24___(Oh)	[Cr2]12[(C6H3)O(CH2)11CH3(CO2)2]24	0	9610.24308	Not in OntoMOPs KG
(4-planar)x12(2-bent)x24___(Oh)	[Cr2]12[(C6H3)O(CH2)4CH3(CO2)2]24	0	7253.8146	Not in OntoMOPs KG
(4-planar)x12(2-bent)x24___(Oh)	[Cr2]12[(C6H3)(OC2H4OH)(CO2)2]24	0	6627.904464	Not in OntoMOPs KG
(4-planar)x12(2-bent)x24___(Oh)	[Cr2]12[(C6H3)NHCOC(CH3)3(CO2)2]24	0	7565.78748	Not in OntoMOPs KG
(4-planar)x12(2-bent)x24___(Oh)	[Cr2]12[(C6H3)CH2CS2(C6H5)(CO2)2]24	0	9176.965536	Not in OntoMOPs KG
(4-planar)x12(2-bent)x24___(Oh)	[Cr2]12[C6H3CH2CS3C4H9(CO2)2]24	0	9466.763616	Not in OntoMOPs KG
(4-planar)x12(2-bent)x24___(Oh)	[Cr2]12[C6H3C2Si(C3H7)3(CO2)2]24	0	9515.298816	10.1021/acsami.7b09339
(4-planar)x12(2-bent)x24___(Oh)	[Cr2]12[(C6H3)(CH2)(C4N2H2O2CH3)(CO2)2]24	0	8501.629104	Not in OntoMOPs KG
(4-planar)x12(2-bent)x24___(Oh)	[Cr2]12[(C6H3)((C2H4O2)(C9H5O2))(CO2)2]24	0	10086.94291	Not in OntoMOPs KG
(4-planar)x12(2-bent)x24___(Oh)	[Cr2]12[(C6H3)(N2(C6H4CH3))(CO2)2]24	0	8021.910336	Not in OntoMOPs KG
(4-planar)x12(2-bent)x24___(Oh)	[Cr2]12[((C6H3)C(CH3)3)(CO2)2]24	0	6533.194176	10.1021/acsami.7b09339
(4-planar)x12(2-bent)x24___(Oh)	[Cr2]12[(C6H3SO3)(CO2)2]24	0	7083.999048	Not in OntoMOPs KG
(4-planar)x12(2-bent)x24___(Oh)	[Cr2]12[(C6H3)O(CH2)13CH3(CO2)2]24	0	10283.50836	Not in OntoMOPs KG
(4-planar)x12(2-bent)x24___(Oh)	[Cr2]12[C6H3CO2C2H4Si(CH3)3(CO2)2]24	0	8648.487024	Not in OntoMOPs KG
(4-planar)x12(2-bent)x24___(Oh)	[Cr2]12[(C6H3)(CO2)3]24	-24	6218.708064	Not in OntoMOPs KG
(4-planar)x12(2-bent)x24___(Oh)	[Cr2]12[C6H3CO2CH3(CO2)2]24	0	6579.528624	Not in OntoMOPs KG
(4-planar)x12(2-bent)x24___(Oh)	[Cr2]12[C6H3OCOCHCH2(CO2)2]24	0	6867.785424	Not in OntoMOPs KG
(4-planar)x12(2-bent)x24___(Oh)	[Cr2]12[C6H3NHCO2C(CH3)3(CO2)2]24	0	7949.775264	Not in OntoMOPs KG
(4-planar)x12(2-bent)x24___(Oh)	[Cr2]12[(C6H2O)(CH2CHCH2)(CO2)2]24	0	6532.17348	Not in OntoMOPs KG
(4-planar)x12(2-bent)x24___(Oh)	[Cr2]12[(C6H3)O(CH2)3CH3(CO2)2]24	0	6917.18196	Not in OntoMOPs KG
(4-planar)x12(2-bent)x24___(Oh)	[Cr2]12[(C6H3)C6H2(OC16H33)3(CO2)2]24	0	24274.91033	Not in OntoMOPs KG
(4-planar)x12(2-bent)x24___(Oh)	[Cr2]12[C6H3N2C6H3(CH3)2(CO2)2]24	0	8358.542976	Not in OntoMOPs KG
(4-planar)x12(2-bent)x24___(Oh)	[Cr2]12[(C6H3)(C2H4O2)(CONHC6H5)(CO2)2]24	0	9486.790248	Not in OntoMOPs KG
(4-planar)x12(2-bent)x24___(Oh)	[Cr2]12[(C6H3)(OCH3)(CO2)2]24	0	5907.28404	Not in OntoMOPs KG
(4-planar)x12(2-bent)x24___(Oh)	[CuPd]12[(C6H4)(CO2)2]24	0	5978.349216	Not in OntoMOPs KG
(4-planar)x12(2-bent)x24___(Oh)	[CuPd]12[(C6H3OH)(CO2)2]24	0	6362.337	Not in OntoMOPs KG
(4-planar)x12(2-bent)x24___(Oh)	[CuPd]12[(C6H3(NO2))(CO2)2]24	0	7058.297664	Not in OntoMOPs KG
(4-planar)x12(2-bent)x24___(Oh)	[CuPd]12[(C6H3(CH3))(CO2)2]24	0	6314.981856	Not in OntoMOPs KG
(4-planar)x12(2-bent)x24___(Oh)	[CuPd]12[(C6H3Br)(CO2)2]24	0	7871.857296	Not in OntoMOPs KG
(4-planar)x12(2-bent)x24___(Oh)	[CuPd]12[(C6H3)((C3H6O2)(C9H5O2))(CO2)2]24	0	11215.26115	Not in OntoMOPs KG
(4-planar)x12(2-bent)x24___(Oh)	[CuPd]12[(C6H3)((C6H12O2)(C9H5O2))(CO2)2]24	0	12225.15907	Not in OntoMOPs KG
(4-planar)x12(2-bent)x24___(Oh)	[CuPd]12[(C6H3)(OC2H4)3(OCH3)(CO2)2]24	0	9870.728832	Not in OntoMOPs KG
(4-planar)x12(2-bent)x24___(Oh)	[CuPd]12[(C6H3)(OCH2C2H)(CO2)2]24	0	7275.48324	Not in OntoMOPs KG
(4-planar)x12(2-bent)x24___(Oh)	[CuPd]12[(C6H3NH2)(CO2)2]24	0	6338.697936	Not in OntoMOPs KG
(4-planar)x12(2-bent)x24___(Oh)	[CuPd]12[(C6H3)(OCH2CH2CH3)(CO2)2]24	0	7372.23492	Not in OntoMOPs KG
(4-planar)x12(2-bent)x24___(Oh)	[CuPd]12[(C10H6)(CO2)2]24	0	7179.752256	Not in OntoMOPs KG
(4-planar)x12(2-bent)x24___(Oh)	[CuPd]12[(C6H3)2(CO2)2]24	-24	7010.393664	Not in OntoMOPs KG
(4-planar)x12(2-bent)x24___(Oh)	[CuPd]12[(C6H3)(OCH2CH3)(CO2)2]24	0	7035.60228	Not in OntoMOPs KG
(4-planar)x12(2-bent)x24___(Oh)	[CuPd]12[(C6H3)(OC2H4)2(OH)(CO2)2]24	0	8476.843128	Not in OntoMOPs KG
(4-planar)x12(2-bent)x24___(Oh)	[CuPd]12[(C6H3)(OC2H4)3(OH)(CO2)2]24	0	9534.096192	Not in OntoMOPs KG

(4-planar)x12(2-bent)x24___(Oh)	[CuPd]12[(C6H3)O(CH2)11CH3(CO2)2]24	0	10401.92868	Not in OntoMOPs KG
(4-planar)x12(2-bent)x24___(Oh)	[CuPd]12[(C6H3)O(CH2)4CH3(CO2)2]24	0	8045.5002	Not in OntoMOPs KG
(4-planar)x12(2-bent)x24___(Oh)	[CuPd]12[(C6H3)(OC2H4OH)(CO2)2]24	0	7419.590064	Not in OntoMOPs KG
(4-planar)x12(2-bent)x24___(Oh)	[CuPd]12[(C6H3)NHCOC(CH3)3(CO2)2]24	0	8357.47308	Not in OntoMOPs KG
(4-planar)x12(2-bent)x24___(Oh)	[CuPd]12[(C6H3)CH2CS2(C6H5)(CO2)2]24	0	9968.651136	Not in OntoMOPs KG
(4-planar)x12(2-bent)x24___(Oh)	[CuPd]12[C6H3CH2CS3C4H9(CO2)2]24	0	10258.44922	Not in OntoMOPs KG
(4-planar)x12(2-bent)x24___(Oh)	[CuPd]12[C6H3C2Si(C3H7)3(CO2)2]24	0	10306.98442	Not in OntoMOPs KG
(4-planar)x12(2-bent)x24___(Oh)	[CuPd]12[(C6H3)(CH2)(C4N2H2O2CH3)(CO2)2]24	0	9293.314704	Not in OntoMOPs KG
(4-planar)x12(2-bent)x24___(Oh)	[CuPd]12[(C6H3)((C2H4O2)(C9H5O2))(CO2)2]24	0	10878.62851	Not in OntoMOPs KG
(4-planar)x12(2-bent)x24___(Oh)	[CuPd]12[(C6H3)(N2(C6H4CH3))(CO2)2]24	0	8813.595936	Not in OntoMOPs KG
(4-planar)x12(2-bent)x24___(Oh)	[CuPd]12[((C6H3)C(CH3)3)(CO2)2]24	0	7324.879776	10.1039/c5cc08336b
(4-planar)x12(2-bent)x24___(Oh)	[CuPd]12[(C6H3SO3)(CO2)2]24	0	7875.684648	Not in OntoMOPs KG
(4-planar)x12(2-bent)x24___(Oh)	[CuPd]12[(C6H3)O(CH2)13CH3(CO2)2]24	0	11075.19396	Not in OntoMOPs KG
(4-planar)x12(2-bent)x24___(Oh)	[CuPd]12[C6H3CO2C2H4Si(CH3)3(CO2)2]24	0	9440.172624	Not in OntoMOPs KG
(4-planar)x12(2-bent)x24___(Oh)	[CuPd]12[(C6H3)(CO2)3]24	-24	7010.393664	Not in OntoMOPs KG
(4-planar)x12(2-bent)x24___(Oh)	[CuPd]12[C6H3CO2CH3(CO2)2]24	0	7371.214224	Not in OntoMOPs KG
(4-planar)x12(2-bent)x24___(Oh)	[CuPd]12[C6H3OCOCHCH2(CO2)2]24	0	7659.471024	Not in OntoMOPs KG
(4-planar)x12(2-bent)x24___(Oh)	[CuPd]12[C6H3NHCO2C(CH3)3(CO2)2]24	0	8741.460864	Not in OntoMOPs KG
(4-planar)x12(2-bent)x24___(Oh)	[CuPd]12[(C6H2O)(CH2CHCH2)(CO2)2]24	0	7323.85908	Not in OntoMOPs KG
(4-planar)x12(2-bent)x24___(Oh)	[CuPd]12[(C6H3)O(CH2)3CH3(CO2)2]24	0	7708.86756	Not in OntoMOPs KG
(4-planar)x12(2-bent)x24___(Oh)	[CuPd]12[(C6H3)C6H2(OC16H33)3(CO2)2]24	0	25066.59593	Not in OntoMOPs KG
(4-planar)x12(2-bent)x24___(Oh)	[CuPd]12[C6H3N2C6H3(CH3)2(CO2)2]24	0	9150.228576	Not in OntoMOPs KG
(4-planar)x12(2-bent)x24___(Oh)	[CuPd]12[(C6H3)(C2H4O2)(CONHC6H5)(CO2)2]24	0	10278.47585	Not in OntoMOPs KG
(4-planar)x12(2-bent)x24___(Oh)	[CuPd]12[(C6H3)(OCH3)(CO2)2]24	0	6698.96964	Not in OntoMOPs KG
(4-planar)x12(2-bent)x24___(Oh)	[CuNi]12[(C6H4)(CO2)2]24	0	5405.630016	Not in OntoMOPs KG
(4-planar)x12(2-bent)x24___(Oh)	[CuNi]12[(C6H3OH)(CO2)2]24	0	5789.6178	Not in OntoMOPs KG
(4-planar)x12(2-bent)x24___(Oh)	[CuNi]12[(C6H3(NO2))(CO2)2]24	0	6485.578464	Not in OntoMOPs KG
(4-planar)x12(2-bent)x24___(Oh)	[CuNi]12[(C6H3(CH3))(CO2)2]24	0	5742.262656	Not in OntoMOPs KG
(4-planar)x12(2-bent)x24___(Oh)	[CuNi]12[(C6H3Br)(CO2)2]24	0	7299.138096	Not in OntoMOPs KG
(4-planar)x12(2-bent)x24___(Oh)	[CuNi]12[(C6H3)((C3H6O2)(C9H5O2))(CO2)2]24	0	10642.54195	Not in OntoMOPs KG
(4-planar)x12(2-bent)x24___(Oh)	[CuNi]12[(C6H3)((C6H12O2)(C9H5O2))(CO2)2]24	0	11652.43987	Not in OntoMOPs KG
(4-planar)x12(2-bent)x24___(Oh)	[CuNi]12[(C6H3)(OC2H4)3(OCH3)(CO2)2]24	0	9298.009632	Not in OntoMOPs KG
(4-planar)x12(2-bent)x24___(Oh)	[CuNi]12[(C6H3)(OCH2C2H)(CO2)2]24	0	6702.76404	Not in OntoMOPs KG
(4-planar)x12(2-bent)x24___(Oh)	[CuNi]12[(C6H3NH2)(CO2)2]24	0	5765.978736	Not in OntoMOPs KG
(4-planar)x12(2-bent)x24___(Oh)	[CuNi]12[(C6H3)(OCH2CH2CH3)(CO2)2]24	0	6799.51572	Not in OntoMOPs KG
(4-planar)x12(2-bent)x24___(Oh)	[CuNi]12[(C10H6)(CO2)2]24	0	6607.033056	Not in OntoMOPs KG
(4-planar)x12(2-bent)x24___(Oh)	[CuNi]12[(C6H3)2(CO2)2]24	-24	6437.674464	Not in OntoMOPs KG
(4-planar)x12(2-bent)x24___(Oh)	[CuNi]12[(C6H3)(OCH2CH3)(CO2)2]24	0	6462.88308	Not in OntoMOPs KG
(4-planar)x12(2-bent)x24___(Oh)	[CuNi]12[(C6H3)(OC2H4)2(OH)(CO2)2]24	0	7904.123928	Not in OntoMOPs KG
(4-planar)x12(2-bent)x24___(Oh)	[CuNi]12[(C6H3)(OC2H4)3(OH)(CO2)2]24	0	8961.376992	Not in OntoMOPs KG
(4-planar)x12(2-bent)x24___(Oh)	[CuNi]12[(C6H3)O(CH2)11CH3(CO2)2]24	0	9829.20948	Not in OntoMOPs KG
(4-planar)x12(2-bent)x24___(Oh)	[CuNi]12[(C6H3)O(CH2)4CH3(CO2)2]24	0	7472.781	Not in OntoMOPs KG
(4-planar)x12(2-bent)x24___(Oh)	[CuNi]12[(C6H3)(OC2H4OH)(CO2)2]24	0	6846.870864	Not in OntoMOPs KG
(4-planar)x12(2-bent)x24___(Oh)	[CuNi]12[(C6H3)NHCOC(CH3)3(CO2)2]24	0	7784.75388	Not in OntoMOPs KG
(4-planar)x12(2-bent)x24___(Oh)	[CuNi]12[(C6H3)CH2CS2(C6H5)(CO2)2]24	0	9395.931936	Not in OntoMOPs KG
(4-planar)x12(2-bent)x24___(Oh)	[CuNi]12[C6H3CH2CS3C4H9(CO2)2]24	0	9685.730016	Not in OntoMOPs KG
(4-planar)x12(2-bent)x24___(Oh)	[CuNi]12[C6H3C2Si(C3H7)3(CO2)2]24	0	9734.265216	Not in OntoMOPs KG
(4-planar)x12(2-bent)x24___(Oh)	[CuNi]12[(C6H3)(CH2)(C4N2H2O2CH3)(CO2)2]24	0	8720.595504	Not in OntoMOPs KG
(4-planar)x12(2-bent)x24___(Oh)	[CuNi]12[(C6H3)((C2H4O2)(C9H5O2))(CO2)2]24	0	10305.90931	Not in OntoMOPs KG
(4-planar)x12(2-bent)x24___(Oh)	[CuNi]12[(C6H3)(N2(C6H4CH3))(CO2)2]24	0	8240.876736	Not in OntoMOPs KG

(4-planar)x12(2-bent)x24___(Oh)	[CuNi]12[((C6H3)C(CH3)3)(CO2)2]24	0	6752.160576	10.1039/C5CC08336B
(4-planar)x12(2-bent)x24___(Oh)	[CuNi]12[(C6H3SO3)(CO2)2]24	0	7302.965448	Not in OntoMOPs KG
(4-planar)x12(2-bent)x24___(Oh)	[CuNi]12[(C6H3)O(CH2)13CH3(CO2)2]24	0	10502.47476	Not in OntoMOPs KG
(4-planar)x12(2-bent)x24___(Oh)	[CuNi]12[C6H3CO2C2H4Si(CH3)3(CO2)2]24	0	8867.453424	Not in OntoMOPs KG
(4-planar)x12(2-bent)x24___(Oh)	[CuNi]12[(C6H3)(CO2)3]24	-24	6437.674464	Not in OntoMOPs KG
(4-planar)x12(2-bent)x24___(Oh)	[CuNi]12[C6H3CO2CH3(CO2)2]24	0	6798.495024	Not in OntoMOPs KG
(4-planar)x12(2-bent)x24___(Oh)	[CuNi]12[C6H3OCOCHCH2(CO2)2]24	0	7086.751824	Not in OntoMOPs KG
(4-planar)x12(2-bent)x24___(Oh)	[CuNi]12[C6H3NHCO2C(CH3)3(CO2)2]24	0	8168.741664	Not in OntoMOPs KG
(4-planar)x12(2-bent)x24___(Oh)	[CuNi]12[(C6H2O)(CH2CHCH2)(CO2)2]24	0	6751.13988	Not in OntoMOPs KG
(4-planar)x12(2-bent)x24___(Oh)	[CuNi]12[(C6H3)O(CH2)3CH3(CO2)2]24	0	7136.14836	Not in OntoMOPs KG
(4-planar)x12(2-bent)x24___(Oh)	[CuNi]12[(C6H3)C6H2(OC16H33)3(CO2)2]24	0	24493.87673	Not in OntoMOPs KG
(4-planar)x12(2-bent)x24___(Oh)	[CuNi]12[C6H3N2C6H3(CH3)2(CO2)2]24	0	8577.509376	Not in OntoMOPs KG
(4-planar)x12(2-bent)x24___(Oh)	[CuNi]12[(C6H3)(C2H4O2)(CONHC6H5)(CO2)2]24	0	9705.756648	Not in OntoMOPs KG
(4-planar)x12(2-bent)x24___(Oh)	[CuNi]12[(C6H3)(OCH3)(CO2)2]24	0	6126.25044	Not in OntoMOPs KG
(4-planar)x12(2-bent)x24___(Oh)	[Mo2]12[(C6H4)(CO2)2]24	0	6241.317216	10.1021/ja1080794
(4-planar)x12(2-bent)x24___(Oh)	[Mo2]12[(C6H3OH)(CO2)2]24	0	6625.305	10.1021/ja1080794
(4-planar)x12(2-bent)x24___(Oh)	[Mo2]12[(C6H3(NO2))(CO2)2]24	0	7321.265664	Not in OntoMOPs KG
(4-planar)x12(2-bent)x24___(Oh)	[Mo2]12[(C6H3(CH3))(CO2)2]24	0	6577.949856	Not in OntoMOPs KG
(4-planar)x12(2-bent)x24___(Oh)	[Mo2]12[(C6H3Br)(CO2)2]24	0	8134.825296	Not in OntoMOPs KG
(4-planar)x12(2-bent)x24___(Oh)	[Mo2]12[(C6H3)((C3H6O2)(C9H5O2))(CO2)2]24	0	11478.22915	Not in OntoMOPs KG
(4-planar)x12(2-bent)x24___(Oh)	[Mo2]12[(C6H3)((C6H12O2)(C9H5O2))(CO2)2]24	0	12488.12707	Not in OntoMOPs KG
(4-planar)x12(2-bent)x24___(Oh)	[Mo2]12[(C6H3)(OC2H4)3(OCH3)(CO2)2]24	0	10133.69683	Not in OntoMOPs KG
(4-planar)x12(2-bent)x24___(Oh)	[Mo2]12[(C6H3)(OCH2C2H)(CO2)2]24	0	7538.45124	Not in OntoMOPs KG
(4-planar)x12(2-bent)x24___(Oh)	[Mo2]12[(C6H3NH2)(CO2)2]24	0	6601.665936	Not in OntoMOPs KG
(4-planar)x12(2-bent)x24___(Oh)	[Mo2]12[(C6H3)(OCH2CH2CH3)(CO2)2]24	0	7635.20292	Not in OntoMOPs KG
(4-planar)x12(2-bent)x24___(Oh)	[Mo2]12[(C10H6)(CO2)2]24	0	7442.720256	Not in OntoMOPs KG
(4-planar)x12(2-bent)x24___(Oh)	[Mo2]12[(C6H3)2(CO2)2]24	-24	7273.361664	Not in OntoMOPs KG
(4-planar)x12(2-bent)x24___(Oh)	[Mo2]12[(C6H3)(OCH2CH3)(CO2)2]24	0	7298.57028	Not in OntoMOPs KG
(4-planar)x12(2-bent)x24___(Oh)	[Mo2]12[(C6H3)(OC2H4)2(OH)(CO2)2]24	0	8739.811128	Not in OntoMOPs KG
(4-planar)x12(2-bent)x24___(Oh)	[Mo2]12[(C6H3)(OC2H4)3(OH)(CO2)2]24	0	9797.064192	Not in OntoMOPs KG
(4-planar)x12(2-bent)x24___(Oh)	[Mo2]12[(C6H3)O(CH2)11CH3(CO2)2]24	0	10664.89668	Not in OntoMOPs KG
(4-planar)x12(2-bent)x24___(Oh)	[Mo2]12[(C6H3)O(CH2)4CH3(CO2)2]24	0	8308.4682	Not in OntoMOPs KG
(4-planar)x12(2-bent)x24___(Oh)	[Mo2]12[(C6H3)(OC2H4OH)(CO2)2]24	0	7682.558064	Not in OntoMOPs KG
(4-planar)x12(2-bent)x24___(Oh)	[Mo2]12[(C6H3)NHCOC(CH3)3(CO2)2]24	0	8620.44108	Not in OntoMOPs KG
(4-planar)x12(2-bent)x24___(Oh)	[Mo2]12[(C6H3)CH2CS2(C6H5)(CO2)2]24	0	10231.61914	Not in OntoMOPs KG
(4-planar)x12(2-bent)x24___(Oh)	[Mo2]12[C6H3CH2CS3C4H9(CO2)2]24	0	10521.41722	Not in OntoMOPs KG
(4-planar)x12(2-bent)x24___(Oh)	[Mo2]12[C6H3C2Si(C3H7)3(CO2)2]24	0	10569.95242	Not in OntoMOPs KG
(4-planar)x12(2-bent)x24___(Oh)	[Mo2]12[(C6H3)(CH2)(C4N2H2O2CH3)(CO2)2]24	0	9556.282704	Not in OntoMOPs KG
(4-planar)x12(2-bent)x24___(Oh)	[Mo2]12[(C6H3)((C2H4O2)(C9H5O2))(CO2)2]24	0	11141.59651	Not in OntoMOPs KG
(4-planar)x12(2-bent)x24___(Oh)	[Mo2]12[(C6H3)(N2(C6H4CH3))(CO2)2]24	0	9076.563936	Not in OntoMOPs KG
(4-planar)x12(2-bent)x24___(Oh)	[Mo2]12[((C6H3)C(CH3)3)(CO2)2]24	0	7587.847776	10.1021/ja1080794
(4-planar)x12(2-bent)x24___(Oh)	[Mo2]12[(C6H3SO3)(CO2)2]24	0	8138.652648	Not in OntoMOPs KG
(4-planar)x12(2-bent)x24___(Oh)	[Mo2]12[(C6H3)O(CH2)13CH3(CO2)2]24	0	11338.16196	Not in OntoMOPs KG
(4-planar)x12(2-bent)x24___(Oh)	[Mo2]12[C6H3CO2C2H4Si(CH3)3(CO2)2]24	0	9703.140624	Not in OntoMOPs KG
(4-planar)x12(2-bent)x24___(Oh)	[Mo2]12[(C6H3)(CO2)3]24	-24	7273.361664	Not in OntoMOPs KG
(4-planar)x12(2-bent)x24___(Oh)	[Mo2]12[C6H3CO2CH3(CO2)2]24	0	7634.182224	Not in OntoMOPs KG
(4-planar)x12(2-bent)x24___(Oh)	[Mo2]12[C6H3OCOCHCH2(CO2)2]24	0	7922.439024	Not in OntoMOPs KG
(4-planar)x12(2-bent)x24___(Oh)	[Mo2]12[C6H3NHCO2C(CH3)3(CO2)2]24	0	9004.428864	Not in OntoMOPs KG
(4-planar)x12(2-bent)x24___(Oh)	[Mo2]12[(C6H2O)(CH2CHCH2)(CO2)2]24	0	7586.82708	Not in OntoMOPs KG
(4-planar)x12(2-bent)x24___(Oh)	[Mo2]12[(C6H3)O(CH2)3CH3(CO2)2]24	0	7971.83556	Not in OntoMOPs KG

(4-planar)x12(2-bent)x24___(Oh)	[Mo2]12[(C6H3)C6H2(OC16H33)3(CO2)2]24	0	25329.56393	Not in OntoMOPs KG
(4-planar)x12(2-bent)x24___(Oh)	[Mo2]12[C6H3N2C6H3(CH3)2(CO2)2]24	0	9413.196576	Not in OntoMOPs KG
(4-planar)x12(2-bent)x24___(Oh)	[Mo2]12[(C6H3)(C2H4O2)(CONHC6H5)(CO2)2]24	0	10541.44385	Not in OntoMOPs KG
(4-planar)x12(2-bent)x24___(Oh)	[Mo2]12[(C6H3)(OCH3)(CO2)2]24	0	6961.93764	Not in OntoMOPs KG
(4-planar)x6(2-bent)x12___(Oh)	[Rh2]6[(C6H3)2NH(CO2)2]12	0	4273.377288	Not in OntoMOPs KG
(4-planar)x6(2-bent)x12___(Oh)	[Rh2]6[(C6H3)2NC3H7(CO2)2]12	0	4778.326248	Not in OntoMOPs KG
(4-planar)x6(2-bent)x12___(Oh)	[Rh2]6[S(C6H3)2(CO2)2]12	0	4477.982928	Not in OntoMOPs KG
(4-planar)x6(2-bent)x12___(Oh)	[Rh2]6[(C4H2S)3(CO2)2]12	0	5247.542928	Not in OntoMOPs KG
(4-planar)x6(2-bent)x12___(Oh)	[Rh2]6[(C10H4)(CO)4N2)(CHCH)2(CH3)2(CH3)2(CO2)2]12	0	6807.938736	Not in OntoMOPs KG
(4-planar)x6(2-bent)x12___(Oh)	[Rh2]6[(C10H4)(CO)4N2)(CHCH)2(CH3)2(CH2CH3)2(CO2)2]12	0	7144.571376	Not in OntoMOPs KG
(4-planar)x6(2-bent)x12___(Oh)	[Rh2]6[(C10H4)(CO)4N2)(CHCH2)2(C6H5)2(CO2)2]12	0	7960.965936	Not in OntoMOPs KG
(4-planar)x6(2-bent)x12___(Oh)	[Rh2]6[(C10H4)(CO)4N2)(CHCH3)2(CO2)2]12	0	6134.673456	Not in OntoMOPs KG
(4-planar)x6(2-bent)x12___(Oh)	[Rh2]6[(C12H6NH)(C6H4)2(CO2)2]12	0	6075.717768	Not in OntoMOPs KG
(4-planar)x6(2-bent)x12___(Oh)	[Ru2]6[(C6H3)2NH(CO2)2]12	0	4251.351288	10.1016/j.ica.2014.09.010
(4-planar)x6(2-bent)x12___(Oh)	[Ru2]6[(C6H3)2NC3H7(CO2)2]12	0	4756.300248	Not in OntoMOPs KG
(4-planar)x6(2-bent)x12___(Oh)	[Ru2]6[S(C6H3)2(CO2)2]12	0	4455.956928	Not in OntoMOPs KG
(4-planar)x6(2-bent)x12___(Oh)	[Ru2]6[(C4H2S)3(CO2)2]12	0	5225.516928	Not in OntoMOPs KG
(4-planar)x6(2-bent)x12___(Oh)	[Ru2]6[(C10H4)(CO)4N2)(CHCH)2(CH3)2(CH3)2(CO2)2]12	0	6785.912736	Not in OntoMOPs KG
(4-planar)x6(2-bent)x12___(Oh)	[Ru2]6[(C10H4)(CO)4N2)(CHCH)2(CH3)2(CH2CH3)2(CO2)2]12	0	7122.545376	Not in OntoMOPs KG
(4-planar)x6(2-bent)x12___(Oh)	[Ru2]6[(C10H4)(CO)4N2)(CHCH2)2(C6H5)2(CO2)2]12	0	7938.939936	Not in OntoMOPs KG
(4-planar)x6(2-bent)x12___(Oh)	[Ru2]6[(C10H4)(CO)4N2)(CHCH3)2(CO2)2]12	0	6112.647456	Not in OntoMOPs KG
(4-planar)x6(2-bent)x12___(Oh)	[Ru2]6[(C12H6NH)(C6H4)2(CO2)2]12	0	6053.691768	Not in OntoMOPs KG
(4-planar)x6(2-bent)x12___(Oh)	[Ni2]6[(C6H3)2NH(CO2)2]12	0	3742.832088	Not in OntoMOPs KG
(4-planar)x6(2-bent)x12___(Oh)	[Ni2]6[(C6H3)2NC3H7(CO2)2]12	0	4247.781048	10.1021/acs.inorgchem.8b01130
(4-planar)x6(2-bent)x12___(Oh)	[Ni2]6[S(C6H3)2(CO2)2]12	0	3947.437728	Not in OntoMOPs KG
(4-planar)x6(2-bent)x12___(Oh)	[Ni2]6[(C4H2S)3(CO2)2]12	0	4716.997728	Not in OntoMOPs KG
(4-planar)x6(2-bent)x12___(Oh)	[Ni2]6[(C10H4)(CO)4N2)(CHCH)2(CH3)2(CH3)2(CO2)2]12	0	6277.393536	Not in OntoMOPs KG
(4-planar)x6(2-bent)x12___(Oh)	[Ni2]6[(C10H4)(CO)4N2)(CHCH)2(CH3)2(CH2CH3)2(CO2)2]12	0	6614.026176	Not in OntoMOPs KG
(4-planar)x6(2-bent)x12___(Oh)	[Ni2]6[(C10H4)(CO)4N2)(CHCH2)2(C6H5)2(CO2)2]12	0	7430.420736	Not in OntoMOPs KG
(4-planar)x6(2-bent)x12___(Oh)	[Ni2]6[(C10H4)(CO)4N2)(CHCH3)2(CO2)2]12	0	5604.128256	Not in OntoMOPs KG
(4-planar)x6(2-bent)x12___(Oh)	[Ni2]6[(C12H6NH)(C6H4)2(CO2)2]12	0	5545.172568	Not in OntoMOPs KG
(4-planar)x6(2-bent)x12___(Oh)	[Cu2]6[(C6H3)2NH(CO2)2]12	0	3801.063288	10.1021/jacs.8b05780
(4-planar)x6(2-bent)x12___(Oh)	[Cu2]6[(C6H3)2NC3H7(CO2)2]12	0	4306.012248	Not in OntoMOPs KG
(4-planar)x6(2-bent)x12___(Oh)	[Cu2]6[S(C6H3)2(CO2)2]12	0	4005.668928	10.1016/j.inoche.2016.05.009
(4-planar)x6(2-bent)x12___(Oh)	[Cu2]6[(C4H2S)3(CO2)2]12	0	4775.228928	10.1021/ja052055c
(4-planar)x6(2-bent)x12___(Oh)	[Cu2]6[(C10H4)(CO)4N2)(CHCH)2(CH3)2(CH3)2(CO2)2]12	0	6335.624736	10.1002/anie.201811037
(4-planar)x6(2-bent)x12___(Oh)	[Cu2]6[(C10H4)(CO)4N2)(CHCH)2(CH3)2(CH2CH3)2(CO2)2]12	0	6672.257376	10.1002/anie.201811037
(4-planar)x6(2-bent)x12___(Oh)	[Cu2]6[(C10H4)(CO)4N2)(CHCH2)2(C6H5)2(CO2)2]12	0	7488.651936	10.1002/anie.201811037
(4-planar)x6(2-bent)x12___(Oh)	[Cu2]6[(C10H4)(CO)4N2)(CHCH3)2(CO2)2]12	0	5662.359456	10.1002/anie.201811037
(4-planar)x6(2-bent)x12___(Oh)	[Cu2]6[(C12H6NH)(C6H4)2(CO2)2]12	0	5603.403768	Not in OntoMOPs KG
(4-planar)x6(2-bent)x12___(Oh)	[Fe2]6[(C6H3)2NH(CO2)2]12	0	3708.651288	Not in OntoMOPs KG
(4-planar)x6(2-bent)x12___(Oh)	[Fe2]6[(C6H3)2NC3H7(CO2)2]12	0	4213.600248	Not in OntoMOPs KG
(4-planar)x6(2-bent)x12___(Oh)	[Fe2]6[S(C6H3)2(CO2)2]12	0	3913.256928	Not in OntoMOPs KG
(4-planar)x6(2-bent)x12___(Oh)	[Fe2]6[(C4H2S)3(CO2)2]12	0	4682.816928	Not in OntoMOPs KG
(4-planar)x6(2-bent)x12___(Oh)	[Fe2]6[(C10H4)(CO)4N2)(CHCH)2(CH3)2(CH3)2(CO2)2]12	0	6243.212736	Not in OntoMOPs KG
(4-planar)x6(2-bent)x12___(Oh)	[Fe2]6[(C10H4)(CO)4N2)(CHCH)2(CH3)2(CH2CH3)2(CO2)2]12	0	6579.845376	Not in OntoMOPs KG
(4-planar)x6(2-bent)x12___(Oh)	[Fe2]6[(C10H4)(CO)4N2)(CHCH2)2(C6H5)2(CO2)2]12	0	7396.239936	Not in OntoMOPs KG
(4-planar)x6(2-bent)x12___(Oh)	[Fe2]6[(C10H4)(CO)4N2)(CHCH3)2(CO2)2]12	0	5569.947456	Not in OntoMOPs KG
(4-planar)x6(2-bent)x12___(Oh)	[Fe2]6[(C12H6NH)(C6H4)2(CO2)2]12	0	5510.991768	Not in OntoMOPs KG
(4-planar)x6(2-bent)x12___(Oh)	[Co2]6[(C6H3)2NH(CO2)2]12	0	3745.709688	Not in OntoMOPs KG

(4-planar)x6(2-bent)x12___(Oh)	[Co2]6[(C6H3)2NC3H7(CO2)2]12	0	4250.658648	10.1021/acs.inorgchem.8b01130
(4-planar)x6(2-bent)x12___(Oh)	[Co2]6[S(C6H3)2(CO2)2]12	0	3950.315328	Not in OntoMOPs KG
(4-planar)x6(2-bent)x12___(Oh)	[Co2]6[(C4H2S)3(CO2)2]12	0	4719.875328	Not in OntoMOPs KG
(4-planar)x6(2-bent)x12___(Oh)	[Co2]6[(C10H4)(CO)4N2)(CHCH)2(CH3)2(CH3)2(CO2)2]12	0	6280.271136	Not in OntoMOPs KG
(4-planar)x6(2-bent)x12___(Oh)	[Co2]6[(C10H4)(CO)4N2)(CHCH)2(CH3)2(CH2CH3)2(CO2)2]12	0	6616.903776	Not in OntoMOPs KG
(4-planar)x6(2-bent)x12___(Oh)	[Co2]6[(C10H4)(CO)4N2)(CHCH2)2(C6H5)2(CO2)2]12	0	7433.298336	Not in OntoMOPs KG
(4-planar)x6(2-bent)x12___(Oh)	[Co2]6[(C10H4)(CO)4N2)(CHCH3)2(CO2)2]12	0	5607.005856	Not in OntoMOPs KG
(4-planar)x6(2-bent)x12___(Oh)	[Co2]6[(C12H6NH)(C6H4)2(CO2)2]12	0	5548.050168	Not in OntoMOPs KG
(4-planar)x6(2-bent)x12___(Oh)	[Cr2]6[(C6H3)2NH(CO2)2]12	0	3662.464488	10.1021/jacs.8b05780
(4-planar)x6(2-bent)x12___(Oh)	[Cr2]6[(C6H3)2NC3H7(CO2)2]12	0	4167.413448	Not in OntoMOPs KG
(4-planar)x6(2-bent)x12___(Oh)	[Cr2]6[S(C6H3)2(CO2)2]12	0	3867.070128	Not in OntoMOPs KG
(4-planar)x6(2-bent)x12___(Oh)	[Cr2]6[(C4H2S)3(CO2)2]12	0	4636.630128	Not in OntoMOPs KG
(4-planar)x6(2-bent)x12___(Oh)	[Cr2]6[(C10H4)(CO)4N2)(CHCH)2(CH3)2(CH3)2(CO2)2]12	0	6197.025936	Not in OntoMOPs KG
(4-planar)x6(2-bent)x12___(Oh)	[Cr2]6[(C10H4)(CO)4N2)(CHCH)2(CH3)2(CH2CH3)2(CO2)2]12	0	6533.658576	Not in OntoMOPs KG
(4-planar)x6(2-bent)x12___(Oh)	[Cr2]6[(C10H4)(CO)4N2)(CHCH2)2(C6H5)2(CO2)2]12	0	7350.053136	Not in OntoMOPs KG
(4-planar)x6(2-bent)x12___(Oh)	[Cr2]6[(C10H4)(CO)4N2)(CHCH3)2(CO2)2]12	0	5523.760656	Not in OntoMOPs KG
(4-planar)x6(2-bent)x12___(Oh)	[Cr2]6[(C12H6NH)(C6H4)2(CO2)2]12	0	5464.804968	Not in OntoMOPs KG
(4-planar)x6(2-bent)x12___(Oh)	[CuPd]6[(C6H3)2NH(CO2)2]12	0	4058.307288	Not in OntoMOPs KG
(4-planar)x6(2-bent)x12___(Oh)	[CuPd]6[(C6H3)2NC3H7(CO2)2]12	0	4563.256248	Not in OntoMOPs KG
(4-planar)x6(2-bent)x12___(Oh)	[CuPd]6[S(C6H3)2(CO2)2]12	0	4262.912928	Not in OntoMOPs KG
(4-planar)x6(2-bent)x12___(Oh)	[CuPd]6[(C4H2S)3(CO2)2]12	0	5032.472928	Not in OntoMOPs KG
(4-planar)x6(2-bent)x12___(Oh)	[CuPd]6[(C10H4)(CO)4N2)(CHCH)2(CH3)2(CH3)2(CO2)2]12	0	6592.868736	Not in OntoMOPs KG
(4-planar)x6(2-bent)x12___(Oh)	[CuPd]6[(C10H4)(CO)4N2)(CHCH)2(CH3)2(CH2CH3)2(CO2)2]12	0	6929.501376	Not in OntoMOPs KG
(4-planar)x6(2-bent)x12___(Oh)	[CuPd]6[(C10H4)(CO)4N2)(CHCH2)2(C6H5)2(CO2)2]12	0	7745.895936	Not in OntoMOPs KG
(4-planar)x6(2-bent)x12___(Oh)	[CuPd]6[(C10H4)(CO)4N2)(CHCH3)2(CO2)2]12	0	5919.603456	Not in OntoMOPs KG
(4-planar)x6(2-bent)x12___(Oh)	[CuPd]6[(C12H6NH)(C6H4)2(CO2)2]12	0	5860.647768	Not in OntoMOPs KG
(4-planar)x6(2-bent)x12___(Oh)	[CuNi]6[(C6H3)2NH(CO2)2]12	0	3771.947688	Not in OntoMOPs KG
(4-planar)x6(2-bent)x12___(Oh)	[CuNi]6[(C6H3)2NC3H7(CO2)2]12	0	4276.896648	Not in OntoMOPs KG
(4-planar)x6(2-bent)x12___(Oh)	[CuNi]6[S(C6H3)2(CO2)2]12	0	3976.553328	Not in OntoMOPs KG
(4-planar)x6(2-bent)x12___(Oh)	[CuNi]6[(C4H2S)3(CO2)2]12	0	4746.113328	Not in OntoMOPs KG
(4-planar)x6(2-bent)x12___(Oh)	[CuNi]6[(C10H4)(CO)4N2)(CHCH)2(CH3)2(CH3)2(CO2)2]12	0	6306.509136	Not in OntoMOPs KG
(4-planar)x6(2-bent)x12___(Oh)	[CuNi]6[(C10H4)(CO)4N2)(CHCH)2(CH3)2(CH2CH3)2(CO2)2]12	0	6643.141776	Not in OntoMOPs KG
(4-planar)x6(2-bent)x12___(Oh)	[CuNi]6[(C10H4)(CO)4N2)(CHCH2)2(C6H5)2(CO2)2]12	0	7459.536336	Not in OntoMOPs KG
(4-planar)x6(2-bent)x12___(Oh)	[CuNi]6[(C10H4)(CO)4N2)(CHCH3)2(CO2)2]12	0	5633.243856	Not in OntoMOPs KG
(4-planar)x6(2-bent)x12___(Oh)	[CuNi]6[(C12H6NH)(C6H4)2(CO2)2]12	0	5574.288168	Not in OntoMOPs KG
(4-planar)x6(2-bent)x12___(Oh)	[Mo2]6[(C6H3)2NH(CO2)2]12	0	4189.791288	10.1021/jacs.8b05780
(4-planar)x6(2-bent)x12___(Oh)	[Mo2]6[(C6H3)2NC3H7(CO2)2]12	0	4694.740248	Not in OntoMOPs KG
(4-planar)x6(2-bent)x12___(Oh)	[Mo2]6[S(C6H3)2(CO2)2]12	0	4394.396928	Not in OntoMOPs KG
(4-planar)x6(2-bent)x12___(Oh)	[Mo2]6[(C4H2S)3(CO2)2]12	0	5163.956928	Not in OntoMOPs KG
(4-planar)x6(2-bent)x12___(Oh)	[Mo2]6[(C10H4)(CO)4N2)(CHCH)2(CH3)2(CH3)2(CO2)2]12	0	6724.352736	Not in OntoMOPs KG
(4-planar)x6(2-bent)x12___(Oh)	[Mo2]6[(C10H4)(CO)4N2)(CHCH)2(CH3)2(CH2CH3)2(CO2)2]12	0	7060.985376	Not in OntoMOPs KG
(4-planar)x6(2-bent)x12___(Oh)	[Mo2]6[(C10H4)(CO)4N2)(CHCH2)2(C6H5)2(CO2)2]12	0	7877.379936	Not in OntoMOPs KG
(4-planar)x6(2-bent)x12___(Oh)	[Mo2]6[(C10H4)(CO)4N2)(CHCH3)2(CO2)2]12	0	6051.087456	Not in OntoMOPs KG
(4-planar)x6(2-bent)x12___(Oh)	[Mo2]6[(C12H6NH)(C6H4)2(CO2)2]12	0	5992.131768	10.1021/ja1080794
(3-pyramidal)x4(2-linear)x6___(Td)	[Zr3O(OH)3(C5H5)3]4[(C6H4)(CO2)2]6	4	3128.57492	10.1021/ic402428m
(3-pyramidal)x4(2-linear)x6___(Td)	[Zr3O(OH)3(C5H5)3]4[(C6H4)2(CO2)2]6	4	3585.14804	10.1021/ic402428m
(3-pyramidal)x4(2-linear)x6___(Td)	[Zr3O(OH)3(C5H5)3]4[(C6H3NH2)(CO2)2]6	4	3218.6621	10.1039/C7SC03847J
(3-pyramidal)x4(2-linear)x6___(Td)	[Zr3O(OH)3(C5H5)3]4[(C6H4)(C3H2N2)2]6	16	3381.098696	Not in OntoMOPs KG
(3-pyramidal)x4(2-linear)x6___(Td)	[Zr3O(OH)3(C5H5)3]4[(C6H3Br)(CO2)2]6	4	3601.95194	Not in OntoMOPs KG
(3-pyramidal)x4(2-linear)x6___(Td)	[Zr3O(OH)3(C5H5)3]4[(C6H4C)2(CO2)2]6	4	3729.27644	Not in OntoMOPs KG

(3-pyramidal)x4(2-linear)x6___(Td)	[Zr3O(OH)3(C5H5)3]4[C2O4]6	4	2672.0018	Not in OntoMOPs KG
(3-pyramidal)x4(2-linear)x6___(Td)	[Zr3O(OH)3(C5H5)3]4[(C16H12)(CO2)2]6	4	3897.59276	Not in OntoMOPs KG
(3-pyramidal)x4(2-linear)x6___(Td)	[Zr3O(OH)3(C5H5)3]4[(C5H3N)2(CO2)2]6	4	3597.00608	10.1002/chem.201604264
(3-pyramidal)x4(2-linear)x6___(Td)	[Zr3O(OH)3(C5H5)3]4[CuCl2(C5H3N)2(CO2)2]6	4	4403.71808	10.1002/chem.201604264
(3-pyramidal)x4(2-linear)x6___(Td)	[Zr3O(OH)3(C5H5)3]4[PdCl2(C5H3N)2(CO2)2]6	4	4660.96208	10.1002/chem.201604264
(3-pyramidal)x4(2-linear)x6___(Td)	[Zr3O(OH)3(C5H5)3]4[(C6H4)3(CO2)2]6	4	4041.72116	Not in OntoMOPs KG
(3-pyramidal)x4(2-linear)x6___(Td)	[Zr3O(OH)3(C5H5)3]4[(C10H6)(CO2)]6	4	3428.92568	Not in OntoMOPs KG
(3-pyramidal)x4(2-linear)x6___(Td)	[Zr3O(OH)3(C5H5)3]4[(C28H34N2O2)Mn(CO2)2]6	4	5080.150052	Not in OntoMOPs KG
(3-pyramidal)x4(2-linear)x6___(Td)	[V3O2(OH)2(HCO2)3]4[(C6H4)(CO2)2]6	0	2400.251944	Not in OntoMOPs KG
(3-pyramidal)x4(2-linear)x6___(Td)	[V3O2(OH)2(HCO2)3]4[(C6H4)2(CO2)2]6	0	2856.825064	Not in OntoMOPs KG
(3-pyramidal)x4(2-linear)x6___(Td)	[V3O2(OH)2(HCO2)3]4[(C6H3NH2)(CO2)2]6	0	2490.339124	Not in OntoMOPs KG
(3-pyramidal)x4(2-linear)x6___(Td)	[V3O2(OH)2(HCO2)3]4[(C6H4)(C3H2N2)2]6	12	2652.77572	10.1039/C5CC05913E
(3-pyramidal)x4(2-linear)x6___(Td)	[V3O2(OH)2(HCO2)3]4[(C6H3Br)(CO2)2]6	0	2873.628964	Not in OntoMOPs KG
(3-pyramidal)x4(2-linear)x6___(Td)	[V3O2(OH)2(HCO2)3]4[(C6H4C)2(CO2)2]6	0	3000.953464	Not in OntoMOPs KG
(3-pyramidal)x4(2-linear)x6___(Td)	[V3O2(OH)2(HCO2)3]4[C2O4]6	0	1943.678824	Not in OntoMOPs KG
(3-pyramidal)x4(2-linear)x6___(Td)	[V3O2(OH)2(HCO2)3]4[(C16H12)(CO2)2]6	0	3169.269784	Not in OntoMOPs KG
(3-pyramidal)x4(2-linear)x6___(Td)	[V3O2(OH)2(HCO2)3]4[(C5H3N)2(CO2)2]6	0	2868.683104	Not in OntoMOPs KG
(3-pyramidal)x4(2-linear)x6___(Td)	[V3O2(OH)2(HCO2)3]4[CuCl2(C5H3N)2(CO2)2]6	0	3675.395104	Not in OntoMOPs KG
(3-pyramidal)x4(2-linear)x6___(Td)	[V3O2(OH)2(HCO2)3]4[PdCl2(C5H3N)2(CO2)2]6	0	3932.639104	Not in OntoMOPs KG
(3-pyramidal)x4(2-linear)x6___(Td)	[V3O2(OH)2(HCO2)3]4[(C6H4)3(CO2)2]6	0	3313.398184	Not in OntoMOPs KG
(3-pyramidal)x4(2-linear)x6___(Td)	[V3O2(OH)2(HCO2)3]4[(C10H6)(CO2)]6	0	2700.602704	Not in OntoMOPs KG
(3-pyramidal)x4(2-linear)x6___(Td)	[V3O2(OH)2(HCO2)3]4[(C28H34N2O2)Mn(CO2)2]6	0	4351.827076	Not in OntoMOPs KG
(3-pyramidal)x4(2-linear)x6___(Td)	[Fe3O(SO4)3(C5H5N)3]4[(C6H4)(CO2)2]6	4	3820.775036	10.1021/ja042802q
(3-pyramidal)x4(2-linear)x6___(Td)	[Fe3O(SO4)3(C5H5N)3]4[(C6H4)2(CO2)2]6	4	4277.348156	10.1021/ja042802q
(3-pyramidal)x4(2-linear)x6___(Td)	[Fe3O(SO4)3(C5H5N)3]4[(C6H3NH2)(CO2)2]6	4	3910.862216	Not in OntoMOPs KG
(3-pyramidal)x4(2-linear)x6___(Td)	[Fe3O(SO4)3(C5H5N)3]4[(C6H4)(C3H2N2)2]6	16	4073.298812	Not in OntoMOPs KG
(3-pyramidal)x4(2-linear)x6___(Td)	[Fe3O(SO4)3(C5H5N)3]4[(C6H3Br)(CO2)2]6	4	4294.152056	Not in OntoMOPs KG
(3-pyramidal)x4(2-linear)x6___(Td)	[Fe3O(SO4)3(C5H5N)3]4[(C6H4C)2(CO2)2]6	4	4421.476556	Not in OntoMOPs KG
(3-pyramidal)x4(2-linear)x6___(Td)	[Fe3O(SO4)3(C5H5N)3]4[C2O4]6	4	3364.201916	Not in OntoMOPs KG
(3-pyramidal)x4(2-linear)x6___(Td)	[Fe3O(SO4)3(C5H5N)3]4[(C16H12)(CO2)2]6	4	4589.792876	10.1021/ja042802q
(3-pyramidal)x4(2-linear)x6___(Td)	[Fe3O(SO4)3(C5H5N)3]4[(C5H3N)2(CO2)2]6	4	4289.206196	Not in OntoMOPs KG
(3-pyramidal)x4(2-linear)x6___(Td)	[Fe3O(SO4)3(C5H5N)3]4[CuCl2(C5H3N)2(CO2)2]6	4	5095.918196	Not in OntoMOPs KG
(3-pyramidal)x4(2-linear)x6___(Td)	[Fe3O(SO4)3(C5H5N)3]4[PdCl2(C5H3N)2(CO2)2]6	4	5353.162196	Not in OntoMOPs KG
(3-pyramidal)x4(2-linear)x6___(Td)	[Fe3O(SO4)3(C5H5N)3]4[(C6H4)3(CO2)2]6	4	4733.921276	10.1021/ja042802q
(3-pyramidal)x4(2-linear)x6___(Td)	[Fe3O(SO4)3(C5H5N)3]4[(C10H6)(CO2)]6	4	4121.125796	Not in OntoMOPs KG
(3-pyramidal)x4(2-linear)x6___(Td)	[Fe3O(SO4)3(C5H5N)3]4[(C28H34N2O2)Mn(CO2)2]6	4	5772.350168	Not in OntoMOPs KG
(3-pyramidal)x4(2-linear)x6___(Td)	[V6O6(OCH3)9(SO4)]4[(C6H4)(CO2)2]6	-8	4092.73746	10.1039/C6CC04583A
(3-pyramidal)x4(2-linear)x6___(Td)	[V6O6(OCH3)9(SO4)]4[(C6H4)2(CO2)2]6	-8	4549.31058	Not in OntoMOPs KG
(3-pyramidal)x4(2-linear)x6___(Td)	[V6O6(OCH3)9(SO4)]4[(C6H3NH2)(CO2)2]6	-8	4182.82464	10.1039/C6CC04583A
(3-pyramidal)x4(2-linear)x6___(Td)	[V6O6(OCH3)9(SO4)]4[(C6H4)(C3H2N2)2]6	4	4345.261236	Not in OntoMOPs KG
(3-pyramidal)x4(2-linear)x6___(Td)	[V6O6(OCH3)9(SO4)]4[(C6H3Br)(CO2)2]6	-8	4566.11448	10.1039/C6CC04583A
(3-pyramidal)x4(2-linear)x6___(Td)	[V6O6(OCH3)9(SO4)]4[(C6H4C)2(CO2)2]6	-8	4693.43898	10.1002/anie.201811027
(3-pyramidal)x4(2-linear)x6___(Td)	[V6O6(OCH3)9(SO4)]4[C2O4]6	-8	3636.16434	Not in OntoMOPs KG
(3-pyramidal)x4(2-linear)x6___(Td)	[V6O6(OCH3)9(SO4)]4[(C16H12)(CO2)2]6	-8	4861.7553	Not in OntoMOPs KG
(3-pyramidal)x4(2-linear)x6___(Td)	[V6O6(OCH3)9(SO4)]4[(C5H3N)2(CO2)2]6	-8	4561.16862	Not in OntoMOPs KG
(3-pyramidal)x4(2-linear)x6___(Td)	[V6O6(OCH3)9(SO4)]4[CuCl2(C5H3N)2(CO2)2]6	-8	5367.88062	Not in OntoMOPs KG
(3-pyramidal)x4(2-linear)x6___(Td)	[V6O6(OCH3)9(SO4)]4[PdCl2(C5H3N)2(CO2)2]6	-8	5625.12462	Not in OntoMOPs KG
(3-pyramidal)x4(2-linear)x6___(Td)	[V6O6(OCH3)9(SO4)]4[(C6H4)3(CO2)2]6	-8	5005.8837	Not in OntoMOPs KG
(3-pyramidal)x4(2-linear)x6___(Td)	[V6O6(OCH3)9(SO4)]4[(C10H6)(CO2)]6	-8	4393.08822	Not in OntoMOPs KG
(3-pyramidal)x4(2-linear)x6___(Td)	[V6O6(OCH3)9(SO4)]4[(C28H34N2O2)Mn(CO2)2]6	-8	6044.312592	Not in OntoMOPs KG

(3-pyramidal)x4(2-linear)x6___(Td)	[V7O10(OCH3)9]4[(C6H4)(CO2)2]6	-12	4168.24346	10.1039/C6DT02764D
(3-pyramidal)x4(2-linear)x6___(Td)	[V7O10(OCH3)9]4[(C6H4)2(CO2)2]6	-12	4624.81658	Not in OntoMOPs KG
(3-pyramidal)x4(2-linear)x6___(Td)	[V7O10(OCH3)9]4[(C6H3NH2)(CO2)2]6	-12	4258.33064	10.1039/C6DT02764D
(3-pyramidal)x4(2-linear)x6___(Td)	[V7O10(OCH3)9]4[(C6H4)(C3H2N2)2]6	0	4420.767236	Not in OntoMOPs KG
(3-pyramidal)x4(2-linear)x6___(Td)	[V7O10(OCH3)9]4[(C6H3Br)(CO2)2]6	-12	4641.62048	Not in OntoMOPs KG
(3-pyramidal)x4(2-linear)x6___(Td)	[V7O10(OCH3)9]4[(C6H4C)2(CO2)2]6	-12	4768.94498	Not in OntoMOPs KG
(3-pyramidal)x4(2-linear)x6___(Td)	[V7O10(OCH3)9]4[C2O4]6	-12	3711.67034	Not in OntoMOPs KG
(3-pyramidal)x4(2-linear)x6___(Td)	[V7O10(OCH3)9]4[(C16H12)(CO2)2]6	-12	4937.2613	Not in OntoMOPs KG
(3-pyramidal)x4(2-linear)x6___(Td)	[V7O10(OCH3)9]4[(C5H3N)2(CO2)2]6	-12	4636.67462	Not in OntoMOPs KG
(3-pyramidal)x4(2-linear)x6___(Td)	[V7O10(OCH3)9]4[CuCl2(C5H3N)2(CO2)2]6	-12	5443.38662	Not in OntoMOPs KG
(3-pyramidal)x4(2-linear)x6___(Td)	[V7O10(OCH3)9]4[PdCl2(C5H3N)2(CO2)2]6	-12	5700.63062	Not in OntoMOPs KG
(3-pyramidal)x4(2-linear)x6___(Td)	[V7O10(OCH3)9]4[(C6H4)3(CO2)2]6	-12	5081.3897	Not in OntoMOPs KG
(3-pyramidal)x4(2-linear)x6___(Td)	[V7O10(OCH3)9]4[(C10H6)(CO2)]6	-12	4468.59422	Not in OntoMOPs KG
(3-pyramidal)x4(2-linear)x6___(Td)	[V7O10(OCH3)9]4[(C28H34N2O2)Mn(CO2)2]6	-12	6119.818592	Not in OntoMOPs KG
(3-pyramidal)x4(2-linear)x6___(Td)	[Pd3PO(C3H7N)3]4[(C6H4)(CO2)2]6	0	3134.745788	Not in OntoMOPs KG
(3-pyramidal)x4(2-linear)x6___(Td)	[Pd3PO(C3H7N)3]4[(C6H4)2(CO2)2]6	0	3591.318908	Not in OntoMOPs KG
(3-pyramidal)x4(2-linear)x6___(Td)	[Pd3PO(C3H7N)3]4[(C6H3NH2)(CO2)2]6	0	3224.832968	Not in OntoMOPs KG
(3-pyramidal)x4(2-linear)x6___(Td)	[Pd3PO(C3H7N)3]4[(C6H4)(C3H2N2)2]6	12	3387.269564	Not in OntoMOPs KG
(3-pyramidal)x4(2-linear)x6___(Td)	[Pd3PO(C3H7N)3]4[(C6H3Br)(CO2)2]6	0	3608.122808	Not in OntoMOPs KG
(3-pyramidal)x4(2-linear)x6___(Td)	[Pd3PO(C3H7N)3]4[(C6H4C)2(CO2)2]6	0	3735.447308	Not in OntoMOPs KG
(3-pyramidal)x4(2-linear)x6___(Td)	[Pd3PO(C3H7N)3]4[C2O4]6	0	2678.172668	10.1021/ic502798r
(3-pyramidal)x4(2-linear)x6___(Td)	[Pd3PO(C3H7N)3]4[(C16H12)(CO2)2]6	0	3903.763628	Not in OntoMOPs KG
(3-pyramidal)x4(2-linear)x6___(Td)	[Pd3PO(C3H7N)3]4[(C5H3N)2(CO2)2]6	0	3603.176948	Not in OntoMOPs KG
(3-pyramidal)x4(2-linear)x6___(Td)	[Pd3PO(C3H7N)3]4[CuCl2(C5H3N)2(CO2)2]6	0	4409.888948	Not in OntoMOPs KG
(3-pyramidal)x4(2-linear)x6___(Td)	[Pd3PO(C3H7N)3]4[PdCl2(C5H3N)2(CO2)2]6	0	4667.132948	Not in OntoMOPs KG
(3-pyramidal)x4(2-linear)x6___(Td)	[Pd3PO(C3H7N)3]4[(C6H4)3(CO2)2]6	0	4047.892028	Not in OntoMOPs KG
(3-pyramidal)x4(2-linear)x6___(Td)	[Pd3PO(C3H7N)3]4[(C10H6)(CO2)]6	0	3435.096548	Not in OntoMOPs KG
(3-pyramidal)x4(2-linear)x6___(Td)	[Pd3PO(C3H7N)3]4[(C28H34N2O2)Mn(CO2)2]6	0	5086.32092	Not in OntoMOPs KG
(3-pyramidal)x8(2-bent)x12___(Th)	[PW9O37Ni6NH2C4H3]8[(C6H4)(CO2)2]12	-24	23567.67866	Not in OntoMOPs KG
(3-pyramidal)x8(2-bent)x12___(Th)	[PW9O37Ni6NH2C4H3]8[(C6H3OH)(CO2)2]12	-24	23759.67256	Not in OntoMOPs KG
(3-pyramidal)x8(2-bent)x12___(Th)	[PW9O37Ni6NH2C4H3]8[(C6H3(NO2))(CO2)2]12	-24	24107.65289	Not in OntoMOPs KG
(3-pyramidal)x8(2-bent)x12___(Th)	[PW9O37Ni6NH2C4H3]8[(C6H3(CH3))(CO2)2]12	-24	23735.99498	Not in OntoMOPs KG
(3-pyramidal)x8(2-bent)x12___(Th)	[PW9O37Ni6NH2C4H3]8[(C6H3Br)(CO2)2]12	-24	24514.4327	Not in OntoMOPs KG
(3-pyramidal)x8(2-bent)x12___(Th)	[PW9O37Ni6NH2C4H3]8[(C6H3)((C3H6O2)(C9H5O2))(CO2)2]12	-24	26186.13463	Not in OntoMOPs KG
(3-pyramidal)x8(2-bent)x12___(Th)	[PW9O37Ni6NH2C4H3]8[(C6H3)((C6H12O2)(C9H5O2))(CO2)2]12	-24	26691.08359	Not in OntoMOPs KG
(3-pyramidal)x8(2-bent)x12___(Th)	[PW9O37Ni6NH2C4H3]8[(C6H3)(OC2H4)3(OCH3)(CO2)2]12	-24	25513.86847	Not in OntoMOPs KG
(3-pyramidal)x8(2-bent)x12___(Th)	[PW9O37Ni6NH2C4H3]8[(C6H3)(OCH2C2H)(CO2)2]12	-24	24216.24568	Not in OntoMOPs KG
(3-pyramidal)x8(2-bent)x12___(Th)	[PW9O37Ni6NH2C4H3]8[(C6H3NH2)(CO2)2]12	-24	23747.85302	Not in OntoMOPs KG
(3-pyramidal)x8(2-bent)x12___(Th)	[PW9O37Ni6NH2C4H3]8[(C6H3)(OCH2CH2CH3)(CO2)2]12	-24	24264.62152	Not in OntoMOPs KG
(3-pyramidal)x8(2-bent)x12___(Th)	[PW9O37Ni6NH2C4H3]8[(C10H6)(CO2)2]12	-24	24168.38018	Not in OntoMOPs KG
(3-pyramidal)x8(2-bent)x12___(Th)	[PW9O37Ni6NH2C4H3]8[(C6H3)2(CO2)2]12	-36	24083.70089	Not in OntoMOPs KG
(3-pyramidal)x8(2-bent)x12___(Th)	[PW9O37Ni6NH2C4H3]8[(C6H3)(OCH2CH3)(CO2)2]12	-24	24096.3052	Not in OntoMOPs KG
(3-pyramidal)x8(2-bent)x12___(Th)	[PW9O37Ni6NH2C4H3]8[(C6H3)(OC2H4)2(OH)(CO2)2]12	-24	24816.92562	Not in OntoMOPs KG
(3-pyramidal)x8(2-bent)x12___(Th)	[PW9O37Ni6NH2C4H3]8[(C6H3)(OC2H4)3(OH)(CO2)2]12	-24	25345.55215	Not in OntoMOPs KG
(3-pyramidal)x8(2-bent)x12___(Th)	[PW9O37Ni6NH2C4H3]8[(C6H3)O(CH2)11CH3(CO2)2]12	-24	25779.4684	Not in OntoMOPs KG
(3-pyramidal)x8(2-bent)x12___(Th)	[PW9O37Ni6NH2C4H3]8[(C6H3)O(CH2)4CH3(CO2)2]12	-24	24601.25416	Not in OntoMOPs KG
(3-pyramidal)x8(2-bent)x12___(Th)	[PW9O37Ni6NH2C4H3]8[(C6H3)(OC2H4OH)(CO2)2]12	-24	24288.29909	Not in OntoMOPs KG
(3-pyramidal)x8(2-bent)x12___(Th)	[PW9O37Ni6NH2C4H3]8[(C6H3)NHCOC(CH3)3(CO2)2]12	-24	24757.2406	Not in OntoMOPs KG
(3-pyramidal)x8(2-bent)x12___(Th)	[PW9O37Ni6NH2C4H3]8[(C6H3)CH2CS2(C6H5)(CO2)2]12	-24	25562.82962	Not in OntoMOPs KG
(3-pyramidal)x8(2-bent)x12___(Th)	[PW9O37Ni6NH2C4H3]8[C6H3CH2CS3C4H9(CO2)2]12	-24	25707.72866	Not in OntoMOPs KG

(3-pyramidal)x8(2-bent)x12___(Th)	[PW9O37Ni6NH2C4H3]8[C6H3C2Si(C3H7)3(CO2)2]12	-24	25731.99626	Not in OntoMOPs KG
(3-pyramidal)x8(2-bent)x12___(Th)	[PW9O37Ni6NH2C4H3]8[(C6H3)(CH2)(C4N2H2O2CH3)(CO2)2]12	-24	25225.16141	Not in OntoMOPs KG
(3-pyramidal)x8(2-bent)x12___(Th)	[PW9O37Ni6NH2C4H3]8[(C6H3)((C2H4O2)(C9H5O2))(CO2)2]12	-24	26017.81831	Not in OntoMOPs KG
(3-pyramidal)x8(2-bent)x12___(Th)	[PW9O37Ni6NH2C4H3]8[(C6H3)(N2(C6H4CH3))(CO2)2]12	-24	24985.30202	Not in OntoMOPs KG
(3-pyramidal)x8(2-bent)x12___(Th)	[PW9O37Ni6NH2C4H3]8[((C6H3)C(CH3)3)(CO2)2]12	-24	24240.94394	Not in OntoMOPs KG
(3-pyramidal)x8(2-bent)x12___(Th)	[PW9O37Ni6NH2C4H3]8[(C6H3SO3)(CO2)2]12	-24	24516.34638	Not in OntoMOPs KG
(3-pyramidal)x8(2-bent)x12___(Th)	[PW9O37Ni6NH2C4H3]8[(C6H3)O(CH2)13CH3(CO2)2]12	-24	26116.10104	Not in OntoMOPs KG
(3-pyramidal)x8(2-bent)x12___(Th)	[PW9O37Ni6NH2C4H3]8[C6H3CO2C2H4Si(CH3)3(CO2)2]12	-24	25298.59037	Not in OntoMOPs KG
(3-pyramidal)x8(2-bent)x12___(Th)	[PW9O37Ni6NH2C4H3]8[(C6H3)(CO2)3]12	-36	24083.70089	10.1021/ja105986b
(3-pyramidal)x8(2-bent)x12___(Th)	[PW9O37Ni6NH2C4H3]8[C6H3CO2CH3(CO2)2]12	-24	24264.11117	Not in OntoMOPs KG
(3-pyramidal)x8(2-bent)x12___(Th)	[PW9O37Ni6NH2C4H3]8[C6H3OCOCHCH2(CO2)2]12	-24	24408.23957	Not in OntoMOPs KG
(3-pyramidal)x8(2-bent)x12___(Th)	[PW9O37Ni6NH2C4H3]8[C6H3NHCO2C(CH3)3(CO2)2]12	-24	24949.23449	Not in OntoMOPs KG
(3-pyramidal)x8(2-bent)x12___(Th)	[PW9O37Ni6NH2C4H3]8[(C6H2O)(CH2CHCH2)(CO2)2]12	-24	24240.4336	Not in OntoMOPs KG
(3-pyramidal)x8(2-bent)x12___(Th)	[PW9O37Ni6NH2C4H3]8[(C6H3)O(CH2)3CH3(CO2)2]12	-24	24432.93784	Not in OntoMOPs KG
(3-pyramidal)x8(2-bent)x12___(Th)	[PW9O37Ni6NH2C4H3]8[(C6H3)C6H2(OC16H33)3(CO2)2]12	-24	33111.80202	Not in OntoMOPs KG
(3-pyramidal)x8(2-bent)x12___(Th)	[PW9O37Ni6NH2C4H3]8[C6H3N2C6H3(CH3)2(CO2)2]12	-24	25153.61834	Not in OntoMOPs KG
(3-pyramidal)x8(2-bent)x12___(Th)	[PW9O37Ni6NH2C4H3]8[(C6H3)(C2H4O2)(CONHC6H5)(CO2)2]12	-24	25717.74198	Not in OntoMOPs KG
(3-planar)x4(3-pyramidal)x4___(Td)	[(C3N3)(C6H4)3(CO2)3]4[Zr3O(OH)3(C5H5)3]4	4	3909.4508	Not in OntoMOPs KG
(3-planar)x4(3-pyramidal)x4___(Td)	[(C3N3)(C6H4)3(CO2)3]4[V3O2(OH)2(HCO2)3]4	0	3181.127824	Not in OntoMOPs KG
(3-planar)x4(3-pyramidal)x4___(Td)	[(C3N3)(C6H4)3(CO2)3]4[Fe3O(SO4)3(C5H5N)3]4	4	4601.650916	Not in OntoMOPs KG
(3-planar)x4(3-pyramidal)x4___(Td)	[(C3N3)(C6H4)3(CO2)3]4[V6O6(OCH3)9(SO4)]4	-8	4873.61334	10.1039/C8DT02580K
(3-planar)x4(3-pyramidal)x4___(Td)	[(C3N3)(C6H4)3(CO2)3]4[V7O10(OCH3)9]4	-12	4949.11934	10.1039/C8DT02580K
(3-planar)x4(3-pyramidal)x4___(Td)	[(C3N3)(C6H4)3(CO2)3]4[Pd3PO(C3H7N)3]4	0	3915.621668	Not in OntoMOPs KG
(3-planar)x4(3-pyramidal)x4___(Td)	[(C6H3)(C6H4)3(CO2)3]4[Zr3O(OH)3(C5H5)3]4	4	3885.4988	10.1021/ic402428m
(3-planar)x4(3-pyramidal)x4___(Td)	[(C6H3)(C6H4)3(CO2)3]4[V3O2(OH)2(HCO2)3]4	0	3157.175824	Not in OntoMOPs KG
(3-planar)x4(3-pyramidal)x4___(Td)	[(C6H3)(C6H4)3(CO2)3]4[Fe3O(SO4)3(C5H5N)3]4	4	4577.698916	10.1021/ja042802q
(3-planar)x4(3-pyramidal)x4___(Td)	[(C6H3)(C6H4)3(CO2)3]4[V6O6(OCH3)9(SO4)]4	-8	4849.66134	10.1039/C6CC04583A
(3-planar)x4(3-pyramidal)x4___(Td)	[(C6H3)(C6H4)3(CO2)3]4[V7O10(OCH3)9]4	-12	4925.16734	Not in OntoMOPs KG
(3-planar)x4(3-pyramidal)x4___(Td)	[(C6H3)(C6H4)3(CO2)3]4[Pd3PO(C3H7N)3]4	0	3891.669668	Not in OntoMOPs KG
(3-planar)x4(3-pyramidal)x4___(Td)	[(C6H3)(CO2)3]4[Zr3O(OH)3(C5H5)3]4	4	2972.35256	10.1021/ic402428m
(3-planar)x4(3-pyramidal)x4___(Td)	[(C6H3)(CO2)3]4[V3O2(OH)2(HCO2)3]4	0	2244.029584	Not in OntoMOPs KG
(3-planar)x4(3-pyramidal)x4___(Td)	[(C6H3)(CO2)3]4[Fe3O(SO4)3(C5H5N)3]4	4	3664.552676	Not in OntoMOPs KG
(3-planar)x4(3-pyramidal)x4___(Td)	[(C6H3)(CO2)3]4[V6O6(OCH3)9(SO4)]4	-8	3936.5151	10.1039/C6CC04583A
(3-planar)x4(3-pyramidal)x4___(Td)	[(C6H3)(CO2)3]4[V7O10(OCH3)9]4	-12	4012.0211	Not in OntoMOPs KG
(3-planar)x4(3-pyramidal)x4___(Td)	[(C6H3)(CO2)3]4[Pd3PO(C3H7N)3]4	0	2978.523428	Not in OntoMOPs KG
(3-planar)x4(3-pyramidal)x4___(Td)	[(C6H3)(C2C6H4)3(CO2)3]4[Zr3O(OH)3(C5H5)3]4	4	4173.7556	Not in OntoMOPs KG
(3-planar)x4(3-pyramidal)x4___(Td)	[(C6H3)(C2C6H4)3(CO2)3]4[V3O2(OH)2(HCO2)3]4	0	3445.432624	Not in OntoMOPs KG
(3-planar)x4(3-pyramidal)x4___(Td)	[(C6H3)(C2C6H4)3(CO2)3]4[Fe3O(SO4)3(C5H5N)3]4	4	4865.955716	Not in OntoMOPs KG
(3-planar)x4(3-pyramidal)x4___(Td)	[(C6H3)(C2C6H4)3(CO2)3]4[V6O6(OCH3)9(SO4)]4	-8	5137.91814	Not in OntoMOPs KG
(3-planar)x4(3-pyramidal)x4___(Td)	[(C6H3)(C2C6H4)3(CO2)3]4[V7O10(OCH3)9]4	-12	5213.42414	Not in OntoMOPs KG
(3-planar)x4(3-pyramidal)x4___(Td)	[(C6H3)(C2C6H4)3(CO2)3]4[Pd3PO(C3H7N)3]4	0	4179.926468	Not in OntoMOPs KG
(3-planar)x4(3-pyramidal)x4___(Td)	[(C6H3)((C6H4)2)3(CO2)3]4[Zr3O(OH)3(C5H5)3]4	4	4798.64504	Not in OntoMOPs KG
(3-planar)x4(3-pyramidal)x4___(Td)	[(C6H3)((C6H4)2)3(CO2)3]4[V3O2(OH)2(HCO2)3]4	0	4070.322064	Not in OntoMOPs KG
(3-planar)x4(3-pyramidal)x4___(Td)	[(C6H3)((C6H4)2)3(CO2)3]4[Fe3O(SO4)3(C5H5N)3]4	4	5490.845156	Not in OntoMOPs KG
(3-planar)x4(3-pyramidal)x4___(Td)	[(C6H3)((C6H4)2)3(CO2)3]4[V6O6(OCH3)9(SO4)]4	-8	5762.80758	Not in OntoMOPs KG
(3-planar)x4(3-pyramidal)x4___(Td)	[(C6H3)((C6H4)2)3(CO2)3]4[V7O10(OCH3)9]4	-12	5838.31358	Not in OntoMOPs KG
(3-planar)x4(3-pyramidal)x4___(Td)	[(C6H3)((C6H4)2)3(CO2)3]4[Pd3PO(C3H7N)3]4	0	4804.815908	Not in OntoMOPs KG
(3-planar)x4(3-pyramidal)x4___(Td)	[(C5NH3)2(CO2)2]4[Zr3O(OH)3(C5H5)3]4	8	3112.632592	Not in OntoMOPs KG
(3-planar)x4(3-pyramidal)x4___(Td)	[(C5NH3)2(CO2)2]4[V3O2(OH)2(HCO2)3]4	4	2384.309616	Not in OntoMOPs KG
(3-planar)x4(3-pyramidal)x4___(Td)	[(C5NH3)2(CO2)2]4[Fe3O(SO4)3(C5H5N)3]4	8	3804.832708	Not in OntoMOPs KG

(3-planar)x4(3-pyramidal)x4___(Td)	[(C5NH3)2(CO2)2]4[V6O6(OCH3)9(SO4)]4	-4	4076.795132	Not in OntoMOPs KG
(3-planar)x4(3-pyramidal)x4___(Td)	[(C5NH3)2(CO2)2]4[V7O10(OCH3)9]4	-8	4152.301132	Not in OntoMOPs KG
(3-planar)x4(3-pyramidal)x4___(Td)	[(C5NH3)2(CO2)2]4[Pd3PO(C3H7N)3]4	4	3118.80346	Not in OntoMOPs KG
(5-pyramidal)x12(3-planar)x20___(Ih)	[WV5O11]12[(C3N3)(C6H4)3(CO2)3]20	-12	16202.32873	10.1021/jacs.8b10866
(5-pyramidal)x12(3-planar)x20___(Ih)	[WV5O11]12[(C6H3)(C6H4)3(CO2)3]20	-12	16082.56873	Not in OntoMOPs KG
(5-pyramidal)x12(3-planar)x20___(Ih)	[WV5O11]12[(C6H3)(CO2)3]20	-12	11516.83753	10.1021/jacs.8b10866
(5-pyramidal)x12(3-planar)x20___(Ih)	[WV5O11]12[(C6H3)(C2C6H4)3(CO2)3]20	-12	17523.85273	Not in OntoMOPs KG
(5-pyramidal)x12(3-planar)x20___(Ih)	[WV5O11]12[(C6H3)((C6H4)2)3(CO2)3]20	-12	20648.29993	Not in OntoMOPs KG
(5-pyramidal)x12(3-planar)x20___(Ih)	[WV5O11]12[(C5NH3)2(CO2)2]20	8	12218.23769	Not in OntoMOPs KG
(5-pyramidal)x12(3-planar)x20___(Ih)	[V6O11]12[(C3N3)(C6H4)3(CO2)3]20	0	14607.54673	Not in OntoMOPs KG
(5-pyramidal)x12(3-planar)x20___(Ih)	[V6O11]12[(C6H3)(C6H4)3(CO2)3]20	0	14487.78673	Not in OntoMOPs KG
(5-pyramidal)x12(3-planar)x20___(Ih)	[V6O11]12[(C6H3)(CO2)3]20	0	9922.055532	10.1002/anie.201900519
(5-pyramidal)x12(3-planar)x20___(Ih)	[V6O11]12[(C6H3)(C2C6H4)3(CO2)3]20	0	15929.07073	Not in OntoMOPs KG
(5-pyramidal)x12(3-planar)x20___(Ih)	[V6O11]12[(C6H3)((C6H4)2)3(CO2)3]20	0	19053.51793	Not in OntoMOPs KG
(5-pyramidal)x12(3-planar)x20___(Ih)	[V6O11]12[(C5NH3)2(CO2)2]20	20	10623.45569	Not in OntoMOPs KG
(5-pyramidal)x12(2-linear)x30___(Ih)	[WV5O11]12[(C6H4)(CO2)2]30	-12	12297.94933	10.1021/jacs.8b10866
(5-pyramidal)x12(2-linear)x30___(Ih)	[WV5O11]12[(C6H4)2(CO2)2]30	-12	14580.81493	Not in OntoMOPs KG
(5-pyramidal)x12(2-linear)x30___(Ih)	[WV5O11]12[(C6H3NH2)(CO2)2]30	-12	12748.38523	Not in OntoMOPs KG
(5-pyramidal)x12(2-linear)x30___(Ih)	[WV5O11]12[(C6H4)(C3H2N2)2]30	48	13560.56821	Not in OntoMOPs KG
(5-pyramidal)x12(2-linear)x30___(Ih)	[WV5O11]12[(C6H3Br)(CO2)2]30	-12	14664.83443	Not in OntoMOPs KG
(5-pyramidal)x12(2-linear)x30___(Ih)	[WV5O11]12[(C6H4C)2(CO2)2]30	-12	15301.45693	Not in OntoMOPs KG
(5-pyramidal)x12(2-linear)x30___(Ih)	[WV5O11]12[C2O4]30	-12	10015.08373	Not in OntoMOPs KG
(5-pyramidal)x12(2-linear)x30___(Ih)	[WV5O11]12[(C16H12)(CO2)2]30	-12	16143.03853	Not in OntoMOPs KG
(5-pyramidal)x12(2-linear)x30___(Ih)	[WV5O11]12[(C5H3N)2(CO2)2]30	-12	14640.10513	Not in OntoMOPs KG
(5-pyramidal)x12(2-linear)x30___(Ih)	[WV5O11]12[CuCl2(C5H3N)2(CO2)2]30	-12	18673.66513	Not in OntoMOPs KG
(5-pyramidal)x12(2-linear)x30___(Ih)	[WV5O11]12[PdCl2(C5H3N)2(CO2)2]30	-12	19959.88513	Not in OntoMOPs KG
(5-pyramidal)x12(2-linear)x30___(Ih)	[WV5O11]12[(C6H4)3(CO2)2]30	-12	16863.68053	Not in OntoMOPs KG
(5-pyramidal)x12(2-linear)x30___(Ih)	[WV5O11]12[(C10H6)(CO2)]30	-12	13799.70313	10.1021/jacs.8b10866
(5-pyramidal)x12(2-linear)x30___(Ih)	[WV5O11]12[(C28H34N2O2)Mn(CO2)2]30	-12	22055.82499	Not in OntoMOPs KG
(5-pyramidal)x12(2-linear)x30___(Ih)	[V6O11]12[(C6H4)(CO2)2]30	0	10703.16733	Not in OntoMOPs KG
(5-pyramidal)x12(2-linear)x30___(Ih)	[V6O11]12[(C6H4)2(CO2)2]30	0	12986.03293	Not in OntoMOPs KG
(5-pyramidal)x12(2-linear)x30___(Ih)	[V6O11]12[(C6H3NH2)(CO2)2]30	0	11153.60323	Not in OntoMOPs KG
(5-pyramidal)x12(2-linear)x30___(Ih)	[V6O11]12[(C6H4)(C3H2N2)2]30	60	11965.78621	Not in OntoMOPs KG
(5-pyramidal)x12(2-linear)x30___(Ih)	[V6O11]12[(C6H3Br)(CO2)2]30	0	13070.05243	Not in OntoMOPs KG
(5-pyramidal)x12(2-linear)x30___(Ih)	[V6O11]12[(C6H4C)2(CO2)2]30	0	13706.67493	Not in OntoMOPs KG
(5-pyramidal)x12(2-linear)x30___(Ih)	[V6O11]12[C2O4]30	0	8420.301732	Not in OntoMOPs KG
(5-pyramidal)x12(2-linear)x30___(Ih)	[V6O11]12[(C16H12)(CO2)2]30	0	14548.25653	Not in OntoMOPs KG
(5-pyramidal)x12(2-linear)x30___(Ih)	[V6O11]12[(C5H3N)2(CO2)2]30	0	13045.32313	Not in OntoMOPs KG
(5-pyramidal)x12(2-linear)x30___(Ih)	[V6O11]12[CuCl2(C5H3N)2(CO2)2]30	0	17078.88313	Not in OntoMOPs KG
(5-pyramidal)x12(2-linear)x30___(Ih)	[V6O11]12[PdCl2(C5H3N)2(CO2)2]30	0	18365.10313	Not in OntoMOPs KG
(5-pyramidal)x12(2-linear)x30___(Ih)	[V6O11]12[(C6H4)3(CO2)2]30	0	15268.89853	Not in OntoMOPs KG
(5-pyramidal)x12(2-linear)x30___(Ih)	[V6O11]12[(C10H6)(CO2)]30	0	12204.92113	Not in OntoMOPs KG
(5-pyramidal)x12(2-linear)x30___(Ih)	[V6O11]12[(C28H34N2O2)Mn(CO2)2]30	0	20461.04299	Not in OntoMOPs KG
(3-planar)x4(2-bent)x6___(Td)	[(C3N3)(C6H4)3(CO2)3]4[Cu2(C10H6N2O2C2H6)2]6	12	5128.981068	Not in OntoMOPs KG
(3-planar)x4(2-bent)x6___(Td)	[(C3N3)(C6H4)3(CO2)3]4[Mo2(HCN2C12H8C2H6)2]6	0	5596.341384	Not in OntoMOPs KG
(3-planar)x4(2-bent)x6___(Td)	[(C3N3)(C6H4)3(CO2)3]4[Mo2(HCN2C12H8O2C2H6)2]6	0	5980.329168	Not in OntoMOPs KG
(3-planar)x4(2-bent)x6___(Td)	[(C3N3)(C6H4)3(CO2)3]4[Rh2(HCN2C12H8O2C2H6)2]6	0	6063.915168	Not in OntoMOPs KG
(3-planar)x4(2-bent)x6___(Td)	[(C6H3)(C6H4)3(CO2)3]4[Cu2(C10H6N2O2C2H6)2]6	12	5105.029068	Not in OntoMOPs KG
(3-planar)x4(2-bent)x6___(Td)	[(C6H3)(C6H4)3(CO2)3]4[Mo2(HCN2C12H8C2H6)2]6	0	5572.389384	Not in OntoMOPs KG
(3-planar)x4(2-bent)x6___(Td)	[(C6H3)(C6H4)3(CO2)3]4[Mo2(HCN2C12H8O2C2H6)2]6	0	5956.377168	Not in OntoMOPs KG

(3-planar)x4(2-bent)x6___(Td)	[(C6H3)(C6H4)3(CO2)3]4[Rh2(HCN2C12H8O2C2H6)2]6	0	6039.963168	Not in OntoMOPs KG
(3-planar)x4(2-bent)x6___(Td)	[(C6H3)(CO2)3]4[Cu2(C10H6N2O2C2H6)2]6	12	4191.882828	10.1039/C9CC05002G
(3-planar)x4(2-bent)x6___(Td)	[(C6H3)(CO2)3]4[Mo2(HCN2C12H8C2H6)2]6	0	4659.243144	10.1039/C9CC05002G
(3-planar)x4(2-bent)x6___(Td)	[(C6H3)(CO2)3]4[Mo2(HCN2C12H8O2C2H6)2]6	0	5043.230928	10.1021/ic0104959
(3-planar)x4(2-bent)x6___(Td)	[(C6H3)(CO2)3]4[Rh2(HCN2C12H8O2C2H6)2]6	0	5126.816928	10.1021/ic0104959
(3-planar)x4(2-bent)x6___(Td)	[(C6H3)(C2C6H4)3(CO2)3]4[Cu2(C10H6N2O2C2H6)2]6	12	5393.285868	Not in OntoMOPs KG
(3-planar)x4(2-bent)x6___(Td)	[(C6H3)(C2C6H4)3(CO2)3]4[Mo2(HCN2C12H8C2H6)2]6	0	5860.646184	Not in OntoMOPs KG
(3-planar)x4(2-bent)x6___(Td)	[(C6H3)(C2C6H4)3(CO2)3]4[Mo2(HCN2C12H8O2C2H6)2]6	0	6244.633968	Not in OntoMOPs KG
(3-planar)x4(2-bent)x6___(Td)	[(C6H3)(C2C6H4)3(CO2)3]4[Rh2(HCN2C12H8O2C2H6)2]6	0	6328.219968	Not in OntoMOPs KG
(3-planar)x4(2-bent)x6___(Td)	[(C6H3)((C6H4)2)3(CO2)3]4[Cu2(C10H6N2O2C2H6)2]6	12	6018.175308	Not in OntoMOPs KG
(3-planar)x4(2-bent)x6___(Td)	[(C6H3)((C6H4)2)3(CO2)3]4[Mo2(HCN2C12H8C2H6)2]6	0	6485.535624	Not in OntoMOPs KG
(3-planar)x4(2-bent)x6___(Td)	[(C6H3)((C6H4)2)3(CO2)3]4[Mo2(HCN2C12H8O2C2H6)2]6	0	6869.523408	Not in OntoMOPs KG
(3-planar)x4(2-bent)x6___(Td)	[(C6H3)((C6H4)2)3(CO2)3]4[Rh2(HCN2C12H8O2C2H6)2]6	0	6953.109408	Not in OntoMOPs KG
(3-planar)x4(2-bent)x6___(Td)	[(C5NH3)2(CO2)2]4[Cu2(C10H6N2O2C2H6)2]6	16	4332.16286	Not in OntoMOPs KG
(3-planar)x4(2-bent)x6___(Td)	[(C5NH3)2(CO2)2]4[Mo2(HCN2C12H8C2H6)2]6	4	4799.523176	Not in OntoMOPs KG
(3-planar)x4(2-bent)x6___(Td)	[(C5NH3)2(CO2)2]4[Mo2(HCN2C12H8O2C2H6)2]6	4	5183.51096	Not in OntoMOPs KG
(3-planar)x4(2-bent)x6___(Td)	[(C5NH3)2(CO2)2]4[Rh2(HCN2C12H8O2C2H6)2]6	4	5267.09696	Not in OntoMOPs KG
(3-pyramidal)x2(2-bent)x3___(D3h)	[Zr3O(OH)3(C5H5)3]2[CH2(C6H4)2(CO2)2]3	2	1834.6531	10.1021/acsami.7b18836
(3-pyramidal)x2(2-bent)x3___(D3h)	[Zr3O(OH)3(C5H5)3]2[C2H4O2(C6H3SO3)2(CO2)2]3	-4	2447.062984	10.1039/C8TA00858B
(3-pyramidal)x2(2-bent)x3___(D3h)	[Zr3O(OH)3(C5H5)3]2[SO2(C6H4)2(CO2)2]3	-4	1984.765966	10.1021/acsami.7b18836
(3-pyramidal)x2(2-bent)x3___(D3h)	[V3O2(OH)2(HCO2)3]2[CH2(C6H4)2(CO2)2]3	0	1470.491612	Not in OntoMOPs KG
(3-pyramidal)x2(2-bent)x3___(D3h)	[V3O2(OH)2(HCO2)3]2[C2H4O2(C6H3SO3)2(CO2)2]3	-6	2082.901496	Not in OntoMOPs KG
(3-pyramidal)x2(2-bent)x3___(D3h)	[V3O2(OH)2(HCO2)3]2[SO2(C6H4)2(CO2)2]3	-6	1620.604478	Not in OntoMOPs KG
(3-pyramidal)x2(2-bent)x3___(D3h)	[Fe3O(SO4)3(C5H5N)3]2[CH2(C6H4)2(CO2)2]3	2	2180.753158	Not in OntoMOPs KG
(3-pyramidal)x2(2-bent)x3___(D3h)	[Fe3O(SO4)3(C5H5N)3]2[C2H4O2(C6H3SO3)2(CO2)2]3	-4	2793.163042	Not in OntoMOPs KG
(3-pyramidal)x2(2-bent)x3___(D3h)	[Fe3O(SO4)3(C5H5N)3]2[SO2(C6H4)2(CO2)2]3	-4	2330.866024	Not in OntoMOPs KG
(3-pyramidal)x2(2-bent)x3___(D3h)	[V6O6(OCH3)9(SO4)]2[CH2(C6H4)2(CO2)2]3	-4	2316.73437	Not in OntoMOPs KG
(3-pyramidal)x2(2-bent)x3___(D3h)	[V6O6(OCH3)9(SO4)]2[C2H4O2(C6H3SO3)2(CO2)2]3	-10	2929.144254	Not in OntoMOPs KG
(3-pyramidal)x2(2-bent)x3___(D3h)	[V6O6(OCH3)9(SO4)]2[SO2(C6H4)2(CO2)2]3	-10	2466.847236	Not in OntoMOPs KG
(3-pyramidal)x2(2-bent)x3___(D3h)	[V7O10(OCH3)9]2[CH2(C6H4)2(CO2)2]3	-6	2354.48737	Not in OntoMOPs KG
(3-pyramidal)x2(2-bent)x3___(D3h)	[V7O10(OCH3)9]2[C2H4O2(C6H3SO3)2(CO2)2]3	-12	2966.897254	Not in OntoMOPs KG
(3-pyramidal)x2(2-bent)x3___(D3h)	[V7O10(OCH3)9]2[SO2(C6H4)2(CO2)2]3	-12	2504.600236	Not in OntoMOPs KG
(3-pyramidal)x2(2-bent)x3___(D3h)	[Pd3PO(C3H7N)3]2[CH2(C6H4)2(CO2)2]3	0	1837.738534	Not in OntoMOPs KG
(3-pyramidal)x2(2-bent)x3___(D3h)	[Pd3PO(C3H7N)3]2[C2H4O2(C6H3SO3)2(CO2)2]3	-6	2450.148418	Not in OntoMOPs KG
(3-pyramidal)x2(2-bent)x3___(D3h)	[Pd3PO(C3H7N)3]2[SO2(C6H4)2(CO2)2]3	-6	1987.8514	Not in OntoMOPs KG
(3-planar)x8(2-bent)x12___(Oh)	[(C3N3)(C6H4)3(CO2)3]8[Cu2(C10H10N2)2]12	24	8804.627328	Not in OntoMOPs KG
(3-planar)x8(2-bent)x12___(Oh)	[(C6H3)(C6H4)3(CO2)3]8[Cu2(C10H10N2)2]12	24	8756.723328	Not in OntoMOPs KG
(3-planar)x8(2-bent)x12___(Oh)	[(C6H3)(CO2)3]8[Cu2(C10H10N2)2]12	24	6930.430848	10.1039/C9CC05002G
(3-planar)x8(2-bent)x12___(Oh)	[(C6H3)(C2C6H4)3(CO2)3]8[Cu2(C10H10N2)2]12	24	9333.236928	Not in OntoMOPs KG
(3-planar)x8(2-bent)x12___(Oh)	[(C6H3)((C6H4)2)3(CO2)3]8[Cu2(C10H10N2)2]12	24	10583.01581	Not in OntoMOPs KG
(3-planar)x8(2-bent)x12___(Oh)	[(C5NH3)2(CO2)2]8[Cu2(C10H10N2)2]12	32	7210.990912	Not in OntoMOPs KG
(4-pyramidal)x6(3-planar)x8___(Oh)	[Ni4C24H12O12S4]6[(C3N3)(C6H4)3(CO2)3]8	0	8663.39988	Not in OntoMOPs KG
(4-pyramidal)x6(3-planar)x8___(Oh)	[Ni4C24H12O12S4]6[(C6H3)(C6H4)3(CO2)3]8	0	8615.49588	Not in OntoMOPs KG
(4-pyramidal)x6(3-planar)x8___(Oh)	[Ni4C24H12O12S4]6[(C6H3)(CO2)3]8	0	6789.2034	10.1021/ja300095j
(4-pyramidal)x6(3-planar)x8___(Oh)	[Ni4C24H12O12S4]6[(C6H3)(C2C6H4)3(CO2)3]8	0	9192.00948	Not in OntoMOPs KG
(4-pyramidal)x6(3-planar)x8___(Oh)	[Ni4C24H12O12S4]6[(C6H3)((C6H4)2)3(CO2)3]8	0	10441.78836	Not in OntoMOPs KG
(4-pyramidal)x6(3-planar)x8___(Oh)	[Ni4C24H12O12S4]6[(C5NH3)2(CO2)2]8	8	7069.763464	Not in OntoMOPs KG
(4-pyramidal)x6(3-planar)x8___(Oh)	[V4O8]6[(C3N3)(C6H4)3(CO2)3]8	0	5521.701936	Not in OntoMOPs KG
(4-pyramidal)x6(3-planar)x8___(Oh)	[V4O8]6[(C6H3)(C6H4)3(CO2)3]8	0	5473.797936	Not in OntoMOPs KG
(4-pyramidal)x6(3-planar)x8___(Oh)	[V4O8]6[(C6H3)(CO2)3]8	0	3647.505456	10.1039/C3SC53099J

(4-pyramidal)x6(3-planar)x8___(Oh)	[V4O8]6[(C6H3)(C2C6H4)3(CO2)3]8	0	6050.311536	Not in OntoMOPs KG
(4-pyramidal)x6(3-planar)x8___(Oh)	[V4O8]6[(C6H3)((C6H4)2)3(CO2)3]8	0	7300.090416	Not in OntoMOPs KG
(4-pyramidal)x6(3-planar)x8___(Oh)	[V4O8]6[(C5NH3)2(CO2)2]8	8	3928.06552	Not in OntoMOPs KG
(4-pyramidal)x6(3-planar)x8___(Oh)	[V5O9]6[(C3N3)(C6H4)3(CO2)3]8	-6	5923.347882	Not in OntoMOPs KG
(4-pyramidal)x6(3-planar)x8___(Oh)	[V5O9]6[(C6H3)(C6H4)3(CO2)3]8	-6	5875.443882	Not in OntoMOPs KG
(4-pyramidal)x6(3-planar)x8___(Oh)	[V5O9]6[(C6H3)(CO2)3]8	-6	4049.151402	10.1039/C3SC53099J
(4-pyramidal)x6(3-planar)x8___(Oh)	[V5O9]6[(C6H3)(C2C6H4)3(CO2)3]8	-6	6451.957482	Not in OntoMOPs KG
(4-pyramidal)x6(3-planar)x8___(Oh)	[V5O9]6[(C6H3)((C6H4)2)3(CO2)3]8	-6	7701.736362	Not in OntoMOPs KG
(4-pyramidal)x6(3-planar)x8___(Oh)	[V5O9]6[(C5NH3)2(CO2)2]8	2	4329.711466	Not in OntoMOPs KG
(4-pyramidal)x6(3-planar)x8___(Oh)	[Co4C48H28O4S4]6[(C3N3)(C6H4)3(CO2)3]8	0	9247.710072	Not in OntoMOPs KG
(4-pyramidal)x6(3-planar)x8___(Oh)	[Co4C48H28O4S4]6[(C6H3)(C6H4)3(CO2)3]8	0	9199.806072	Not in OntoMOPs KG
(4-pyramidal)x6(3-planar)x8___(Oh)	[Co4C48H28O4S4]6[(C6H3)(CO2)3]8	0	7373.513592	10.1002/anie.201106732
(4-pyramidal)x6(3-planar)x8___(Oh)	[Co4C48H28O4S4]6[(C6H3)(C2C6H4)3(CO2)3]8	0	9776.319672	Not in OntoMOPs KG
(4-pyramidal)x6(3-planar)x8___(Oh)	[Co4C48H28O4S4]6[(C6H3)((C6H4)2)3(CO2)3]8	0	11026.09855	Not in OntoMOPs KG
(4-pyramidal)x6(3-planar)x8___(Oh)	[Co4C48H28O4S4]6[(C5NH3)2(CO2)2]8	8	7654.073656	Not in OntoMOPs KG
(4-pyramidal)x6(3-planar)x8___(Oh)	[Co4C40H44O4S4]6[(C3N3)(C6H4)3(CO2)3]8	0	10015.68564	Not in OntoMOPs KG
(4-pyramidal)x6(3-planar)x8___(Oh)	[Co4C40H44O4S4]6[(C6H3)(C6H4)3(CO2)3]8	0	9967.78164	10.1002/anie.201106732
(4-pyramidal)x6(3-planar)x8___(Oh)	[Co4C40H44O4S4]6[(C6H3)(CO2)3]8	0	8141.48916	10.1039/C2CC34265K
(4-pyramidal)x6(3-planar)x8___(Oh)	[Co4C40H44O4S4]6[(C6H3)(C2C6H4)3(CO2)3]8	0	10544.29524	Not in OntoMOPs KG
(4-pyramidal)x6(3-planar)x8___(Oh)	[Co4C40H44O4S4]6[(C6H3)((C6H4)2)3(CO2)3]8	0	11794.07412	Not in OntoMOPs KG
(4-pyramidal)x6(3-planar)x8___(Oh)	[Co4C40H44O4S4]6[(C5NH3)2(CO2)2]8	8	8422.049224	10.1021/ic501012e
(4-pyramidal)x6(3-planar)x8___(Oh)	[Co4C40H44O12S4]6[(C3N3)(C6H4)3(CO2)3]8	0	11362.2162	Not in OntoMOPs KG
(4-pyramidal)x6(3-planar)x8___(Oh)	[Co4C40H44O12S4]6[(C6H3)(C6H4)3(CO2)3]8	0	11314.3122	Not in OntoMOPs KG
(4-pyramidal)x6(3-planar)x8___(Oh)	[Co4C40H44O12S4]6[(C6H3)(CO2)3]8	0	9488.01972	Not in OntoMOPs KG
(4-pyramidal)x6(3-planar)x8___(Oh)	[Co4C40H44O12S4]6[(C6H3)(C2C6H4)3(CO2)3]8	0	11890.8258	10.1039/C2CC34265K
(4-pyramidal)x6(3-planar)x8___(Oh)	[Co4C40H44O12S4]6[(C6H3)((C6H4)2)3(CO2)3]8	0	13140.60468	10.1039/C5DT01526J
(4-pyramidal)x6(3-planar)x8___(Oh)	[Co4C40H44O12S4]6[(C5NH3)2(CO2)2]8	8	9768.579784	Not in OntoMOPs KG
(4-pyramidal)x6(3-planar)x8___(Oh)	[Ni4C40H44S4O12]6[(C3N3)(C6H4)3(CO2)3]8	0	10009.93044	Not in OntoMOPs KG
(4-pyramidal)x6(3-planar)x8___(Oh)	[Ni4C40H44S4O12]6[(C6H3)(C6H4)3(CO2)3]8	0	9962.02644	Not in OntoMOPs KG
(4-pyramidal)x6(3-planar)x8___(Oh)	[Ni4C40H44S4O12]6[(C6H3)(CO2)3]8	0	8135.73396	10.1021/ja300095j
(4-pyramidal)x6(3-planar)x8___(Oh)	[Ni4C40H44S4O12]6[(C6H3)(C2C6H4)3(CO2)3]8	0	10538.54004	Not in OntoMOPs KG
(4-pyramidal)x6(3-planar)x8___(Oh)	[Ni4C40H44S4O12]6[(C6H3)((C6H4)2)3(CO2)3]8	0	11788.31892	Not in OntoMOPs KG
(4-pyramidal)x6(3-planar)x8___(Oh)	[Ni4C40H44S4O12]6[(C5NH3)2(CO2)2]8	8	8416.294024	Not in OntoMOPs KG
(4-pyramidal)x6(3-planar)x8___(Oh)	[Mg4C40H44O12S4]6[(C3N3)(C6H4)3(CO2)3]8	0	9184.60884	Not in OntoMOPs KG
(4-pyramidal)x6(3-planar)x8___(Oh)	[Mg4C40H44O12S4]6[(C6H3)(C6H4)3(CO2)3]8	0	9136.70484	Not in OntoMOPs KG
(4-pyramidal)x6(3-planar)x8___(Oh)	[Mg4C40H44O12S4]6[(C6H3)(CO2)3]8	0	7310.41236	10.1021/ja300095j
(4-pyramidal)x6(3-planar)x8___(Oh)	[Mg4C40H44O12S4]6[(C6H3)(C2C6H4)3(CO2)3]8	0	9713.21844	Not in OntoMOPs KG
(4-pyramidal)x6(3-planar)x8___(Oh)	[Mg4C40H44O12S4]6[(C6H3)((C6H4)2)3(CO2)3]8	0	10962.99732	Not in OntoMOPs KG
(4-pyramidal)x6(3-planar)x8___(Oh)	[Mg4C40H44O12S4]6[(C5NH3)2(CO2)2]8	8	7590.972424	Not in OntoMOPs KG
(4-pyramidal)x6(3-planar)x8___(Oh)	[Fe4C40H44S4O4]6[(C3N3)(C6H4)3(CO2)3]8	0	9173.593272	Not in OntoMOPs KG
(4-pyramidal)x6(3-planar)x8___(Oh)	[Fe4C40H44S4O4]6[(C6H3)(C6H4)3(CO2)3]8	0	9125.689272	Not in OntoMOPs KG
(4-pyramidal)x6(3-planar)x8___(Oh)	[Fe4C40H44S4O4]6[(C6H3)(CO2)3]8	0	7299.396792	Not in OntoMOPs KG
(4-pyramidal)x6(3-planar)x8___(Oh)	[Fe4C40H44S4O4]6[(C6H3)(C2C6H4)3(CO2)3]8	0	9702.202872	Not in OntoMOPs KG
(4-pyramidal)x6(3-planar)x8___(Oh)	[Fe4C40H44S4O4]6[(C6H3)((C6H4)2)3(CO2)3]8	0	10951.98175	Not in OntoMOPs KG
(4-pyramidal)x6(3-planar)x8___(Oh)	[Fe4C40H44S4O4]6[(C5NH3)2(CO2)2]8	8	7579.956856	10.1021/ic501012e
(4-pyramidal)x6(3-planar)x8___(Oh)	[(CHC6HO3)4(C3H6OH)4]6[Cu3]8	-24	6161.314416	10.1073/pnas.0408113102
(4-pyramidal)x6(3-planar)x8___(Oh)	[(CHC6HO3)4(C3H6OH)4]6[Co3]8	0	6050.607216	Not in OntoMOPs KG
(4-pyramidal)x6(3-planar)x8___(Oh)	[(CHC6HO3)4(C3H6OH)4]6[Mg3]8	-24	5219.530416	Not in OntoMOPs KG
(4-pyramidal)x6(3-planar)x8___(Oh)	[(CHC6HO3)4(C3H6OH)4]6[V3O3]8	0	6242.7942	Not in OntoMOPs KG
(4-pyramidal)x6(3-planar)x8___(Oh)	[(CHC6HO3)4(C5H11)4]6[Cu3]8	-24	6450.591912	Not in OntoMOPs KG

(4-pyramidal)x6(3-planar)x8___(Oh)	[(CHC6HO3)4(C5H11)4]6[Co3]8	0	6339.884712	10.1021/acs.cgd.6b00306
(4-pyramidal)x6(3-planar)x8___(Oh)	[(CHC6HO3)4(C5H11)4]6[Mg3]8	-24	5508.807912	Not in OntoMOPs KG
(4-pyramidal)x6(3-planar)x8___(Oh)	[(CHC6HO3)4(C5H11)4]6[V3O3]8	0	6532.071696	Not in OntoMOPs KG
(4-pyramidal)x6(3-planar)x8___(Oh)	[(C6HO3)4(C4H8)4]6[Cu3]8	-24	5777.326632	Not in OntoMOPs KG
(4-pyramidal)x6(3-planar)x8___(Oh)	[(C6HO3)4(C4H8)4]6[Co3]8	0	5666.619432	Not in OntoMOPs KG
(4-pyramidal)x6(3-planar)x8___(Oh)	[(C6HO3)4(C4H8)4]6[Mg3]8	-24	4835.542632	10.1039/C7CC01208J
(4-pyramidal)x6(3-planar)x8___(Oh)	[(C6HO3)4(C4H8)4]6[V3O3]8	0	5858.806416	10.1038/s41467-018-07427-z
(4-pyramidal)x6(3-planar)x8___(Oh)	[Co4C24H8O24S8]6[(C3N3)(C6H4)3(CO2)3]8	0	8669.15508	Not in OntoMOPs KG
(4-pyramidal)x6(3-planar)x8___(Oh)	[Co4C24H8O24S8]6[(C6H3)(C6H4)3(CO2)3]8	0	8621.25108	10.1016/j.chempr.2018.01.004
(4-pyramidal)x6(3-planar)x8___(Oh)	[Co4C24H8O24S8]6[(C6H3)(CO2)3]8	0	6794.9586	Not in OntoMOPs KG
(4-pyramidal)x6(3-planar)x8___(Oh)	[Co4C24H8O24S8]6[(C6H3)(C2C6H4)3(CO2)3]8	0	9197.76468	Not in OntoMOPs KG
(4-pyramidal)x6(3-planar)x8___(Oh)	[Co4C24H8O24S8]6[(C6H3)((C6H4)2)3(CO2)3]8	0	10447.54356	Not in OntoMOPs KG
(4-pyramidal)x6(3-planar)x8___(Oh)	[Co4C24H8O24S8]6[(C5NH3)2(CO2)2]8	8	7075.518664	Not in OntoMOPs KG
(4-pyramidal)x6(3-planar)x8___(Oh)	[Co4C24H12O12S4]6[(C3N3)(C6H4)3(CO2)3]8	0	9727.471992	Not in OntoMOPs KG
(4-pyramidal)x6(3-planar)x8___(Oh)	[Co4C24H12O12S4]6[(C6H3)(C6H4)3(CO2)3]8	0	9679.567992	Not in OntoMOPs KG
(4-pyramidal)x6(3-planar)x8___(Oh)	[Co4C24H12O12S4]6[(C6H3)(CO2)3]8	0	7853.275512	Not in OntoMOPs KG
(4-pyramidal)x6(3-planar)x8___(Oh)	[Co4C24H12O12S4]6[(C6H3)(C2C6H4)3(CO2)3]8	0	10256.08159	Not in OntoMOPs KG
(4-pyramidal)x6(3-planar)x8___(Oh)	[Co4C24H12O12S4]6[(C6H3)((C6H4)2)3(CO2)3]8	0	11505.86047	Not in OntoMOPs KG
(4-pyramidal)x6(3-planar)x8___(Oh)	[Co4C24H12O12S4]6[(C5NH3)2(CO2)2]8	8	8133.835576	Not in OntoMOPs KG
(4-pyramidal)x6(3-planar)x8___(Oh)	[Zn4C40H44O12S4]6[(C3N3)(C6H4)3(CO2)3]8	0	10170.64884	Not in OntoMOPs KG
(4-pyramidal)x6(3-planar)x8___(Oh)	[Zn4C40H44O12S4]6[(C6H3)(C6H4)3(CO2)3]8	0	10122.74484	Not in OntoMOPs KG
(4-pyramidal)x6(3-planar)x8___(Oh)	[Zn4C40H44O12S4]6[(C6H3)(CO2)3]8	0	8296.45236	Not in OntoMOPs KG
(4-pyramidal)x6(3-planar)x8___(Oh)	[Zn4C40H44O12S4]6[(C6H3)(C2C6H4)3(CO2)3]8	0	10699.25844	Not in OntoMOPs KG
(4-pyramidal)x6(3-planar)x8___(Oh)	[Zn4C40H44O12S4]6[(C6H3)((C6H4)2)3(CO2)3]8	0	11949.03732	Not in OntoMOPs KG
(4-pyramidal)x6(3-planar)x8___(Oh)	[Zn4C40H44O12S4]6[(C5NH3)2(CO2)2]8	8	8577.012424	Not in OntoMOPs KG
(4-pyramidal)x6(3-planar)x8___(Oh)	[Co4C56H76O12S4]6[(C3N3)(C6H4)3(CO2)3]8	-24	10566.49051	Not in OntoMOPs KG
(4-pyramidal)x6(3-planar)x8___(Oh)	[Co4C56H76O12S4]6[(C6H3)(C6H4)3(CO2)3]8	-24	10518.58651	Not in OntoMOPs KG
(4-pyramidal)x6(3-planar)x8___(Oh)	[Co4C56H76O12S4]6[(C6H3)(CO2)3]8	-24	8692.294032	Not in OntoMOPs KG
(4-pyramidal)x6(3-planar)x8___(Oh)	[Co4C56H76O12S4]6[(C6H3)(C2C6H4)3(CO2)3]8	-24	11095.10011	Not in OntoMOPs KG
(4-pyramidal)x6(3-planar)x8___(Oh)	[Co4C56H76O12S4]6[(C6H3)((C6H4)2)3(CO2)3]8	-24	12344.87899	Not in OntoMOPs KG
(4-pyramidal)x6(3-planar)x8___(Oh)	[Co4C56H76O12S4]6[(C5NH3)2(CO2)2]8	-16	8972.854096	Not in OntoMOPs KG
(4-pyramidal)x6(3-planar)x8___(Oh)	[Mg4C56H76O12S4]6[(C3N3)(C6H4)3(CO2)3]8	0	10531.1394	Not in OntoMOPs KG
(4-pyramidal)x6(3-planar)x8___(Oh)	[Mg4C56H76O12S4]6[(C6H3)(C6H4)3(CO2)3]8	0	10483.2354	Not in OntoMOPs KG
(4-pyramidal)x6(3-planar)x8___(Oh)	[Mg4C56H76O12S4]6[(C6H3)(CO2)3]8	0	8656.94292	Not in OntoMOPs KG
(4-pyramidal)x6(3-planar)x8___(Oh)	[Mg4C56H76O12S4]6[(C6H3)(C2C6H4)3(CO2)3]8	0	11059.749	Not in OntoMOPs KG
(4-pyramidal)x6(3-planar)x8___(Oh)	[Mg4C56H76O12S4]6[(C6H3)((C6H4)2)3(CO2)3]8	0	12309.52788	Not in OntoMOPs KG
(4-pyramidal)x6(3-planar)x8___(Oh)	[Mg4C56H76O12S4]6[(C5NH3)2(CO2)2]8	8	8937.502984	Not in OntoMOPs KG
(4-pyramidal)x6(3-planar)x8___(Oh)	[Ni4C56H76O12S4]6[(C3N3)(C6H4)3(CO2)3]8	0	11356.461	Not in OntoMOPs KG
(4-pyramidal)x6(3-planar)x8___(Oh)	[Ni4C56H76O12S4]6[(C6H3)(C6H4)3(CO2)3]8	0	11308.557	Not in OntoMOPs KG
(4-pyramidal)x6(3-planar)x8___(Oh)	[Ni4C56H76O12S4]6[(C6H3)(CO2)3]8	0	9482.26452	Not in OntoMOPs KG
(4-pyramidal)x6(3-planar)x8___(Oh)	[Ni4C56H76O12S4]6[(C6H3)(C2C6H4)3(CO2)3]8	0	11885.0706	Not in OntoMOPs KG
(4-pyramidal)x6(3-planar)x8___(Oh)	[Ni4C56H76O12S4]6[(C6H3)((C6H4)2)3(CO2)3]8	0	13134.84948	Not in OntoMOPs KG
(4-pyramidal)x6(3-planar)x8___(Oh)	[Ni4C56H76O12S4]6[(C5NH3)2(CO2)2]8	8	9762.824584	Not in OntoMOPs KG
(3-pyramidal)x8(2-bent)x12___(Cs)	[Zr3O(OH)3(C5H5)3]8[(C5H5)(CH3)3(CO2)2]12	8	6666.34624	10.1002/chem.201700798
(3-pyramidal)x8(2-bent)x12___(Cs)	[V3O2(OH)2(HCO2)3]8[(C5H5)(CH3)3(CO2)2]12	0	5209.700288	Not in OntoMOPs KG
(3-pyramidal)x8(2-bent)x12___(Cs)	[Fe3O(SO4)3(C5H5N)3]8[(C5H5)(CH3)3(CO2)2]12	8	8050.746472	Not in OntoMOPs KG
(3-pyramidal)x8(2-bent)x12___(Cs)	[V6O6(OCH3)9(SO4)]8[(C5H5)(CH3)3(CO2)2]12	-16	8594.67132	Not in OntoMOPs KG
(3-pyramidal)x8(2-bent)x12___(Cs)	[V7O10(OCH3)9]8[(C5H5)(CH3)3(CO2)2]12	-24	8745.68332	Not in OntoMOPs KG
(3-pyramidal)x8(2-bent)x12___(Cs)	[Pd3PO(C3H7N)3]8[(C5H5)(CH3)3(CO2)2]12	0	6678.687976	Not in OntoMOPs KG
(4-planar)x12(2-bent)x24___(D3h)	[Rh2]12[(C6H4)(CO2)2]24	0	6408.489216	Not in OntoMOPs KG

(4-planar)x12(2-bent)x24___(D3h)	[Rh2]12[(C6H3OH)(CO2)2]24	0	6792.477	Not in OntoMOPs KG
(4-planar)x12(2-bent)x24___(D3h)	[Rh2]12[(C6H3(NO2))(CO2)2]24	0	7488.437664	Not in OntoMOPs KG
(4-planar)x12(2-bent)x24___(D3h)	[Rh2]12[(C6H3(CH3))(CO2)2]24	0	6745.121856	Not in OntoMOPs KG
(4-planar)x12(2-bent)x24___(D3h)	[Rh2]12[(C6H3Br)(CO2)2]24	0	8301.997296	Not in OntoMOPs KG
(4-planar)x12(2-bent)x24___(D3h)	[Rh2]12[(C6H3)((C3H6O2)(C9H5O2))(CO2)2]24	0	11645.40115	Not in OntoMOPs KG
(4-planar)x12(2-bent)x24___(D3h)	[Rh2]12[(C6H3)((C6H12O2)(C9H5O2))(CO2)2]24	0	12655.29907	Not in OntoMOPs KG
(4-planar)x12(2-bent)x24___(D3h)	[Rh2]12[(C6H3)(OC2H4)3(OCH3)(CO2)2]24	0	10300.86883	Not in OntoMOPs KG
(4-planar)x12(2-bent)x24___(D3h)	[Rh2]12[(C6H3)(OCH2C2H)(CO2)2]24	0	7705.62324	Not in OntoMOPs KG
(4-planar)x12(2-bent)x24___(D3h)	[Rh2]12[(C6H3NH2)(CO2)2]24	0	6768.837936	Not in OntoMOPs KG
(4-planar)x12(2-bent)x24___(D3h)	[Rh2]12[(C6H3)(OCH2CH2CH3)(CO2)2]24	0	7802.37492	Not in OntoMOPs KG
(4-planar)x12(2-bent)x24___(D3h)	[Rh2]12[(C10H6)(CO2)2]24	0	7609.892256	Not in OntoMOPs KG
(4-planar)x12(2-bent)x24___(D3h)	[Rh2]12[(C6H3)2(CO2)2]24	-24	7440.533664	Not in OntoMOPs KG
(4-planar)x12(2-bent)x24___(D3h)	[Rh2]12[(C6H3)(OCH2CH3)(CO2)2]24	0	7465.74228	Not in OntoMOPs KG
(4-planar)x12(2-bent)x24___(D3h)	[Rh2]12[(C6H3)(OC2H4)2(OH)(CO2)2]24	0	8906.983128	Not in OntoMOPs KG
(4-planar)x12(2-bent)x24___(D3h)	[Rh2]12[(C6H3)(OC2H4)3(OH)(CO2)2]24	0	9964.236192	Not in OntoMOPs KG
(4-planar)x12(2-bent)x24___(D3h)	[Rh2]12[(C6H3)O(CH2)11CH3(CO2)2]24	0	10832.06868	Not in OntoMOPs KG
(4-planar)x12(2-bent)x24___(D3h)	[Rh2]12[(C6H3)O(CH2)4CH3(CO2)2]24	0	8475.6402	Not in OntoMOPs KG
(4-planar)x12(2-bent)x24___(D3h)	[Rh2]12[(C6H3)(OC2H4OH)(CO2)2]24	0	7849.730064	Not in OntoMOPs KG
(4-planar)x12(2-bent)x24___(D3h)	[Rh2]12[(C6H3)NHCOC(CH3)3(CO2)2]24	0	8787.61308	Not in OntoMOPs KG
(4-planar)x12(2-bent)x24___(D3h)	[Rh2]12[(C6H3)CH2CS2(C6H5)(CO2)2]24	0	10398.79114	Not in OntoMOPs KG
(4-planar)x12(2-bent)x24___(D3h)	[Rh2]12[C6H3CH2CS3C4H9(CO2)2]24	0	10688.58922	Not in OntoMOPs KG
(4-planar)x12(2-bent)x24___(D3h)	[Rh2]12[C6H3C2Si(C3H7)3(CO2)2]24	0	10737.12442	Not in OntoMOPs KG
(4-planar)x12(2-bent)x24___(D3h)	[Rh2]12[(C6H3)(CH2)(C4N2H2O2CH3)(CO2)2]24	0	9723.454704	Not in OntoMOPs KG
(4-planar)x12(2-bent)x24___(D3h)	[Rh2]12[(C6H3)((C2H4O2)(C9H5O2))(CO2)2]24	0	11308.76851	Not in OntoMOPs KG
(4-planar)x12(2-bent)x24___(D3h)	[Rh2]12[(C6H3)(N2(C6H4CH3))(CO2)2]24	0	9243.735936	Not in OntoMOPs KG
(4-planar)x12(2-bent)x24___(D3h)	[Rh2]12[((C6H3)C(CH3)3)(CO2)2]24	0	7755.019776	Not in OntoMOPs KG
(4-planar)x12(2-bent)x24___(D3h)	[Rh2]12[(C6H3SO3)(CO2)2]24	0	8305.824648	Not in OntoMOPs KG
(4-planar)x12(2-bent)x24___(D3h)	[Rh2]12[(C6H3)O(CH2)13CH3(CO2)2]24	0	11505.33396	Not in OntoMOPs KG
(4-planar)x12(2-bent)x24___(D3h)	[Rh2]12[C6H3CO2C2H4Si(CH3)3(CO2)2]24	0	9870.312624	Not in OntoMOPs KG
(4-planar)x12(2-bent)x24___(D3h)	[Rh2]12[(C6H3)(CO2)3]24	-24	7440.533664	Not in OntoMOPs KG
(4-planar)x12(2-bent)x24___(D3h)	[Rh2]12[C6H3CO2CH3(CO2)2]24	0	7801.354224	Not in OntoMOPs KG
(4-planar)x12(2-bent)x24___(D3h)	[Rh2]12[C6H3OCOCHCH2(CO2)2]24	0	8089.611024	Not in OntoMOPs KG
(4-planar)x12(2-bent)x24___(D3h)	[Rh2]12[C6H3NHCO2C(CH3)3(CO2)2]24	0	9171.600864	Not in OntoMOPs KG
(4-planar)x12(2-bent)x24___(D3h)	[Rh2]12[(C6H2O)(CH2CHCH2)(CO2)2]24	0	7753.99908	Not in OntoMOPs KG
(4-planar)x12(2-bent)x24___(D3h)	[Rh2]12[(C6H3)O(CH2)3CH3(CO2)2]24	0	8139.00756	Not in OntoMOPs KG
(4-planar)x12(2-bent)x24___(D3h)	[Rh2]12[(C6H3)C6H2(OC16H33)3(CO2)2]24	0	25496.73593	Not in OntoMOPs KG
(4-planar)x12(2-bent)x24___(D3h)	[Rh2]12[C6H3N2C6H3(CH3)2(CO2)2]24	0	9580.368576	Not in OntoMOPs KG
(4-planar)x12(2-bent)x24___(D3h)	[Rh2]12[(C6H3)(C2H4O2)(CONHC6H5)(CO2)2]24	0	10708.61585	Not in OntoMOPs KG
(4-planar)x12(2-bent)x24___(D3h)	[Rh2]12[(C6H3)(OCH3)(CO2)2]24	0	7129.10964	Not in OntoMOPs KG
(4-planar)x12(2-bent)x24___(D3h)	[Ru2]12[(C6H4)(CO2)2]24	0	6364.437216	Not in OntoMOPs KG
(4-planar)x12(2-bent)x24___(D3h)	[Ru2]12[(C6H3OH)(CO2)2]24	0	6748.425	Not in OntoMOPs KG
(4-planar)x12(2-bent)x24___(D3h)	[Ru2]12[(C6H3(NO2))(CO2)2]24	0	7444.385664	Not in OntoMOPs KG
(4-planar)x12(2-bent)x24___(D3h)	[Ru2]12[(C6H3(CH3))(CO2)2]24	0	6701.069856	Not in OntoMOPs KG
(4-planar)x12(2-bent)x24___(D3h)	[Ru2]12[(C6H3Br)(CO2)2]24	0	8257.945296	Not in OntoMOPs KG
(4-planar)x12(2-bent)x24___(D3h)	[Ru2]12[(C6H3)((C3H6O2)(C9H5O2))(CO2)2]24	0	11601.34915	Not in OntoMOPs KG
(4-planar)x12(2-bent)x24___(D3h)	[Ru2]12[(C6H3)((C6H12O2)(C9H5O2))(CO2)2]24	0	12611.24707	Not in OntoMOPs KG
(4-planar)x12(2-bent)x24___(D3h)	[Ru2]12[(C6H3)(OC2H4)3(OCH3)(CO2)2]24	0	10256.81683	Not in OntoMOPs KG
(4-planar)x12(2-bent)x24___(D3h)	[Ru2]12[(C6H3)(OCH2C2H)(CO2)2]24	0	7661.57124	Not in OntoMOPs KG
(4-planar)x12(2-bent)x24___(D3h)	[Ru2]12[(C6H3NH2)(CO2)2]24	0	6724.785936	Not in OntoMOPs KG
(4-planar)x12(2-bent)x24___(D3h)	[Ru2]12[(C6H3)(OCH2CH2CH3)(CO2)2]24	0	7758.32292	Not in OntoMOPs KG

(4-planar)x12(2-bent)x24___(D3h)	[Ru2]12[(C10H6)(CO2)2]24	0	7565.840256	Not in OntoMOPs KG
(4-planar)x12(2-bent)x24___(D3h)	[Ru2]12[(C6H3)2(CO2)2]24	-24	7396.481664	Not in OntoMOPs KG
(4-planar)x12(2-bent)x24___(D3h)	[Ru2]12[(C6H3)(OCH2CH3)(CO2)2]24	0	7421.69028	Not in OntoMOPs KG
(4-planar)x12(2-bent)x24___(D3h)	[Ru2]12[(C6H3)(OC2H4)2(OH)(CO2)2]24	0	8862.931128	Not in OntoMOPs KG
(4-planar)x12(2-bent)x24___(D3h)	[Ru2]12[(C6H3)(OC2H4)3(OH)(CO2)2]24	0	9920.184192	Not in OntoMOPs KG
(4-planar)x12(2-bent)x24___(D3h)	[Ru2]12[(C6H3)O(CH2)11CH3(CO2)2]24	0	10788.01668	Not in OntoMOPs KG
(4-planar)x12(2-bent)x24___(D3h)	[Ru2]12[(C6H3)O(CH2)4CH3(CO2)2]24	0	8431.5882	Not in OntoMOPs KG
(4-planar)x12(2-bent)x24___(D3h)	[Ru2]12[(C6H3)(OC2H4OH)(CO2)2]24	0	7805.678064	Not in OntoMOPs KG
(4-planar)x12(2-bent)x24___(D3h)	[Ru2]12[(C6H3)NHCOC(CH3)3(CO2)2]24	0	8743.56108	Not in OntoMOPs KG
(4-planar)x12(2-bent)x24___(D3h)	[Ru2]12[(C6H3)CH2CS2(C6H5)(CO2)2]24	0	10354.73914	Not in OntoMOPs KG
(4-planar)x12(2-bent)x24___(D3h)	[Ru2]12[C6H3CH2CS3C4H9(CO2)2]24	0	10644.53722	Not in OntoMOPs KG
(4-planar)x12(2-bent)x24___(D3h)	[Ru2]12[C6H3C2Si(C3H7)3(CO2)2]24	0	10693.07242	Not in OntoMOPs KG
(4-planar)x12(2-bent)x24___(D3h)	[Ru2]12[(C6H3)(CH2)(C4N2H2O2CH3)(CO2)2]24	0	9679.402704	Not in OntoMOPs KG
(4-planar)x12(2-bent)x24___(D3h)	[Ru2]12[(C6H3)((C2H4O2)(C9H5O2))(CO2)2]24	0	11264.71651	Not in OntoMOPs KG
(4-planar)x12(2-bent)x24___(D3h)	[Ru2]12[(C6H3)(N2(C6H4CH3))(CO2)2]24	0	9199.683936	Not in OntoMOPs KG
(4-planar)x12(2-bent)x24___(D3h)	[Ru2]12[((C6H3)C(CH3)3)(CO2)2]24	0	7710.967776	Not in OntoMOPs KG
(4-planar)x12(2-bent)x24___(D3h)	[Ru2]12[(C6H3SO3)(CO2)2]24	0	8261.772648	Not in OntoMOPs KG
(4-planar)x12(2-bent)x24___(D3h)	[Ru2]12[(C6H3)O(CH2)13CH3(CO2)2]24	0	11461.28196	Not in OntoMOPs KG
(4-planar)x12(2-bent)x24___(D3h)	[Ru2]12[C6H3CO2C2H4Si(CH3)3(CO2)2]24	0	9826.260624	Not in OntoMOPs KG
(4-planar)x12(2-bent)x24___(D3h)	[Ru2]12[(C6H3)(CO2)3]24	-24	7396.481664	Not in OntoMOPs KG
(4-planar)x12(2-bent)x24___(D3h)	[Ru2]12[C6H3CO2CH3(CO2)2]24	0	7757.302224	Not in OntoMOPs KG
(4-planar)x12(2-bent)x24___(D3h)	[Ru2]12[C6H3OCOCHCH2(CO2)2]24	0	8045.559024	Not in OntoMOPs KG
(4-planar)x12(2-bent)x24___(D3h)	[Ru2]12[C6H3NHCO2C(CH3)3(CO2)2]24	0	9127.548864	Not in OntoMOPs KG
(4-planar)x12(2-bent)x24___(D3h)	[Ru2]12[(C6H2O)(CH2CHCH2)(CO2)2]24	0	7709.94708	Not in OntoMOPs KG
(4-planar)x12(2-bent)x24___(D3h)	[Ru2]12[(C6H3)O(CH2)3CH3(CO2)2]24	0	8094.95556	Not in OntoMOPs KG
(4-planar)x12(2-bent)x24___(D3h)	[Ru2]12[(C6H3)C6H2(OC16H33)3(CO2)2]24	0	25452.68393	Not in OntoMOPs KG
(4-planar)x12(2-bent)x24___(D3h)	[Ru2]12[C6H3N2C6H3(CH3)2(CO2)2]24	0	9536.316576	Not in OntoMOPs KG
(4-planar)x12(2-bent)x24___(D3h)	[Ru2]12[(C6H3)(C2H4O2)(CONHC6H5)(CO2)2]24	0	10664.56385	Not in OntoMOPs KG
(4-planar)x12(2-bent)x24___(D3h)	[Ru2]12[(C6H3)(OCH3)(CO2)2]24	0	7085.05764	Not in OntoMOPs KG
(4-planar)x12(2-bent)x24___(D3h)	[Ni2]12[(C6H4)(CO2)2]24	0	5347.398816	Not in OntoMOPs KG
(4-planar)x12(2-bent)x24___(D3h)	[Ni2]12[(C6H3OH)(CO2)2]24	0	5731.3866	Not in OntoMOPs KG
(4-planar)x12(2-bent)x24___(D3h)	[Ni2]12[(C6H3(NO2))(CO2)2]24	0	6427.347264	Not in OntoMOPs KG
(4-planar)x12(2-bent)x24___(D3h)	[Ni2]12[(C6H3(CH3))(CO2)2]24	0	5684.031456	Not in OntoMOPs KG
(4-planar)x12(2-bent)x24___(D3h)	[Ni2]12[(C6H3Br)(CO2)2]24	0	7240.906896	Not in OntoMOPs KG
(4-planar)x12(2-bent)x24___(D3h)	[Ni2]12[(C6H3)((C3H6O2)(C9H5O2))(CO2)2]24	0	10584.31075	Not in OntoMOPs KG
(4-planar)x12(2-bent)x24___(D3h)	[Ni2]12[(C6H3)((C6H12O2)(C9H5O2))(CO2)2]24	0	11594.20867	Not in OntoMOPs KG
(4-planar)x12(2-bent)x24___(D3h)	[Ni2]12[(C6H3)(OC2H4)3(OCH3)(CO2)2]24	0	9239.778432	Not in OntoMOPs KG
(4-planar)x12(2-bent)x24___(D3h)	[Ni2]12[(C6H3)(OCH2C2H)(CO2)2]24	0	6644.53284	Not in OntoMOPs KG
(4-planar)x12(2-bent)x24___(D3h)	[Ni2]12[(C6H3NH2)(CO2)2]24	0	5707.747536	Not in OntoMOPs KG
(4-planar)x12(2-bent)x24___(D3h)	[Ni2]12[(C6H3)(OCH2CH2CH3)(CO2)2]24	0	6741.28452	Not in OntoMOPs KG
(4-planar)x12(2-bent)x24___(D3h)	[Ni2]12[(C10H6)(CO2)2]24	0	6548.801856	Not in OntoMOPs KG
(4-planar)x12(2-bent)x24___(D3h)	[Ni2]12[(C6H3)2(CO2)2]24	-24	6379.443264	Not in OntoMOPs KG
(4-planar)x12(2-bent)x24___(D3h)	[Ni2]12[(C6H3)(OCH2CH3)(CO2)2]24	0	6404.65188	Not in OntoMOPs KG
(4-planar)x12(2-bent)x24___(D3h)	[Ni2]12[(C6H3)(OC2H4)2(OH)(CO2)2]24	0	7845.892728	Not in OntoMOPs KG
(4-planar)x12(2-bent)x24___(D3h)	[Ni2]12[(C6H3)(OC2H4)3(OH)(CO2)2]24	0	8903.145792	Not in OntoMOPs KG
(4-planar)x12(2-bent)x24___(D3h)	[Ni2]12[(C6H3)O(CH2)11CH3(CO2)2]24	0	9770.97828	Not in OntoMOPs KG
(4-planar)x12(2-bent)x24___(D3h)	[Ni2]12[(C6H3)O(CH2)4CH3(CO2)2]24	0	7414.5498	Not in OntoMOPs KG
(4-planar)x12(2-bent)x24___(D3h)	[Ni2]12[(C6H3)(OC2H4OH)(CO2)2]24	0	6788.639664	Not in OntoMOPs KG
(4-planar)x12(2-bent)x24___(D3h)	[Ni2]12[(C6H3)NHCOC(CH3)3(CO2)2]24	0	7726.52268	Not in OntoMOPs KG
(4-planar)x12(2-bent)x24___(D3h)	[Ni2]12[(C6H3)CH2CS2(C6H5)(CO2)2]24	0	9337.700736	Not in OntoMOPs KG

(4-planar)x12(2-bent)x24___(D3h)	[Ni2]12[C6H3CH2CS3C4H9(CO2)2]24	0	9627.498816	Not in OntoMOPs KG
(4-planar)x12(2-bent)x24___(D3h)	[Ni2]12[C6H3C2Si(C3H7)3(CO2)2]24	0	9676.034016	Not in OntoMOPs KG
(4-planar)x12(2-bent)x24___(D3h)	[Ni2]12[(C6H3)(CH2)(C4N2H2O2CH3)(CO2)2]24	0	8662.364304	Not in OntoMOPs KG
(4-planar)x12(2-bent)x24___(D3h)	[Ni2]12[(C6H3)((C2H4O2)(C9H5O2))(CO2)2]24	0	10247.67811	Not in OntoMOPs KG
(4-planar)x12(2-bent)x24___(D3h)	[Ni2]12[(C6H3)(N2(C6H4CH3))(CO2)2]24	0	8182.645536	Not in OntoMOPs KG
(4-planar)x12(2-bent)x24___(D3h)	[Ni2]12[((C6H3)C(CH3)3)(CO2)2]24	0	6693.929376	Not in OntoMOPs KG
(4-planar)x12(2-bent)x24___(D3h)	[Ni2]12[(C6H3SO3)(CO2)2]24	0	7244.734248	Not in OntoMOPs KG
(4-planar)x12(2-bent)x24___(D3h)	[Ni2]12[(C6H3)O(CH2)13CH3(CO2)2]24	0	10444.24356	Not in OntoMOPs KG
(4-planar)x12(2-bent)x24___(D3h)	[Ni2]12[C6H3CO2C2H4Si(CH3)3(CO2)2]24	0	8809.222224	Not in OntoMOPs KG
(4-planar)x12(2-bent)x24___(D3h)	[Ni2]12[(C6H3)(CO2)3]24	-24	6379.443264	Not in OntoMOPs KG
(4-planar)x12(2-bent)x24___(D3h)	[Ni2]12[C6H3CO2CH3(CO2)2]24	0	6740.263824	Not in OntoMOPs KG
(4-planar)x12(2-bent)x24___(D3h)	[Ni2]12[C6H3OCOCHCH2(CO2)2]24	0	7028.520624	Not in OntoMOPs KG
(4-planar)x12(2-bent)x24___(D3h)	[Ni2]12[C6H3NHCO2C(CH3)3(CO2)2]24	0	8110.510464	Not in OntoMOPs KG
(4-planar)x12(2-bent)x24___(D3h)	[Ni2]12[(C6H2O)(CH2CHCH2)(CO2)2]24	0	6692.90868	Not in OntoMOPs KG
(4-planar)x12(2-bent)x24___(D3h)	[Ni2]12[(C6H3)O(CH2)3CH3(CO2)2]24	0	7077.91716	Not in OntoMOPs KG
(4-planar)x12(2-bent)x24___(D3h)	[Ni2]12[(C6H3)C6H2(OC16H33)3(CO2)2]24	0	24435.64553	Not in OntoMOPs KG
(4-planar)x12(2-bent)x24___(D3h)	[Ni2]12[C6H3N2C6H3(CH3)2(CO2)2]24	0	8519.278176	Not in OntoMOPs KG
(4-planar)x12(2-bent)x24___(D3h)	[Ni2]12[(C6H3)(C2H4O2)(CONHC6H5)(CO2)2]24	0	9647.525448	Not in OntoMOPs KG
(4-planar)x12(2-bent)x24___(D3h)	[Ni2]12[(C6H3)(OCH3)(CO2)2]24	0	6068.01924	Not in OntoMOPs KG
(4-planar)x12(2-bent)x24___(D3h)	[Cu2]12[(C6H4)(CO2)2]24	0	5463.861216	Not in OntoMOPs KG
(4-planar)x12(2-bent)x24___(D3h)	[Cu2]12[(C6H3OH)(CO2)2]24	0	5847.849	Not in OntoMOPs KG
(4-planar)x12(2-bent)x24___(D3h)	[Cu2]12[(C6H3(NO2))(CO2)2]24	0	6543.809664	Not in OntoMOPs KG
(4-planar)x12(2-bent)x24___(D3h)	[Cu2]12[(C6H3(CH3))(CO2)2]24	0	5800.493856	Not in OntoMOPs KG
(4-planar)x12(2-bent)x24___(D3h)	[Cu2]12[(C6H3Br)(CO2)2]24	0	7357.369296	Not in OntoMOPs KG
(4-planar)x12(2-bent)x24___(D3h)	[Cu2]12[(C6H3)((C3H6O2)(C9H5O2))(CO2)2]24	0	10700.77315	Not in OntoMOPs KG
(4-planar)x12(2-bent)x24___(D3h)	[Cu2]12[(C6H3)((C6H12O2)(C9H5O2))(CO2)2]24	0	11710.67107	Not in OntoMOPs KG
(4-planar)x12(2-bent)x24___(D3h)	[Cu2]12[(C6H3)(OC2H4)3(OCH3)(CO2)2]24	0	9356.240832	Not in OntoMOPs KG
(4-planar)x12(2-bent)x24___(D3h)	[Cu2]12[(C6H3)(OCH2C2H)(CO2)2]24	0	6760.99524	Not in OntoMOPs KG
(4-planar)x12(2-bent)x24___(D3h)	[Cu2]12[(C6H3NH2)(CO2)2]24	0	5824.209936	Not in OntoMOPs KG
(4-planar)x12(2-bent)x24___(D3h)	[Cu2]12[(C6H3)(OCH2CH2CH3)(CO2)2]24	0	6857.74692	Not in OntoMOPs KG
(4-planar)x12(2-bent)x24___(D3h)	[Cu2]12[(C10H6)(CO2)2]24	0	6665.264256	Not in OntoMOPs KG
(4-planar)x12(2-bent)x24___(D3h)	[Cu2]12[(C6H3)2(CO2)2]24	-24	6495.905664	Not in OntoMOPs KG
(4-planar)x12(2-bent)x24___(D3h)	[Cu2]12[(C6H3)(OCH2CH3)(CO2)2]24	0	6521.11428	Not in OntoMOPs KG
(4-planar)x12(2-bent)x24___(D3h)	[Cu2]12[(C6H3)(OC2H4)2(OH)(CO2)2]24	0	7962.355128	Not in OntoMOPs KG
(4-planar)x12(2-bent)x24___(D3h)	[Cu2]12[(C6H3)(OC2H4)3(OH)(CO2)2]24	0	9019.608192	Not in OntoMOPs KG
(4-planar)x12(2-bent)x24___(D3h)	[Cu2]12[(C6H3)O(CH2)11CH3(CO2)2]24	0	9887.44068	Not in OntoMOPs KG
(4-planar)x12(2-bent)x24___(D3h)	[Cu2]12[(C6H3)O(CH2)4CH3(CO2)2]24	0	7531.0122	Not in OntoMOPs KG
(4-planar)x12(2-bent)x24___(D3h)	[Cu2]12[(C6H3)(OC2H4OH)(CO2)2]24	0	6905.102064	Not in OntoMOPs KG
(4-planar)x12(2-bent)x24___(D3h)	[Cu2]12[(C6H3)NHCOC(CH3)3(CO2)2]24	0	7842.98508	Not in OntoMOPs KG
(4-planar)x12(2-bent)x24___(D3h)	[Cu2]12[(C6H3)CH2CS2(C6H5)(CO2)2]24	0	9454.163136	Not in OntoMOPs KG
(4-planar)x12(2-bent)x24___(D3h)	[Cu2]12[C6H3CH2CS3C4H9(CO2)2]24	0	9743.961216	Not in OntoMOPs KG
(4-planar)x12(2-bent)x24___(D3h)	[Cu2]12[C6H3C2Si(C3H7)3(CO2)2]24	0	9792.496416	Not in OntoMOPs KG
(4-planar)x12(2-bent)x24___(D3h)	[Cu2]12[(C6H3)(CH2)(C4N2H2O2CH3)(CO2)2]24	0	8778.826704	Not in OntoMOPs KG
(4-planar)x12(2-bent)x24___(D3h)	[Cu2]12[(C6H3)((C2H4O2)(C9H5O2))(CO2)2]24	0	10364.14051	Not in OntoMOPs KG
(4-planar)x12(2-bent)x24___(D3h)	[Cu2]12[(C6H3)(N2(C6H4CH3))(CO2)2]24	0	8299.107936	Not in OntoMOPs KG
(4-planar)x12(2-bent)x24___(D3h)	[Cu2]12[((C6H3)C(CH3)3)(CO2)2]24	0	6810.391776	Not in OntoMOPs KG
(4-planar)x12(2-bent)x24___(D3h)	[Cu2]12[(C6H3SO3)(CO2)2]24	0	7361.196648	Not in OntoMOPs KG
(4-planar)x12(2-bent)x24___(D3h)	[Cu2]12[(C6H3)O(CH2)13CH3(CO2)2]24	0	10560.70596	Not in OntoMOPs KG
(4-planar)x12(2-bent)x24___(D3h)	[Cu2]12[C6H3CO2C2H4Si(CH3)3(CO2)2]24	0	8925.684624	Not in OntoMOPs KG
(4-planar)x12(2-bent)x24___(D3h)	[Cu2]12[(C6H3)(CO2)3]24	-24	6495.905664	Not in OntoMOPs KG

(4-planar)x12(2-bent)x24___(D3h)	[Cu2]12[C6H3CO2CH3(CO2)2]24	0	6856.726224	Not in OntoMOPs KG
(4-planar)x12(2-bent)x24___(D3h)	[Cu2]12[C6H3OCOCHCH2(CO2)2]24	0	7144.983024	Not in OntoMOPs KG
(4-planar)x12(2-bent)x24___(D3h)	[Cu2]12[C6H3NHCO2C(CH3)3(CO2)2]24	0	8226.972864	Not in OntoMOPs KG
(4-planar)x12(2-bent)x24___(D3h)	[Cu2]12[(C6H2O)(CH2CHCH2)(CO2)2]24	0	6809.37108	Not in OntoMOPs KG
(4-planar)x12(2-bent)x24___(D3h)	[Cu2]12[(C6H3)O(CH2)3CH3(CO2)2]24	0	7194.37956	Not in OntoMOPs KG
(4-planar)x12(2-bent)x24___(D3h)	[Cu2]12[(C6H3)C6H2(OC16H33)3(CO2)2]24	0	24552.10793	Not in OntoMOPs KG
(4-planar)x12(2-bent)x24___(D3h)	[Cu2]12[C6H3N2C6H3(CH3)2(CO2)2]24	0	8635.740576	Not in OntoMOPs KG
(4-planar)x12(2-bent)x24___(D3h)	[Cu2]12[(C6H3)(C2H4O2)(CONHC6H5)(CO2)2]24	0	9763.987848	Not in OntoMOPs KG
(4-planar)x12(2-bent)x24___(D3h)	[Cu2]12[(C6H3)(OCH3)(CO2)2]24	0	6184.48164	10.1021/cg034199d
(4-planar)x12(2-bent)x24___(D3h)	[Fe2]12[(C6H4)(CO2)2]24	0	5279.037216	Not in OntoMOPs KG
(4-planar)x12(2-bent)x24___(D3h)	[Fe2]12[(C6H3OH)(CO2)2]24	0	5663.025	Not in OntoMOPs KG
(4-planar)x12(2-bent)x24___(D3h)	[Fe2]12[(C6H3(NO2))(CO2)2]24	0	6358.985664	Not in OntoMOPs KG
(4-planar)x12(2-bent)x24___(D3h)	[Fe2]12[(C6H3(CH3))(CO2)2]24	0	5615.669856	Not in OntoMOPs KG
(4-planar)x12(2-bent)x24___(D3h)	[Fe2]12[(C6H3Br)(CO2)2]24	0	7172.545296	Not in OntoMOPs KG
(4-planar)x12(2-bent)x24___(D3h)	[Fe2]12[(C6H3)((C3H6O2)(C9H5O2))(CO2)2]24	0	10515.94915	Not in OntoMOPs KG
(4-planar)x12(2-bent)x24___(D3h)	[Fe2]12[(C6H3)((C6H12O2)(C9H5O2))(CO2)2]24	0	11525.84707	Not in OntoMOPs KG
(4-planar)x12(2-bent)x24___(D3h)	[Fe2]12[(C6H3)(OC2H4)3(OCH3)(CO2)2]24	0	9171.416832	Not in OntoMOPs KG
(4-planar)x12(2-bent)x24___(D3h)	[Fe2]12[(C6H3)(OCH2C2H)(CO2)2]24	0	6576.17124	Not in OntoMOPs KG
(4-planar)x12(2-bent)x24___(D3h)	[Fe2]12[(C6H3NH2)(CO2)2]24	0	5639.385936	Not in OntoMOPs KG
(4-planar)x12(2-bent)x24___(D3h)	[Fe2]12[(C6H3)(OCH2CH2CH3)(CO2)2]24	0	6672.92292	Not in OntoMOPs KG
(4-planar)x12(2-bent)x24___(D3h)	[Fe2]12[(C10H6)(CO2)2]24	0	6480.440256	Not in OntoMOPs KG
(4-planar)x12(2-bent)x24___(D3h)	[Fe2]12[(C6H3)2(CO2)2]24	-24	6311.081664	Not in OntoMOPs KG
(4-planar)x12(2-bent)x24___(D3h)	[Fe2]12[(C6H3)(OCH2CH3)(CO2)2]24	0	6336.29028	Not in OntoMOPs KG
(4-planar)x12(2-bent)x24___(D3h)	[Fe2]12[(C6H3)(OC2H4)2(OH)(CO2)2]24	0	7777.531128	Not in OntoMOPs KG
(4-planar)x12(2-bent)x24___(D3h)	[Fe2]12[(C6H3)(OC2H4)3(OH)(CO2)2]24	0	8834.784192	Not in OntoMOPs KG
(4-planar)x12(2-bent)x24___(D3h)	[Fe2]12[(C6H3)O(CH2)11CH3(CO2)2]24	0	9702.61668	Not in OntoMOPs KG
(4-planar)x12(2-bent)x24___(D3h)	[Fe2]12[(C6H3)O(CH2)4CH3(CO2)2]24	0	7346.1882	Not in OntoMOPs KG
(4-planar)x12(2-bent)x24___(D3h)	[Fe2]12[(C6H3)(OC2H4OH)(CO2)2]24	0	6720.278064	Not in OntoMOPs KG
(4-planar)x12(2-bent)x24___(D3h)	[Fe2]12[(C6H3)NHCOC(CH3)3(CO2)2]24	0	7658.16108	Not in OntoMOPs KG
(4-planar)x12(2-bent)x24___(D3h)	[Fe2]12[(C6H3)CH2CS2(C6H5)(CO2)2]24	0	9269.339136	Not in OntoMOPs KG
(4-planar)x12(2-bent)x24___(D3h)	[Fe2]12[C6H3CH2CS3C4H9(CO2)2]24	0	9559.137216	Not in OntoMOPs KG
(4-planar)x12(2-bent)x24___(D3h)	[Fe2]12[C6H3C2Si(C3H7)3(CO2)2]24	0	9607.672416	Not in OntoMOPs KG
(4-planar)x12(2-bent)x24___(D3h)	[Fe2]12[(C6H3)(CH2)(C4N2H2O2CH3)(CO2)2]24	0	8594.002704	Not in OntoMOPs KG
(4-planar)x12(2-bent)x24___(D3h)	[Fe2]12[(C6H3)((C2H4O2)(C9H5O2))(CO2)2]24	0	10179.31651	Not in OntoMOPs KG
(4-planar)x12(2-bent)x24___(D3h)	[Fe2]12[(C6H3)(N2(C6H4CH3))(CO2)2]24	0	8114.283936	Not in OntoMOPs KG
(4-planar)x12(2-bent)x24___(D3h)	[Fe2]12[((C6H3)C(CH3)3)(CO2)2]24	0	6625.567776	Not in OntoMOPs KG
(4-planar)x12(2-bent)x24___(D3h)	[Fe2]12[(C6H3SO3)(CO2)2]24	0	7176.372648	Not in OntoMOPs KG
(4-planar)x12(2-bent)x24___(D3h)	[Fe2]12[(C6H3)O(CH2)13CH3(CO2)2]24	0	10375.88196	Not in OntoMOPs KG
(4-planar)x12(2-bent)x24___(D3h)	[Fe2]12[C6H3CO2C2H4Si(CH3)3(CO2)2]24	0	8740.860624	Not in OntoMOPs KG
(4-planar)x12(2-bent)x24___(D3h)	[Fe2]12[(C6H3)(CO2)3]24	-24	6311.081664	Not in OntoMOPs KG
(4-planar)x12(2-bent)x24___(D3h)	[Fe2]12[C6H3CO2CH3(CO2)2]24	0	6671.902224	Not in OntoMOPs KG
(4-planar)x12(2-bent)x24___(D3h)	[Fe2]12[C6H3OCOCHCH2(CO2)2]24	0	6960.159024	Not in OntoMOPs KG
(4-planar)x12(2-bent)x24___(D3h)	[Fe2]12[C6H3NHCO2C(CH3)3(CO2)2]24	0	8042.148864	Not in OntoMOPs KG
(4-planar)x12(2-bent)x24___(D3h)	[Fe2]12[(C6H2O)(CH2CHCH2)(CO2)2]24	0	6624.54708	Not in OntoMOPs KG
(4-planar)x12(2-bent)x24___(D3h)	[Fe2]12[(C6H3)O(CH2)3CH3(CO2)2]24	0	7009.55556	Not in OntoMOPs KG
(4-planar)x12(2-bent)x24___(D3h)	[Fe2]12[(C6H3)C6H2(OC16H33)3(CO2)2]24	0	24367.28393	Not in OntoMOPs KG
(4-planar)x12(2-bent)x24___(D3h)	[Fe2]12[C6H3N2C6H3(CH3)2(CO2)2]24	0	8450.916576	Not in OntoMOPs KG
(4-planar)x12(2-bent)x24___(D3h)	[Fe2]12[(C6H3)(C2H4O2)(CONHC6H5)(CO2)2]24	0	9579.163848	Not in OntoMOPs KG
(4-planar)x12(2-bent)x24___(D3h)	[Fe2]12[(C6H3)(OCH3)(CO2)2]24	0	5999.65764	Not in OntoMOPs KG
(4-planar)x12(2-bent)x24___(D3h)	[Co2]12[(C6H4)(CO2)2]24	0	5353.154016	Not in OntoMOPs KG

(4-planar)x12(2-bent)x24___(D3h)	[Co2]12[(C6H3OH)(CO2)2]24	0	5737.1418	Not in OntoMOPs KG
(4-planar)x12(2-bent)x24___(D3h)	[Co2]12[(C6H3(NO2))(CO2)2]24	0	6433.102464	Not in OntoMOPs KG
(4-planar)x12(2-bent)x24___(D3h)	[Co2]12[(C6H3(CH3))(CO2)2]24	0	5689.786656	Not in OntoMOPs KG
(4-planar)x12(2-bent)x24___(D3h)	[Co2]12[(C6H3Br)(CO2)2]24	0	7246.662096	Not in OntoMOPs KG
(4-planar)x12(2-bent)x24___(D3h)	[Co2]12[(C6H3)((C3H6O2)(C9H5O2))(CO2)2]24	0	10590.06595	Not in OntoMOPs KG
(4-planar)x12(2-bent)x24___(D3h)	[Co2]12[(C6H3)((C6H12O2)(C9H5O2))(CO2)2]24	0	11599.96387	Not in OntoMOPs KG
(4-planar)x12(2-bent)x24___(D3h)	[Co2]12[(C6H3)(OC2H4)3(OCH3)(CO2)2]24	0	9245.533632	Not in OntoMOPs KG
(4-planar)x12(2-bent)x24___(D3h)	[Co2]12[(C6H3)(OCH2C2H)(CO2)2]24	0	6650.28804	Not in OntoMOPs KG
(4-planar)x12(2-bent)x24___(D3h)	[Co2]12[(C6H3NH2)(CO2)2]24	0	5713.502736	Not in OntoMOPs KG
(4-planar)x12(2-bent)x24___(D3h)	[Co2]12[(C6H3)(OCH2CH2CH3)(CO2)2]24	0	6747.03972	Not in OntoMOPs KG
(4-planar)x12(2-bent)x24___(D3h)	[Co2]12[(C10H6)(CO2)2]24	0	6554.557056	Not in OntoMOPs KG
(4-planar)x12(2-bent)x24___(D3h)	[Co2]12[(C6H3)2(CO2)2]24	-24	6385.198464	Not in OntoMOPs KG
(4-planar)x12(2-bent)x24___(D3h)	[Co2]12[(C6H3)(OCH2CH3)(CO2)2]24	0	6410.40708	Not in OntoMOPs KG
(4-planar)x12(2-bent)x24___(D3h)	[Co2]12[(C6H3)(OC2H4)2(OH)(CO2)2]24	0	7851.647928	Not in OntoMOPs KG
(4-planar)x12(2-bent)x24___(D3h)	[Co2]12[(C6H3)(OC2H4)3(OH)(CO2)2]24	0	8908.900992	Not in OntoMOPs KG
(4-planar)x12(2-bent)x24___(D3h)	[Co2]12[(C6H3)O(CH2)11CH3(CO2)2]24	0	9776.73348	Not in OntoMOPs KG
(4-planar)x12(2-bent)x24___(D3h)	[Co2]12[(C6H3)O(CH2)4CH3(CO2)2]24	0	7420.305	Not in OntoMOPs KG
(4-planar)x12(2-bent)x24___(D3h)	[Co2]12[(C6H3)(OC2H4OH)(CO2)2]24	0	6794.394864	Not in OntoMOPs KG
(4-planar)x12(2-bent)x24___(D3h)	[Co2]12[(C6H3)NHCOC(CH3)3(CO2)2]24	0	7732.27788	Not in OntoMOPs KG
(4-planar)x12(2-bent)x24___(D3h)	[Co2]12[(C6H3)CH2CS2(C6H5)(CO2)2]24	0	9343.455936	Not in OntoMOPs KG
(4-planar)x12(2-bent)x24___(D3h)	[Co2]12[C6H3CH2CS3C4H9(CO2)2]24	0	9633.254016	Not in OntoMOPs KG
(4-planar)x12(2-bent)x24___(D3h)	[Co2]12[C6H3C2Si(C3H7)3(CO2)2]24	0	9681.789216	Not in OntoMOPs KG
(4-planar)x12(2-bent)x24___(D3h)	[Co2]12[(C6H3)(CH2)(C4N2H2O2CH3)(CO2)2]24	0	8668.119504	Not in OntoMOPs KG
(4-planar)x12(2-bent)x24___(D3h)	[Co2]12[(C6H3)((C2H4O2)(C9H5O2))(CO2)2]24	0	10253.43331	Not in OntoMOPs KG
(4-planar)x12(2-bent)x24___(D3h)	[Co2]12[(C6H3)(N2(C6H4CH3))(CO2)2]24	0	8188.400736	Not in OntoMOPs KG
(4-planar)x12(2-bent)x24___(D3h)	[Co2]12[((C6H3)C(CH3)3)(CO2)2]24	0	6699.684576	Not in OntoMOPs KG
(4-planar)x12(2-bent)x24___(D3h)	[Co2]12[(C6H3SO3)(CO2)2]24	0	7250.489448	Not in OntoMOPs KG
(4-planar)x12(2-bent)x24___(D3h)	[Co2]12[(C6H3)O(CH2)13CH3(CO2)2]24	0	10449.99876	Not in OntoMOPs KG
(4-planar)x12(2-bent)x24___(D3h)	[Co2]12[C6H3CO2C2H4Si(CH3)3(CO2)2]24	0	8814.977424	Not in OntoMOPs KG
(4-planar)x12(2-bent)x24___(D3h)	[Co2]12[(C6H3)(CO2)3]24	-24	6385.198464	Not in OntoMOPs KG
(4-planar)x12(2-bent)x24___(D3h)	[Co2]12[C6H3CO2CH3(CO2)2]24	0	6746.019024	Not in OntoMOPs KG
(4-planar)x12(2-bent)x24___(D3h)	[Co2]12[C6H3OCOCHCH2(CO2)2]24	0	7034.275824	Not in OntoMOPs KG
(4-planar)x12(2-bent)x24___(D3h)	[Co2]12[C6H3NHCO2C(CH3)3(CO2)2]24	0	8116.265664	Not in OntoMOPs KG
(4-planar)x12(2-bent)x24___(D3h)	[Co2]12[(C6H2O)(CH2CHCH2)(CO2)2]24	0	6698.66388	Not in OntoMOPs KG
(4-planar)x12(2-bent)x24___(D3h)	[Co2]12[(C6H3)O(CH2)3CH3(CO2)2]24	0	7083.67236	Not in OntoMOPs KG
(4-planar)x12(2-bent)x24___(D3h)	[Co2]12[(C6H3)C6H2(OC16H33)3(CO2)2]24	0	24441.40073	Not in OntoMOPs KG
(4-planar)x12(2-bent)x24___(D3h)	[Co2]12[C6H3N2C6H3(CH3)2(CO2)2]24	0	8525.033376	Not in OntoMOPs KG
(4-planar)x12(2-bent)x24___(D3h)	[Co2]12[(C6H3)(C2H4O2)(CONHC6H5)(CO2)2]24	0	9653.280648	Not in OntoMOPs KG
(4-planar)x12(2-bent)x24___(D3h)	[Co2]12[(C6H3)(OCH3)(CO2)2]24	0	6073.77444	Not in OntoMOPs KG
(4-planar)x12(2-bent)x24___(D3h)	[Cr2]12[(C6H4)(CO2)2]24	0	5186.663616	Not in OntoMOPs KG
(4-planar)x12(2-bent)x24___(D3h)	[Cr2]12[(C6H3OH)(CO2)2]24	0	5570.6514	Not in OntoMOPs KG
(4-planar)x12(2-bent)x24___(D3h)	[Cr2]12[(C6H3(NO2))(CO2)2]24	0	6266.612064	Not in OntoMOPs KG
(4-planar)x12(2-bent)x24___(D3h)	[Cr2]12[(C6H3(CH3))(CO2)2]24	0	5523.296256	Not in OntoMOPs KG
(4-planar)x12(2-bent)x24___(D3h)	[Cr2]12[(C6H3Br)(CO2)2]24	0	7080.171696	Not in OntoMOPs KG
(4-planar)x12(2-bent)x24___(D3h)	[Cr2]12[(C6H3)((C3H6O2)(C9H5O2))(CO2)2]24	0	10423.57555	Not in OntoMOPs KG
(4-planar)x12(2-bent)x24___(D3h)	[Cr2]12[(C6H3)((C6H12O2)(C9H5O2))(CO2)2]24	0	11433.47347	Not in OntoMOPs KG
(4-planar)x12(2-bent)x24___(D3h)	[Cr2]12[(C6H3)(OC2H4)3(OCH3)(CO2)2]24	0	9079.043232	Not in OntoMOPs KG
(4-planar)x12(2-bent)x24___(D3h)	[Cr2]12[(C6H3)(OCH2C2H)(CO2)2]24	0	6483.79764	Not in OntoMOPs KG
(4-planar)x12(2-bent)x24___(D3h)	[Cr2]12[(C6H3NH2)(CO2)2]24	0	5547.012336	Not in OntoMOPs KG
(4-planar)x12(2-bent)x24___(D3h)	[Cr2]12[(C6H3)(OCH2CH2CH3)(CO2)2]24	0	6580.54932	Not in OntoMOPs KG

(4-planar)x12(2-bent)x24___(D3h)	[Cr2]12[(C10H6)(CO2)2]24	0	6388.066656	Not in OntoMOPs KG
(4-planar)x12(2-bent)x24___(D3h)	[Cr2]12[(C6H3)2(CO2)2]24	-24	6218.708064	Not in OntoMOPs KG
(4-planar)x12(2-bent)x24___(D3h)	[Cr2]12[(C6H3)(OCH2CH3)(CO2)2]24	0	6243.91668	Not in OntoMOPs KG
(4-planar)x12(2-bent)x24___(D3h)	[Cr2]12[(C6H3)(OC2H4)2(OH)(CO2)2]24	0	7685.157528	Not in OntoMOPs KG
(4-planar)x12(2-bent)x24___(D3h)	[Cr2]12[(C6H3)(OC2H4)3(OH)(CO2)2]24	0	8742.410592	Not in OntoMOPs KG
(4-planar)x12(2-bent)x24___(D3h)	[Cr2]12[(C6H3)O(CH2)11CH3(CO2)2]24	0	9610.24308	Not in OntoMOPs KG
(4-planar)x12(2-bent)x24___(D3h)	[Cr2]12[(C6H3)O(CH2)4CH3(CO2)2]24	0	7253.8146	Not in OntoMOPs KG
(4-planar)x12(2-bent)x24___(D3h)	[Cr2]12[(C6H3)(OC2H4OH)(CO2)2]24	0	6627.904464	Not in OntoMOPs KG
(4-planar)x12(2-bent)x24___(D3h)	[Cr2]12[(C6H3)NHCOC(CH3)3(CO2)2]24	0	7565.78748	Not in OntoMOPs KG
(4-planar)x12(2-bent)x24___(D3h)	[Cr2]12[(C6H3)CH2CS2(C6H5)(CO2)2]24	0	9176.965536	Not in OntoMOPs KG
(4-planar)x12(2-bent)x24___(D3h)	[Cr2]12[C6H3CH2CS3C4H9(CO2)2]24	0	9466.763616	Not in OntoMOPs KG
(4-planar)x12(2-bent)x24___(D3h)	[Cr2]12[C6H3C2Si(C3H7)3(CO2)2]24	0	9515.298816	Not in OntoMOPs KG
(4-planar)x12(2-bent)x24___(D3h)	[Cr2]12[(C6H3)(CH2)(C4N2H2O2CH3)(CO2)2]24	0	8501.629104	Not in OntoMOPs KG
(4-planar)x12(2-bent)x24___(D3h)	[Cr2]12[(C6H3)((C2H4O2)(C9H5O2))(CO2)2]24	0	10086.94291	Not in OntoMOPs KG
(4-planar)x12(2-bent)x24___(D3h)	[Cr2]12[(C6H3)(N2(C6H4CH3))(CO2)2]24	0	8021.910336	Not in OntoMOPs KG
(4-planar)x12(2-bent)x24___(D3h)	[Cr2]12[((C6H3)C(CH3)3)(CO2)2]24	0	6533.194176	Not in OntoMOPs KG
(4-planar)x12(2-bent)x24___(D3h)	[Cr2]12[(C6H3SO3)(CO2)2]24	0	7083.999048	Not in OntoMOPs KG
(4-planar)x12(2-bent)x24___(D3h)	[Cr2]12[(C6H3)O(CH2)13CH3(CO2)2]24	0	10283.50836	Not in OntoMOPs KG
(4-planar)x12(2-bent)x24___(D3h)	[Cr2]12[C6H3CO2C2H4Si(CH3)3(CO2)2]24	0	8648.487024	Not in OntoMOPs KG
(4-planar)x12(2-bent)x24___(D3h)	[Cr2]12[(C6H3)(CO2)3]24	-24	6218.708064	Not in OntoMOPs KG
(4-planar)x12(2-bent)x24___(D3h)	[Cr2]12[C6H3CO2CH3(CO2)2]24	0	6579.528624	Not in OntoMOPs KG
(4-planar)x12(2-bent)x24___(D3h)	[Cr2]12[C6H3OCOCHCH2(CO2)2]24	0	6867.785424	Not in OntoMOPs KG
(4-planar)x12(2-bent)x24___(D3h)	[Cr2]12[C6H3NHCO2C(CH3)3(CO2)2]24	0	7949.775264	Not in OntoMOPs KG
(4-planar)x12(2-bent)x24___(D3h)	[Cr2]12[(C6H2O)(CH2CHCH2)(CO2)2]24	0	6532.17348	Not in OntoMOPs KG
(4-planar)x12(2-bent)x24___(D3h)	[Cr2]12[(C6H3)O(CH2)3CH3(CO2)2]24	0	6917.18196	Not in OntoMOPs KG
(4-planar)x12(2-bent)x24___(D3h)	[Cr2]12[(C6H3)C6H2(OC16H33)3(CO2)2]24	0	24274.91033	Not in OntoMOPs KG
(4-planar)x12(2-bent)x24___(D3h)	[Cr2]12[C6H3N2C6H3(CH3)2(CO2)2]24	0	8358.542976	Not in OntoMOPs KG
(4-planar)x12(2-bent)x24___(D3h)	[Cr2]12[(C6H3)(C2H4O2)(CONHC6H5)(CO2)2]24	0	9486.790248	Not in OntoMOPs KG
(4-planar)x12(2-bent)x24___(D3h)	[Cr2]12[(C6H3)(OCH3)(CO2)2]24	0	5907.28404	Not in OntoMOPs KG
(4-planar)x12(2-bent)x24___(D3h)	[CuPd]12[(C6H4)(CO2)2]24	0	5978.349216	Not in OntoMOPs KG
(4-planar)x12(2-bent)x24___(D3h)	[CuPd]12[(C6H3OH)(CO2)2]24	0	6362.337	Not in OntoMOPs KG
(4-planar)x12(2-bent)x24___(D3h)	[CuPd]12[(C6H3(NO2))(CO2)2]24	0	7058.297664	Not in OntoMOPs KG
(4-planar)x12(2-bent)x24___(D3h)	[CuPd]12[(C6H3(CH3))(CO2)2]24	0	6314.981856	Not in OntoMOPs KG
(4-planar)x12(2-bent)x24___(D3h)	[CuPd]12[(C6H3Br)(CO2)2]24	0	7871.857296	Not in OntoMOPs KG
(4-planar)x12(2-bent)x24___(D3h)	[CuPd]12[(C6H3)((C3H6O2)(C9H5O2))(CO2)2]24	0	11215.26115	Not in OntoMOPs KG
(4-planar)x12(2-bent)x24___(D3h)	[CuPd]12[(C6H3)((C6H12O2)(C9H5O2))(CO2)2]24	0	12225.15907	Not in OntoMOPs KG
(4-planar)x12(2-bent)x24___(D3h)	[CuPd]12[(C6H3)(OC2H4)3(OCH3)(CO2)2]24	0	9870.728832	Not in OntoMOPs KG
(4-planar)x12(2-bent)x24___(D3h)	[CuPd]12[(C6H3)(OCH2C2H)(CO2)2]24	0	7275.48324	Not in OntoMOPs KG
(4-planar)x12(2-bent)x24___(D3h)	[CuPd]12[(C6H3NH2)(CO2)2]24	0	6338.697936	Not in OntoMOPs KG
(4-planar)x12(2-bent)x24___(D3h)	[CuPd]12[(C6H3)(OCH2CH2CH3)(CO2)2]24	0	7372.23492	Not in OntoMOPs KG
(4-planar)x12(2-bent)x24___(D3h)	[CuPd]12[(C10H6)(CO2)2]24	0	7179.752256	Not in OntoMOPs KG
(4-planar)x12(2-bent)x24___(D3h)	[CuPd]12[(C6H3)2(CO2)2]24	-24	7010.393664	Not in OntoMOPs KG
(4-planar)x12(2-bent)x24___(D3h)	[CuPd]12[(C6H3)(OCH2CH3)(CO2)2]24	0	7035.60228	Not in OntoMOPs KG
(4-planar)x12(2-bent)x24___(D3h)	[CuPd]12[(C6H3)(OC2H4)2(OH)(CO2)2]24	0	8476.843128	Not in OntoMOPs KG
(4-planar)x12(2-bent)x24___(D3h)	[CuPd]12[(C6H3)(OC2H4)3(OH)(CO2)2]24	0	9534.096192	Not in OntoMOPs KG
(4-planar)x12(2-bent)x24___(D3h)	[CuPd]12[(C6H3)O(CH2)11CH3(CO2)2]24	0	10401.92868	Not in OntoMOPs KG
(4-planar)x12(2-bent)x24___(D3h)	[CuPd]12[(C6H3)O(CH2)4CH3(CO2)2]24	0	8045.5002	Not in OntoMOPs KG
(4-planar)x12(2-bent)x24___(D3h)	[CuPd]12[(C6H3)(OC2H4OH)(CO2)2]24	0	7419.590064	Not in OntoMOPs KG
(4-planar)x12(2-bent)x24___(D3h)	[CuPd]12[(C6H3)NHCOC(CH3)3(CO2)2]24	0	8357.47308	Not in OntoMOPs KG
(4-planar)x12(2-bent)x24___(D3h)	[CuPd]12[(C6H3)CH2CS2(C6H5)(CO2)2]24	0	9968.651136	Not in OntoMOPs KG

(4-planar)x12(2-bent)x24___(D3h)	[CuPd]12[C6H3CH2CS3C4H9(CO2)2]24	0	10258.44922	Not in OntoMOPs KG
(4-planar)x12(2-bent)x24___(D3h)	[CuPd]12[C6H3C2Si(C3H7)3(CO2)2]24	0	10306.98442	Not in OntoMOPs KG
(4-planar)x12(2-bent)x24___(D3h)	[CuPd]12[(C6H3)(CH2)(C4N2H2O2CH3)(CO2)2]24	0	9293.314704	Not in OntoMOPs KG
(4-planar)x12(2-bent)x24___(D3h)	[CuPd]12[(C6H3)((C2H4O2)(C9H5O2))(CO2)2]24	0	10878.62851	Not in OntoMOPs KG
(4-planar)x12(2-bent)x24___(D3h)	[CuPd]12[(C6H3)(N2(C6H4CH3))(CO2)2]24	0	8813.595936	Not in OntoMOPs KG
(4-planar)x12(2-bent)x24___(D3h)	[CuPd]12[((C6H3)C(CH3)3)(CO2)2]24	0	7324.879776	Not in OntoMOPs KG
(4-planar)x12(2-bent)x24___(D3h)	[CuPd]12[(C6H3SO3)(CO2)2]24	0	7875.684648	Not in OntoMOPs KG
(4-planar)x12(2-bent)x24___(D3h)	[CuPd]12[(C6H3)O(CH2)13CH3(CO2)2]24	0	11075.19396	Not in OntoMOPs KG
(4-planar)x12(2-bent)x24___(D3h)	[CuPd]12[C6H3CO2C2H4Si(CH3)3(CO2)2]24	0	9440.172624	Not in OntoMOPs KG
(4-planar)x12(2-bent)x24___(D3h)	[CuPd]12[(C6H3)(CO2)3]24	-24	7010.393664	Not in OntoMOPs KG
(4-planar)x12(2-bent)x24___(D3h)	[CuPd]12[C6H3CO2CH3(CO2)2]24	0	7371.214224	Not in OntoMOPs KG
(4-planar)x12(2-bent)x24___(D3h)	[CuPd]12[C6H3OCOCHCH2(CO2)2]24	0	7659.471024	Not in OntoMOPs KG
(4-planar)x12(2-bent)x24___(D3h)	[CuPd]12[C6H3NHCO2C(CH3)3(CO2)2]24	0	8741.460864	Not in OntoMOPs KG
(4-planar)x12(2-bent)x24___(D3h)	[CuPd]12[(C6H2O)(CH2CHCH2)(CO2)2]24	0	7323.85908	Not in OntoMOPs KG
(4-planar)x12(2-bent)x24___(D3h)	[CuPd]12[(C6H3)O(CH2)3CH3(CO2)2]24	0	7708.86756	Not in OntoMOPs KG
(4-planar)x12(2-bent)x24___(D3h)	[CuPd]12[(C6H3)C6H2(OC16H33)3(CO2)2]24	0	25066.59593	Not in OntoMOPs KG
(4-planar)x12(2-bent)x24___(D3h)	[CuPd]12[C6H3N2C6H3(CH3)2(CO2)2]24	0	9150.228576	Not in OntoMOPs KG
(4-planar)x12(2-bent)x24___(D3h)	[CuPd]12[(C6H3)(C2H4O2)(CONHC6H5)(CO2)2]24	0	10278.47585	Not in OntoMOPs KG
(4-planar)x12(2-bent)x24___(D3h)	[CuPd]12[(C6H3)(OCH3)(CO2)2]24	0	6698.96964	Not in OntoMOPs KG
(4-planar)x12(2-bent)x24___(D3h)	[CuNi]12[(C6H4)(CO2)2]24	0	5405.630016	Not in OntoMOPs KG
(4-planar)x12(2-bent)x24___(D3h)	[CuNi]12[(C6H3OH)(CO2)2]24	0	5789.6178	Not in OntoMOPs KG
(4-planar)x12(2-bent)x24___(D3h)	[CuNi]12[(C6H3(NO2))(CO2)2]24	0	6485.578464	Not in OntoMOPs KG
(4-planar)x12(2-bent)x24___(D3h)	[CuNi]12[(C6H3(CH3))(CO2)2]24	0	5742.262656	Not in OntoMOPs KG
(4-planar)x12(2-bent)x24___(D3h)	[CuNi]12[(C6H3Br)(CO2)2]24	0	7299.138096	Not in OntoMOPs KG
(4-planar)x12(2-bent)x24___(D3h)	[CuNi]12[(C6H3)((C3H6O2)(C9H5O2))(CO2)2]24	0	10642.54195	Not in OntoMOPs KG
(4-planar)x12(2-bent)x24___(D3h)	[CuNi]12[(C6H3)((C6H12O2)(C9H5O2))(CO2)2]24	0	11652.43987	Not in OntoMOPs KG
(4-planar)x12(2-bent)x24___(D3h)	[CuNi]12[(C6H3)(OC2H4)3(OCH3)(CO2)2]24	0	9298.009632	Not in OntoMOPs KG
(4-planar)x12(2-bent)x24___(D3h)	[CuNi]12[(C6H3)(OCH2C2H)(CO2)2]24	0	6702.76404	Not in OntoMOPs KG
(4-planar)x12(2-bent)x24___(D3h)	[CuNi]12[(C6H3NH2)(CO2)2]24	0	5765.978736	Not in OntoMOPs KG
(4-planar)x12(2-bent)x24___(D3h)	[CuNi]12[(C6H3)(OCH2CH2CH3)(CO2)2]24	0	6799.51572	Not in OntoMOPs KG
(4-planar)x12(2-bent)x24___(D3h)	[CuNi]12[(C10H6)(CO2)2]24	0	6607.033056	Not in OntoMOPs KG
(4-planar)x12(2-bent)x24___(D3h)	[CuNi]12[(C6H3)2(CO2)2]24	-24	6437.674464	Not in OntoMOPs KG
(4-planar)x12(2-bent)x24___(D3h)	[CuNi]12[(C6H3)(OCH2CH3)(CO2)2]24	0	6462.88308	Not in OntoMOPs KG
(4-planar)x12(2-bent)x24___(D3h)	[CuNi]12[(C6H3)(OC2H4)2(OH)(CO2)2]24	0	7904.123928	Not in OntoMOPs KG
(4-planar)x12(2-bent)x24___(D3h)	[CuNi]12[(C6H3)(OC2H4)3(OH)(CO2)2]24	0	8961.376992	Not in OntoMOPs KG
(4-planar)x12(2-bent)x24___(D3h)	[CuNi]12[(C6H3)O(CH2)11CH3(CO2)2]24	0	9829.20948	Not in OntoMOPs KG
(4-planar)x12(2-bent)x24___(D3h)	[CuNi]12[(C6H3)O(CH2)4CH3(CO2)2]24	0	7472.781	Not in OntoMOPs KG
(4-planar)x12(2-bent)x24___(D3h)	[CuNi]12[(C6H3)(OC2H4OH)(CO2)2]24	0	6846.870864	Not in OntoMOPs KG
(4-planar)x12(2-bent)x24___(D3h)	[CuNi]12[(C6H3)NHCOC(CH3)3(CO2)2]24	0	7784.75388	Not in OntoMOPs KG
(4-planar)x12(2-bent)x24___(D3h)	[CuNi]12[(C6H3)CH2CS2(C6H5)(CO2)2]24	0	9395.931936	Not in OntoMOPs KG
(4-planar)x12(2-bent)x24___(D3h)	[CuNi]12[C6H3CH2CS3C4H9(CO2)2]24	0	9685.730016	Not in OntoMOPs KG
(4-planar)x12(2-bent)x24___(D3h)	[CuNi]12[C6H3C2Si(C3H7)3(CO2)2]24	0	9734.265216	Not in OntoMOPs KG
(4-planar)x12(2-bent)x24___(D3h)	[CuNi]12[(C6H3)(CH2)(C4N2H2O2CH3)(CO2)2]24	0	8720.595504	Not in OntoMOPs KG
(4-planar)x12(2-bent)x24___(D3h)	[CuNi]12[(C6H3)((C2H4O2)(C9H5O2))(CO2)2]24	0	10305.90931	Not in OntoMOPs KG
(4-planar)x12(2-bent)x24___(D3h)	[CuNi]12[(C6H3)(N2(C6H4CH3))(CO2)2]24	0	8240.876736	Not in OntoMOPs KG
(4-planar)x12(2-bent)x24___(D3h)	[CuNi]12[((C6H3)C(CH3)3)(CO2)2]24	0	6752.160576	Not in OntoMOPs KG
(4-planar)x12(2-bent)x24___(D3h)	[CuNi]12[(C6H3SO3)(CO2)2]24	0	7302.965448	Not in OntoMOPs KG
(4-planar)x12(2-bent)x24___(D3h)	[CuNi]12[(C6H3)O(CH2)13CH3(CO2)2]24	0	10502.47476	Not in OntoMOPs KG
(4-planar)x12(2-bent)x24___(D3h)	[CuNi]12[C6H3CO2C2H4Si(CH3)3(CO2)2]24	0	8867.453424	Not in OntoMOPs KG
(4-planar)x12(2-bent)x24___(D3h)	[CuNi]12[(C6H3)(CO2)3]24	-24	6437.674464	Not in OntoMOPs KG

(4-planar)x12(2-bent)x24___(D3h)	[CuNi]12[C6H3CO2CH3(CO2)2]24	0	6798.495024	Not in OntoMOPs KG
(4-planar)x12(2-bent)x24___(D3h)	[CuNi]12[C6H3OCOCHCH2(CO2)2]24	0	7086.751824	Not in OntoMOPs KG
(4-planar)x12(2-bent)x24___(D3h)	[CuNi]12[C6H3NHCO2C(CH3)3(CO2)2]24	0	8168.741664	Not in OntoMOPs KG
(4-planar)x12(2-bent)x24___(D3h)	[CuNi]12[(C6H2O)(CH2CHCH2)(CO2)2]24	0	6751.13988	Not in OntoMOPs KG
(4-planar)x12(2-bent)x24___(D3h)	[CuNi]12[(C6H3)O(CH2)3CH3(CO2)2]24	0	7136.14836	Not in OntoMOPs KG
(4-planar)x12(2-bent)x24___(D3h)	[CuNi]12[(C6H3)C6H2(OC16H33)3(CO2)2]24	0	24493.87673	Not in OntoMOPs KG
(4-planar)x12(2-bent)x24___(D3h)	[CuNi]12[C6H3N2C6H3(CH3)2(CO2)2]24	0	8577.509376	Not in OntoMOPs KG
(4-planar)x12(2-bent)x24___(D3h)	[CuNi]12[(C6H3)(C2H4O2)(CONHC6H5)(CO2)2]24	0	9705.756648	Not in OntoMOPs KG
(4-planar)x12(2-bent)x24___(D3h)	[CuNi]12[(C6H3)(OCH3)(CO2)2]24	0	6126.25044	Not in OntoMOPs KG
(4-planar)x12(2-bent)x24___(D3h)	[Mo2]12[(C6H4)(CO2)2]24	0	6241.317216	10.1021/ic050460z
(4-planar)x12(2-bent)x24___(D3h)	[Mo2]12[(C6H3OH)(CO2)2]24	0	6625.305	Not in OntoMOPs KG
(4-planar)x12(2-bent)x24___(D3h)	[Mo2]12[(C6H3(NO2))(CO2)2]24	0	7321.265664	Not in OntoMOPs KG
(4-planar)x12(2-bent)x24___(D3h)	[Mo2]12[(C6H3(CH3))(CO2)2]24	0	6577.949856	Not in OntoMOPs KG
(4-planar)x12(2-bent)x24___(D3h)	[Mo2]12[(C6H3Br)(CO2)2]24	0	8134.825296	Not in OntoMOPs KG
(4-planar)x12(2-bent)x24___(D3h)	[Mo2]12[(C6H3)((C3H6O2)(C9H5O2))(CO2)2]24	0	11478.22915	Not in OntoMOPs KG
(4-planar)x12(2-bent)x24___(D3h)	[Mo2]12[(C6H3)((C6H12O2)(C9H5O2))(CO2)2]24	0	12488.12707	Not in OntoMOPs KG
(4-planar)x12(2-bent)x24___(D3h)	[Mo2]12[(C6H3)(OC2H4)3(OCH3)(CO2)2]24	0	10133.69683	Not in OntoMOPs KG
(4-planar)x12(2-bent)x24___(D3h)	[Mo2]12[(C6H3)(OCH2C2H)(CO2)2]24	0	7538.45124	Not in OntoMOPs KG
(4-planar)x12(2-bent)x24___(D3h)	[Mo2]12[(C6H3NH2)(CO2)2]24	0	6601.665936	Not in OntoMOPs KG
(4-planar)x12(2-bent)x24___(D3h)	[Mo2]12[(C6H3)(OCH2CH2CH3)(CO2)2]24	0	7635.20292	Not in OntoMOPs KG
(4-planar)x12(2-bent)x24___(D3h)	[Mo2]12[(C10H6)(CO2)2]24	0	7442.720256	10.1021/ja1080794
(4-planar)x12(2-bent)x24___(D3h)	[Mo2]12[(C6H3)2(CO2)2]24	-24	7273.361664	Not in OntoMOPs KG
(4-planar)x12(2-bent)x24___(D3h)	[Mo2]12[(C6H3)(OCH2CH3)(CO2)2]24	0	7298.57028	Not in OntoMOPs KG
(4-planar)x12(2-bent)x24___(D3h)	[Mo2]12[(C6H3)(OC2H4)2(OH)(CO2)2]24	0	8739.811128	Not in OntoMOPs KG
(4-planar)x12(2-bent)x24___(D3h)	[Mo2]12[(C6H3)(OC2H4)3(OH)(CO2)2]24	0	9797.064192	Not in OntoMOPs KG
(4-planar)x12(2-bent)x24___(D3h)	[Mo2]12[(C6H3)O(CH2)11CH3(CO2)2]24	0	10664.89668	Not in OntoMOPs KG
(4-planar)x12(2-bent)x24___(D3h)	[Mo2]12[(C6H3)O(CH2)4CH3(CO2)2]24	0	8308.4682	Not in OntoMOPs KG
(4-planar)x12(2-bent)x24___(D3h)	[Mo2]12[(C6H3)(OC2H4OH)(CO2)2]24	0	7682.558064	Not in OntoMOPs KG
(4-planar)x12(2-bent)x24___(D3h)	[Mo2]12[(C6H3)NHCOC(CH3)3(CO2)2]24	0	8620.44108	Not in OntoMOPs KG
(4-planar)x12(2-bent)x24___(D3h)	[Mo2]12[(C6H3)CH2CS2(C6H5)(CO2)2]24	0	10231.61914	Not in OntoMOPs KG
(4-planar)x12(2-bent)x24___(D3h)	[Mo2]12[C6H3CH2CS3C4H9(CO2)2]24	0	10521.41722	Not in OntoMOPs KG
(4-planar)x12(2-bent)x24___(D3h)	[Mo2]12[C6H3C2Si(C3H7)3(CO2)2]24	0	10569.95242	Not in OntoMOPs KG
(4-planar)x12(2-bent)x24___(D3h)	[Mo2]12[(C6H3)(CH2)(C4N2H2O2CH3)(CO2)2]24	0	9556.282704	Not in OntoMOPs KG
(4-planar)x12(2-bent)x24___(D3h)	[Mo2]12[(C6H3)((C2H4O2)(C9H5O2))(CO2)2]24	0	11141.59651	Not in OntoMOPs KG
(4-planar)x12(2-bent)x24___(D3h)	[Mo2]12[(C6H3)(N2(C6H4CH3))(CO2)2]24	0	9076.563936	Not in OntoMOPs KG
(4-planar)x12(2-bent)x24___(D3h)	[Mo2]12[((C6H3)C(CH3)3)(CO2)2]24	0	7587.847776	Not in OntoMOPs KG
(4-planar)x12(2-bent)x24___(D3h)	[Mo2]12[(C6H3SO3)(CO2)2]24	0	8138.652648	Not in OntoMOPs KG
(4-planar)x12(2-bent)x24___(D3h)	[Mo2]12[(C6H3)O(CH2)13CH3(CO2)2]24	0	11338.16196	Not in OntoMOPs KG
(4-planar)x12(2-bent)x24___(D3h)	[Mo2]12[C6H3CO2C2H4Si(CH3)3(CO2)2]24	0	9703.140624	Not in OntoMOPs KG
(4-planar)x12(2-bent)x24___(D3h)	[Mo2]12[(C6H3)(CO2)3]24	-24	7273.361664	Not in OntoMOPs KG
(4-planar)x12(2-bent)x24___(D3h)	[Mo2]12[C6H3CO2CH3(CO2)2]24	0	7634.182224	Not in OntoMOPs KG
(4-planar)x12(2-bent)x24___(D3h)	[Mo2]12[C6H3OCOCHCH2(CO2)2]24	0	7922.439024	Not in OntoMOPs KG
(4-planar)x12(2-bent)x24___(D3h)	[Mo2]12[C6H3NHCO2C(CH3)3(CO2)2]24	0	9004.428864	Not in OntoMOPs KG
(4-planar)x12(2-bent)x24___(D3h)	[Mo2]12[(C6H2O)(CH2CHCH2)(CO2)2]24	0	7586.82708	Not in OntoMOPs KG
(4-planar)x12(2-bent)x24___(D3h)	[Mo2]12[(C6H3)O(CH2)3CH3(CO2)2]24	0	7971.83556	Not in OntoMOPs KG
(4-planar)x12(2-bent)x24___(D3h)	[Mo2]12[(C6H3)C6H2(OC16H33)3(CO2)2]24	0	25329.56393	Not in OntoMOPs KG
(4-planar)x12(2-bent)x24___(D3h)	[Mo2]12[C6H3N2C6H3(CH3)2(CO2)2]24	0	9413.196576	Not in OntoMOPs KG
(4-planar)x12(2-bent)x24___(D3h)	[Mo2]12[(C6H3)(C2H4O2)(CONHC6H5)(CO2)2]24	0	10541.44385	Not in OntoMOPs KG
(4-planar)x12(2-bent)x24___(D3h)	[Mo2]12[(C6H3)(OCH3)(CO2)2]24	0	6961.93764	Not in OntoMOPs KG
(4-pyramidal)x6(3-pyramidal)x8___(Oh)	[Ni4C24H12O12S4]6[(C6H9)(CO2)3]8	0	6837.57924	Not in OntoMOPs KG

(4-pyramidal)x6(3-pyramidal)x8___(Oh)	[Ni4C24H12O12S4]6[(C10H6)4(C6HC2H4)2(CPO4)(CO2)4]8	0	13051.36241	Not in OntoMOPs KG
(4-pyramidal)x6(3-pyramidal)x8___(Oh)	[Ni4C24H12O12S4]6[(C6H4)4(C6HC2H4)2(CPO4)(CO2)4]8	0	13051.36241	Not in OntoMOPs KG
(4-pyramidal)x6(3-pyramidal)x8___(Oh)	[V4O8]6[(C6H9)(CO2)3]8	0	3695.881296	Not in OntoMOPs KG
(4-pyramidal)x6(3-pyramidal)x8___(Oh)	[V4O8]6[(C10H6)4(C6HC2H4)2(CPO4)(CO2)4]8	0	9909.664464	Not in OntoMOPs KG
(4-pyramidal)x6(3-pyramidal)x8___(Oh)	[V4O8]6[(C6H4)4(C6HC2H4)2(CPO4)(CO2)4]8	0	9909.664464	Not in OntoMOPs KG
(4-pyramidal)x6(3-pyramidal)x8___(Oh)	[V5O9]6[(C6H9)(CO2)3]8	-6	4097.527242	Not in OntoMOPs KG
(4-pyramidal)x6(3-pyramidal)x8___(Oh)	[V5O9]6[(C10H6)4(C6HC2H4)2(CPO4)(CO2)4]8	-6	10311.31041	Not in OntoMOPs KG
(4-pyramidal)x6(3-pyramidal)x8___(Oh)	[V5O9]6[(C6H4)4(C6HC2H4)2(CPO4)(CO2)4]8	-6	10311.31041	Not in OntoMOPs KG
(4-pyramidal)x6(3-pyramidal)x8___(Oh)	[Co4C48H28O4S4]6[(C6H9)(CO2)3]8	0	7421.889432	Not in OntoMOPs KG
(4-pyramidal)x6(3-pyramidal)x8___(Oh)	[Co4C48H28O4S4]6[(C10H6)4(C6HC2H4)2(CPO4)(CO2)4]8	0	13635.6726	Not in OntoMOPs KG
(4-pyramidal)x6(3-pyramidal)x8___(Oh)	[Co4C48H28O4S4]6[(C6H4)4(C6HC2H4)2(CPO4)(CO2)4]8	0	13635.6726	Not in OntoMOPs KG
(4-pyramidal)x6(3-pyramidal)x8___(Oh)	[Co4C40H44O4S4]6[(C6H9)(CO2)3]8	0	8189.865	Not in OntoMOPs KG
(4-pyramidal)x6(3-pyramidal)x8___(Oh)	[Co4C40H44O4S4]6[(C10H6)4(C6HC2H4)2(CPO4)(CO2)4]8	0	14403.64817	Not in OntoMOPs KG
(4-pyramidal)x6(3-pyramidal)x8___(Oh)	[Co4C40H44O4S4]6[(C6H4)4(C6HC2H4)2(CPO4)(CO2)4]8	0	14403.64817	Not in OntoMOPs KG
(4-pyramidal)x6(3-pyramidal)x8___(Oh)	[Co4C40H44O12S4]6[(C6H9)(CO2)3]8	0	9536.39556	Not in OntoMOPs KG
(4-pyramidal)x6(3-pyramidal)x8___(Oh)	[Co4C40H44O12S4]6[(C10H6)4(C6HC2H4)2(CPO4)(CO2)4]8	0	15750.17873	10.1038/s41467-019-08416-6
(4-pyramidal)x6(3-pyramidal)x8___(Oh)	[Co4C40H44O12S4]6[(C6H4)4(C6HC2H4)2(CPO4)(CO2)4]8	0	15750.17873	10.1038/s41467-019-08416-6
(4-pyramidal)x6(3-pyramidal)x8___(Oh)	[Ni4C40H44S4O12]6[(C6H9)(CO2)3]8	0	8184.1098	Not in OntoMOPs KG
(4-pyramidal)x6(3-pyramidal)x8___(Oh)	[Ni4C40H44S4O12]6[(C10H6)4(C6HC2H4)2(CPO4)(CO2)4]8	0	14397.89297	Not in OntoMOPs KG
(4-pyramidal)x6(3-pyramidal)x8___(Oh)	[Ni4C40H44S4O12]6[(C6H4)4(C6HC2H4)2(CPO4)(CO2)4]8	0	14397.89297	10.1038/s41467-019-08416-6
(4-pyramidal)x6(3-pyramidal)x8___(Oh)	[Mg4C40H44O12S4]6[(C6H9)(CO2)3]8	0	7358.7882	Not in OntoMOPs KG
(4-pyramidal)x6(3-pyramidal)x8___(Oh)	[Mg4C40H44O12S4]6[(C10H6)4(C6HC2H4)2(CPO4)(CO2)4]8	0	13572.57137	Not in OntoMOPs KG
(4-pyramidal)x6(3-pyramidal)x8___(Oh)	[Mg4C40H44O12S4]6[(C6H4)4(C6HC2H4)2(CPO4)(CO2)4]8	0	13572.57137	Not in OntoMOPs KG
(4-pyramidal)x6(3-pyramidal)x8___(Oh)	[Fe4C40H44S4O4]6[(C6H9)(CO2)3]8	0	7347.772632	Not in OntoMOPs KG
(4-pyramidal)x6(3-pyramidal)x8___(Oh)	[Fe4C40H44S4O4]6[(C10H6)4(C6HC2H4)2(CPO4)(CO2)4]8	0	13561.5558	Not in OntoMOPs KG
(4-pyramidal)x6(3-pyramidal)x8___(Oh)	[Fe4C40H44S4O4]6[(C6H4)4(C6HC2H4)2(CPO4)(CO2)4]8	0	13561.5558	Not in OntoMOPs KG
(4-pyramidal)x6(3-pyramidal)x8___(Oh)	[(CHC6HO3)4(C3H6OH)4]6[Ni3]8	-24	6044.852016	10.1021/jacs.7b00037
(4-pyramidal)x6(3-pyramidal)x8___(Oh)	[(CHC6HO3)4(C5H11)4]6[Ni3]8	-24	6334.129512	Not in OntoMOPs KG
(4-pyramidal)x6(3-pyramidal)x8___(Oh)	[(C6HO3)4(C4H8)4]6[Ni3]8	-24	5660.864232	10.1002/anie.201107182
(4-pyramidal)x6(3-pyramidal)x8___(Oh)	[Co4C24H8O24S8]6[(C6H9)(CO2)3]8	0	6843.33444	Not in OntoMOPs KG
(4-pyramidal)x6(3-pyramidal)x8___(Oh)	[Co4C24H8O24S8]6[(C10H6)4(C6HC2H4)2(CPO4)(CO2)4]8	0	13057.11761	Not in OntoMOPs KG
(4-pyramidal)x6(3-pyramidal)x8___(Oh)	[Co4C24H8O24S8]6[(C6H4)4(C6HC2H4)2(CPO4)(CO2)4]8	0	13057.11761	Not in OntoMOPs KG
(4-pyramidal)x6(3-pyramidal)x8___(Oh)	[Co4C24H12O12S4]6[(C6H9)(CO2)3]8	0	7901.651352	10.1021/ja300095j
(4-pyramidal)x6(3-pyramidal)x8___(Oh)	[Co4C24H12O12S4]6[(C10H6)4(C6HC2H4)2(CPO4)(CO2)4]8	0	14115.43452	Not in OntoMOPs KG
(4-pyramidal)x6(3-pyramidal)x8___(Oh)	[Co4C24H12O12S4]6[(C6H4)4(C6HC2H4)2(CPO4)(CO2)4]8	0	14115.43452	Not in OntoMOPs KG
(4-pyramidal)x6(3-pyramidal)x8___(Oh)	[Zn4C40H44O12S4]6[(C6H9)(CO2)3]8	0	8344.8282	Not in OntoMOPs KG
(4-pyramidal)x6(3-pyramidal)x8___(Oh)	[Zn4C40H44O12S4]6[(C10H6)4(C6HC2H4)2(CPO4)(CO2)4]8	0	14558.61137	Not in OntoMOPs KG
(4-pyramidal)x6(3-pyramidal)x8___(Oh)	[Zn4C40H44O12S4]6[(C6H4)4(C6HC2H4)2(CPO4)(CO2)4]8	0	14558.61137	Not in OntoMOPs KG
(4-pyramidal)x6(3-pyramidal)x8___(Oh)	[Co4C56H76O12S4]6[(C6H9)(CO2)3]8	-24	8740.669872	Not in OntoMOPs KG
(4-pyramidal)x6(3-pyramidal)x8___(Oh)	[Co4C56H76O12S4]6[(C10H6)4(C6HC2H4)2(CPO4)(CO2)4]8	-24	14954.45304	Not in OntoMOPs KG
(4-pyramidal)x6(3-pyramidal)x8___(Oh)	[Co4C56H76O12S4]6[(C6H4)4(C6HC2H4)2(CPO4)(CO2)4]8	-24	14954.45304	Not in OntoMOPs KG
(4-pyramidal)x6(3-pyramidal)x8___(Oh)	[Mg4C56H76O12S4]6[(C6H9)(CO2)3]8	0	8705.31876	Not in OntoMOPs KG
(4-pyramidal)x6(3-pyramidal)x8___(Oh)	[Mg4C56H76O12S4]6[(C10H6)4(C6HC2H4)2(CPO4)(CO2)4]8	0	14919.10193	Not in OntoMOPs KG
(4-pyramidal)x6(3-pyramidal)x8___(Oh)	[Mg4C56H76O12S4]6[(C6H4)4(C6HC2H4)2(CPO4)(CO2)4]8	0	14919.10193	Not in OntoMOPs KG
(4-pyramidal)x6(3-pyramidal)x8___(Oh)	[Ni4C56H76O12S4]6[(C6H9)(CO2)3]8	0	9530.64036	Not in OntoMOPs KG
(4-pyramidal)x6(3-pyramidal)x8___(Oh)	[Ni4C56H76O12S4]6[(C10H6)4(C6HC2H4)2(CPO4)(CO2)4]8	0	15744.42353	Not in OntoMOPs KG
(4-pyramidal)x6(3-pyramidal)x8___(Oh)	[Ni4C56H76O12S4]6[(C6H4)4(C6HC2H4)2(CPO4)(CO2)4]8	0	15744.42353	Not in OntoMOPs KG
(4-planar)x6(3-pyramidal)x8___(Oh)	[Rh2]6[(C6H3)(CONHC6H4)3(CO2)3]8	0	5750.685672	Not in OntoMOPs KG
(4-planar)x6(3-pyramidal)x8___(Oh)	[Rh2]6[C6(CH3)3(CH2)3(CO2)3]8	0	3565.065168	Not in OntoMOPs KG
(4-planar)x6(3-pyramidal)x8___(Oh)	[Rh2]6[C6H3(CONHCHCH3)3(CO2)3]8	0	4597.658472	Not in OntoMOPs KG

(4-planar)x6(3-pyramidal)x8___(Oh)	[Rh2]6[(C6H3)(OC6H4)3(CO2)3]8	0	5102.080152	Not in OntoMOPs KG
(4-planar)x6(3-pyramidal)x8___(Oh)	[Ru2]6[(C6H3)(CONHC6H4)3(CO2)3]8	0	5728.659672	Not in OntoMOPs KG
(4-planar)x6(3-pyramidal)x8___(Oh)	[Ru2]6[C6(CH3)3(CH2)3(CO2)3]8	0	3543.039168	Not in OntoMOPs KG
(4-planar)x6(3-pyramidal)x8___(Oh)	[Ru2]6[C6H3(CONHCHCH3)3(CO2)3]8	0	4575.632472	Not in OntoMOPs KG
(4-planar)x6(3-pyramidal)x8___(Oh)	[Ru2]6[(C6H3)(OC6H4)3(CO2)3]8	0	5080.054152	Not in OntoMOPs KG
(4-planar)x6(3-pyramidal)x8___(Oh)	[Ni2]6[(C6H3)(CONHC6H4)3(CO2)3]8	0	5220.140472	Not in OntoMOPs KG
(4-planar)x6(3-pyramidal)x8___(Oh)	[Ni2]6[C6(CH3)3(CH2)3(CO2)3]8	0	3034.519968	Not in OntoMOPs KG
(4-planar)x6(3-pyramidal)x8___(Oh)	[Ni2]6[C6H3(CONHCHCH3)3(CO2)3]8	0	4067.113272	Not in OntoMOPs KG
(4-planar)x6(3-pyramidal)x8___(Oh)	[Ni2]6[(C6H3)(OC6H4)3(CO2)3]8	0	4571.534952	Not in OntoMOPs KG
(4-planar)x6(3-pyramidal)x8___(Oh)	[Cu2]6[(C6H3)(CONHC6H4)3(CO2)3]8	0	5278.371672	10.1021/ic802382p
(4-planar)x6(3-pyramidal)x8___(Oh)	[Cu2]6[C6(CH3)3(CH2)3(CO2)3]8	0	3092.751168	10.1021/cg4018322
(4-planar)x6(3-pyramidal)x8___(Oh)	[Cu2]6[C6H3(CONHCHCH3)3(CO2)3]8	0	4125.344472	10.1039/C5RA26357C
(4-planar)x6(3-pyramidal)x8___(Oh)	[Cu2]6[(C6H3)(OC6H4)3(CO2)3]8	0	4629.766152	10.1016/j.inoche.2019.107540
(4-planar)x6(3-pyramidal)x8___(Oh)	[Fe2]6[(C6H3)(CONHC6H4)3(CO2)3]8	0	5185.959672	Not in OntoMOPs KG
(4-planar)x6(3-pyramidal)x8___(Oh)	[Fe2]6[C6(CH3)3(CH2)3(CO2)3]8	0	3000.339168	Not in OntoMOPs KG
(4-planar)x6(3-pyramidal)x8___(Oh)	[Fe2]6[C6H3(CONHCHCH3)3(CO2)3]8	0	4032.932472	Not in OntoMOPs KG
(4-planar)x6(3-pyramidal)x8___(Oh)	[Fe2]6[(C6H3)(OC6H4)3(CO2)3]8	0	4537.354152	Not in OntoMOPs KG
(4-planar)x6(3-pyramidal)x8___(Oh)	[Co2]6[(C6H3)(CONHC6H4)3(CO2)3]8	0	5223.018072	Not in OntoMOPs KG
(4-planar)x6(3-pyramidal)x8___(Oh)	[Co2]6[C6(CH3)3(CH2)3(CO2)3]8	0	3037.397568	Not in OntoMOPs KG
(4-planar)x6(3-pyramidal)x8___(Oh)	[Co2]6[C6H3(CONHCHCH3)3(CO2)3]8	0	4069.990872	Not in OntoMOPs KG
(4-planar)x6(3-pyramidal)x8___(Oh)	[Co2]6[(C6H3)(OC6H4)3(CO2)3]8	0	4574.412552	Not in OntoMOPs KG
(4-planar)x6(3-pyramidal)x8___(Oh)	[Cr2]6[(C6H3)(CONHC6H4)3(CO2)3]8	0	5139.772872	Not in OntoMOPs KG
(4-planar)x6(3-pyramidal)x8___(Oh)	[Cr2]6[C6(CH3)3(CH2)3(CO2)3]8	0	2954.152368	Not in OntoMOPs KG
(4-planar)x6(3-pyramidal)x8___(Oh)	[Cr2]6[C6H3(CONHCHCH3)3(CO2)3]8	0	3986.745672	Not in OntoMOPs KG
(4-planar)x6(3-pyramidal)x8___(Oh)	[Cr2]6[(C6H3)(OC6H4)3(CO2)3]8	0	4491.167352	Not in OntoMOPs KG
(4-planar)x6(3-pyramidal)x8___(Oh)	[CuPd]6[(C6H3)(CONHC6H4)3(CO2)3]8	0	5535.615672	Not in OntoMOPs KG
(4-planar)x6(3-pyramidal)x8___(Oh)	[CuPd]6[C6(CH3)3(CH2)3(CO2)3]8	0	3349.995168	Not in OntoMOPs KG
(4-planar)x6(3-pyramidal)x8___(Oh)	[CuPd]6[C6H3(CONHCHCH3)3(CO2)3]8	0	4382.588472	Not in OntoMOPs KG
(4-planar)x6(3-pyramidal)x8___(Oh)	[CuPd]6[(C6H3)(OC6H4)3(CO2)3]8	0	4887.010152	Not in OntoMOPs KG
(4-planar)x6(3-pyramidal)x8___(Oh)	[CuNi]6[(C6H3)(CONHC6H4)3(CO2)3]8	0	5249.256072	Not in OntoMOPs KG
(4-planar)x6(3-pyramidal)x8___(Oh)	[CuNi]6[C6(CH3)3(CH2)3(CO2)3]8	0	3063.635568	Not in OntoMOPs KG
(4-planar)x6(3-pyramidal)x8___(Oh)	[CuNi]6[C6H3(CONHCHCH3)3(CO2)3]8	0	4096.228872	Not in OntoMOPs KG
(4-planar)x6(3-pyramidal)x8___(Oh)	[CuNi]6[(C6H3)(OC6H4)3(CO2)3]8	0	4600.650552	Not in OntoMOPs KG
(4-planar)x6(3-pyramidal)x8___(Oh)	[Mo2]6[(C6H3)(CONHC6H4)3(CO2)3]8	0	5667.099672	Not in OntoMOPs KG
(4-planar)x6(3-pyramidal)x8___(Oh)	[Mo2]6[C6(CH3)3(CH2)3(CO2)3]8	0	3481.479168	Not in OntoMOPs KG
(4-planar)x6(3-pyramidal)x8___(Oh)	[Mo2]6[C6H3(CONHCHCH3)3(CO2)3]8	0	4514.072472	Not in OntoMOPs KG
(4-planar)x6(3-pyramidal)x8___(Oh)	[Mo2]6[(C6H3)(OC6H4)3(CO2)3]8	0	5018.494152	Not in OntoMOPs KG
(4-pyramidal)x3(2-bent)x6___(D3h)	[Ni4C24H12O12S4]3[(C4H2S)(CO2)2]6	0	3586.9917	Not in OntoMOPs KG
(4-pyramidal)x3(2-bent)x6___(D3h)	[V4O8]3[(C4H2S)(CO2)2]6	0	2016.142728	Not in OntoMOPs KG
(4-pyramidal)x3(2-bent)x6___(D3h)	[V5O9]3[(C4H2S)(CO2)2]6	-3	2216.965701	Not in OntoMOPs KG
(4-pyramidal)x3(2-bent)x6___(D3h)	[Co4C48H28O4S4]3[(C4H2S)(CO2)2]6	0	3879.146796	Not in OntoMOPs KG
(4-pyramidal)x3(2-bent)x6___(D3h)	[Co4C40H44O4S4]3[(C4H2S)(CO2)2]6	0	4263.13458	Not in OntoMOPs KG
(4-pyramidal)x3(2-bent)x6___(D3h)	[Co4C40H44O12S4]3[(C4H2S)(CO2)2]6	0	4936.39986	10.1021/jacs.6b11218
(4-pyramidal)x3(2-bent)x6___(D3h)	[Ni4C40H44S4O12]3[(C4H2S)(CO2)2]6	0	4260.25698	Not in OntoMOPs KG
(4-pyramidal)x3(2-bent)x6___(D3h)	[Mg4C40H44O12S4]3[(C4H2S)(CO2)2]6	0	3847.59618	Not in OntoMOPs KG
(4-pyramidal)x3(2-bent)x6___(D3h)	[Fe4C40H44S4O4]3[(C4H2S)(CO2)2]6	0	3842.088396	Not in OntoMOPs KG
(4-pyramidal)x3(2-bent)x6___(D3h)	[Co4C24H8O24S8]3[(C4H2S)(CO2)2]6	0	3589.8693	Not in OntoMOPs KG
(4-pyramidal)x3(2-bent)x6___(D3h)	[Co4C24H12O12S4]3[(C4H2S)(CO2)2]6	0	4119.027756	Not in OntoMOPs KG
(4-pyramidal)x6(2-linear)x12___(Oh)	[Ni4C24H12O12S4]6[(C6H4)(CO2)2]12	0	7101.64812	Not in OntoMOPs KG
(4-pyramidal)x6(2-linear)x12___(Oh)	[Ni4C24H12O12S4]6[(C6H4)2(CO2)2]12	0	8014.79436	Not in OntoMOPs KG

(4-pyramidal)x6(2-linear)x12___(Oh)	[Ni4C24H12O12S4]6[(C6H3NH2)(CO2)2]12	0	7281.82248	Not in OntoMOPs KG
(4-pyramidal)x6(2-linear)x12___(Oh)	[Ni4C24H12O12S4]6[(C6H4)(C3H2N2)2]12	24	7606.695672	Not in OntoMOPs KG
(4-pyramidal)x6(2-linear)x12___(Oh)	[Ni4C24H12O12S4]6[(C6H3Br)(CO2)2]12	0	8048.40216	Not in OntoMOPs KG
(4-pyramidal)x6(2-linear)x12___(Oh)	[Ni4C24H12O12S4]6[(C6H4C)2(CO2)2]12	0	8303.05116	Not in OntoMOPs KG
(4-pyramidal)x6(2-linear)x12___(Oh)	[Ni4C24H12O12S4]6[C2O4]12	0	6188.50188	Not in OntoMOPs KG
(4-pyramidal)x6(2-linear)x12___(Oh)	[Ni4C24H12O12S4]6[(C16H12)(CO2)2]12	0	8639.6838	Not in OntoMOPs KG
(4-pyramidal)x6(2-linear)x12___(Oh)	[Ni4C24H12O12S4]6[(C5H3N)2(CO2)2]12	0	8038.51044	Not in OntoMOPs KG
(4-pyramidal)x6(2-linear)x12___(Oh)	[Ni4C24H12O12S4]6[CuCl2(C5H3N)2(CO2)2]12	0	9651.93444	Not in OntoMOPs KG
(4-pyramidal)x6(2-linear)x12___(Oh)	[Ni4C24H12O12S4]6[PdCl2(C5H3N)2(CO2)2]12	0	10166.42244	Not in OntoMOPs KG
(4-pyramidal)x6(2-linear)x12___(Oh)	[Ni4C24H12O12S4]6[(C6H4)3(CO2)2]12	0	8927.9406	Not in OntoMOPs KG
(4-pyramidal)x6(2-linear)x12___(Oh)	[Ni4C24H12O12S4]6[(C28H34N2O2)Mn(CO2)2]12	0	11004.79838	Not in OntoMOPs KG
(4-pyramidal)x6(2-linear)x12___(Oh)	[Ni4C24H12O12S4]6[(C10H6)(CO2)]12	0	7702.34964	Not in OntoMOPs KG
(4-pyramidal)x6(2-linear)x12___(Oh)	[V4O8]6[(C6H4)(CO2)2]12	0	3959.950176	Not in OntoMOPs KG
(4-pyramidal)x6(2-linear)x12___(Oh)	[V4O8]6[(C6H4)2(CO2)2]12	0	4873.096416	Not in OntoMOPs KG
(4-pyramidal)x6(2-linear)x12___(Oh)	[V4O8]6[(C6H3NH2)(CO2)2]12	0	4140.124536	Not in OntoMOPs KG
(4-pyramidal)x6(2-linear)x12___(Oh)	[V4O8]6[(C6H4)(C3H2N2)2]12	24	4464.997728	Not in OntoMOPs KG
(4-pyramidal)x6(2-linear)x12___(Oh)	[V4O8]6[(C6H3Br)(CO2)2]12	0	4906.704216	Not in OntoMOPs KG
(4-pyramidal)x6(2-linear)x12___(Oh)	[V4O8]6[(C6H4C)2(CO2)2]12	0	5161.353216	Not in OntoMOPs KG
(4-pyramidal)x6(2-linear)x12___(Oh)	[V4O8]6[C2O4]12	0	3046.803936	Not in OntoMOPs KG
(4-pyramidal)x6(2-linear)x12___(Oh)	[V4O8]6[(C16H12)(CO2)2]12	0	5497.985856	Not in OntoMOPs KG
(4-pyramidal)x6(2-linear)x12___(Oh)	[V4O8]6[(C5H3N)2(CO2)2]12	0	4896.812496	Not in OntoMOPs KG
(4-pyramidal)x6(2-linear)x12___(Oh)	[V4O8]6[CuCl2(C5H3N)2(CO2)2]12	0	6510.236496	Not in OntoMOPs KG
(4-pyramidal)x6(2-linear)x12___(Oh)	[V4O8]6[PdCl2(C5H3N)2(CO2)2]12	0	7024.724496	Not in OntoMOPs KG
(4-pyramidal)x6(2-linear)x12___(Oh)	[V4O8]6[(C6H4)3(CO2)2]12	0	5786.242656	Not in OntoMOPs KG
(4-pyramidal)x6(2-linear)x12___(Oh)	[V4O8]6[(C28H34N2O2)Mn(CO2)2]12	0	7863.10044	Not in OntoMOPs KG
(4-pyramidal)x6(2-linear)x12___(Oh)	[V4O8]6[(C10H6)(CO2)]12	0	4560.651696	Not in OntoMOPs KG
(4-pyramidal)x6(2-linear)x12___(Oh)	[V5O9]6[(C6H4)(CO2)2]12	-6	4361.596122	10.1039/C5DT04764A
(4-pyramidal)x6(2-linear)x12___(Oh)	[V5O9]6[(C6H4)2(CO2)2]12	-6	5274.742362	Not in OntoMOPs KG
(4-pyramidal)x6(2-linear)x12___(Oh)	[V5O9]6[(C6H3NH2)(CO2)2]12	-6	4541.770482	10.1039/C5DT04764A
(4-pyramidal)x6(2-linear)x12___(Oh)	[V5O9]6[(C6H4)(C3H2N2)2]12	18	4866.643674	Not in OntoMOPs KG
(4-pyramidal)x6(2-linear)x12___(Oh)	[V5O9]6[(C6H3Br)(CO2)2]12	-6	5308.350162	10.1039/C5DT04764A
(4-pyramidal)x6(2-linear)x12___(Oh)	[V5O9]6[(C6H4C)2(CO2)2]12	-6	5562.999162	Not in OntoMOPs KG
(4-pyramidal)x6(2-linear)x12___(Oh)	[V5O9]6[C2O4]12	-6	3448.449882	Not in OntoMOPs KG
(4-pyramidal)x6(2-linear)x12___(Oh)	[V5O9]6[(C16H12)(CO2)2]12	-6	5899.631802	Not in OntoMOPs KG
(4-pyramidal)x6(2-linear)x12___(Oh)	[V5O9]6[(C5H3N)2(CO2)2]12	-6	5298.458442	Not in OntoMOPs KG
(4-pyramidal)x6(2-linear)x12___(Oh)	[V5O9]6[CuCl2(C5H3N)2(CO2)2]12	-6	6911.882442	Not in OntoMOPs KG
(4-pyramidal)x6(2-linear)x12___(Oh)	[V5O9]6[PdCl2(C5H3N)2(CO2)2]12	-6	7426.370442	Not in OntoMOPs KG
(4-pyramidal)x6(2-linear)x12___(Oh)	[V5O9]6[(C6H4)3(CO2)2]12	-6	6187.888602	Not in OntoMOPs KG
(4-pyramidal)x6(2-linear)x12___(Oh)	[V5O9]6[(C28H34N2O2)Mn(CO2)2]12	-6	8264.746386	Not in OntoMOPs KG
(4-pyramidal)x6(2-linear)x12___(Oh)	[V5O9]6[(C10H6)(CO2)]12	-6	4962.297642	Not in OntoMOPs KG
(4-pyramidal)x6(2-linear)x12___(Oh)	[Co4C48H28O4S4]6[(C6H4)(CO2)2]12	0	7685.958312	Not in OntoMOPs KG
(4-pyramidal)x6(2-linear)x12___(Oh)	[Co4C48H28O4S4]6[(C6H4)2(CO2)2]12	0	8599.104552	Not in OntoMOPs KG
(4-pyramidal)x6(2-linear)x12___(Oh)	[Co4C48H28O4S4]6[(C6H3NH2)(CO2)2]12	0	7866.132672	Not in OntoMOPs KG
(4-pyramidal)x6(2-linear)x12___(Oh)	[Co4C48H28O4S4]6[(C6H4)(C3H2N2)2]12	24	8191.005864	Not in OntoMOPs KG
(4-pyramidal)x6(2-linear)x12___(Oh)	[Co4C48H28O4S4]6[(C6H3Br)(CO2)2]12	0	8632.712352	Not in OntoMOPs KG
(4-pyramidal)x6(2-linear)x12___(Oh)	[Co4C48H28O4S4]6[(C6H4C)2(CO2)2]12	0	8887.361352	Not in OntoMOPs KG
(4-pyramidal)x6(2-linear)x12___(Oh)	[Co4C48H28O4S4]6[C2O4]12	0	6772.812072	Not in OntoMOPs KG
(4-pyramidal)x6(2-linear)x12___(Oh)	[Co4C48H28O4S4]6[(C16H12)(CO2)2]12	0	9223.993992	Not in OntoMOPs KG
(4-pyramidal)x6(2-linear)x12___(Oh)	[Co4C48H28O4S4]6[(C5H3N)2(CO2)2]12	0	8622.820632	Not in OntoMOPs KG
(4-pyramidal)x6(2-linear)x12___(Oh)	[Co4C48H28O4S4]6[CuCl2(C5H3N)2(CO2)2]12	0	10236.24463	Not in OntoMOPs KG

(4-pyramidal)x6(2-linear)x12___(Oh)	[Co4C48H28O4S4]6[PdCl2(C5H3N)2(CO2)2]12	0	10750.73263	Not in OntoMOPs KG
(4-pyramidal)x6(2-linear)x12___(Oh)	[Co4C48H28O4S4]6[(C6H4)3(CO2)2]12	0	9512.250792	Not in OntoMOPs KG
(4-pyramidal)x6(2-linear)x12___(Oh)	[Co4C48H28O4S4]6[(C28H34N2O2)Mn(CO2)2]12	0	11589.10858	Not in OntoMOPs KG
(4-pyramidal)x6(2-linear)x12___(Oh)	[Co4C48H28O4S4]6[(C10H6)(CO2)]12	0	8286.659832	Not in OntoMOPs KG
(4-pyramidal)x6(2-linear)x12___(Oh)	[Co4C40H44O4S4]6[(C6H4)(CO2)2]12	0	8453.93388	10.1021/ja502839b
(4-pyramidal)x6(2-linear)x12___(Oh)	[Co4C40H44O4S4]6[(C6H4)2(CO2)2]12	0	9367.08012	Not in OntoMOPs KG
(4-pyramidal)x6(2-linear)x12___(Oh)	[Co4C40H44O4S4]6[(C6H3NH2)(CO2)2]12	0	8634.10824	Not in OntoMOPs KG
(4-pyramidal)x6(2-linear)x12___(Oh)	[Co4C40H44O4S4]6[(C6H4)(C3H2N2)2]12	24	8958.981432	Not in OntoMOPs KG
(4-pyramidal)x6(2-linear)x12___(Oh)	[Co4C40H44O4S4]6[(C6H3Br)(CO2)2]12	0	9400.68792	Not in OntoMOPs KG
(4-pyramidal)x6(2-linear)x12___(Oh)	[Co4C40H44O4S4]6[(C6H4C)2(CO2)2]12	0	9655.33692	Not in OntoMOPs KG
(4-pyramidal)x6(2-linear)x12___(Oh)	[Co4C40H44O4S4]6[C2O4]12	0	7540.78764	Not in OntoMOPs KG
(4-pyramidal)x6(2-linear)x12___(Oh)	[Co4C40H44O4S4]6[(C16H12)(CO2)2]12	0	9991.96956	Not in OntoMOPs KG
(4-pyramidal)x6(2-linear)x12___(Oh)	[Co4C40H44O4S4]6[(C5H3N)2(CO2)2]12	0	9390.7962	Not in OntoMOPs KG
(4-pyramidal)x6(2-linear)x12___(Oh)	[Co4C40H44O4S4]6[CuCl2(C5H3N)2(CO2)2]12	0	11004.2202	Not in OntoMOPs KG
(4-pyramidal)x6(2-linear)x12___(Oh)	[Co4C40H44O4S4]6[PdCl2(C5H3N)2(CO2)2]12	0	11518.7082	Not in OntoMOPs KG
(4-pyramidal)x6(2-linear)x12___(Oh)	[Co4C40H44O4S4]6[(C6H4)3(CO2)2]12	0	10280.22636	Not in OntoMOPs KG
(4-pyramidal)x6(2-linear)x12___(Oh)	[Co4C40H44O4S4]6[(C28H34N2O2)Mn(CO2)2]12	0	12357.08414	Not in OntoMOPs KG
(4-pyramidal)x6(2-linear)x12___(Oh)	[Co4C40H44O4S4]6[(C10H6)(CO2)]12	0	9054.6354	Not in OntoMOPs KG
(4-pyramidal)x6(2-linear)x12___(Oh)	[Co4C40H44O12S4]6[(C6H4)(CO2)2]12	0	9800.46444	Not in OntoMOPs KG
(4-pyramidal)x6(2-linear)x12___(Oh)	[Co4C40H44O12S4]6[(C6H4)2(CO2)2]12	0	10713.61068	Not in OntoMOPs KG
(4-pyramidal)x6(2-linear)x12___(Oh)	[Co4C40H44O12S4]6[(C6H3NH2)(CO2)2]12	0	9980.6388	Not in OntoMOPs KG
(4-pyramidal)x6(2-linear)x12___(Oh)	[Co4C40H44O12S4]6[(C6H4)(C3H2N2)2]12	24	10305.51199	Not in OntoMOPs KG
(4-pyramidal)x6(2-linear)x12___(Oh)	[Co4C40H44O12S4]6[(C6H3Br)(CO2)2]12	0	10747.21848	Not in OntoMOPs KG
(4-pyramidal)x6(2-linear)x12___(Oh)	[Co4C40H44O12S4]6[(C6H4C)2(CO2)2]12	0	11001.86748	Not in OntoMOPs KG
(4-pyramidal)x6(2-linear)x12___(Oh)	[Co4C40H44O12S4]6[C2O4]12	0	8887.3182	Not in OntoMOPs KG
(4-pyramidal)x6(2-linear)x12___(Oh)	[Co4C40H44O12S4]6[(C16H12)(CO2)2]12	0	11338.50012	Not in OntoMOPs KG
(4-pyramidal)x6(2-linear)x12___(Oh)	[Co4C40H44O12S4]6[(C5H3N)2(CO2)2]12	0	10737.32676	Not in OntoMOPs KG
(4-pyramidal)x6(2-linear)x12___(Oh)	[Co4C40H44O12S4]6[CuCl2(C5H3N)2(CO2)2]12	0	12350.75076	Not in OntoMOPs KG
(4-pyramidal)x6(2-linear)x12___(Oh)	[Co4C40H44O12S4]6[PdCl2(C5H3N)2(CO2)2]12	0	12865.23876	Not in OntoMOPs KG
(4-pyramidal)x6(2-linear)x12___(Oh)	[Co4C40H44O12S4]6[(C6H4)3(CO2)2]12	0	11626.75692	Not in OntoMOPs KG
(4-pyramidal)x6(2-linear)x12___(Oh)	[Co4C40H44O12S4]6[(C28H34N2O2)Mn(CO2)2]12	0	13703.6147	Not in OntoMOPs KG
(4-pyramidal)x6(2-linear)x12___(Oh)	[Co4C40H44O12S4]6[(C10H6)(CO2)]12	0	10401.16596	Not in OntoMOPs KG
(4-pyramidal)x6(2-linear)x12___(Oh)	[Ni4C40H44S4O12]6[(C6H4)(CO2)2]12	0	8448.17868	10.1021/ja502839b
(4-pyramidal)x6(2-linear)x12___(Oh)	[Ni4C40H44S4O12]6[(C6H4)2(CO2)2]12	0	9361.32492	Not in OntoMOPs KG
(4-pyramidal)x6(2-linear)x12___(Oh)	[Ni4C40H44S4O12]6[(C6H3NH2)(CO2)2]12	0	8628.35304	Not in OntoMOPs KG
(4-pyramidal)x6(2-linear)x12___(Oh)	[Ni4C40H44S4O12]6[(C6H4)(C3H2N2)2]12	24	8953.226232	Not in OntoMOPs KG
(4-pyramidal)x6(2-linear)x12___(Oh)	[Ni4C40H44S4O12]6[(C6H3Br)(CO2)2]12	0	9394.93272	Not in OntoMOPs KG
(4-pyramidal)x6(2-linear)x12___(Oh)	[Ni4C40H44S4O12]6[(C6H4C)2(CO2)2]12	0	9649.58172	Not in OntoMOPs KG
(4-pyramidal)x6(2-linear)x12___(Oh)	[Ni4C40H44S4O12]6[C2O4]12	0	7535.03244	Not in OntoMOPs KG
(4-pyramidal)x6(2-linear)x12___(Oh)	[Ni4C40H44S4O12]6[(C16H12)(CO2)2]12	0	9986.21436	Not in OntoMOPs KG
(4-pyramidal)x6(2-linear)x12___(Oh)	[Ni4C40H44S4O12]6[(C5H3N)2(CO2)2]12	0	9385.041	Not in OntoMOPs KG
(4-pyramidal)x6(2-linear)x12___(Oh)	[Ni4C40H44S4O12]6[CuCl2(C5H3N)2(CO2)2]12	0	10998.465	Not in OntoMOPs KG
(4-pyramidal)x6(2-linear)x12___(Oh)	[Ni4C40H44S4O12]6[PdCl2(C5H3N)2(CO2)2]12	0	11512.953	Not in OntoMOPs KG
(4-pyramidal)x6(2-linear)x12___(Oh)	[Ni4C40H44S4O12]6[(C6H4)3(CO2)2]12	0	10274.47116	Not in OntoMOPs KG
(4-pyramidal)x6(2-linear)x12___(Oh)	[Ni4C40H44S4O12]6[(C28H34N2O2)Mn(CO2)2]12	0	12351.32894	Not in OntoMOPs KG
(4-pyramidal)x6(2-linear)x12___(Oh)	[Ni4C40H44S4O12]6[(C10H6)(CO2)]12	0	9048.8802	Not in OntoMOPs KG
(4-pyramidal)x6(2-linear)x12___(Oh)	[Mg4C40H44O12S4]6[(C6H4)(CO2)2]12	0	7622.85708	Not in OntoMOPs KG
(4-pyramidal)x6(2-linear)x12___(Oh)	[Mg4C40H44O12S4]6[(C6H4)2(CO2)2]12	0	8536.00332	Not in OntoMOPs KG
(4-pyramidal)x6(2-linear)x12___(Oh)	[Mg4C40H44O12S4]6[(C6H3NH2)(CO2)2]12	0	7803.03144	Not in OntoMOPs KG
(4-pyramidal)x6(2-linear)x12___(Oh)	[Mg4C40H44O12S4]6[(C6H4)(C3H2N2)2]12	24	8127.904632	Not in OntoMOPs KG

(4-pyramidal)x6(2-linear)x12___(Oh)	[Mg4C40H44O12S4]6[(C6H3Br)(CO2)2]12	0	8569.61112	Not in OntoMOPs KG
(4-pyramidal)x6(2-linear)x12___(Oh)	[Mg4C40H44O12S4]6[(C6H4C)2(CO2)2]12	0	8824.26012	Not in OntoMOPs KG
(4-pyramidal)x6(2-linear)x12___(Oh)	[Mg4C40H44O12S4]6[C2O4]12	0	6709.71084	Not in OntoMOPs KG
(4-pyramidal)x6(2-linear)x12___(Oh)	[Mg4C40H44O12S4]6[(C16H12)(CO2)2]12	0	9160.89276	Not in OntoMOPs KG
(4-pyramidal)x6(2-linear)x12___(Oh)	[Mg4C40H44O12S4]6[(C5H3N)2(CO2)2]12	0	8559.7194	Not in OntoMOPs KG
(4-pyramidal)x6(2-linear)x12___(Oh)	[Mg4C40H44O12S4]6[CuCl2(C5H3N)2(CO2)2]12	0	10173.1434	Not in OntoMOPs KG
(4-pyramidal)x6(2-linear)x12___(Oh)	[Mg4C40H44O12S4]6[PdCl2(C5H3N)2(CO2)2]12	0	10687.6314	Not in OntoMOPs KG
(4-pyramidal)x6(2-linear)x12___(Oh)	[Mg4C40H44O12S4]6[(C6H4)3(CO2)2]12	0	9449.14956	Not in OntoMOPs KG
(4-pyramidal)x6(2-linear)x12___(Oh)	[Mg4C40H44O12S4]6[(C28H34N2O2)Mn(CO2)2]12	0	11526.00734	Not in OntoMOPs KG
(4-pyramidal)x6(2-linear)x12___(Oh)	[Mg4C40H44O12S4]6[(C10H6)(CO2)]12	0	8223.5586	Not in OntoMOPs KG
(4-pyramidal)x6(2-linear)x12___(Oh)	[Fe4C40H44S4O4]6[(C6H4)(CO2)2]12	0	7611.841512	Not in OntoMOPs KG
(4-pyramidal)x6(2-linear)x12___(Oh)	[Fe4C40H44S4O4]6[(C6H4)2(CO2)2]12	0	8524.987752	Not in OntoMOPs KG
(4-pyramidal)x6(2-linear)x12___(Oh)	[Fe4C40H44S4O4]6[(C6H3NH2)(CO2)2]12	0	7792.015872	Not in OntoMOPs KG
(4-pyramidal)x6(2-linear)x12___(Oh)	[Fe4C40H44S4O4]6[(C6H4)(C3H2N2)2]12	24	8116.889064	Not in OntoMOPs KG
(4-pyramidal)x6(2-linear)x12___(Oh)	[Fe4C40H44S4O4]6[(C6H3Br)(CO2)2]12	0	8558.595552	Not in OntoMOPs KG
(4-pyramidal)x6(2-linear)x12___(Oh)	[Fe4C40H44S4O4]6[(C6H4C)2(CO2)2]12	0	8813.244552	Not in OntoMOPs KG
(4-pyramidal)x6(2-linear)x12___(Oh)	[Fe4C40H44S4O4]6[C2O4]12	0	6698.695272	Not in OntoMOPs KG
(4-pyramidal)x6(2-linear)x12___(Oh)	[Fe4C40H44S4O4]6[(C16H12)(CO2)2]12	0	9149.877192	Not in OntoMOPs KG
(4-pyramidal)x6(2-linear)x12___(Oh)	[Fe4C40H44S4O4]6[(C5H3N)2(CO2)2]12	0	8548.703832	Not in OntoMOPs KG
(4-pyramidal)x6(2-linear)x12___(Oh)	[Fe4C40H44S4O4]6[CuCl2(C5H3N)2(CO2)2]12	0	10162.12783	Not in OntoMOPs KG
(4-pyramidal)x6(2-linear)x12___(Oh)	[Fe4C40H44S4O4]6[PdCl2(C5H3N)2(CO2)2]12	0	10676.61583	Not in OntoMOPs KG
(4-pyramidal)x6(2-linear)x12___(Oh)	[Fe4C40H44S4O4]6[(C6H4)3(CO2)2]12	0	9438.133992	Not in OntoMOPs KG
(4-pyramidal)x6(2-linear)x12___(Oh)	[Fe4C40H44S4O4]6[(C28H34N2O2)Mn(CO2)2]12	0	11514.99178	Not in OntoMOPs KG
(4-pyramidal)x6(2-linear)x12___(Oh)	[Fe4C40H44S4O4]6[(C10H6)(CO2)]12	0	8212.543032	Not in OntoMOPs KG
(4-pyramidal)x6(2-linear)x12___(Oh)	[Co4C24H8O24S8]6[(C6H4)(CO2)2]12	0	7107.40332	Not in OntoMOPs KG
(4-pyramidal)x6(2-linear)x12___(Oh)	[Co4C24H8O24S8]6[(C6H4)2(CO2)2]12	0	8020.54956	Not in OntoMOPs KG
(4-pyramidal)x6(2-linear)x12___(Oh)	[Co4C24H8O24S8]6[(C6H3NH2)(CO2)2]12	0	7287.57768	Not in OntoMOPs KG
(4-pyramidal)x6(2-linear)x12___(Oh)	[Co4C24H8O24S8]6[(C6H4)(C3H2N2)2]12	24	7612.450872	Not in OntoMOPs KG
(4-pyramidal)x6(2-linear)x12___(Oh)	[Co4C24H8O24S8]6[(C6H3Br)(CO2)2]12	0	8054.15736	Not in OntoMOPs KG
(4-pyramidal)x6(2-linear)x12___(Oh)	[Co4C24H8O24S8]6[(C6H4C)2(CO2)2]12	0	8308.80636	Not in OntoMOPs KG
(4-pyramidal)x6(2-linear)x12___(Oh)	[Co4C24H8O24S8]6[C2O4]12	0	6194.25708	Not in OntoMOPs KG
(4-pyramidal)x6(2-linear)x12___(Oh)	[Co4C24H8O24S8]6[(C16H12)(CO2)2]12	0	8645.439	Not in OntoMOPs KG
(4-pyramidal)x6(2-linear)x12___(Oh)	[Co4C24H8O24S8]6[(C5H3N)2(CO2)2]12	0	8044.26564	Not in OntoMOPs KG
(4-pyramidal)x6(2-linear)x12___(Oh)	[Co4C24H8O24S8]6[CuCl2(C5H3N)2(CO2)2]12	0	9657.68964	Not in OntoMOPs KG
(4-pyramidal)x6(2-linear)x12___(Oh)	[Co4C24H8O24S8]6[PdCl2(C5H3N)2(CO2)2]12	0	10172.17764	Not in OntoMOPs KG
(4-pyramidal)x6(2-linear)x12___(Oh)	[Co4C24H8O24S8]6[(C6H4)3(CO2)2]12	0	8933.6958	Not in OntoMOPs KG
(4-pyramidal)x6(2-linear)x12___(Oh)	[Co4C24H8O24S8]6[(C28H34N2O2)Mn(CO2)2]12	0	11010.55358	Not in OntoMOPs KG
(4-pyramidal)x6(2-linear)x12___(Oh)	[Co4C24H8O24S8]6[(C10H6)(CO2)]12	0	7708.10484	Not in OntoMOPs KG
(4-pyramidal)x6(2-linear)x12___(Oh)	[Zn4C40H44O12S4]6[(C6H4)(CO2)2]12	0	8608.89708	Not in OntoMOPs KG
(4-pyramidal)x6(2-linear)x12___(Oh)	[Zn4C40H44O12S4]6[(C6H4)2(CO2)2]12	0	9522.04332	Not in OntoMOPs KG
(4-pyramidal)x6(2-linear)x12___(Oh)	[Zn4C40H44O12S4]6[(C6H3NH2)(CO2)2]12	0	8789.07144	Not in OntoMOPs KG
(4-pyramidal)x6(2-linear)x12___(Oh)	[Zn4C40H44O12S4]6[(C6H4)(C3H2N2)2]12	24	9113.944632	Not in OntoMOPs KG
(4-pyramidal)x6(2-linear)x12___(Oh)	[Zn4C40H44O12S4]6[(C6H3Br)(CO2)2]12	0	9555.65112	Not in OntoMOPs KG
(4-pyramidal)x6(2-linear)x12___(Oh)	[Zn4C40H44O12S4]6[(C6H4C)2(CO2)2]12	0	9810.30012	Not in OntoMOPs KG
(4-pyramidal)x6(2-linear)x12___(Oh)	[Zn4C40H44O12S4]6[C2O4]12	0	7695.75084	Not in OntoMOPs KG
(4-pyramidal)x6(2-linear)x12___(Oh)	[Zn4C40H44O12S4]6[(C16H12)(CO2)2]12	0	10146.93276	Not in OntoMOPs KG
(4-pyramidal)x6(2-linear)x12___(Oh)	[Zn4C40H44O12S4]6[(C5H3N)2(CO2)2]12	0	9545.7594	Not in OntoMOPs KG
(4-pyramidal)x6(2-linear)x12___(Oh)	[Zn4C40H44O12S4]6[CuCl2(C5H3N)2(CO2)2]12	0	11159.1834	Not in OntoMOPs KG
(4-pyramidal)x6(2-linear)x12___(Oh)	[Zn4C40H44O12S4]6[PdCl2(C5H3N)2(CO2)2]12	0	11673.6714	Not in OntoMOPs KG
(4-pyramidal)x6(2-linear)x12___(Oh)	[Zn4C40H44O12S4]6[(C6H4)3(CO2)2]12	0	10435.18956	Not in OntoMOPs KG

(4-pyramidal)x6(2-linear)x12___(Oh)	[Zn4C40H44O12S4]6[(C28H34N2O2)Mn(CO2)2]12	0	12512.04734	10.1002/anie.201711310
(4-pyramidal)x6(2-linear)x12___(Oh)	[Zn4C40H44O12S4]6[(C10H6)(CO2)]12	0	9209.5986	Not in OntoMOPs KG
(4-pyramidal)x6(2-linear)x12___(Oh)	[Co4C56H76O12S4]6[(C6H4)(CO2)2]12	-24	9004.738752	10.1021/ja502839b
(4-pyramidal)x6(2-linear)x12___(Oh)	[Co4C56H76O12S4]6[(C6H4)2(CO2)2]12	-24	9917.884992	Not in OntoMOPs KG
(4-pyramidal)x6(2-linear)x12___(Oh)	[Co4C56H76O12S4]6[(C6H3NH2)(CO2)2]12	-24	9184.913112	Not in OntoMOPs KG
(4-pyramidal)x6(2-linear)x12___(Oh)	[Co4C56H76O12S4]6[(C6H4)(C3H2N2)2]12	0	9509.786304	Not in OntoMOPs KG
(4-pyramidal)x6(2-linear)x12___(Oh)	[Co4C56H76O12S4]6[(C6H3Br)(CO2)2]12	-24	9951.492792	Not in OntoMOPs KG
(4-pyramidal)x6(2-linear)x12___(Oh)	[Co4C56H76O12S4]6[(C6H4C)2(CO2)2]12	-24	10206.14179	Not in OntoMOPs KG
(4-pyramidal)x6(2-linear)x12___(Oh)	[Co4C56H76O12S4]6[C2O4]12	-24	8091.592512	Not in OntoMOPs KG
(4-pyramidal)x6(2-linear)x12___(Oh)	[Co4C56H76O12S4]6[(C16H12)(CO2)2]12	-24	10542.77443	Not in OntoMOPs KG
(4-pyramidal)x6(2-linear)x12___(Oh)	[Co4C56H76O12S4]6[(C5H3N)2(CO2)2]12	-24	9941.601072	Not in OntoMOPs KG
(4-pyramidal)x6(2-linear)x12___(Oh)	[Co4C56H76O12S4]6[CuCl2(C5H3N)2(CO2)2]12	-24	11555.02507	Not in OntoMOPs KG
(4-pyramidal)x6(2-linear)x12___(Oh)	[Co4C56H76O12S4]6[PdCl2(C5H3N)2(CO2)2]12	-24	12069.51307	Not in OntoMOPs KG
(4-pyramidal)x6(2-linear)x12___(Oh)	[Co4C56H76O12S4]6[(C6H4)3(CO2)2]12	-24	10831.03123	Not in OntoMOPs KG
(4-pyramidal)x6(2-linear)x12___(Oh)	[Co4C56H76O12S4]6[(C28H34N2O2)Mn(CO2)2]12	-24	12907.88902	Not in OntoMOPs KG
(4-pyramidal)x6(2-linear)x12___(Oh)	[Co4C56H76O12S4]6[(C10H6)(CO2)]12	-24	9605.440272	Not in OntoMOPs KG
(4-pyramidal)x6(2-linear)x12___(Oh)	[Mg4C56H76O12S4]6[(C6H4)(CO2)2]12	0	8969.38764	10.1021/ja502839b
(4-pyramidal)x6(2-linear)x12___(Oh)	[Mg4C56H76O12S4]6[(C6H4)2(CO2)2]12	0	9882.53388	Not in OntoMOPs KG
(4-pyramidal)x6(2-linear)x12___(Oh)	[Mg4C56H76O12S4]6[(C6H3NH2)(CO2)2]12	0	9149.562	Not in OntoMOPs KG
(4-pyramidal)x6(2-linear)x12___(Oh)	[Mg4C56H76O12S4]6[(C6H4)(C3H2N2)2]12	24	9474.435192	Not in OntoMOPs KG
(4-pyramidal)x6(2-linear)x12___(Oh)	[Mg4C56H76O12S4]6[(C6H3Br)(CO2)2]12	0	9916.14168	Not in OntoMOPs KG
(4-pyramidal)x6(2-linear)x12___(Oh)	[Mg4C56H76O12S4]6[(C6H4C)2(CO2)2]12	0	10170.79068	Not in OntoMOPs KG
(4-pyramidal)x6(2-linear)x12___(Oh)	[Mg4C56H76O12S4]6[C2O4]12	0	8056.2414	Not in OntoMOPs KG
(4-pyramidal)x6(2-linear)x12___(Oh)	[Mg4C56H76O12S4]6[(C16H12)(CO2)2]12	0	10507.42332	Not in OntoMOPs KG
(4-pyramidal)x6(2-linear)x12___(Oh)	[Mg4C56H76O12S4]6[(C5H3N)2(CO2)2]12	0	9906.24996	Not in OntoMOPs KG
(4-pyramidal)x6(2-linear)x12___(Oh)	[Mg4C56H76O12S4]6[CuCl2(C5H3N)2(CO2)2]12	0	11519.67396	Not in OntoMOPs KG
(4-pyramidal)x6(2-linear)x12___(Oh)	[Mg4C56H76O12S4]6[PdCl2(C5H3N)2(CO2)2]12	0	12034.16196	Not in OntoMOPs KG
(4-pyramidal)x6(2-linear)x12___(Oh)	[Mg4C56H76O12S4]6[(C6H4)3(CO2)2]12	0	10795.68012	Not in OntoMOPs KG
(4-pyramidal)x6(2-linear)x12___(Oh)	[Mg4C56H76O12S4]6[(C28H34N2O2)Mn(CO2)2]12	0	12872.5379	Not in OntoMOPs KG
(4-pyramidal)x6(2-linear)x12___(Oh)	[Mg4C56H76O12S4]6[(C10H6)(CO2)]12	0	9570.08916	Not in OntoMOPs KG
(4-pyramidal)x6(2-linear)x12___(Oh)	[Ni4C56H76O12S4]6[(C6H4)(CO2)2]12	0	9794.70924	10.1021/ja502839b
(4-pyramidal)x6(2-linear)x12___(Oh)	[Ni4C56H76O12S4]6[(C6H4)2(CO2)2]12	0	10707.85548	Not in OntoMOPs KG
(4-pyramidal)x6(2-linear)x12___(Oh)	[Ni4C56H76O12S4]6[(C6H3NH2)(CO2)2]12	0	9974.8836	Not in OntoMOPs KG
(4-pyramidal)x6(2-linear)x12___(Oh)	[Ni4C56H76O12S4]6[(C6H4)(C3H2N2)2]12	24	10299.75679	Not in OntoMOPs KG
(4-pyramidal)x6(2-linear)x12___(Oh)	[Ni4C56H76O12S4]6[(C6H3Br)(CO2)2]12	0	10741.46328	Not in OntoMOPs KG
(4-pyramidal)x6(2-linear)x12___(Oh)	[Ni4C56H76O12S4]6[(C6H4C)2(CO2)2]12	0	10996.11228	Not in OntoMOPs KG
(4-pyramidal)x6(2-linear)x12___(Oh)	[Ni4C56H76O12S4]6[C2O4]12	0	8881.563	Not in OntoMOPs KG
(4-pyramidal)x6(2-linear)x12___(Oh)	[Ni4C56H76O12S4]6[(C16H12)(CO2)2]12	0	11332.74492	Not in OntoMOPs KG
(4-pyramidal)x6(2-linear)x12___(Oh)	[Ni4C56H76O12S4]6[(C5H3N)2(CO2)2]12	0	10731.57156	Not in OntoMOPs KG
(4-pyramidal)x6(2-linear)x12___(Oh)	[Ni4C56H76O12S4]6[CuCl2(C5H3N)2(CO2)2]12	0	12344.99556	Not in OntoMOPs KG
(4-pyramidal)x6(2-linear)x12___(Oh)	[Ni4C56H76O12S4]6[PdCl2(C5H3N)2(CO2)2]12	0	12859.48356	Not in OntoMOPs KG
(4-pyramidal)x6(2-linear)x12___(Oh)	[Ni4C56H76O12S4]6[(C6H4)3(CO2)2]12	0	11621.00172	Not in OntoMOPs KG
(4-pyramidal)x6(2-linear)x12___(Oh)	[Ni4C56H76O12S4]6[(C28H34N2O2)Mn(CO2)2]12	0	13697.8595	Not in OntoMOPs KG
(4-pyramidal)x6(2-linear)x12___(Oh)	[Ni4C56H76O12S4]6[(C10H6)(CO2)]12	0	10395.41076	Not in OntoMOPs KG
(4-pyramidal)x6(2-linear)x12___(Oh)	[Co4C24H12O12S4]6[(C6H4)(CO2)2]12	0	8165.720232	Not in OntoMOPs KG
(4-pyramidal)x6(2-linear)x12___(Oh)	[Co4C24H12O12S4]6[(C6H4)2(CO2)2]12	0	9078.866472	Not in OntoMOPs KG
(4-pyramidal)x6(2-linear)x12___(Oh)	[Co4C24H12O12S4]6[(C6H3NH2)(CO2)2]12	0	8345.894592	Not in OntoMOPs KG
(4-pyramidal)x6(2-linear)x12___(Oh)	[Co4C24H12O12S4]6[(C6H4)(C3H2N2)2]12	24	8670.767784	Not in OntoMOPs KG
(4-pyramidal)x6(2-linear)x12___(Oh)	[Co4C24H12O12S4]6[(C6H3Br)(CO2)2]12	0	9112.474272	Not in OntoMOPs KG
(4-pyramidal)x6(2-linear)x12___(Oh)	[Co4C24H12O12S4]6[(C6H4C)2(CO2)2]12	0	9367.123272	Not in OntoMOPs KG

(4-pyramidal)x6(2-linear)x12___(Oh)	[Co4C24H12O12S4]6[C2O4]12	0	7252.573992	Not in OntoMOPs KG
(4-pyramidal)x6(2-linear)x12___(Oh)	[Co4C24H12O12S4]6[(C16H12)(CO2)2]12	0	9703.755912	Not in OntoMOPs KG
(4-pyramidal)x6(2-linear)x12___(Oh)	[Co4C24H12O12S4]6[(C5H3N)2(CO2)2]12	0	9102.582552	Not in OntoMOPs KG
(4-pyramidal)x6(2-linear)x12___(Oh)	[Co4C24H12O12S4]6[CuCl2(C5H3N)2(CO2)2]12	0	10716.00655	Not in OntoMOPs KG
(4-pyramidal)x6(2-linear)x12___(Oh)	[Co4C24H12O12S4]6[PdCl2(C5H3N)2(CO2)2]12	0	11230.49455	Not in OntoMOPs KG
(4-pyramidal)x6(2-linear)x12___(Oh)	[Co4C24H12O12S4]6[(C6H4)3(CO2)2]12	0	9992.012712	Not in OntoMOPs KG
(4-pyramidal)x6(2-linear)x12___(Oh)	[Co4C24H12O12S4]6[(C28H34N2O2)Mn(CO2)2]12	0	12068.8705	Not in OntoMOPs KG
(4-pyramidal)x6(2-linear)x12___(Oh)	[Co4C24H12O12S4]6[(C10H6)(CO2)]12	0	8766.421752	Not in OntoMOPs KG
(4-pyramidal)x6(2-bent)x12___(D3h)	[Ni4C24H12O12S4]6[(C4H2S)(CO2)2]12	0	7173.9834	Not in OntoMOPs KG
(4-pyramidal)x6(2-bent)x12___(D3h)	[V4O8]6[(C4H2S)(CO2)2]12	0	4032.285456	Not in OntoMOPs KG
(4-pyramidal)x6(2-bent)x12___(D3h)	[V5O9]6[(C4H2S)(CO2)2]12	-6	4433.931402	Not in OntoMOPs KG
(4-pyramidal)x6(2-bent)x12___(D3h)	[Co4C48H28O4S4]6[(C4H2S)(CO2)2]12	0	7758.293592	Not in OntoMOPs KG
(4-pyramidal)x6(2-bent)x12___(D3h)	[Co4C40H44O4S4]6[(C4H2S)(CO2)2]12	0	8526.26916	Not in OntoMOPs KG
(4-pyramidal)x6(2-bent)x12___(D3h)	[Co4C40H44O12S4]6[(C4H2S)(CO2)2]12	0	9872.79972	Not in OntoMOPs KG
(4-pyramidal)x6(2-bent)x12___(D3h)	[Ni4C40H44S4O12]6[(C4H2S)(CO2)2]12	0	8520.51396	10.1021/jacs.6b11218
(4-pyramidal)x6(2-bent)x12___(D3h)	[Mg4C40H44O12S4]6[(C4H2S)(CO2)2]12	0	7695.19236	Not in OntoMOPs KG
(4-pyramidal)x6(2-bent)x12___(D3h)	[Fe4C40H44S4O4]6[(C4H2S)(CO2)2]12	0	7684.176792	Not in OntoMOPs KG
(4-pyramidal)x6(2-bent)x12___(D3h)	[Co4C24H8O24S8]6[(C4H2S)(CO2)2]12	0	7179.7386	Not in OntoMOPs KG
(4-pyramidal)x6(2-bent)x12___(D3h)	[Co4C24H12O12S4]6[(C4H2S)(CO2)2]12	0	8238.055512	Not in OntoMOPs KG
(4-pyramidal)x6(2-bent)x12___(D3h)	[Zn4C40H44O12S4]6[(C4H2S)(CO2)2]12	0	8681.23236	Not in OntoMOPs KG
(4-pyramidal)x6(2-bent)x12___(D3h)	[Co4C56H76O12S4]6[(C4H2S)(CO2)2]12	-24	9077.074032	Not in OntoMOPs KG
(4-pyramidal)x6(2-bent)x12___(D3h)	[Mg4C56H76O12S4]6[(C4H2S)(CO2)2]12	0	9041.72292	Not in OntoMOPs KG
(4-pyramidal)x6(2-bent)x12___(D3h)	[Ni4C56H76O12S4]6[(C4H2S)(CO2)2]12	0	9867.04452	Not in OntoMOPs KG

7. Algorithmic Output II - Visual Construction of new MOPs

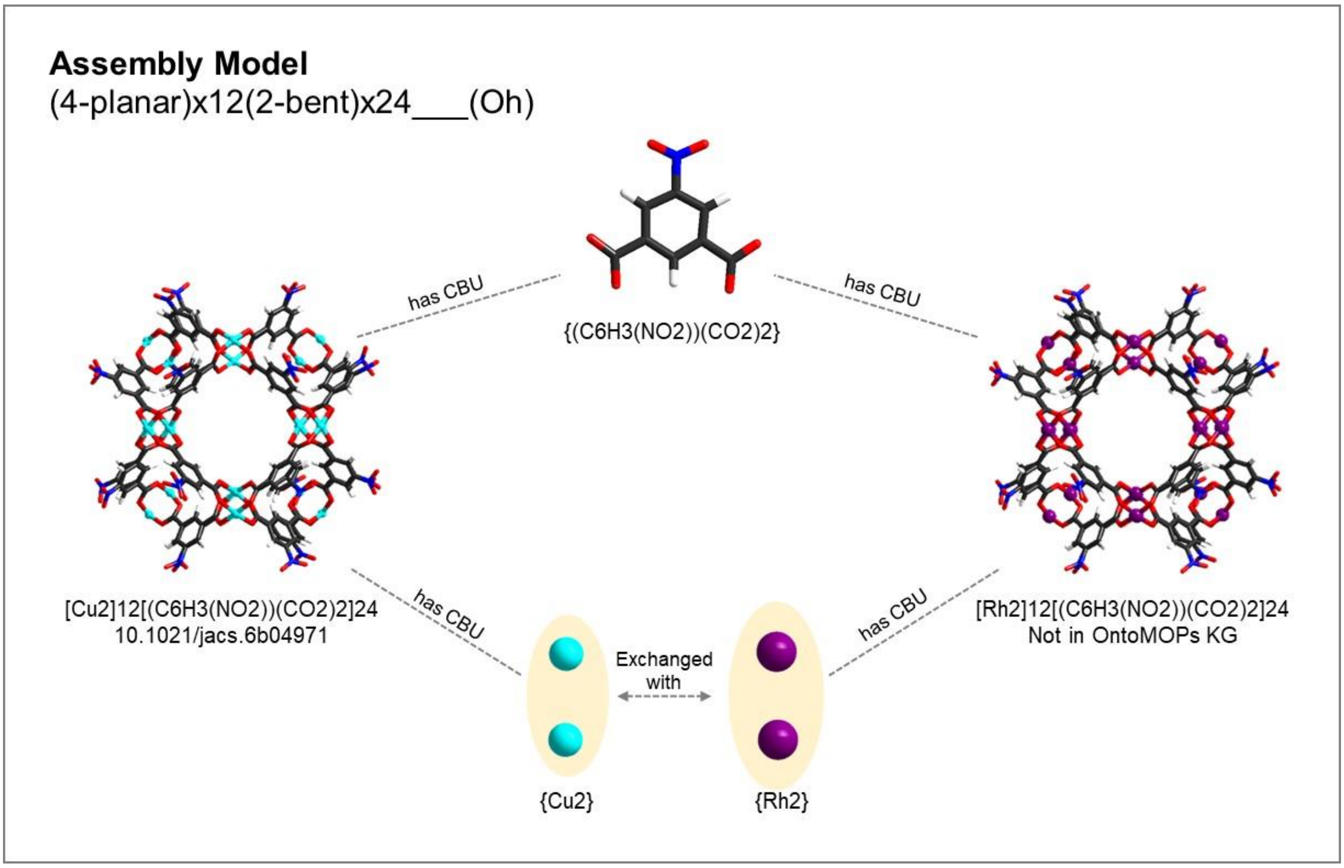

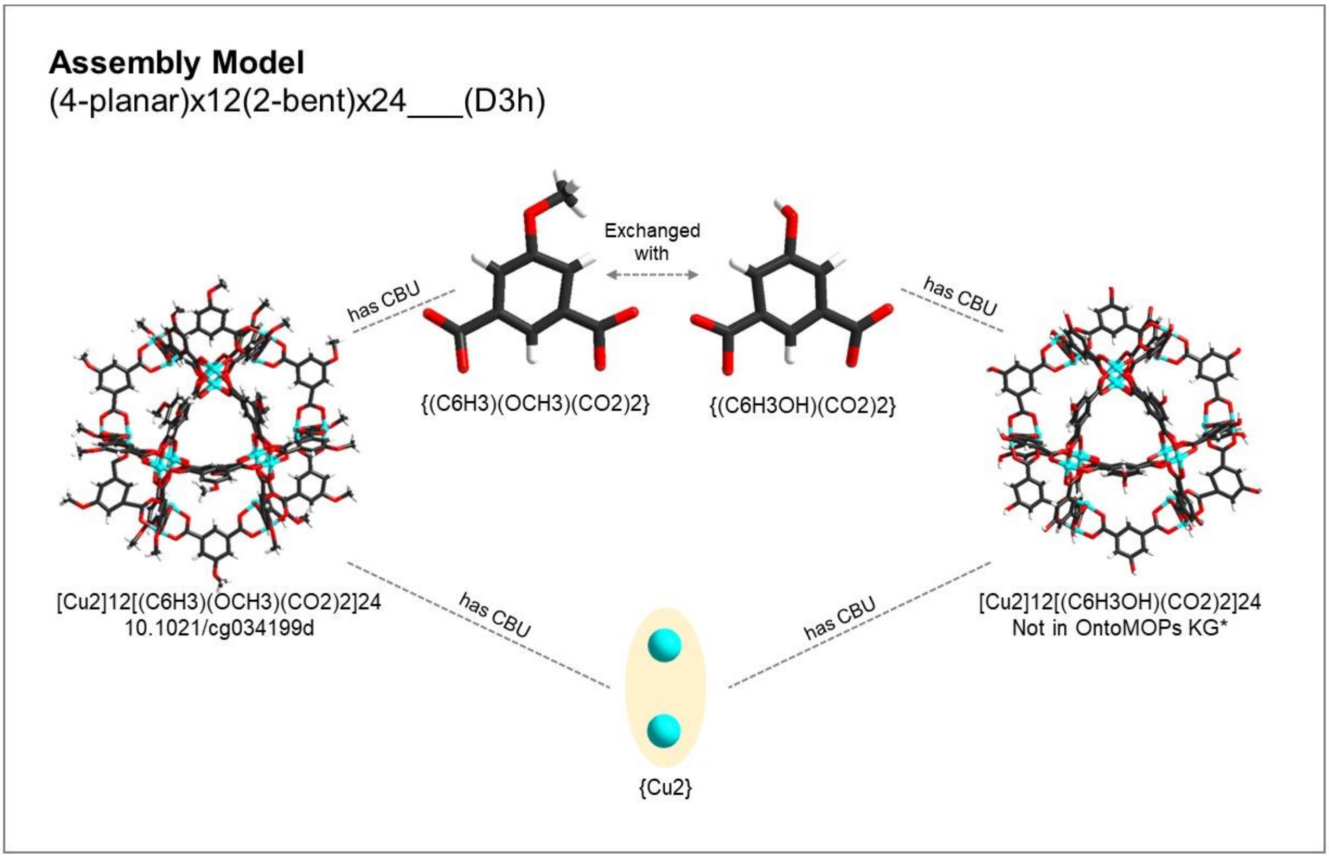

* Isomeric [Cu2]12[(C6H3OH)(CO2)2]24 built based on (4-planar)x12(2-bent)x24___(Oh) exists in the KG and has been reported in 10.1038/nchem.803

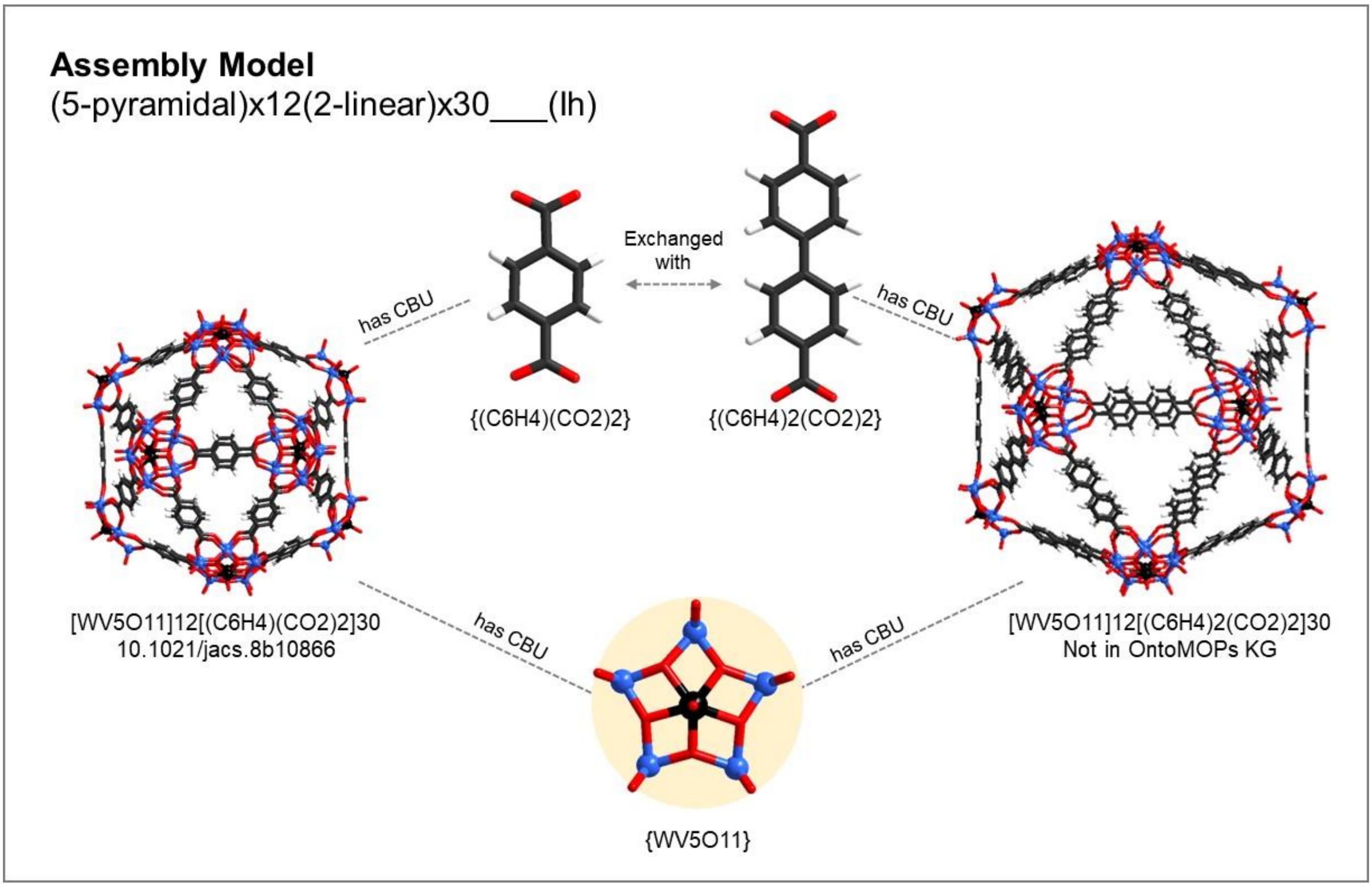
Assembly Model
(5-pyramidal)x12(2-linear)x30___(Ih)
Exchanged with
has CBU
has CBU
{(C6H4)(CO2)2}
{(C6H4)2(CO2)2}
[WV5O11]12[(C6H4)(CO2)2]30
10.1021/jacs.8b10866
has CBU
has CBU
[WV5O11]12[(C6H4)2(CO2)2]30
Not in OntoMOPs KG
{WV5O11}

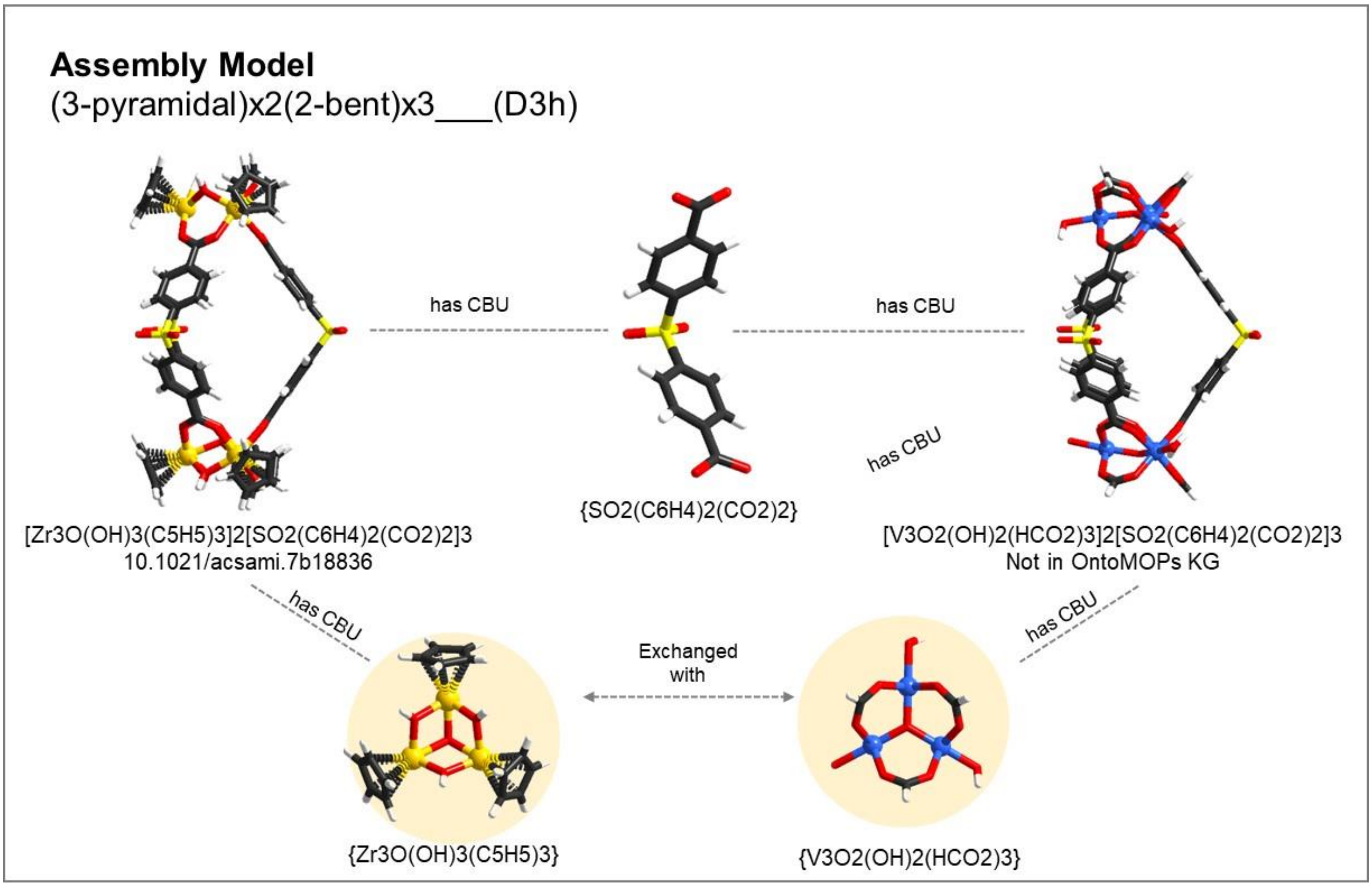
Assembly Model
(3-pyramidal)x2(2-bent)x3___(D3h)
has CBU
has CBU
has CBU
{SO2(C6H4)2(CO2)2}
[Zr3O(OH)3(C5H5)3]2[SO2(C6H4)2(CO2)2]3
10.1021/acsami.7b18836
[V3O2(OH)2(HCO2)3]2[SO2(C6H4)2(CO2)2]3
Not in OntoMOPs KG
has CBU
Exchanged with
has CBU
{Zr3O(OH)3(C5H5)3}
{V3O2(OH)2(HCO2)3}

Assembly Model
(3-planar)x4(3-pyramidal)x4___(Td)

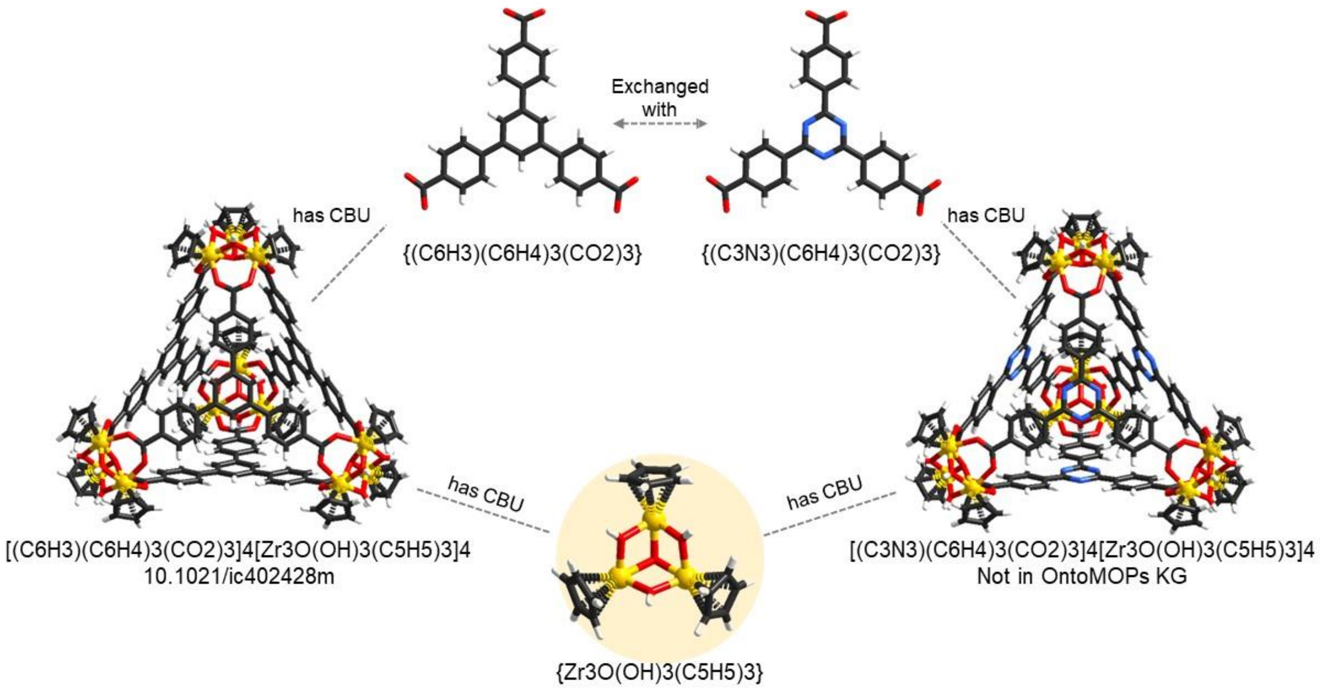

Assembly Model
(3-pyramidal)x8(2-bent)x12___(Th)

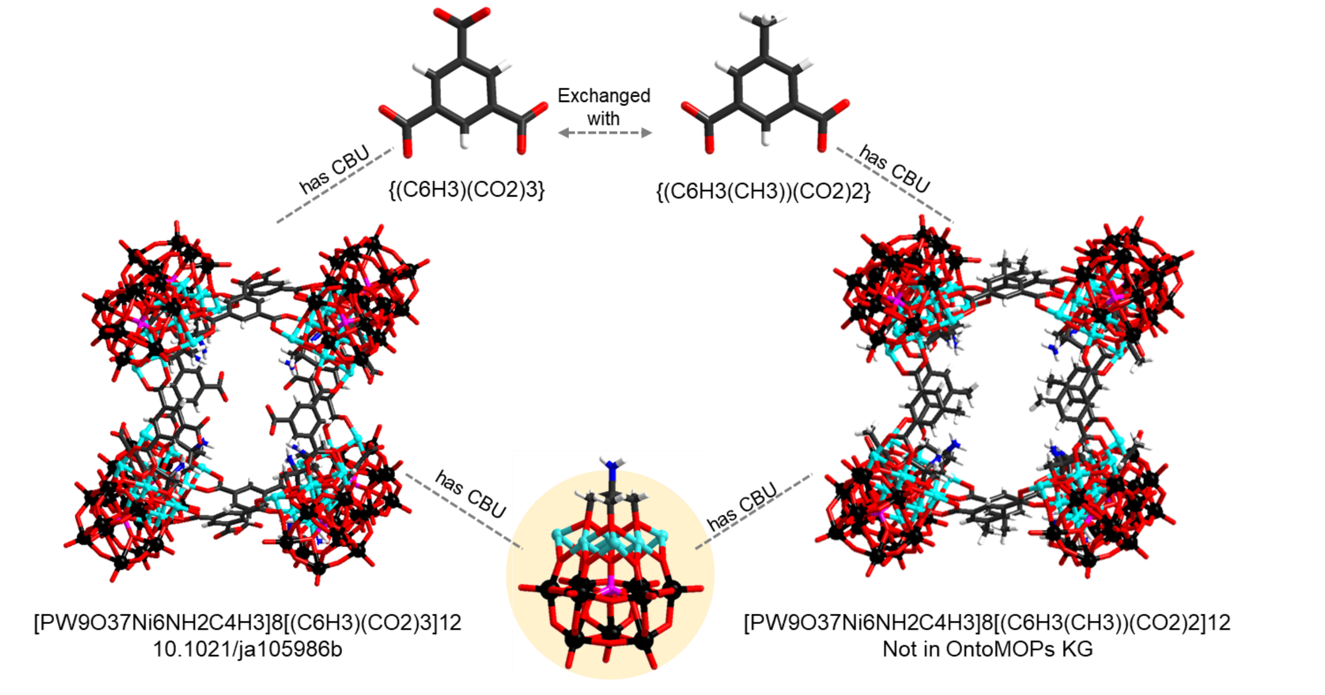

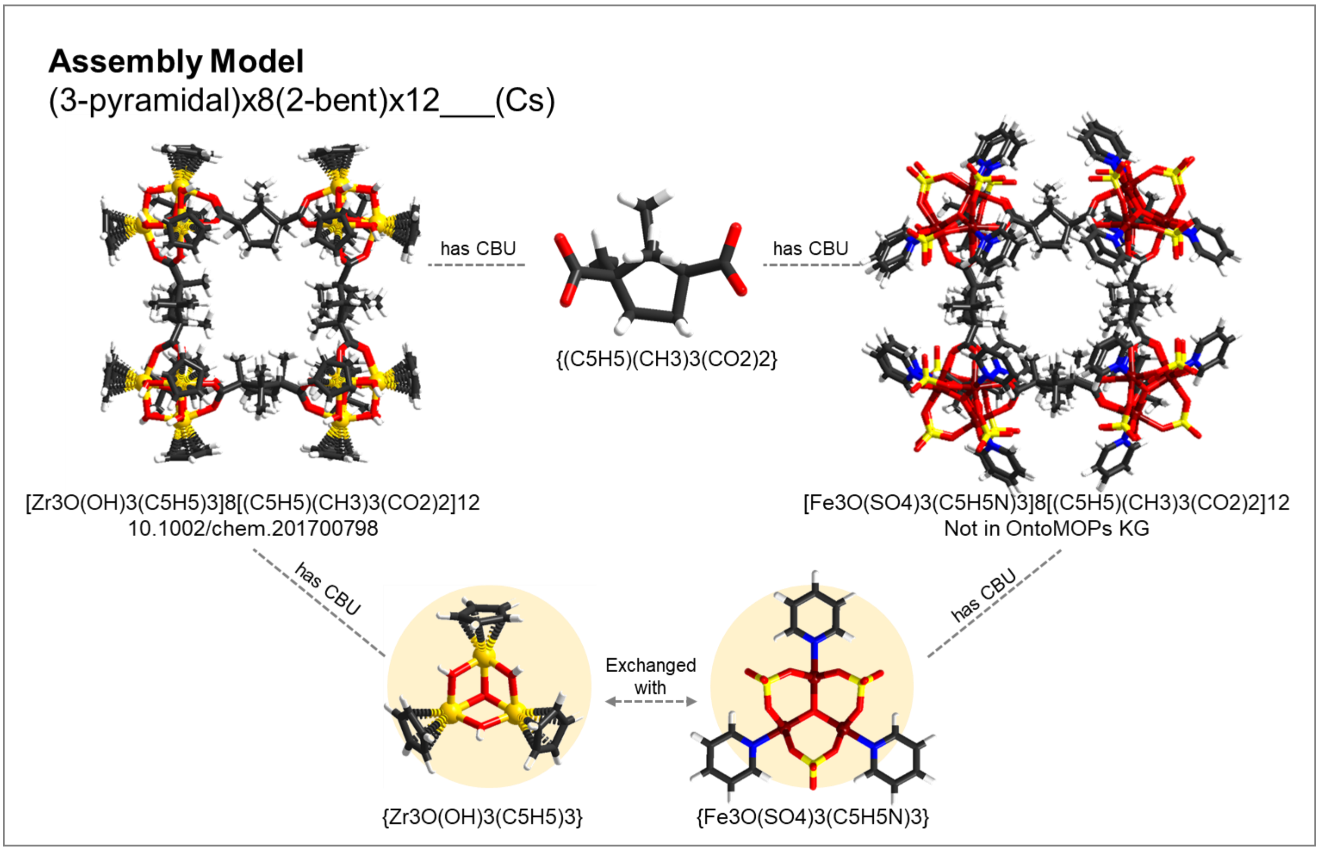
Assembly Model
(3-pyramidal)x8(2-bent)x12___(Cs)
has CBU
has CBU
{(C5H5)(CH3)3(CO2)2}
[Zr3O(OH)3(C5H5)3]8[(C5H5)(CH3)3(CO2)2]12
10.1002/chem.201700798
[Fe3O(SO4)3(C5H5N)3]8[(C5H5)(CH3)3(CO2)2]12
Not in OntoMOPs KG
has CBU
has CBU
Exchanged with
{Zr3O(OH)3(C5H5)3}
{Fe3O(SO4)3(C5H5N)3}

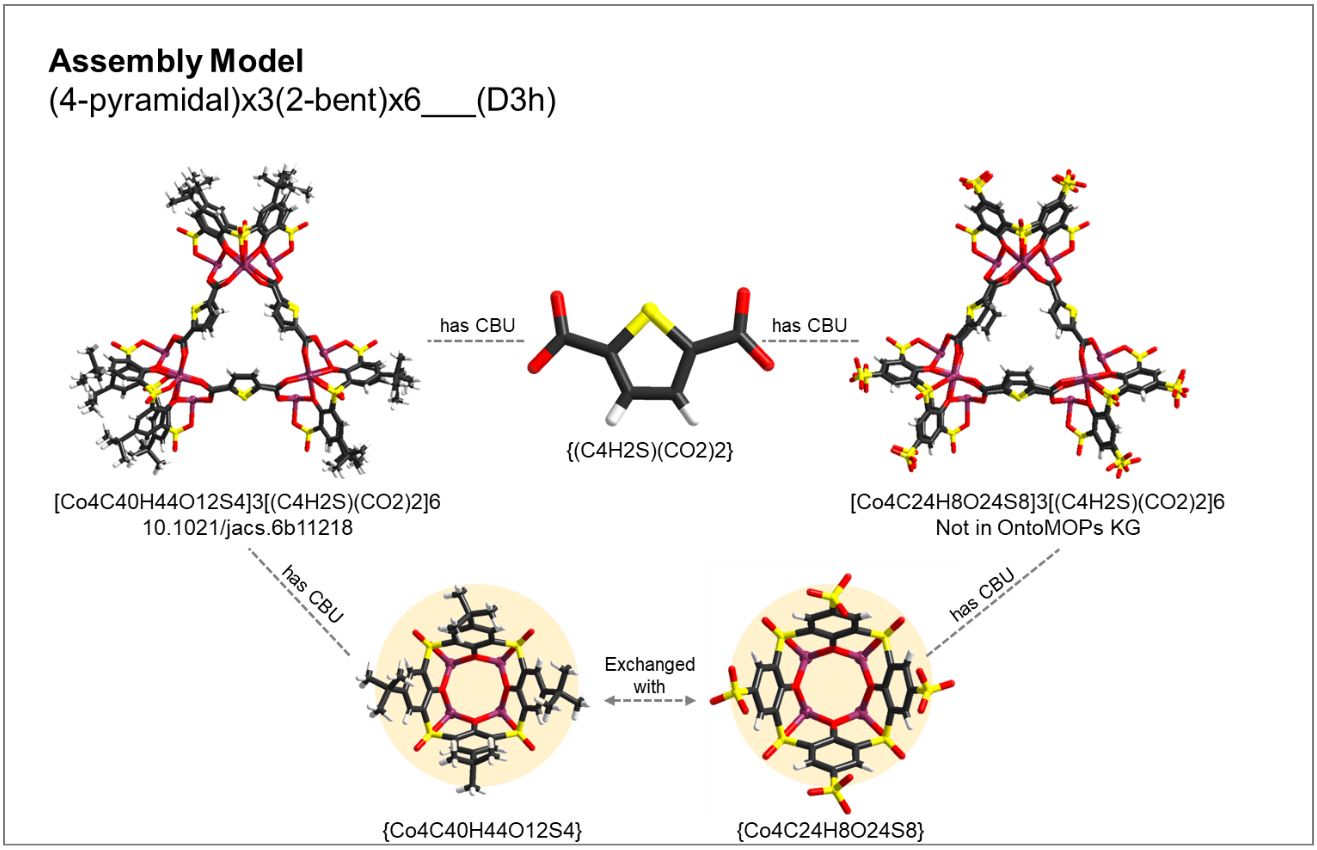
Assembly Model
(4-pyramidal)x3(2-bent)x6___(D3h)
has CBU
has CBU
{(C4H2S)(CO2)2}
[Co4C40H44O12S4]3[(C4H2S)(CO2)2]6
10.1021/jacs.6b11218
[Co4C24H8O24S8]3[(C4H2S)(CO2)2]6
Not in OntoMOPs KG
has CBU
has CBU
Exchanged with
{Co4C40H44O12S4}
{Co4C24H8O24S8}

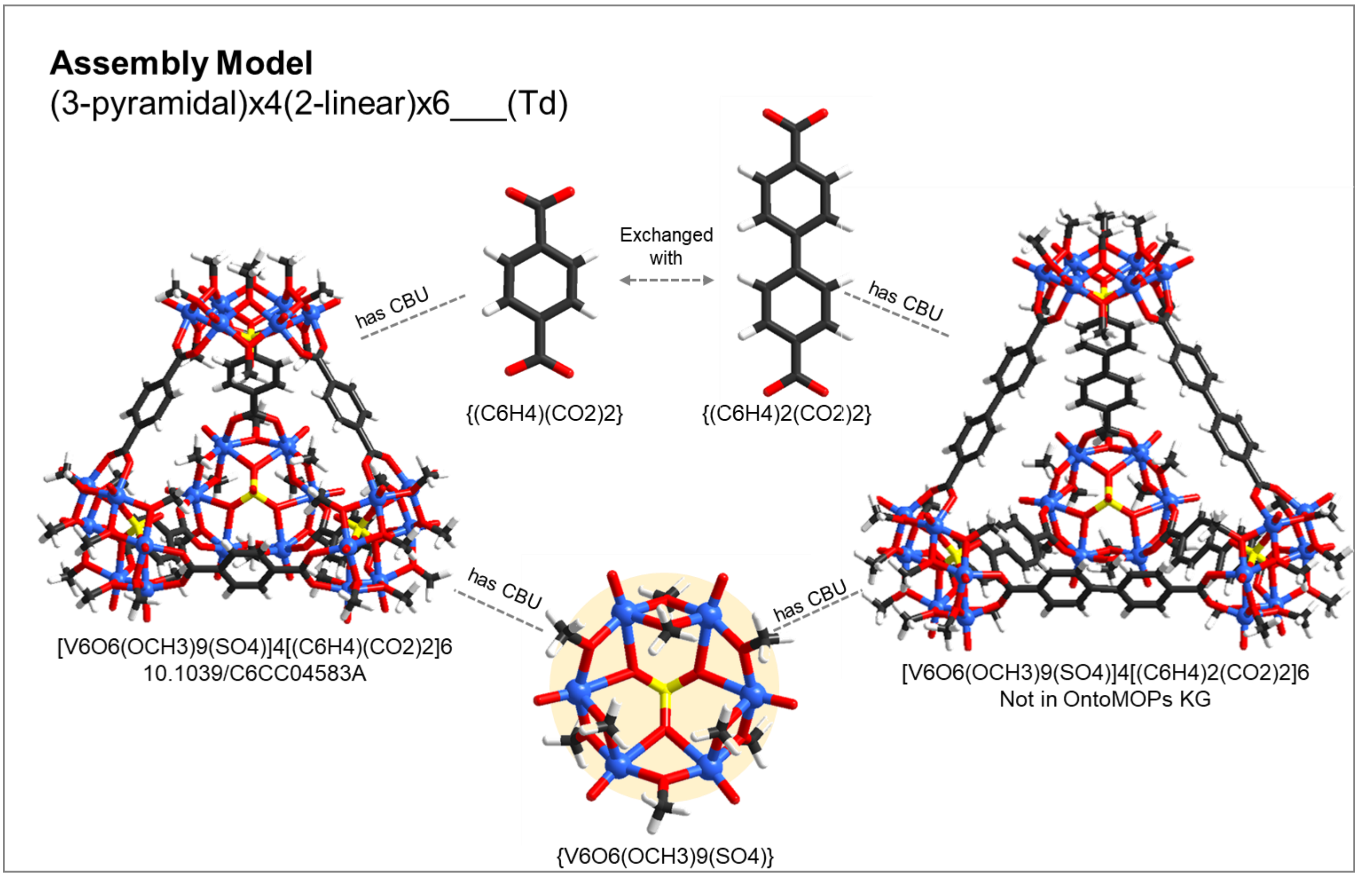

Assembly Model
(3-pyramidal)x4(2-linear)x6___(Td)
Exchanged with
has CBU
has CBU
has CBU
has CBU
{(C6H4)(CO2)2}
{(C6H4)2(CO2)2}
[V6O6(OCH3)9(SO4)]4[(C6H4)(CO2)2]6
10.1039/C6CC04583A
[V6O6(OCH3)9(SO4)]4[(C6H4)2(CO2)2]6
Not in OntoMOPs KG
{V6O6(OCH3)9(SO4)}

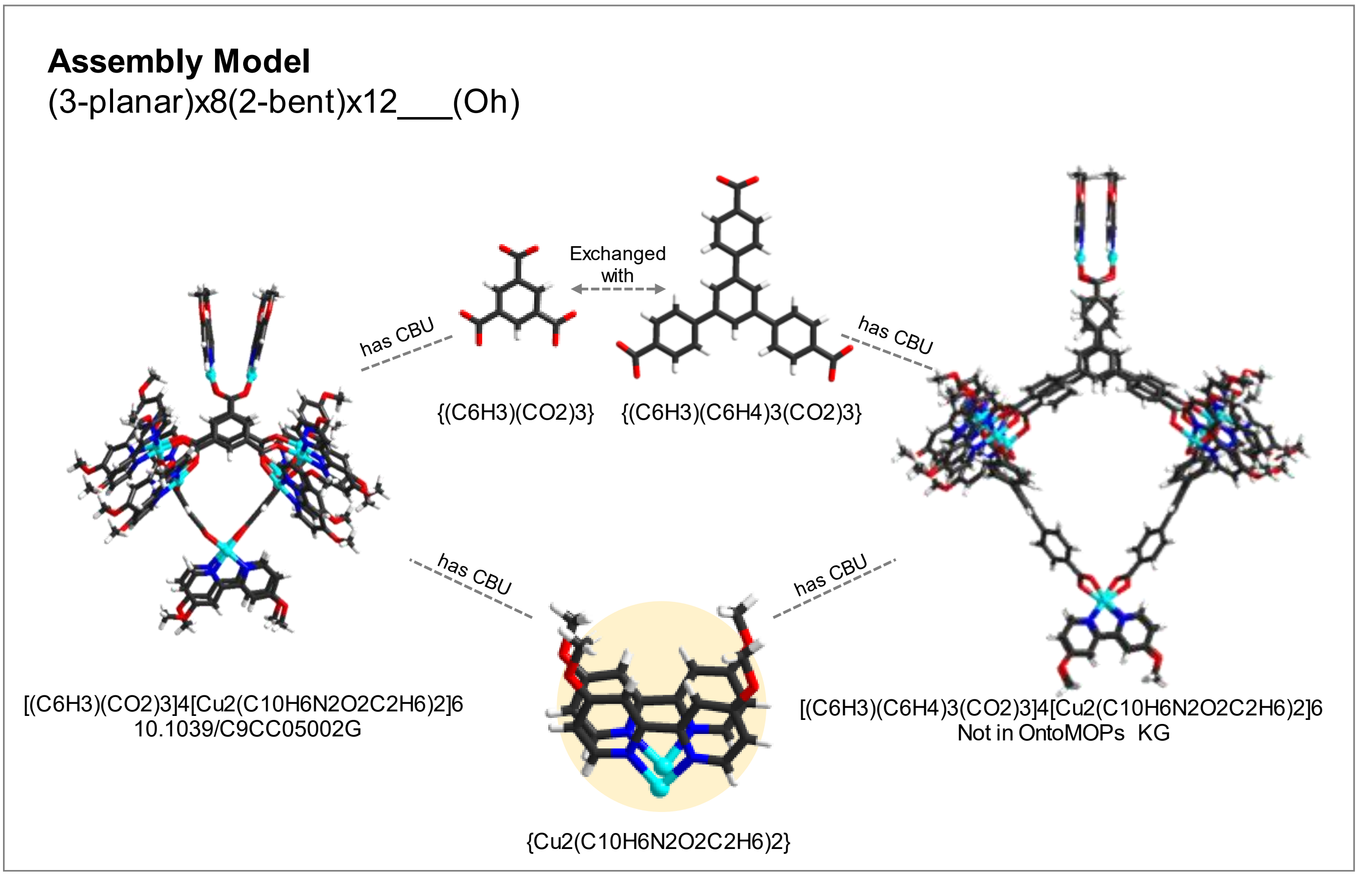

Assembly Model
(3-planar)x8(2-bent)x12___(Oh)
Exchanged with
has CBU
has CBU
has CBU
has CBU
{(C6H3)(CO2)3}
{(C6H3)(C6H4)3(CO2)3}
[(C6H3)(CO2)3]4[Cu2(C10H6N2O2C2H6)2]6
10.1039/C9CC05002G
[(C6H3)(C6H4)3(CO2)3]4[Cu2(C10H6N2O2C2H6)2]6
Not in OntoMOPs KG
{Cu2(C10H6N2O2C2H6)2}

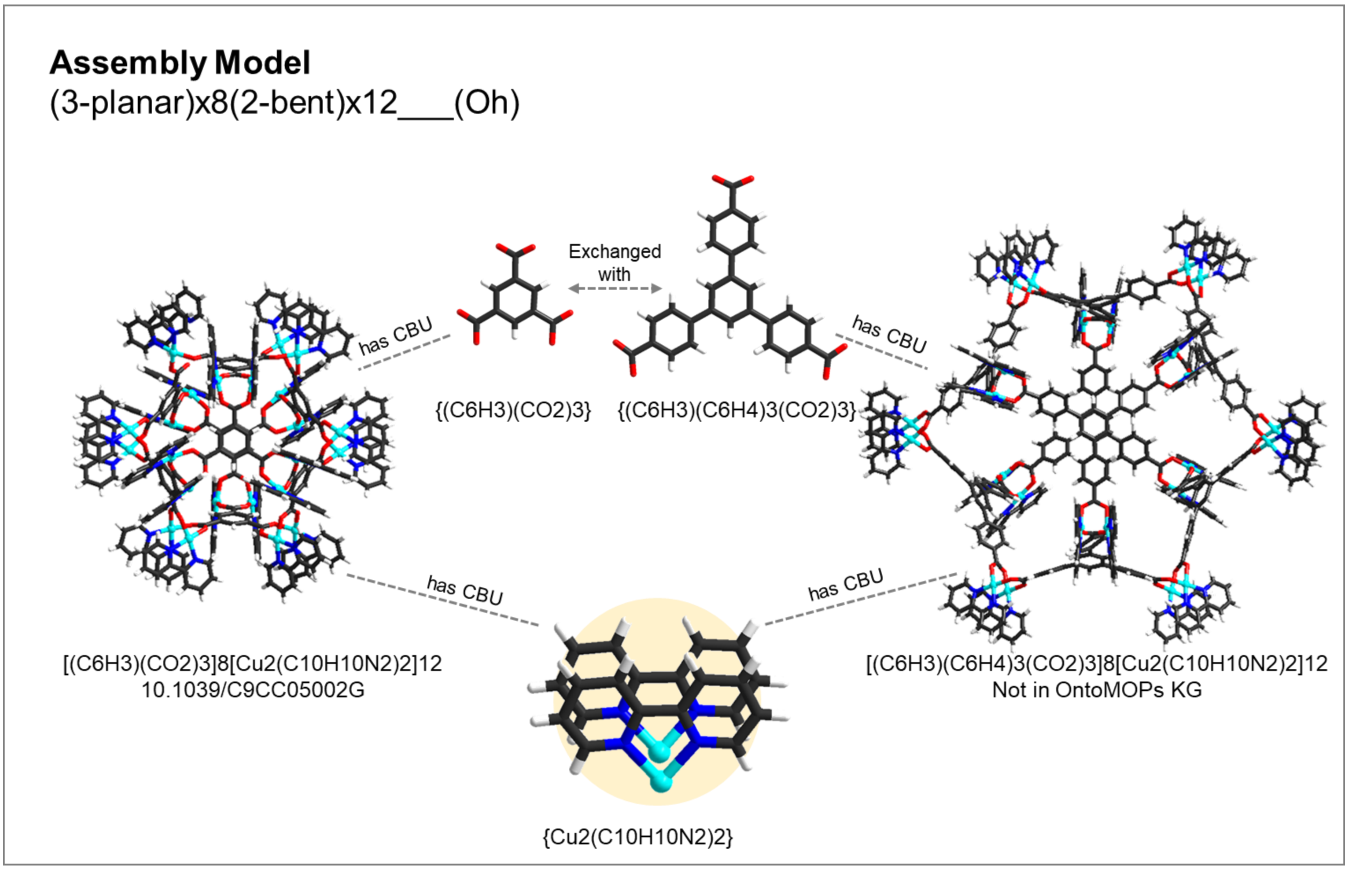
Assembly Model
(3-planar)x8(2-bent)x12___(Oh)
Exchanged with
has CBU
has CBU
{(C6H3)(CO2)3}
{(C6H3)(C6H4)3(CO2)3}
has CBU
has CBU
[(C6H3)(CO2)3]8[Cu2(C10H10N2)2]12
10.1039/C9CC05002G
[(C6H3)(C6H4)3(CO2)3]8[Cu2(C10H10N2)2]12
Not in OntoMOPs KG
{Cu2(C10H10N2)2}

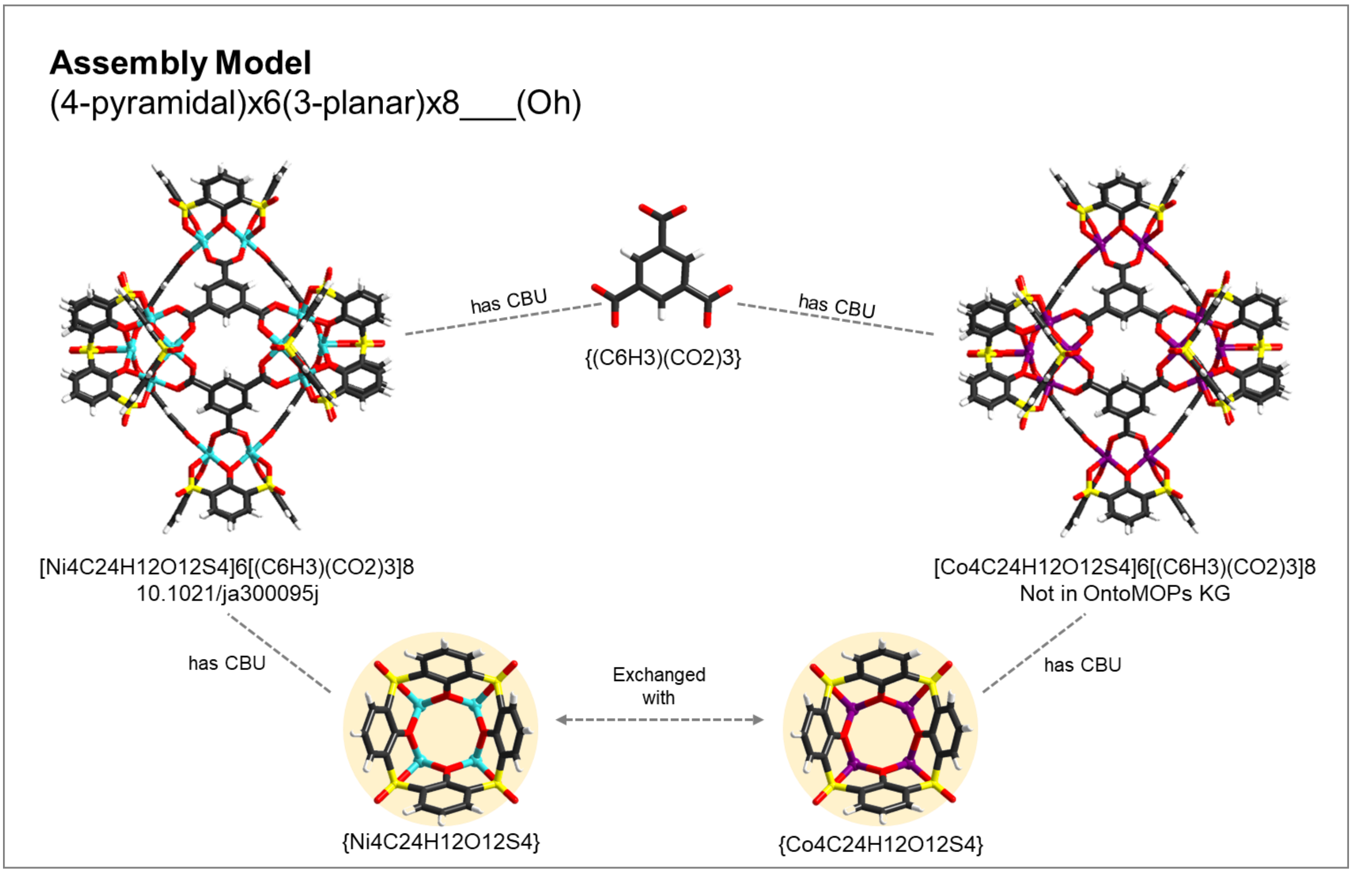
Assembly Model
(4-pyramidal)x6(3-planar)x8___(Oh)
has CBU
has CBU
{(C6H3)(CO2)3}
[Ni4C24H12O12S4]6[(C6H3)(CO2)3]8
10.1021/ja300095j
[Co4C24H12O12S4]6[(C6H3)(CO2)3]8
Not in OntoMOPs KG
has CBU
Exchanged with
has CBU
{Ni4C24H12O12S4}
{Co4C24H12O12S4}

Assembly Model
(4-pyramidal)x6(3-pyramidal)x8___(Oh)

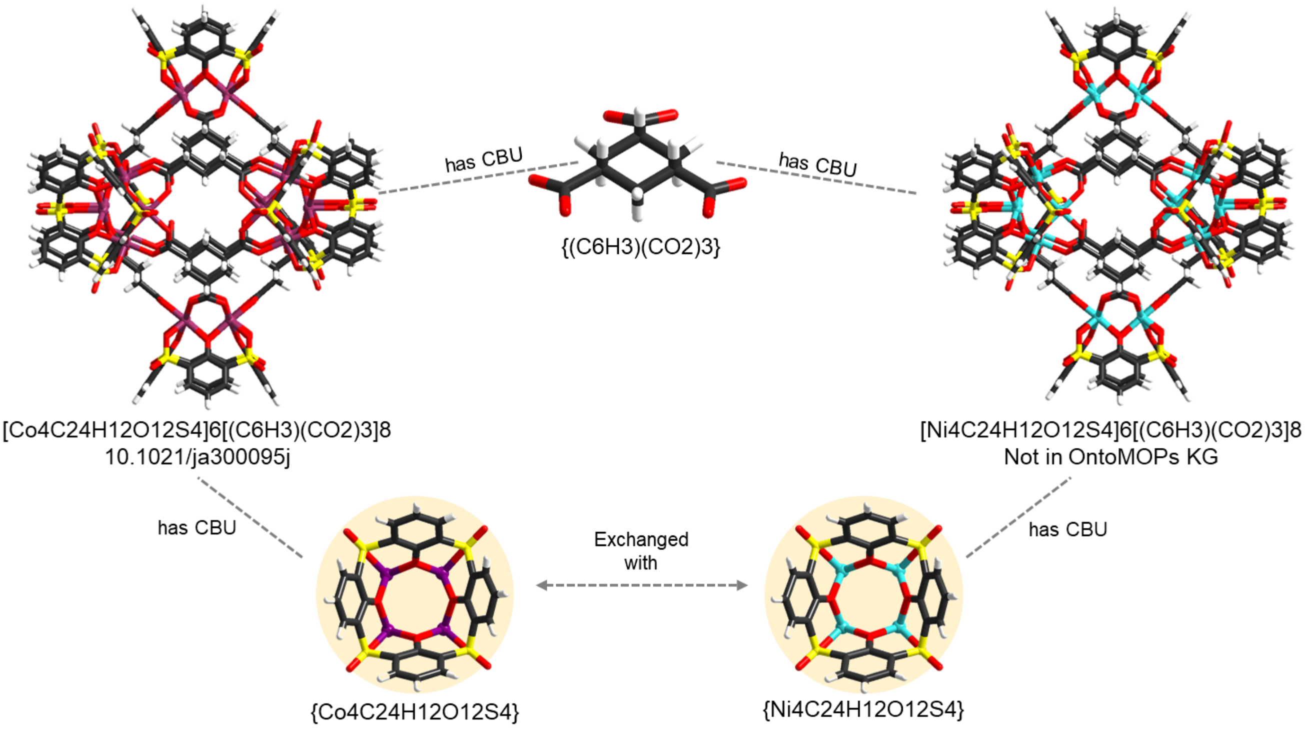

Assembly Model
(4-planar)x6(3-pyramidal)x8___(Oh)

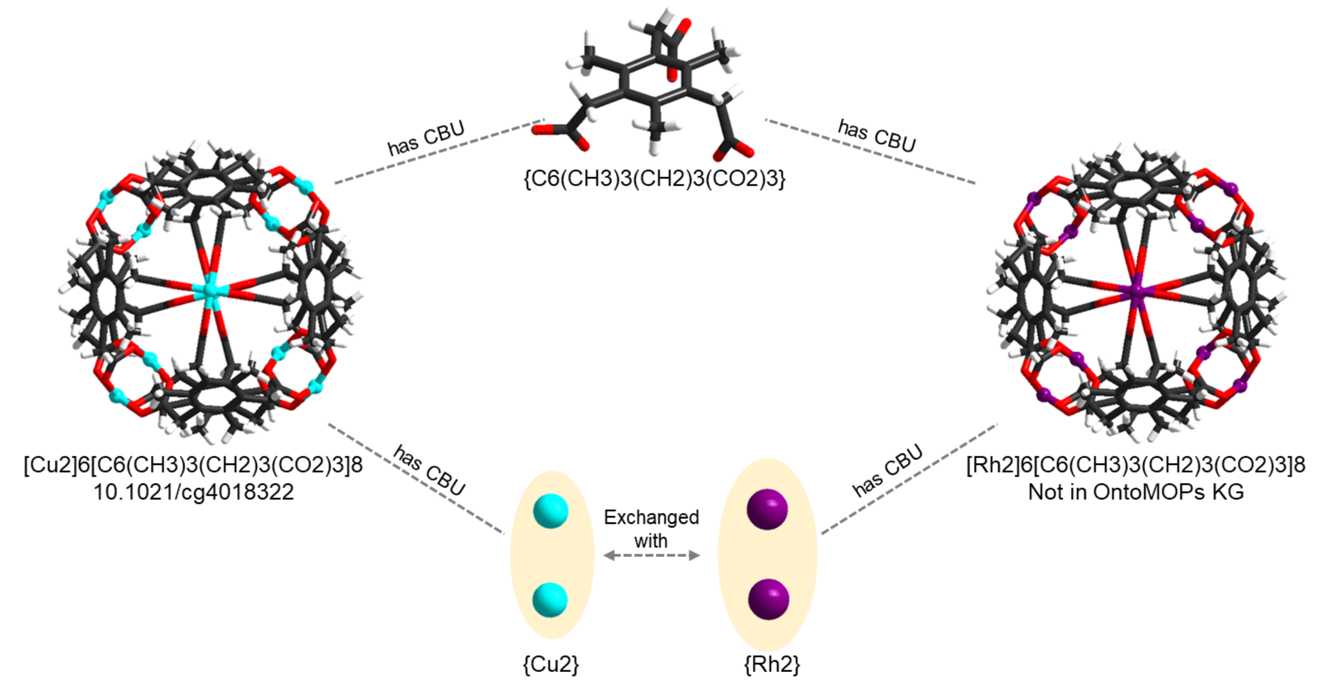

Assembly Model
(4-planar)x6(2-bent)x12___(Oh)

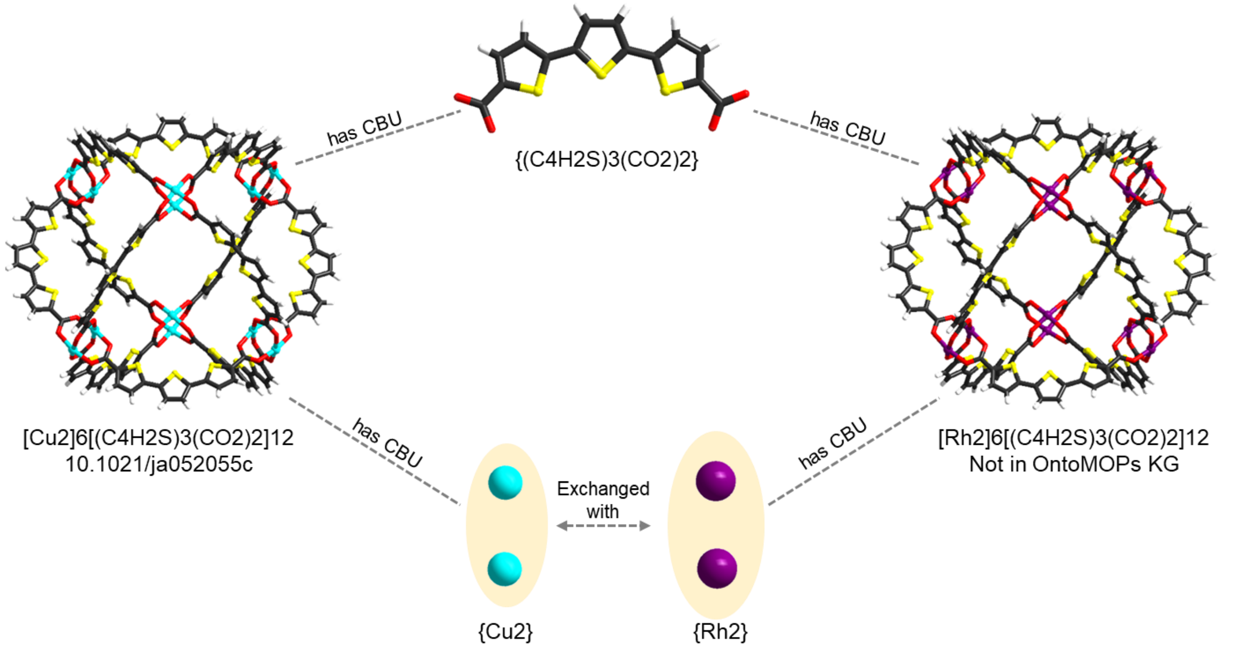

Assembly Model
(4-pyramidal)x6(2-linear)x12___(Oh)

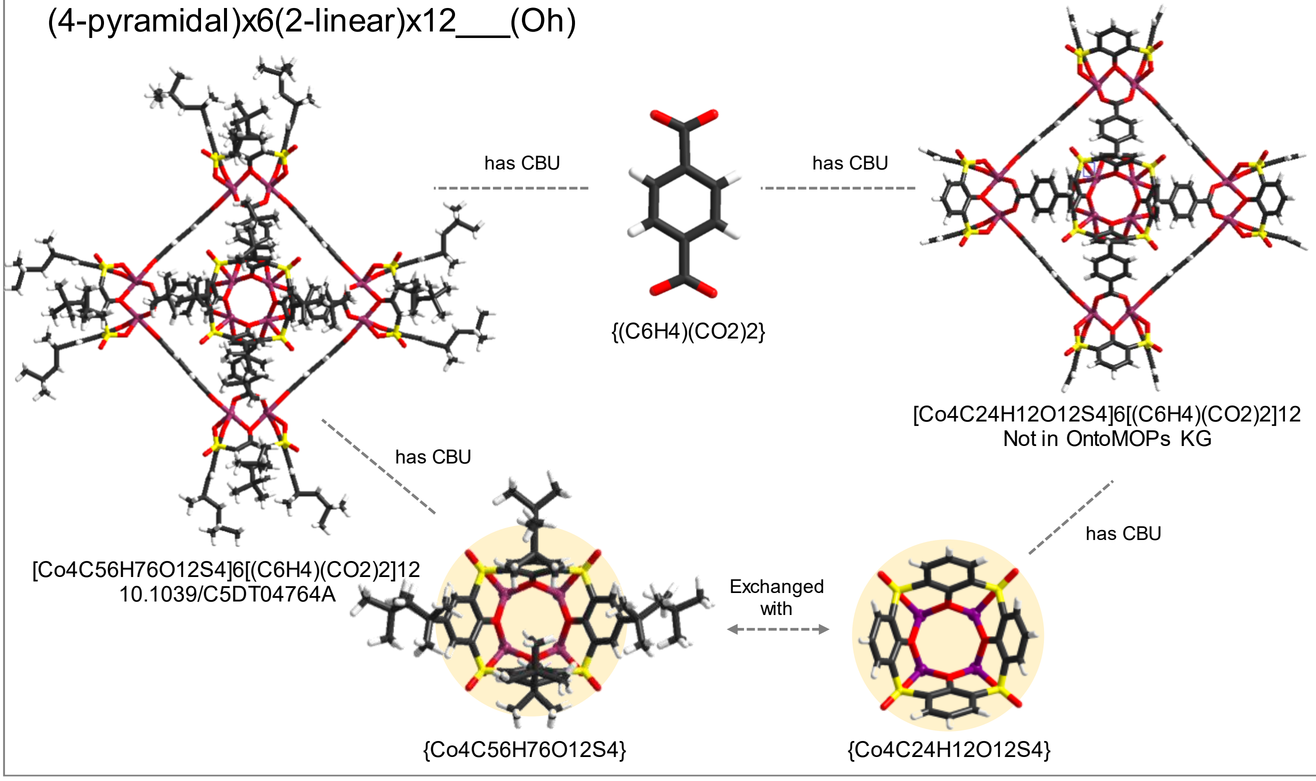

Assembly Model
(4-pyramidal)x6(2-bent)x12___(D3h)

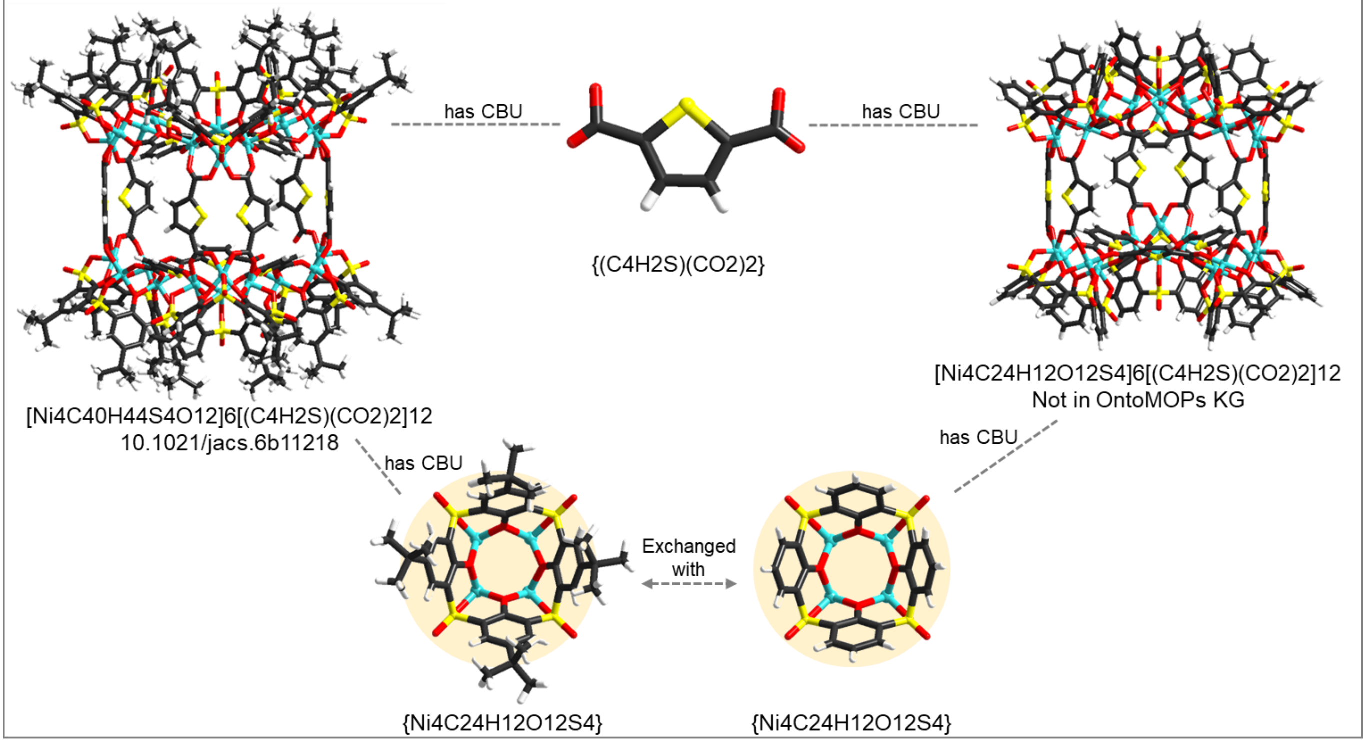

Assembly Model
(5-pyramidal)x12(3-planar)x20___(Ih)

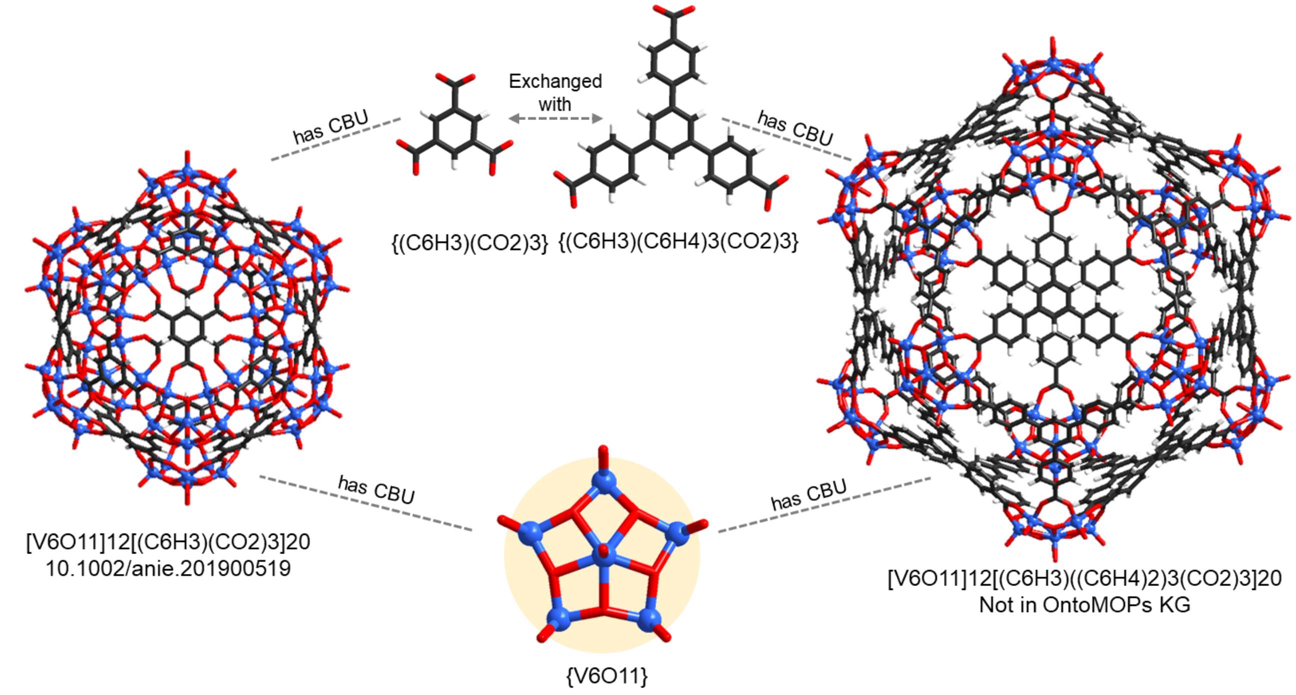

Supplement: Supplementary file 1 — ja2c03402_si_001.pdf [file ja2c03402_si_001.pdf]
